# Supplementary material for: Photoelectrochemical oxidative C(sp3)−H borylation of unactivated hydrocarbons
Source: Nat Commun. 2023 Oct 16;14:6530. doi: 10.1038/s41467-023-42264-9 (PMC10579347; doi:10.1038/s41467-023-42264-9)
Supplement: Supplementary file 1 — Supplementary Information [file 41467_2023_42264_MOESM1_ESM.pdf]

Supplementary Information for

Photoelectrochemical Oxidative C(sp^3)–H Borylation of Unactivated Hydrocarbons

Ping-Fu Zhong,[1] Jia-Lin Tu,[1] Yating Zhao,[2] Nan Zhong,[1] Chao Yang,[1] Lin Guo*[1] and Wujiong Xia*[1,3]

[1] State Key Lab of Urban Water Resource and Environment, Harbin Institute of Technology (Shenzhen), Shenzhen 518055, China

[2] College of Chemical and Material Engineering, Quzhou University, Quzhou 324000, China

[3] School of Chemistry and Chemical Engineering, Henan Normal University, Xinxiang, Henan 453007, China

* Email: guolin@hit.edu.cn; xiawj@hit.edu.cn

Table of Contents

1. General Information 3

2. Experimental Procedures 6

2.1 General procedure for preparing substrates 6

2.2. Supplementary general procedure for photoelectrochemical reaction 7

2.3. Supplementary optimization studies 9

2.4. Procedure for gram-scale reactions in batch and in continuous-flow 11

2.5. H/D scrambling and kinetic isotopic effect (KIE) experiment 15

2.6. Chlorine radical-trapping experiment 23

2.7. Divided-cell and control experiment 25

2.8. Regioselectivity studies on the reaction of 2,3-dimethylbutane (DMB) 28

2.9. C(sp^3)–H borylation in the presence of Cl_2 32

2.10. Cyclic voltammetry experiments 33

2.11. EPR studies 34

2.12. Regioselectivity studies with *n*-pentane 35

2.13. Explanation of the different results with *n*Bu_4NCl and Et_4NCl 39

2.14. Time profile of the transformation with the ON-OFF over time 40

2.15. Study on the HAT species 42

3. Product Characterization 53

4. NMR Spectra 87

5. GC Spectra 150

6. Supplementary References 161

1. General Information

Without special instructions, all reagents and solvents were commercially available and were not further purified. Column chromatography was carried out using silica gel (300-400 mesh). NMR spectroscopy was performed on a Quantum-I Plus 400 NMR spectrometer. Chemical shifts were reported in ppm. ^{1}H-NMR spectra were referenced to $CDCl_3$ (7.26 ppm), and ^{13}C-NMR spectra were referenced to $CDCl_3$ (77.0 ppm). Peak multiplicities were designated by the following abbreviations: s, singlet; d, doublet; t, triplet; m, multiplet; brs, broad singlet and *J*, coupling constant in Hz. The HR-MS spectra were recorded on a Waters Xevo G2QTOF/UPLC mass spectrometer using electrospray ionization. GC analysis was performed on 7890A-5975C/Agilent. EPR experiments were conducted using Bruker Elexsys E500 Spectrometer. Cyclic voltammograms were recorded on a CHI 770E potentiostat. The photoreaction instrument (WPP-TEC-1020SL) was purchased from WATTCAS, China. The electrochemical reactions were performed on a DJS-292B potentiostat (made in China) in constant current mode. All commercially available reagents and solvents were used as received unless otherwise specified.

Photochemical equipment and electric reactor

(1) Photochemical equipment

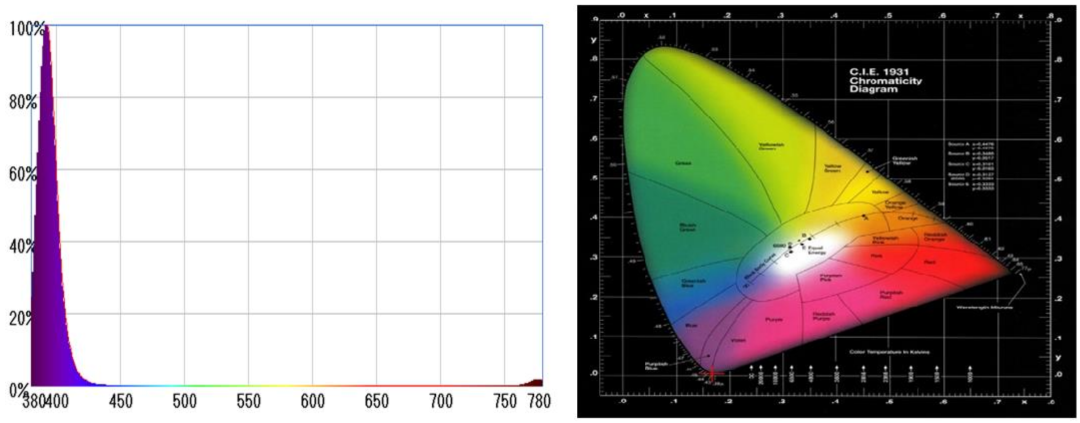

Supplementary Figure 1. Spectrophotocolormeter analysis report (from Shenzhen Learnew Optoelectronics Technology CO., LTD.)

Color Parameters：

CIE(1931:) x =0.1776 y =0.0296

CIE(1960:) u =0.2367 v =0.0592

CIE(1976:) u' =0.2367 v' =0.0888

Color Temperature：Tc=25000K Dominant Wave: WL.D=435.20nm Purity: PUR=93.54

Peak Wave: WL.P=392.5nm Delta Wave: WL.H=18.0nm

Color Tolerance: SDCM=186.7 Ra:Ra=15.0

CRI1=56.1 CRI2=16.3 CRI3=0.0 CRI4=0.0 CRI5=47.6

CRI6=0.0 CRI7=0.0 CRI8=0.0 CRI9=0.0 CRI10=0.0

CRI11=0.0 CRI12=0.0 CRI13=42.0 CRI14=6.3 CRI15=66.7

Photology Parameters:

Lum Flux: Φ(lm)=4.75lm Optical Power: Φe(mW)=2769.6mW η(lm/W)=0.4lm/W

Eletric Parameters:

Forward Voltage: VF = 22.68 V Forward Current: IF = 498.9 mA Power = 11.32 W

Status:

Wavelength Range：380nm---780nm Intergration Time ：1000 ms

Test Project：LED COB TESTING	Test Equipment：ZP OPTO SYSTEM
Product Model: HIGH POWER COB	Manufacturer: LEARNEW OPTO
Temperature:25	Humidity:40%
Tester:MESSI LAN	Time: 2019-02-20 15:01
Test Mechanism: ZP OPTO LAB	

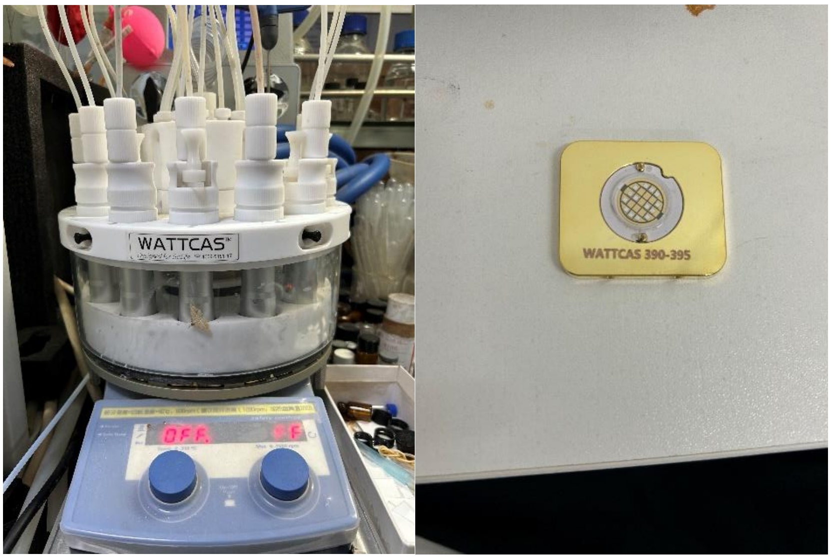

Supplementary Figure 2. Reaction setup. **Left** Photoreaction instrument (WPP-TEC-1020SL) from WATTCAS, **Right** Plank of photoreactor (390-395 nm)

(2) Electric reactor

Model: QJ3005T, Current range:0 - 5 A, Voltage range: 0 - 30 V.

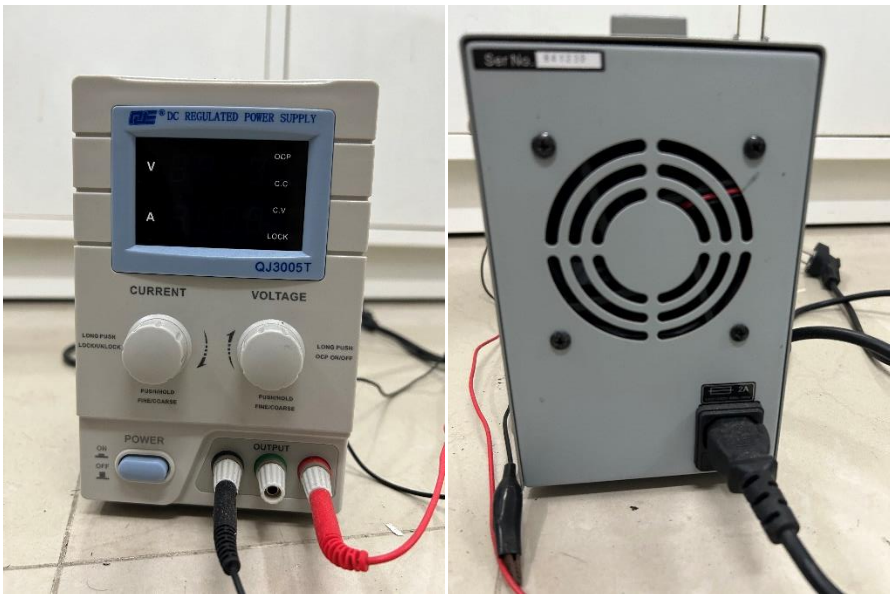

Supplementary Figure 3. Electric reactor. **Left** Frontside, **Right** Backside.

2. Experimental Procedures

2.1 General procedure for preparing substrates

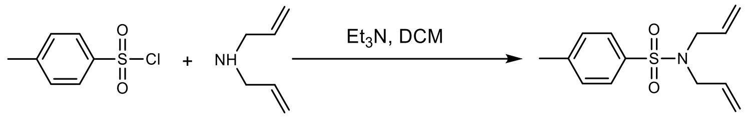

Supplementary Figure 4. Synthesis of compound **62**

According to existing literature reports,[1] in a round bottomed flask on ice, drip triethylamine (843 μL, 6 mmol, 1.2 equivalent) into the dichloromethane (20 mL) solution of diallylamine (640 μL, 6 mmol, 1.2 equivalent). This will be reflected in 0˚C at for 30 minutes. Slowly add 4-toluenesulfonyl chloride (953 mg, 5 mmol, 1 equivalent) to the reaction. Slowly raise the temperature of the reactant to room temperature and stir overnight. Wash the reactants with water and salt water. Organically dry the layer with sodium sulfate and concentrate the reaction mixture under vacuum and purify it through rapid column chromatography to obtain a transparent oil like substance. (1.2 g, 94%).

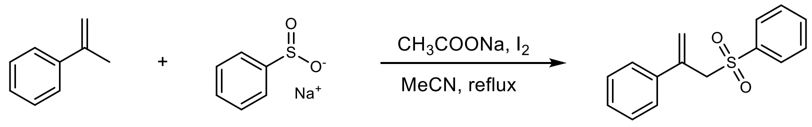

Supplementary Figure 5. Synthesis of ((2-phenylallyl)sulfonyl)benzene

According to existing literature reports,[2] to a suspension of $PhSO_2Na$ (3.79 g, 23.1 mmol) and NaOAc (947 mg, 11.6 mmol) in MeCN (30 mL), 2-phenylpropene (1.00 mL, 7.70 mmol) was added followed by I_2 (2.93 g, 11.6 mmol). The mixture was heated at reflux for 3 h before allowing to cool to rt and quenching with saturated sodium thiosulfate solution (5 mL). The mixture was basified with saturated aq. $NaHCO_3$ and extracted with EtOAc (3 × 20 mL). The combined organic phases were washed with H_2O, brine, dried over anhydrous Na_2SO_4, and concentrated in vacuo. The residue was purified by flash column chromatography to give (1.58 g, 80% yield) as a white solid.

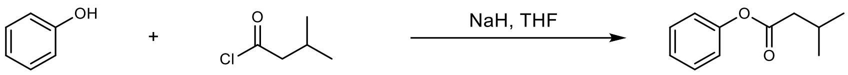

Supplementary Figure 6. Synthesis of compound **S57**

According to existing literature reports,[3] slowly add NaH (800 mg) to a THF (20 mL) flask containing phenol (1.88 g, 20 mmol), and then add isovaleryl chloride (2.4 mL, 20 mmol) dropwise. Stir the mixture overnight at room temperature. After removing the solvent, treat the residue with water and use ethyl acetate (20 mL × 3) Extraction. Dry the combined organic solvent on anhydrous Na_2SO_4. Remove the solvent and distill the residue to obtain a colorless oily substance (3.2g, 90%).

2.2. Supplementary general procedure for photoelectrochemical reaction

General Procedure A: Standard Procedure for substrates scope (alkanes, halides, and silanes)

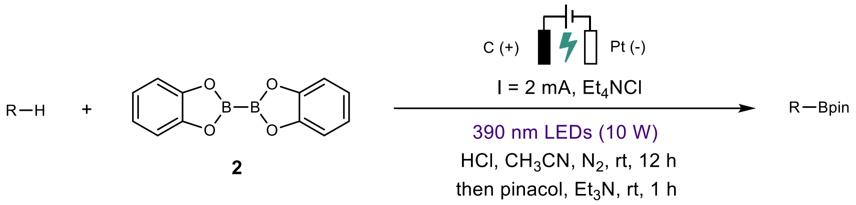

Supplementary Figure 7. Photoelectrochemical reaction of alkanes, halides, and silanes

To a 10 mL vial equipped with a stir bar was added $B_2(cat)_2$ (48 mg, 0.20 mmol, 1.0 equiv.), Et_4NCl (0.1 mmol, 0.5 equiv.), and HCl (concentrated, 0.8 mmol, 4.0 equiv.). MeCN (6 mL) was then added followed by the alkane (2.0 mmol, 10 equiv.). The reaction mixture was stirred at 680 rpm, irradiated with 390 nm LED lamps and 2.0 mA electrolysis for 12 h. The reaction temperature was maintained at approximately room temperature by cooling with a desk fan. After irradiation, a solution of pinacol (71 mg, 0.60 mmol, 3.0 equiv.) and Et_3N (0.84 mL, 6.0 mmol, 30 equiv.) in CH_2Cl_2 (1 mL) was added and stirring was continued for 1 h. The reaction mixture was concentrated in vacuo and purified by flash column chromatography to afford the corresponding boronate ester product.

General Procedure B: Standard Procedure for substrates scope (ethers, esters, and nitriles)

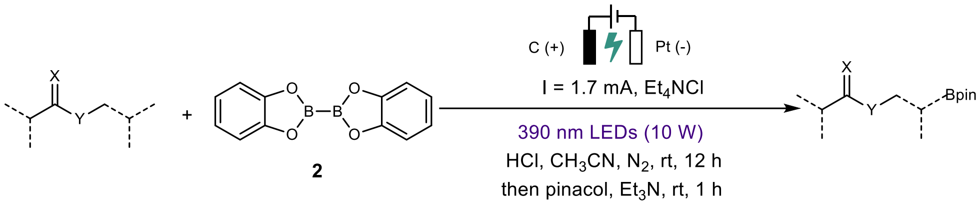

Supplementary Figure 8. Photoelectrochemical reaction of ethers, esters, and nitriles

To a 10 mL vial equipped with a stir bar was added $B_2(cat)_2$ (48 mg, 0.20 mmol, 1.0 equiv.), Et_4NCl (0.1 mmol, 0.5 equiv.), and HCl in 1,4-dioxane (0.8 mmol, 4.0 equiv.). Dry MeCN (6 mL) was then added followed by the alkane (2.0 mmol, 10 equiv.). The reaction mixture was stirred at 680 rpm, irradiated with 390 nm LED lamps and 1.7 mA electrolysis for 12 h. The reaction temperature was maintained at approximately room temperature by cooling with a desk fan. After irradiation, a solution of pinacol (71 mg, 0.60 mmol, 3.0 equiv.) and Et_3N (0.84 mL, 6.0 mmol, 30 equiv.) in CH_2Cl_2 (1 mL) was added and stirring was continued for 1 h. The reaction mixture was concentrated in vacuo and purified by flash column chromatography to afford the corresponding boronate ester product.

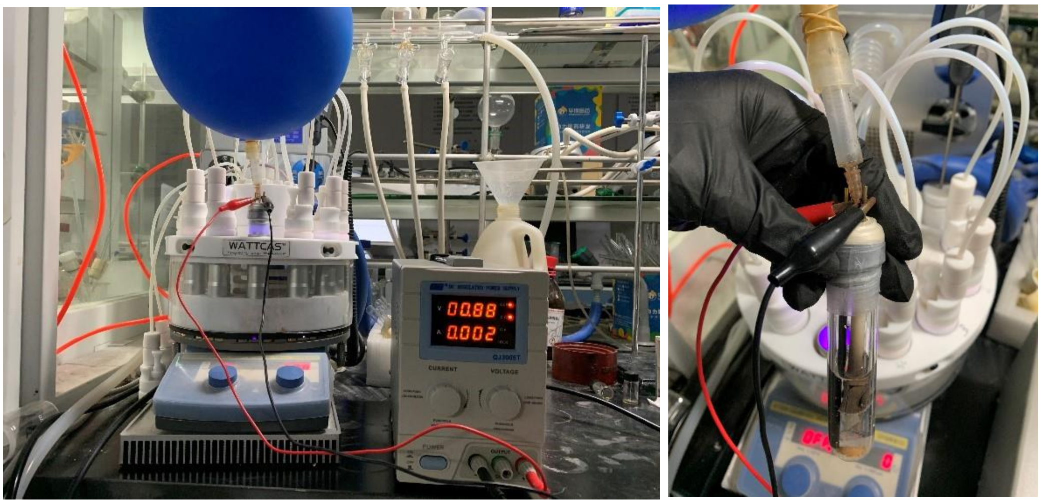

Supplementary Figure 9. Reaction setup for the photoelectrochemical reaction.

2.3. Supplementary optimization studies

Supplementary Table 1: More optimization of conditions

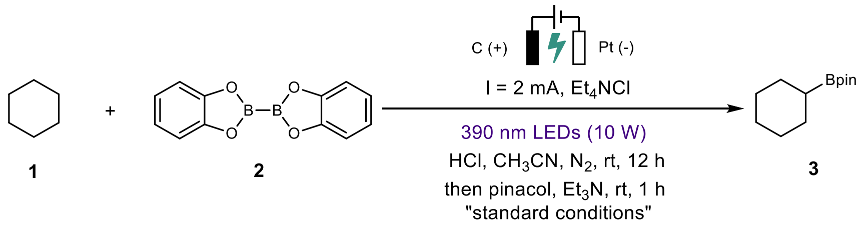

Entry	Deviation from standard conditions	Yield (%)[a]
1	Acetone instead of CH_3CN	24
2	$CHCl_3$ instead of CH_3CN	37
3	MeOH instead of CH_3CN	trace
4	EA instead of CH_3CN	trace
5	Toluene instead of CH_3CN	trace
6	Et_4NBF_4 instead of Et_4NCl	62
7	*n*Bu_4NBF_4 instead of Et_4NCl	trace
8	2 equiv HCl	56
9	6 equiv HCl	60
10	5 equiv **1**	58
11	20 equiv **1**	73

[a] Yields determined by NMR analysis using 1,3,5-$(OMe)_3C_6H_3$ as the internal standard.

Supplementary Table 2: Screening different solvents

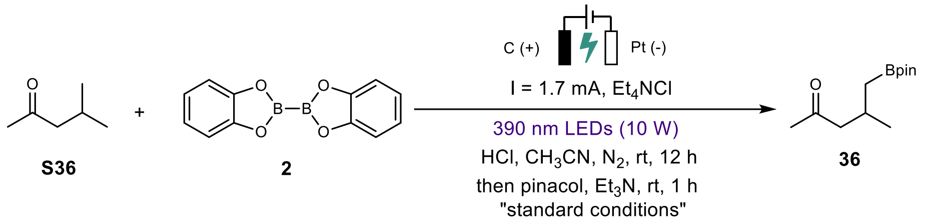

Entry	Deviation from standard conditions	Yield (%)[a]
1	none	29
2	$CHCl_3$ instead of HCl	35
3	HCl in EA	49
4	HCl in CH_3CH_2OH	54
5	HCl in 1,4-Dioxane	65

[a] Yields determined by NMR analysis using 1,3,5-$(OMe)_3C_6H_3$ as the internal standard.

Supplementary Table 3: Screening different currents

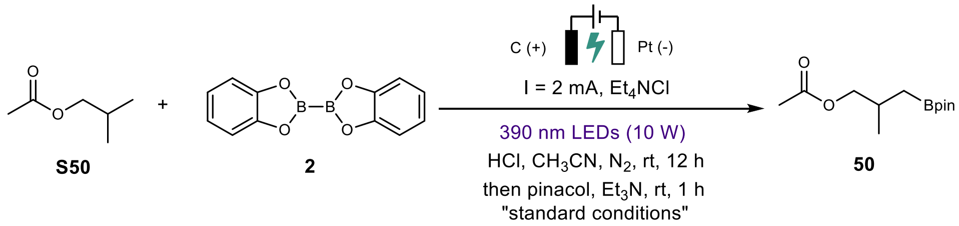

Entry	Deviation from standard conditions	Yield (%)[a]
1	none	trace
2	1.9 mA	12
3	1.8 mA	32
4	1.7 mA	56[c]
5	1.6 mA	45

[a] Yields determined by NMR analysis using 1,3,5-$(OMe)_3C_6H_3$ as the internal standard.

2.4. Procedure for gram-scale reactions in batch and in continuous-flow

(1) Procedure for gram-scale reaction in batch

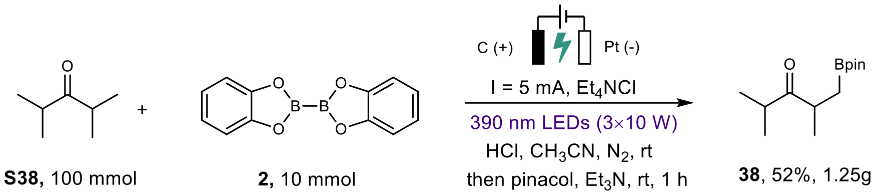

Supplementary Figure 10. Gram-scale reaction in batch

The 10 mmol scale synthesis of **38** was conducted in a 50mL cell with two pieces of C (φ = 6 mm) as the anode, a Pt plate cathode (10 x 10 x 0.1 mm), and a constant current of 5 mA. Four 30 W LEDs (390 nm) are placed evenly around the reactor. The reaction mixture consisted **2** (2.38 g, 10.0 mmol), Et_4NCl (0.84 g, 5 mmol), HCl (in 1,4-Dioxane, 5.0 mL, 20 mmol), **S38** (14 mL, 100 mmol), MeCN (25 mL). The reaction time was 24 h and the internal temperature was reaction temperature. After irradiation, a solution of pinacol (3.54 g, 30 mmol, 3.0 equiv.) and Et_3N (20.2 mL, 200 mmol, 20 equiv.) in CH_2Cl_2 (10 mL) was added and stirring was continued for 1 h. The reaction mixture was concentrated in vacuo and then purified by flash column chromatography (10% EtOAc/henane) to give **38** (1.25 g, 5.2 mmol, 52%) as a colourless oil.

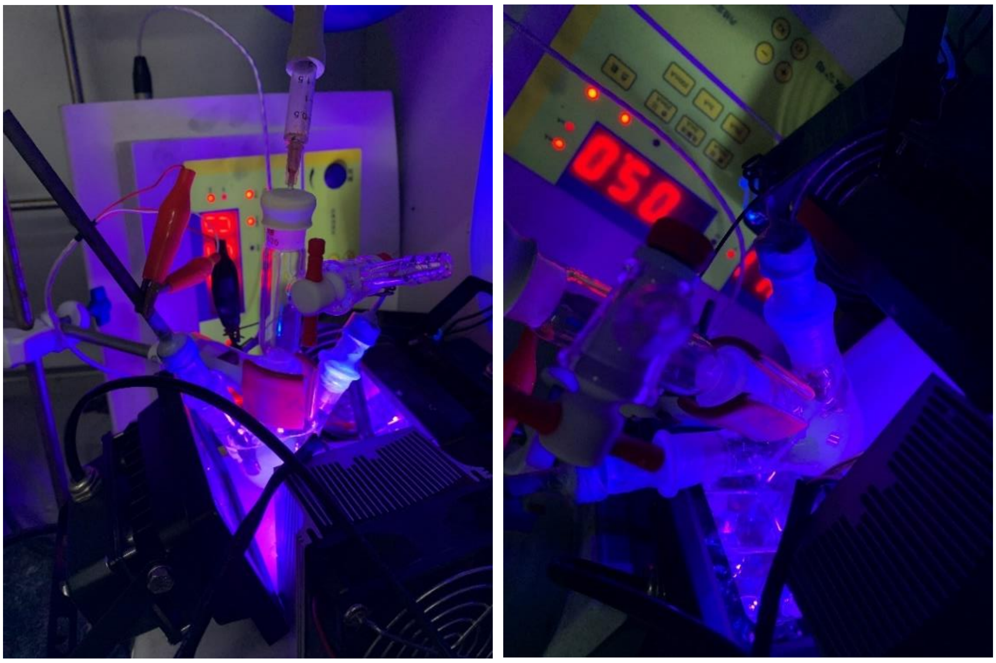

Supplementary Figure 11. Reaction setup for the gram scale reaction in batch

(2) Procedure for gram-scale reaction in continuous flow

Note: The design is shown in Supplementary Figure 12. The flow electrolysis cell is assembled using two aluminum bodies (① and ⑤, 120 mm x 110 mm x 8 mm) with a groove (70 mm x 60 mm x 8 mm). The main material of ② is translucent quartz glass (70 mm x 60 mm x 3 mm). ③ is a reaction module with internal reaction electrodes (⑥ With grooves, 70 mm x 60 mm x 5 mm), and the external material is mainly polytetrafluoroethylene. ④ is a Graphene module with grooves (Thickness is 10 mm). flow reactor: 120 * 110 * 50mm.

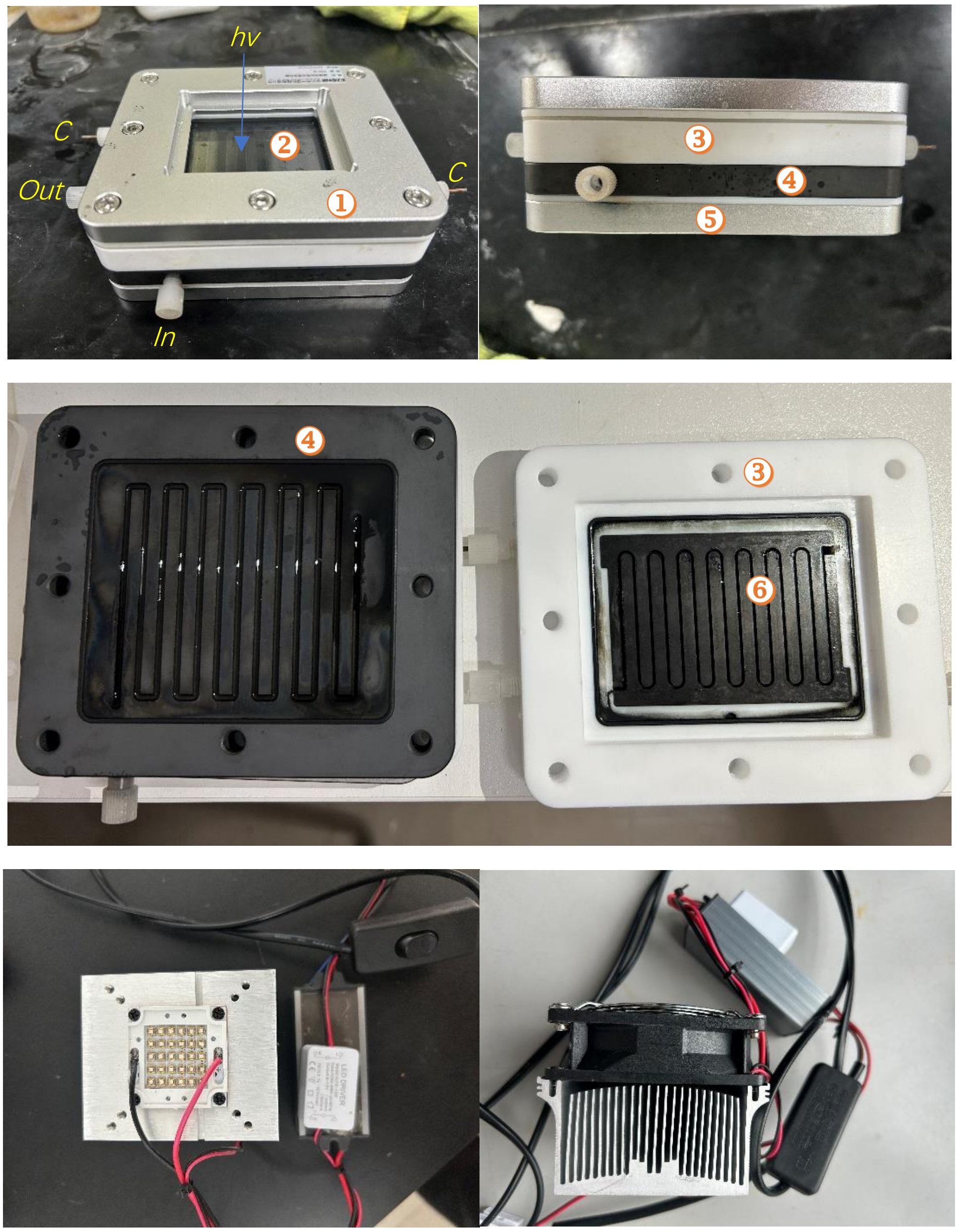

Supplementary Figure 12. Flow reactor

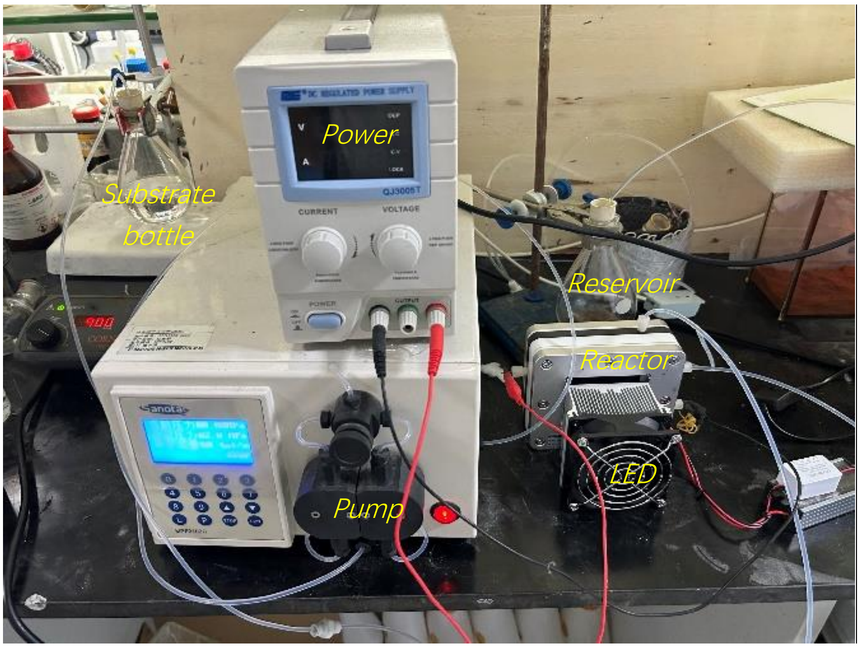

Supplementary Figure 13. Flow reaction device setup

To ensure that the system is in an oxygen free state, a pump is first used to fill the entire pipeline with acetonitrile in a nitrogen atmosphere, during which a high flow rate is required to eliminate bubbles. Then place the degassed reaction bottle into the reactor. Electrolysis is a Electrolytic cell equipped with graphite anode and graphite cathode. The constant current is 10 mA, the volume is 6 cm^3, and the distance between electrodes is 2 mm. Use a pump to flow through the Electrolytic cell at a flow rate of 0.1 mL/min. Use a dry Round-bottom flask at the outlet to collect the reaction liquid, and then use Pinacol and triethylamine for post-treatment.

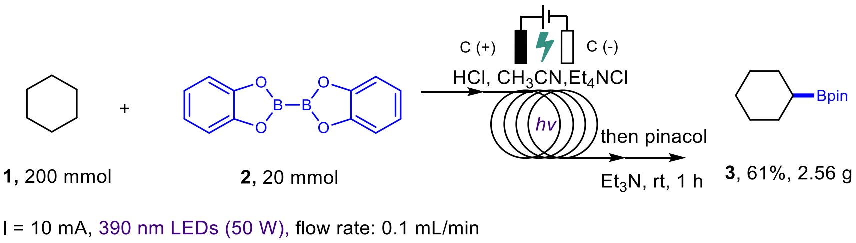

Supplementary Figure 14. Gram-scale reaction in continuous-flow (substrate **1**)

By applying a flow rate of 0.1 mL min^{-1}, an oven dried 250 mL round bottomed flask was charged with a stir bar. The reaction mixture consisted **2** (7.76 g, 20.0 mmol), Et_4NCl (1.68 g, 10 mmol), HCl (concentrated, 5.0 mL, 60 mmol), **1** (21.6 mL, 200

mmol), MeCN (150 mL). The reaction mixture was stirred at 900 rpm, irradiated with 390 nm LED (50 W) lamps and 10.0 mA electrolysis. Use a fan to blow to approximately room temperature. After irradiation, a solution of pinacol (7.08 g, 60 mmol, 3.0 equiv.) and Et_3N (40.4 mL, 400 mmol, 20 equiv.) in CH_2Cl_2 (20 mL) was added and stirring was continued for 1 h. The reaction mixture was concentrated in vacuo and then purified by flash column chromatography (10% EtOAc/henane) to give **3** (2.56 g, 12.2 mmol, 61%) as a colourless oil.

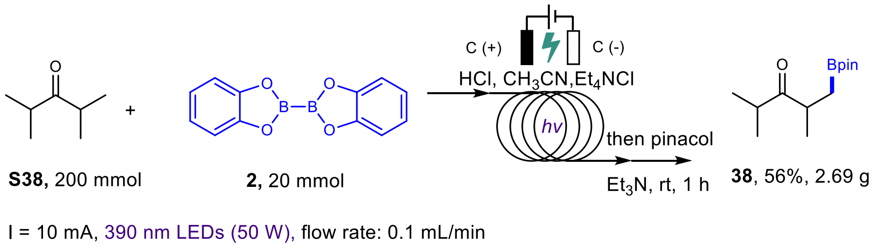

Supplementary Figure 15. Gram-scale reaction in continuous-flow (substrate **S38**)

By applying a flow rate of 0.1 mL min^{-1}, an oven dried 250 mL round bottomed flask was charged with a stir bar. The reaction mixture consisted **2** (7.76 g, 20.0 mmol), Et_4NCl (1.68 g, 10 mmol), HCl (in 1,4-Dioxane, 15.0 mL, 60 mmol), **S38** (28 mL, 200 mmol), MeCN (150 mL). The reaction mixture was stirred at 900 rpm, irradiated with 390 nm LED (50 W) lamps and 10.0 mA electrolysis. Use a fan to blow to approximately room temperature. After irradiation, a solution of pinacol (7.08 g, 60 mmol, 3.0 equiv.) and Et_3N (40.4 mL, 400 mmol, 20 equiv.) in CH_2Cl_2 (20 mL) was added and stirring was continued for 1 h. The reaction mixture was concentrated in vacuo and then purified by flash column chromatography (10% EtOAc/henane) to give **38** (2.69 g, 11.2 mmol, 56%) as a colourless oil.

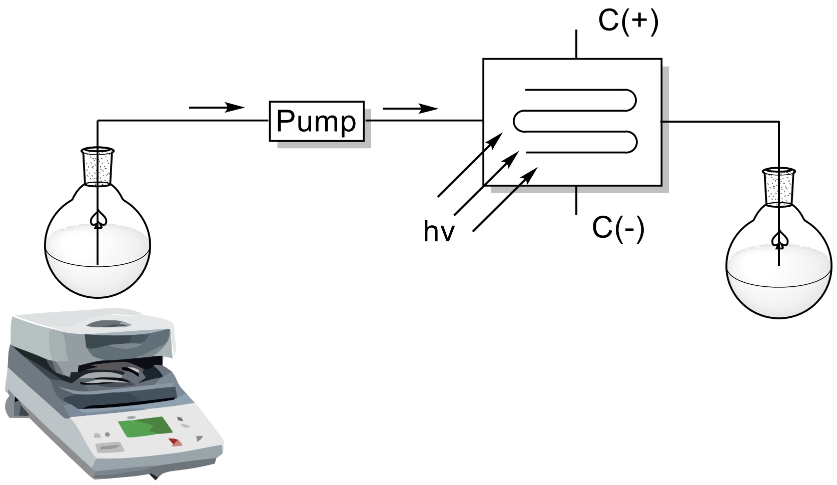

Supplementary Figure 16. Design of the flow reactor for the gram-scale reaction

2.5. H/D scrambling and kinetic isotopic effect (KIE) experiment

(1) H/D scrambling

According to existing literature reports, Cl radicals undergo HAT based on their inherent selectivity (tertiary > secondary > primary), but more substituted radicals react slower with certain electrophilic reagents, resulting in faster functionalization of primary radicals. This means that the formation of alkyl radicals is reversible (or alkyl can be "isomerized" by subsequent reversible HAT with other molecules of the substrate) and rapid (consistent with KIE value 1). We validated this conclusion through H/D scrambling experiments. The following three deuterated compounds (**S55-*d***, **S56-*d***, and **S57-*d***) were synthesized with moderate to high levels of deuterium incorporation at the tertiary sites, based on the method developed by Wu and colleagues (*Chem. Sci*. **2020**, *11*, 8912).

Procedure: Substrate (4.0 mmol), TBADT (0.26 g, 2 mol%), thiol (100 uL, 10 mol%), D_2O (7.2 mL, 100equiv), TBAB (0.26 g, 20 mol%) and CH_3CN (4 mL, 1.0 M) were added to a Schlenk tube (25 mL) equipped with a stir bar. The mixture was operated by freeze-pump-thaw procedures three times before being charged with argon. The reactor was placed under 390 nm Kessil light (80 W) and kept stirring for 24 h. the crude reaction mixture was extracted with diethyl ether (3 × 8 mL). The combined organic

layer was concentrated and purified by flash column chromatography over silica gel to afford the deuterated product.

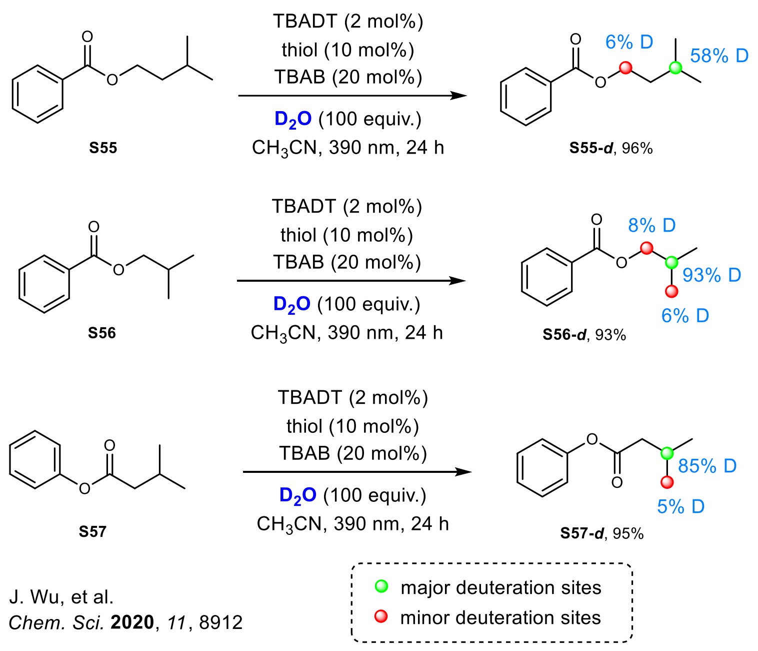

Supplementary Figure 17. Synthesis of deuterated substrates

We chose **S55-d** (with 58% deuterium incorporation at the tertiary site), **S56-d** (with 93% deuterium incorporation at the tertiary site), and **S57-d** (with 85% deuterium incorporation at the tertiary site) as suitable substrates for the H/D scrambling experiment. As shown in the following scheme, substrate **S55-d** undergoes C(sp^3)–H borylation reaction under the standard conditions to obtain the corresponding borylated product **55-d** in 52% yield. As expected, C(sp^3)–H borylation only occurred at the terminal methyl group and significant H/D perturbations were observed, with only 23% D-incorporation at the tertiary site (35% lower than the original D-incorporation). The reaction of **S56-d** as substrate achieved similar results (36% lower than the original D-incorporation at the tertiary site), and the reaction of **S57-d** also achieved similar results (27% lower than the original D-incorporation at the tertiary site). All these results indicate that the reaction can preferentially form tertiary carbon radicals at first. Due to the slower reactivity of these more substituted radicals with certain radical acceptors, the primary carbon radicals are subsequently generated through a reversible HAT process and then react with $B_2(cat)_2$ to achieve the unconventional site selectivity.

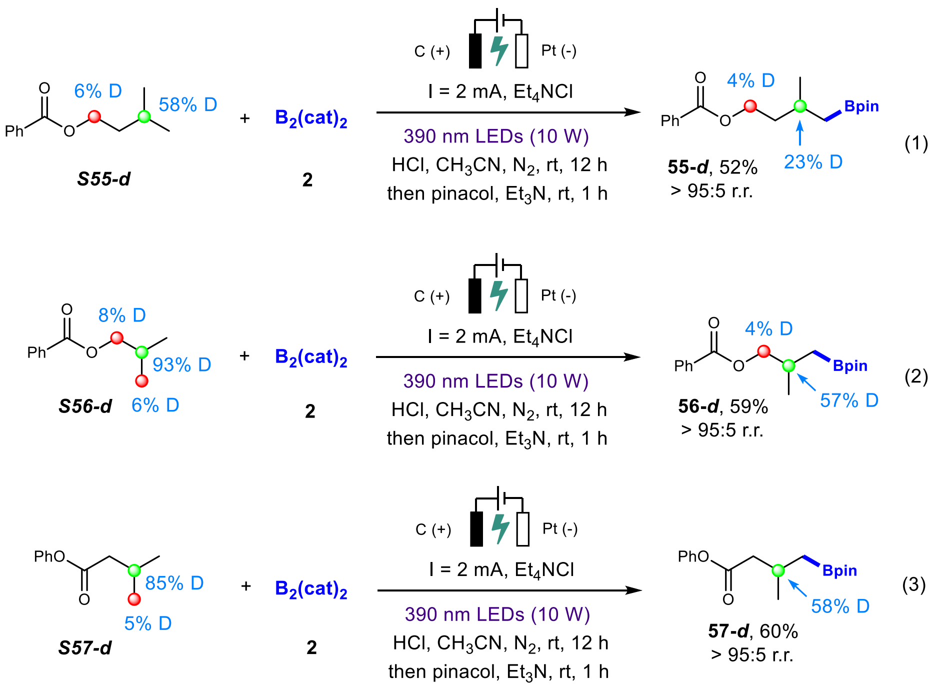

Supplementary Figure 18. H/D scrambling experiment

^{1}H NMR spectrum (400 MHz, $CDCl_3$) of **S55-d**

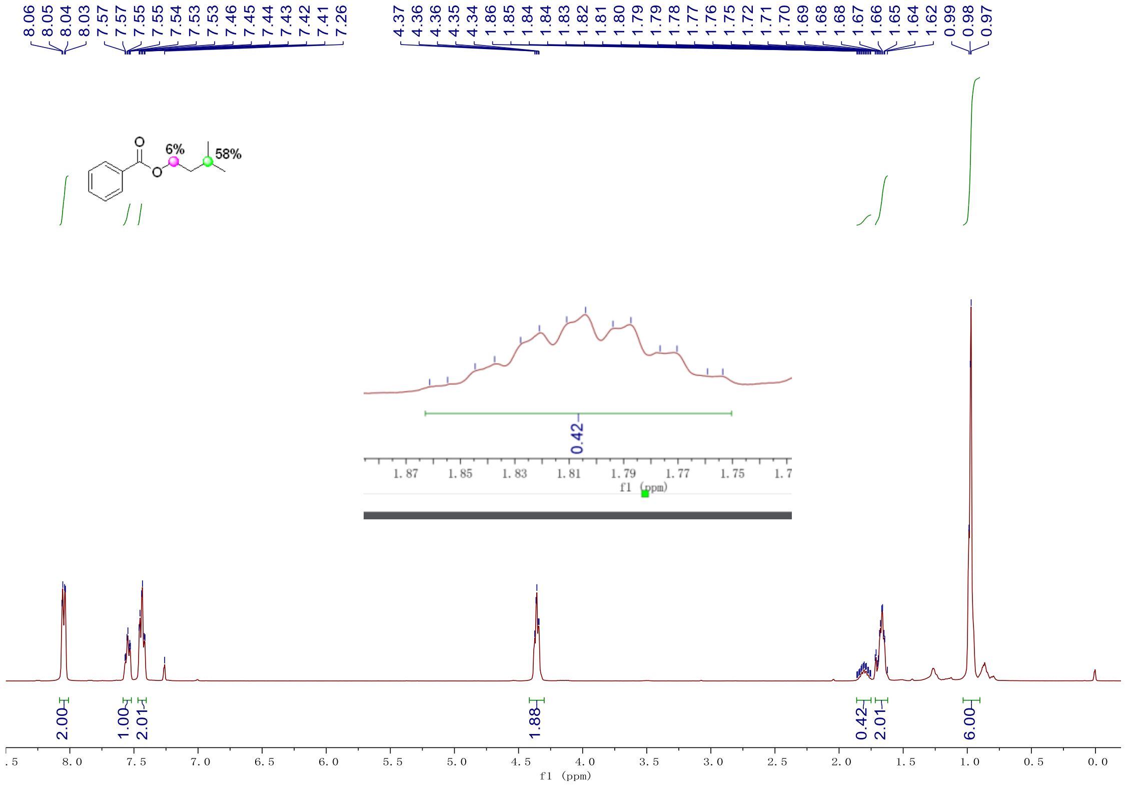

^{1}H NMR spectrum (400 MHz, $CDCl_3$) of **55-d**

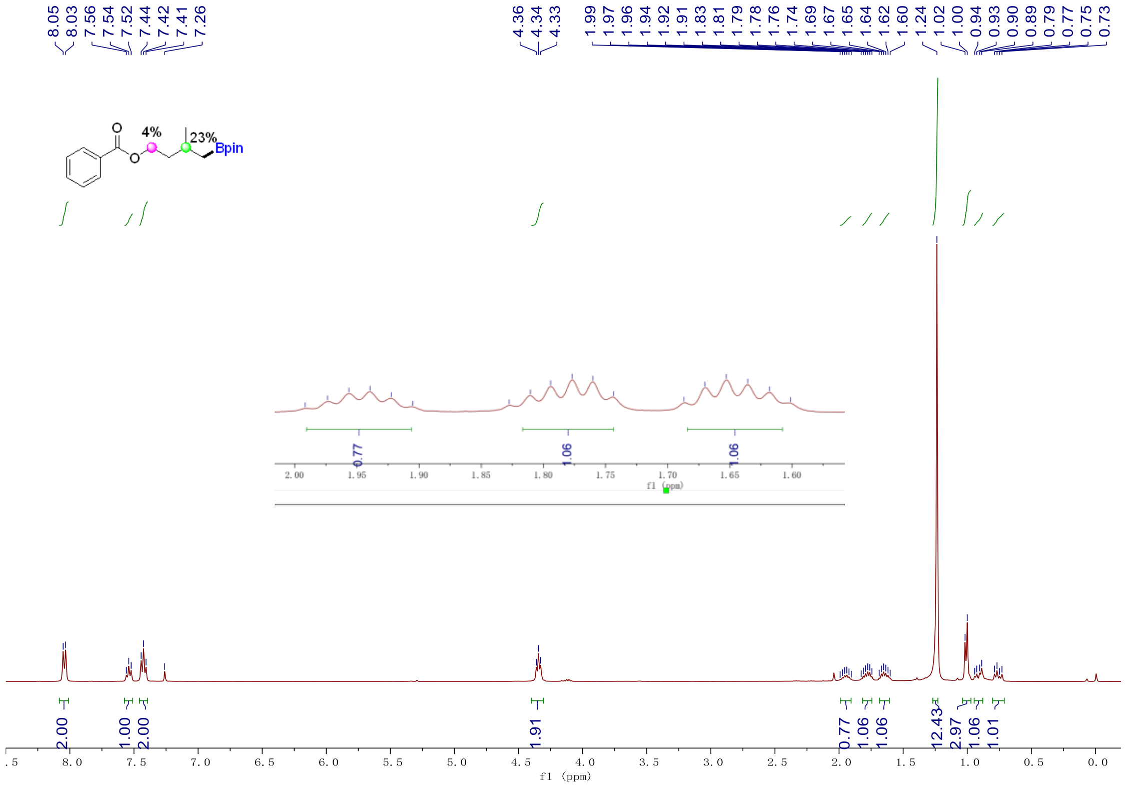

^{1}H NMR spectrum (400 MHz, $CDCl_3$) of **S56-d**

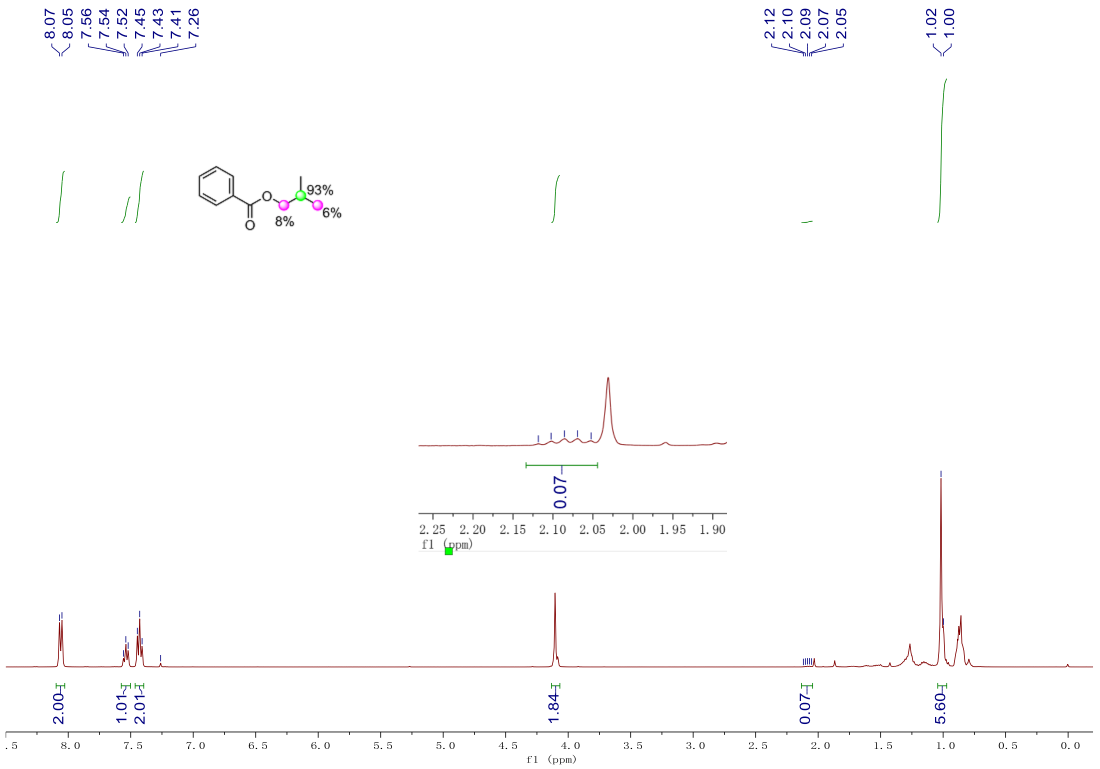

^{1}H NMR spectrum (400 MHz, $CDCl_3$) of **56-d**

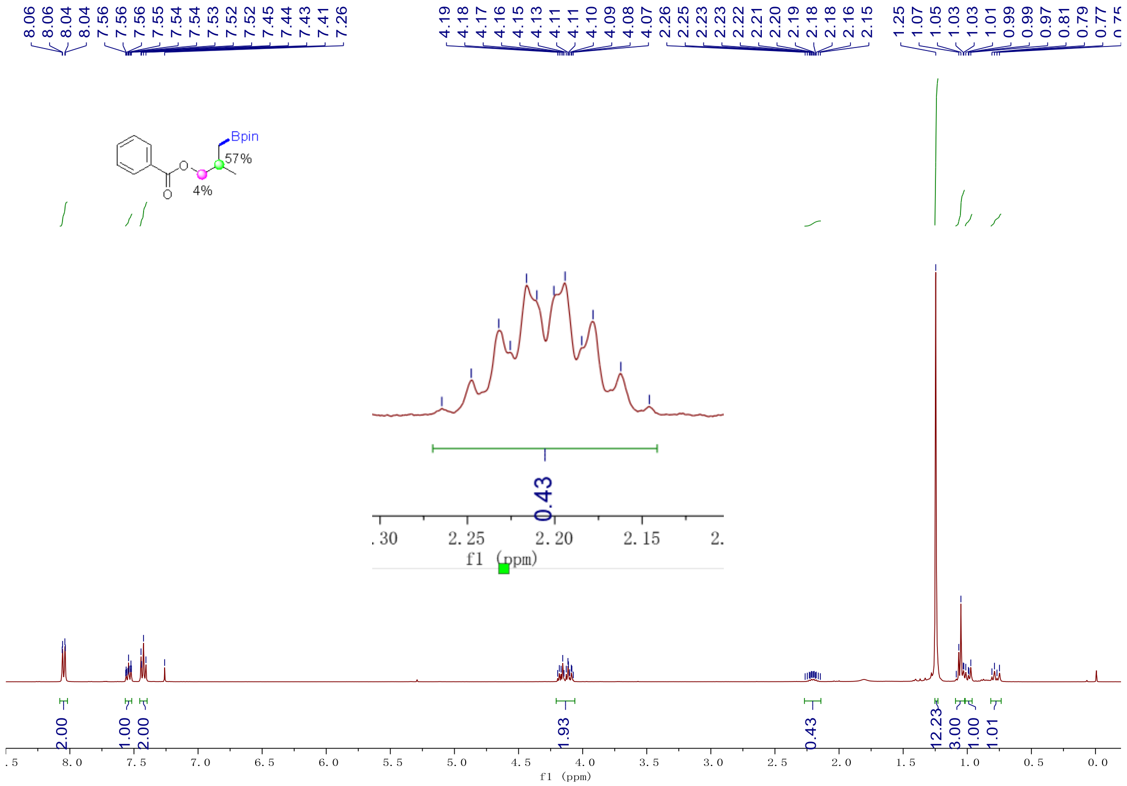

^{1}H NMR spectrum (400 MHz, $CDCl_3$) of **S57-d**

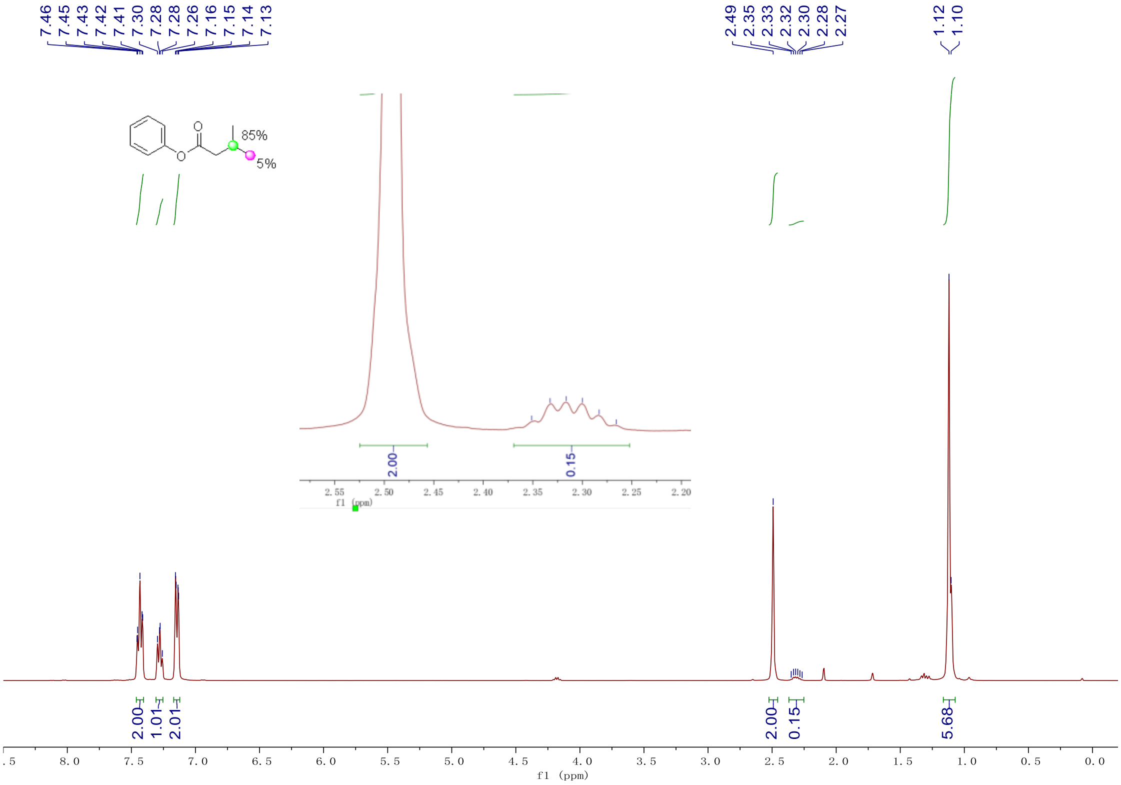

^{1}H NMR spectrum (400 MHz, $CDCl_3$) of **57-d**

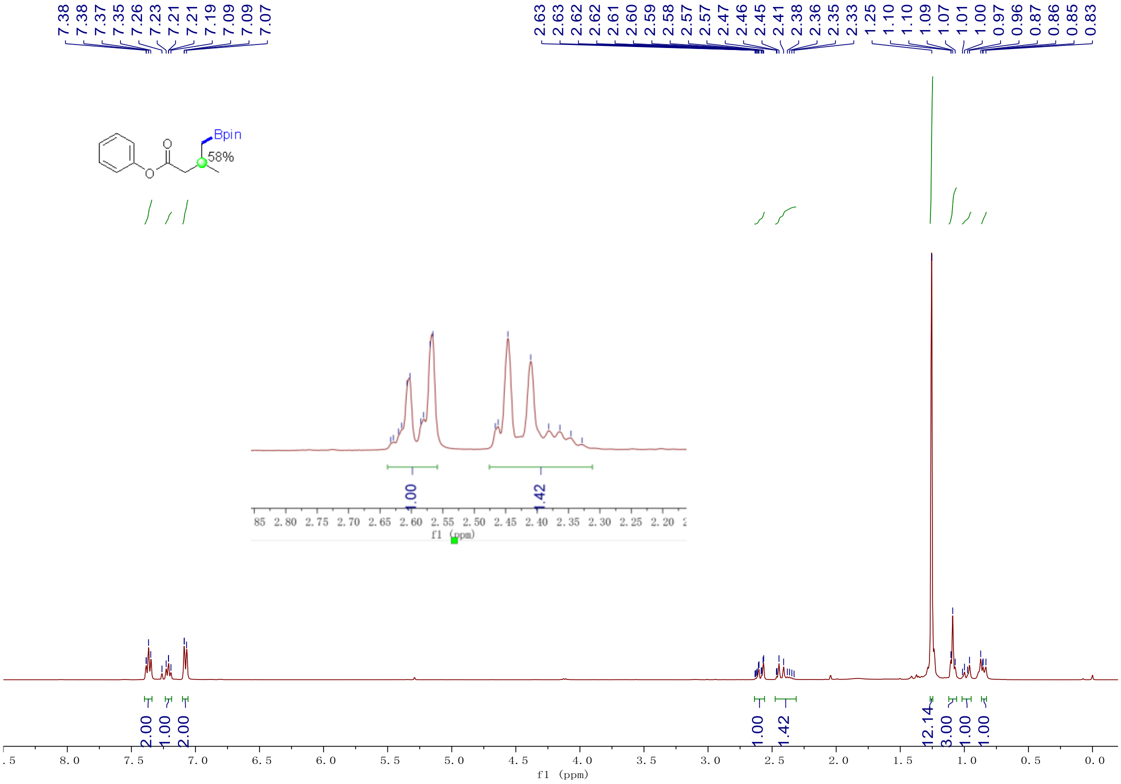

(2) Kinetic isotopic effect (KIE) experiment

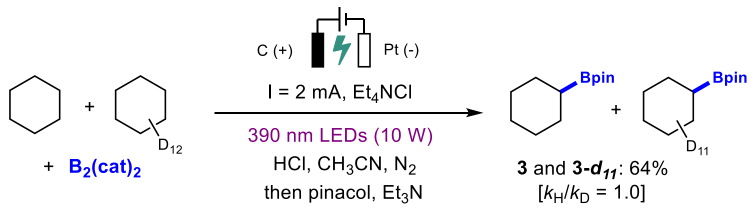

Supplementary Figure 19. Kinetic isotopic effect (KIE) experiment

To a 10 mL vial equipped with a stir bar was added $B_2(cat)_2$ (48 mg, 0.20 mmol, 1.0 equiv.), Et_4NCl (0.1 mmol, 0.5 equiv.), and HCl (concentrated, 0.8 mmol, 4.0 equiv.). MeCN (6 mL) was then added followed by the cyclohexane and cyclohexane-d{12} (n:n=1:1, 2.0 mmol, 10 equiv.). The reaction mixture was stirred at 680 rpm, irradiated with 390 nm LED lamps and 2.0 mA for 12 h. The reaction temperature was maintained at approximately room temperature by cooling with a desk fan. After irradiation, a solution of pinacol (71 mg, 0.60 mmol, 3.0 equiv.) and Et_3N (0.84 mL, 6.0 mmol, 30 equiv.) in CH_2Cl_2 (1 mL) was added and stirring was continued for 1 h. The reaction mixture was concentrated in vacuo and purified by flash column chromatography. The target compound was obtained with a yield of 64%. Comparing the ^1H NMR spectra, we found the ratio of **3**: **3-*d*$_{11}$** was 1:1, so the intermolecular KIE value was 1.0.

2-Cyclohexyl-4,4,5,5-tetramethyl-1,3,2-dioxaborolane (3) and **2-(cyclohexyl-d$_{11}$)-4,4,5,5-tetramethyl-1,3,2-dioxaborolane (3-*d*$_{11}$)**

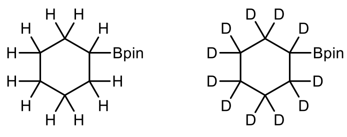

3 **3-*d*$_{11}$**

^1H NMR (400 MHz, Chloroform-*d*) δ 1.73 – 1.47 (m, 4H), 1.32 – 1.24 (m, 4H), 1.22 (s, 12H), 1.02 – 0.85 (m, 0.8H). ^{13}C NMR (101 MHz, Chloroform-*d*) δ 82.8, 28.1, 27.3, 26.9, 24.9.

^{1}H NMR (400 MHz, $CDCl_3$) of **3** and **3-*d*₁₁**

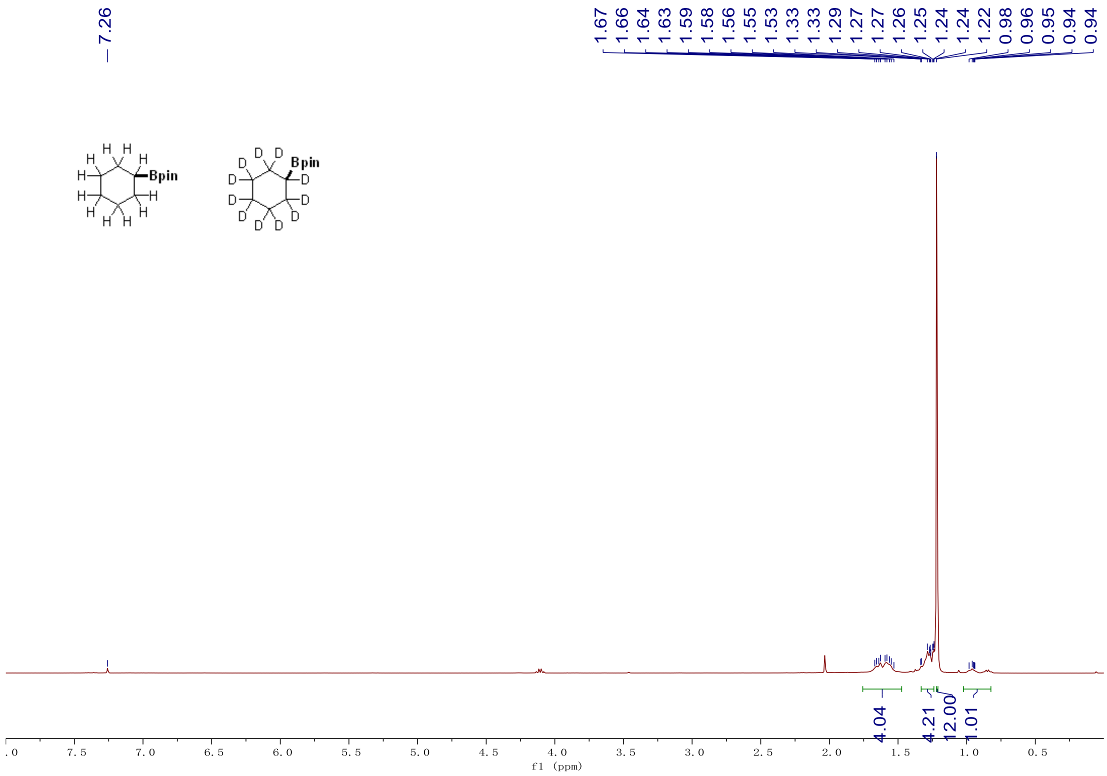

^{13}C NMR (101 MHz, $CDCl_3$) of **3** and **3-*d*₁₁**

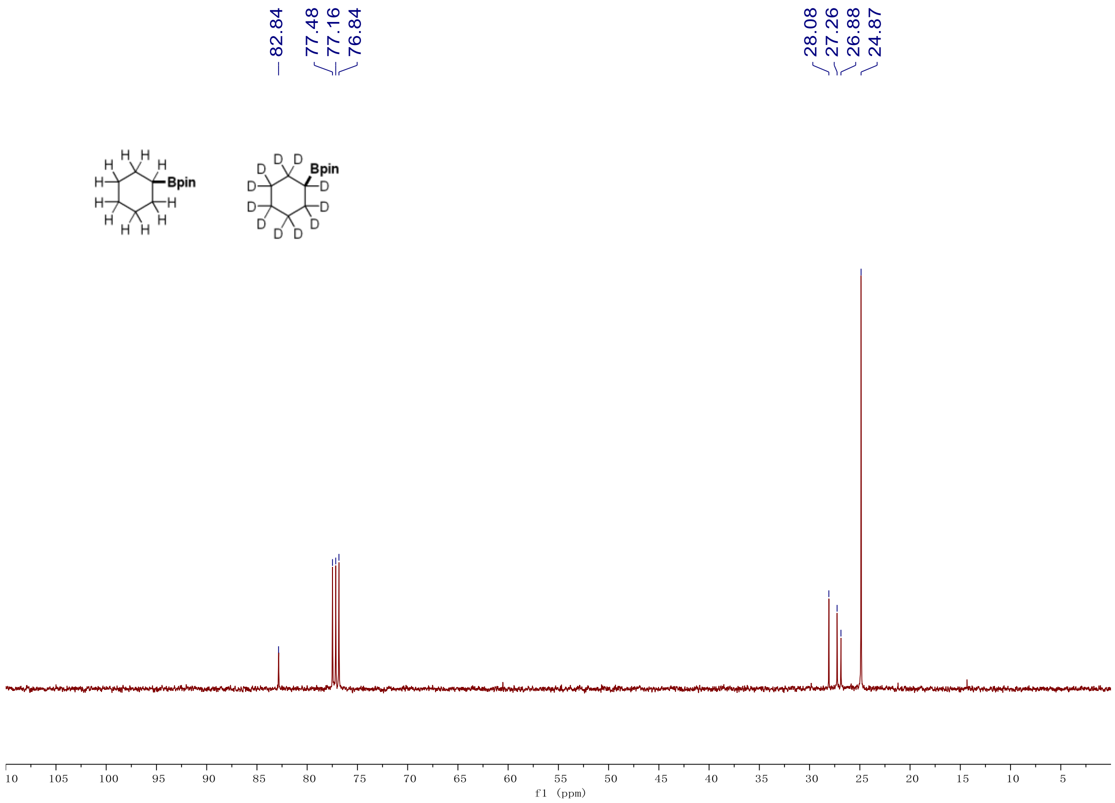

2.6. Chlorine radical-trapping experiment

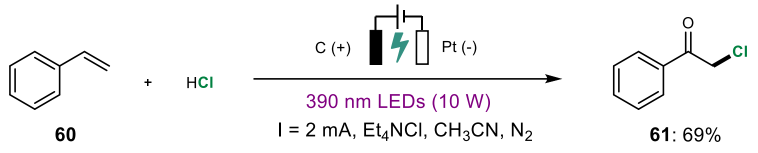

Supplementary Figure 20. Chlorine radical-trapping reaction of styrene **60**

To a 10 mL vial equipped with a C anode (φ = 3 mm), a platinum plate cathode (10 x 10 x 0.1 mm), and a magnetic stir bar was added styrene (**60**, 0.3 mmol, 1 equiv). After three cycles of evacuation and backfilling of the reaction flask with argon, a solution of Et_4NCl (0.15 mmol, 0.5 equiv) in CH_3CN (6 mL) and HCl (concentrated, 1.2 mmol, 4 equiv) were added. The electrolysis was carried out in dark using a constant current of 2 mA at room temperature until complete consumption of styrene. The reaction mixture was concentrated in vacuo and purified by flash column chromatography. The target compound was obtained with a yield of 69%. All recorded spectroscopic data matched those previously reported in the literatures.[4-6]

2-Chloro-1-phenylethan-1-one (61)

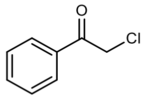

^{1}H NMR (400 MHz, Chloroform-*d*) δ 7.95 (d, *J* = 7.9 Hz, 2H), 7.61 (t, *J* = 7.4 Hz, 1H), 7.49 (t, *J* = 6.9 Hz, 2H), 4.72 (s, 2H).

^{13}C NMR (101 MHz, Chloroform-*d*) δ 191.2, 134.3, 134.1, 129.0, 128.6, 46.2.

This compound has been previously reported; the spectroscopic data are identical to those in reference (*Adv. Synth. Catal.* **2013**, *355*, 1077.).

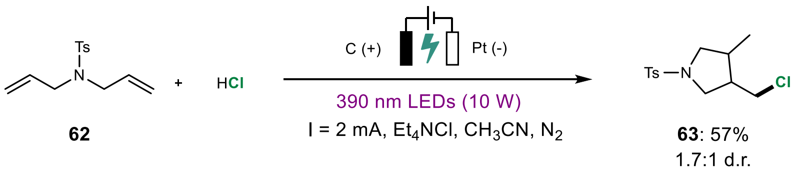

Supplementary Figure 21. Chlorine radical-trapping reaction of diene **62** with HCl

To a 10 mL vial equipped with a C anode (φ = 3 mm), a platinum plate cathode (10 x 10 x 0.1 mm), and a magnetic stir bar was added *N*,*N*-diallyltosylamine (**62**, 0.3 mmol, 1 equiv). After three cycles of evacuation and backfilling of the reaction flask with argon, a solution of Et_4NCl (0.15 mmol, 0.5 equiv) in CH_3CN (6 mL) and HCl (concentrated, 1.2 mmol, 4 equiv) were added. The electrolysis was carried out in dark using a constant current of 2 mA at room temperature until complete consumption of substrate **62**. The reaction mixture was concentrated in vacuo and purified by flash column chromatography. The target compound was obtained with a yield of 57%.

3-(chloromethyl)-4-methyl-1-tosylpyrrolidine (63)

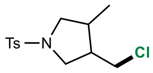

^{1}H NMR (400 MHz, Chloroform-*d*) δ 7.69 (dt, *J* = 8.1, 2.2 Hz, 2H), 7.32 (d, *J* = 7.9 Hz, 2H), 3.55 – 3.34 (m, 3H), 3.35 – 3.25 (m, 0.42 H), 3.23 – 3.09 (m, 1.58H), 2.99 (ddd, *J* = 9.8, 4.9, 1.6 Hz, 0.66 H), 2.83 – 2.75 (m, 0.39 H), 2.47 – 2.25 (m, 4.52 H), 1.97 (dp, *J* = 20.2, 7.4, 6.2 Hz, 0.76 H), 0.94 (dd, *J* = 6.3, 1.7 Hz, 1H), 0.81 (dd, *J* = 6.9, 1.6 Hz, 2H).

^{13}C NMR (101 MHz, Chloroform-*d*) δ 143.7, 143.6, 133.6, 133.3, 129.8, 127.6, 127.5, 54.8, 54.4, 51.4, 50.1, 47.7, 44.9, 44.4, 42.8, 36.4, 34.9, 21.6, 16.6, 12.8.

HRMS (ESI$^+$) calcd. for $C_{13}H_{19}ClNO_2S^+$ $[M+H]^+$,M/Z: 288.0820, found 288.0817.

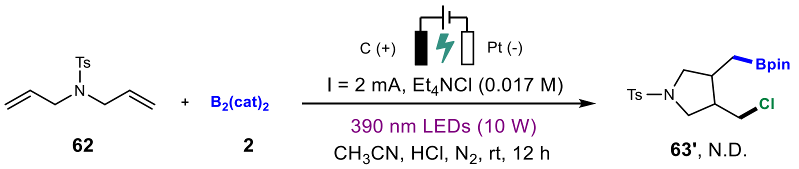

Supplementary Figure 22. Chlorine radical-trapping reaction of diene **62** with B_2cat_2

In the presence of $B_2(cat)_2$, the formation of borylated compound **63’** via chlorine radical addition–5-*exo*-trig cyclization–borylation sequence was not detected.

2.7. Divided-cell and control experiment

(1) Procedure for divided-cell reaction

Supplementary Table 4: Divided-cell experiment

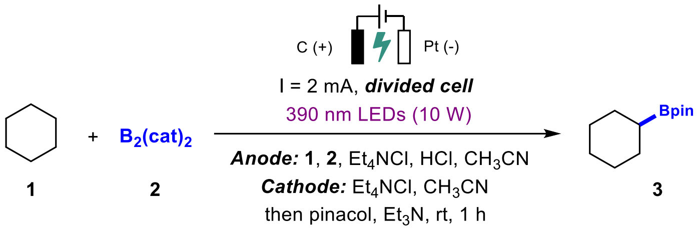

Entry	Deviation from standard conditions	Yield (%)
1	None	46
2	Without HCl at anode	41

A divided-cell experiment was carried out. Substrates **1** and **2**, along with Et_4NCl, HCl, and CH_3CN, was placed into the anode chamber, while Et_4NCl and CH_3CN were added into the cathode chamber. As expected, upon light irradiation and electrolysis for 12 h the desired product **3** was detected in the anode chamber with 46% yield, suggesting that the photoelectrochemical C(sp^3)–H borylation occurred surrounding the graphite rod anode. Interestingly, a moderate yield (41%) of alkyl boronate **3** was still afforded in the absence of HCl at the anode chamber, probably because the $B_2(cat)_2$ reagent in the anode chamber of divided-cell would not be electrochemically reduced, and therefore it does not need the protection of HCl anymore. The protons that come from trace amount of H_2O in the cathode cell are combined with electrons at the cathode to generate H_2.

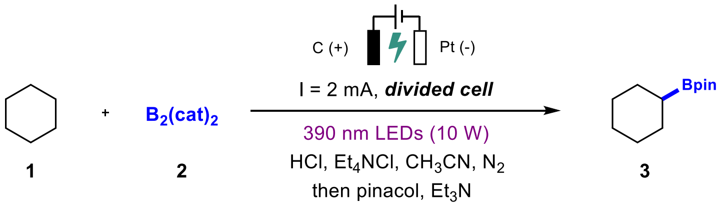

Supplementary Figure 23. Divided-cell experiment

Procedure: To a 10 mL divided-cell equipped with a stir bar, In anode reaction cell was added $B_2(cat)_2$ (48 mg, 0.20 mmol, 1.0 equiv.), Et_4NCl (0.1 mmol, 0.5 equiv.), and HCl

or not (concentrated, 0.8 mmol, 4.0 equiv.). MeCN (6 mL) was then added followed by the alkane (2.0 mmol, 10 equiv.). In cathodic reaction cell was added Et_4NCl (0.1 mmol, 0.5 equiv.) and MeCN (6 mL). The reaction mixture was stirred at 680 rpm, irradiated with 390 nm LED lamps and 2.0 mA for 12 h. The reaction temperature was maintained at approximately room temperature by cooling with a desk fan. After irradiation, a solution of pinacol (71 mg, 0.60 mmol, 3.0 equiv.) and Et_3N (0.84 mL, 6.0 mmol, 20 equiv.) in CH_2Cl_2 (1 mL) was added and stirring was continued for 1 h. The reaction mixture was concentrated in vacuo and then purified by flash column chromatography (5% EtOAc/henane) to give **3** as a colourless oil.

(2) Procedure for undivided-cell reaction

Supplementary Table 5: Control experiment for undivided-cell reaction

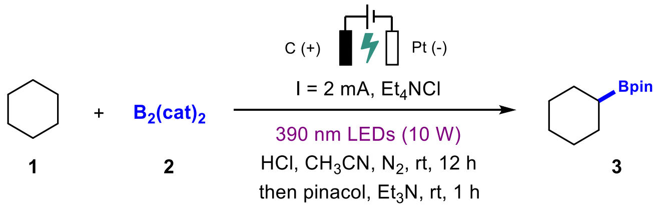

Entry	Deviation from standard conditions	Yield (%)
1	None	72
2	NaCl / CsCl / LiCl instead of HCl	< 5
3	H_3PO_4 instead of HCl	67

In order to further understand the role of HCl in this photoelectrochemical system, several control experiments were carried out in an undivided-cell system. Replacing HCl with other inorganic chloride salts, such as NaCl, CsCl, and LiCl, was found to be ineffective. Although the use of other acids instead of HCl, such as TFA and TfOH, led to complete loss of reaction efficiency (Table 1, entry 15), substituting HCl by H_3PO_4 successfully gave the desired C–H borylated product **3** in 67% yield, which indicates that HCl mainly plays a crucial role as proton source rather than chlorine source. At the cathode surface, protons undergo cathodic reduction to generate H_2, and protect the substrate $B_2(cat)_2$ from single-electron reduction in certain conditions (*J. Am. Chem. Soc.* **2021**, *143*, 12985.).

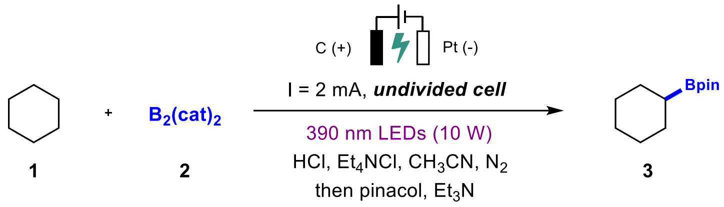

Supplementary Figure 24. Undivided-cell experiment

To a 10 mL undivided-cell equipped with a stir bar, In anode reaction cell was added $B_2(cat)_2$ (48 mg, 0.20 mmol, 1.0 equiv.), Et_4NCl (0.1 mmol, 0.5 equiv.), and Acid or chlorine source (concentrated, 0.4-0.8 mmol, 2.0-4.0 equiv.). MeCN (6 mL) was then added followed by the alkane (2.0 mmol, 10 equiv.). In cathodic reaction cell was added Et_4NCl (0.1 mmol, 0.5 equiv.) and MeCN (6 mL). The reaction mixture was stirred at 680 rpm, irradiated with 390 nm LED lamps and 2.0 mA for 12 h. The reaction temperature was maintained at approximately room temperature by cooling with a desk fan. After irradiation, a solution of pinacol (71 mg, 0.60 mmol, 3.0 equiv.) and Et_3N (0.84 mL, 6.0 mmol, 20 equiv.) in CH_2Cl_2 (1 mL) was added and stirring was continued for 1 h. The reaction mixture was concentrated in vacuo and then purified by flash column chromatography (5% EtOAc/henane) to give **3** as a colourless oil.

(3) Divided-cell experiment with ClB(cat)

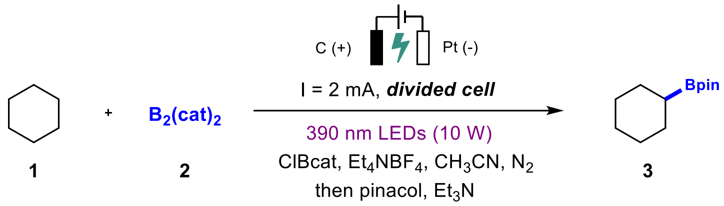

Supplementary Figure 25. Divided-cell experiment with ClB(cat)

To demonstrate the importance of ClB(cat) in the reaction, divided-cell experiment was performed under standard conditions. Use Et_4NBF_4 (0.5 eq.) as the electrolyte and ClBcat (0.5 eq.) as the chlorine source for the reaction. Two hours of reaction sampling followed by post-processing for GC analysis, and eight hours of reaction sampling for GC analysis. The yield of the target compounds obtained was found to be 8%.

2.8. Regioselectivity studies on the reaction of 2,3-dimethylbutane (DMB)

Since the chlorine radical-mediated HAT step has proved to be not turnover-limiting through our KIE experiment, we hypothesized that the afforded regioselectivity is highly dependent on the choice of radical acceptors. To testify our hypothesis, we chose 2,3-dimethylbutane (DMB), a branched isomer of hexane that was recently utilized to probe the HAT species by Zuo (*J. Am. Chem. Soc.* **2020**, *142*, 6216; *J. Am. Chem. Soc.* **2023**, *145*, 359.) and Walsh/Schelter (*Science* **2021**, *372*, 847.), as a standard hydrocarbon substrate for the evaluation of site selectivity. The two methine positions of DMB contain weaker 3° C–H bonds in comparison to the four distal methyl groups. Several radical acceptors have been tested under our photoelectrochemical conditions as well as other chlorine radical-mediated photochemical systems (ref. a-c). All these results indicate that the chlorine radical-mediated HAT process preferentially occur at the weaker 3° C–H bonds according to its intrinsic selectivity. However, the site selectivity is also largely affected by the use of radical acceptors.

Supplementary Table 6: Discussion of regioselectivity for various radical acceptors

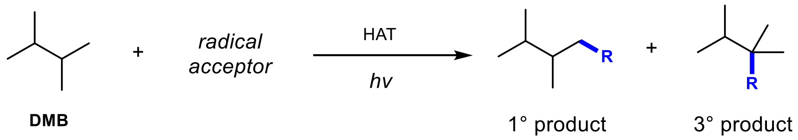

HAT condition	radical acceptor	intrinsic selectivity (1°:3°)	yield (%)
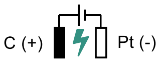 390 nm LEDs (10 W), I = 2 mA, Et_4NCl, CH_3CN, N_2	$B_2(cat)_2$	> 20:1	72
	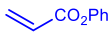	1:6	74
	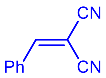	1:3	70
$FeCl_3 \cdot 6H_2O$[a]	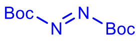	1:5	84
Mes-Acr$^+$/$n$$Bu_4NCl$[b]	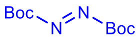	1:6	26
$FeCl_3 \cdot 6H_2O$[c]	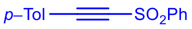	> 99:1	62

a) Duan, C. et al. *Green Chem.* **2021**, *23*, 6984-6989.
b) Zuo, Z. et al. *J. Am. Chem. Soc.* **2023**, *145*, 359-376.
c) Duan, C. et al. *Green Chem.* **2021**, *23*, 9406-9411.

Procedure: To a 10 mL vial equipped with a stir bar was add free radical acceptor (0.20 mmol, 1.0 equivalent), Et_4NCl (0.1 mmol, 0.5 equivalent), and HCl (concentrated, 0.8 mmol, 4.0 equivalent) to a 10 mL vial containing a stirring rod. Then add MeCN (6 mL), and then add 2,3-dimethylbutane (2.0 mmol, 10 equivalents). The reaction mixture was stirred at 680 rpm, illuminated with a 390 nm LED lamp, and electrolyzed at 2.0 mA for 12 hours. Maintain the reaction temperature at approximately room temperature by cooling with a desktop fan. After irradiation, the reaction mixture was vacuum concentrated and purified through rapid column chromatography to obtain the corresponding transparent oil like product. The intrinsic selectivity of 1°/3° C(sp^3)–H bonds was determined by ^{1}H NMR analysis.

Under the standard photoelectrochemical conditions of our C(sp^3)–H borylation reaction, control reaction revealed that the addition of benzylidenemalononitrile (**64**) as a typical radical acceptor greatly inhibited the desired C–H borylation reaction, while generating C–H alkylated product **65** preferentially at the weaker 3° C–H bond (intrinsic selectivity of 1°/3°: 1:3). Consistent with this finding, the addition of phenyl acrylate (**66**) into the photoelectrochemical system with DMB (**S9**) and $B_2(cat)_2$ (**2**) produced C–H alkylated product **67** in 65% yield, and again, the alkylation at the 3° C–H bond is preferred (intrinsic selectivity of 1°/3°: 1:6). These results supported the existence of tertiary carbon radicals as well as the reversible HAT process, which is consistent with the H/D scrambling results.

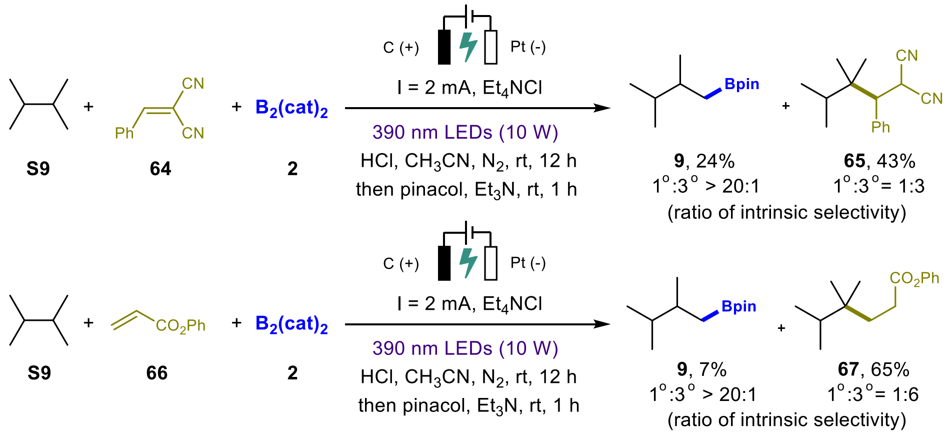

Supplementary Figure 26. Trapping by other radical acceptors

Procedure: To a 10 mL vial equipped with a stir bar was add free radical acceptor (0.20 mmol, 1.0 equivalent), $B_2(cat)_2$ (48 mg, 0.20 mmol, 1.0 equiv.), Et_4NCl (0.1 mmol, 0.5 equivalent), and HCl (concentrated, 0.8 mmol, 4.0 equivalent) to a 10 mL vial containing a stirring rod. Then add MeCN (6 mL), and then add 2,3-dimethylbutane (2.0 mmol, 10 equivalents). The reaction mixture was stirred at 680 rpm, illuminated with a 390 nm LED lamp, and electrolyzed at 2.0 mA for 12 hours. Maintain the reaction temperature at approximately room temperature by cooling with a desktop fan. After irradiation, a solution of pinacol (71 mg, 0.60 mmol, 3.0 equiv.) and Et_3N (0.84 mL, 6.0 mmol, 20 equiv.) in CH_2Cl_2 (1 mL) was added and stirring was continued for 1 h. The intrinsic selectivity of 1°/3° C(sp^3)−H bonds was determined by ^1H NMR analysis.

(1) Selective study on the reaction of DMB with 2-benzylidenemalononitrile (**64**)

Alkyl boronate ester **9** was formed in 24% yield with complete terminal 1° C−H selectivity. The alkylated product **65** was formed in 43% yield and 2:1 ratio of 1° and 3° alkylated products (intrinsic selectivity of 1°/3°: 1:3).

2-(2,2,3-trimethyl-1-phenylbutyl)malononitrile (65)

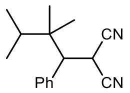

^1H NMR (400 MHz, Chloroform-*d*) δ 7.45 – 7.31 (m, 15H), 4.22 (d, *J* = 5.1 Hz, 1H), 3.89 (d, *J* = 6.0 Hz, 1H), 3.86 (d, *J* = 6.3 Hz, 1H), 3.35 – 3.24 (m, 3H), 2.16 – 1.98 (m, 2H), 1.81 (ddd, *J* = 14.0, 9.6, 6.3 Hz, 1H), 1.73 – 1.58 (m, 4H), 1.52 (td, *J* = 6.8, 4.5 Hz, 1H), 1.36 – 1.26 (m, 2H), 1.12 (s, 3H), 0.95 – 0.75 (m, 30H).

^{13}C NMR (101 MHz, Chloroform-*d*) δ 137.4, 136.5, 136.1, 129.9, 129.3, 129.3, 128.9, 128.9, 128.8, 128.6, 128.0, 127.9, 113.6, 113.3, 112.2, 112.1, 112.0, 53.3, 44.7, 44.5, 39.9, 36.9, 35.8, 35.5, 35.3, 34.1, 32.9, 31.0, 30.2, 30.1, 25.0, 21.6, 20.9, 20.5, 19.4, 18.6, 17.5, 17.3, 16.4, 15.1, 15.1.

This compound has been previously reported; the spectroscopic data are identical to those in reference (*ACS Catal.* **2022**, *120*, 10441.).

(2) Selective study on the reaction of DMB with phenyl acrylate (**66**)

Alkyl boronate ester **9** was formed in 7% yield with complete terminal 1° C−H selectivity. The alkylated product **67** was formed in 65% yield and 1:1 ratio of 1° and 3° alkylated products (intrinsic selectivity of 1°/3°: 1:6).

phenyl 4,4,5-trimethylhexanoate (67)

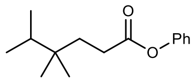

^{1}H NMR (400 MHz, Chloroform-*d*) δ 7.38 (t, *J* = 7.8 Hz, 2H), 7.23 (t, *J* = 7.6 Hz, 1H), 7.11 – 7.05 (m, 2H), 2.59 – 2.47 (m, 2H), 1.88 – 1.30 (m, 5H), 1.29 – 1.15 (m, 1H), 0.93 – 0.80 (m, 11H).

^{13}C NMR (101 MHz, Chloroform-*d*) δ 173.2, 172.5, 150.9, 129.5, 125.8, 121.7, 121.7, 38. 5, 35.5, 35.0, 34.9, 34.8, 33.7, 32.0, 29.7, 23.9, 23.1, 20.3, 18.1, 17.5, 15.4.

This compound has been previously reported; the spectroscopic data are identical to those in reference (*Nature* **2016**, *539*, 268.).

2.9. C(sp^3)–H borylation in the presence of Cl_2

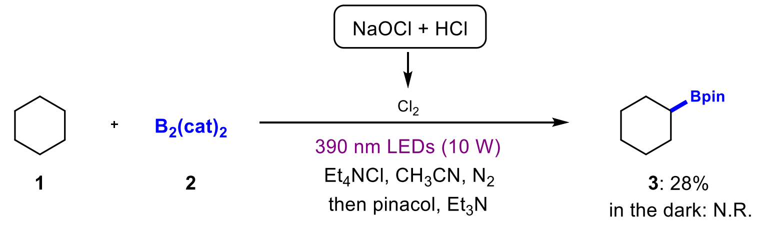

Supplementary Figure 27. C(sp^3)–H borylation in the presence of Cl_2

To a 10 mL vial equipped with a stir bar was added compound **1** (2 mmol, 10 equiv), $B_2(cat)_2$ (48 mg, 0.20 mmol, 1.0 equiv.), Et_4NCl (0.1 mmol, 0.5 equiv.), and HCl (concentrated, 0.8 mmol, 4.0 equiv.), and CH_3CN (6 mL). NaOCl solution (10% available chlorine, 0.5 mL, 6 equiv) was then added dropwise to the mixture. The reaction mixture was stirred at 680 rpm, irradiated with 390 nm LED lamps and 2.0 mA for 12 h. The reaction temperature was maintained at approximately room temperature by cooling with a desk fan. After irradiation, a solution of pinacol (71 mg, 0.60 mmol, 3.0 equiv.) and Et_3N (0.84 mL, 6.0 mmol, 30 equiv.) in CH_2Cl_2 (1 mL) was added and stirring was continued for 1 h. The reaction mixture was concentrated in vacuo and then purified by flash column chromatography (5% EtOAc/henane) to give **3** (12.2 mg, 0.06 mmol, 28%) as a colourless oil. Compound **3** was not detected if the reaction was carried out in dark.

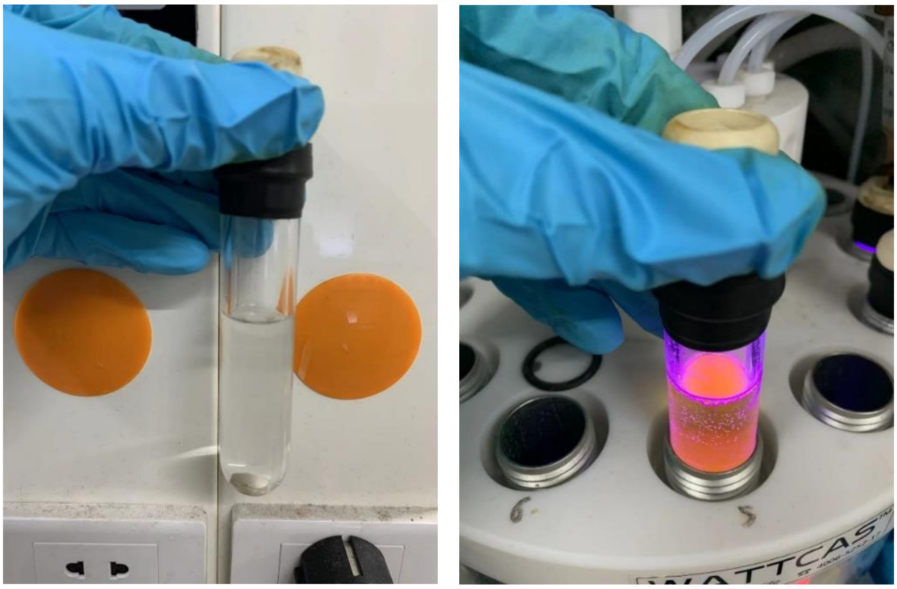

Supplementary Figure 28. Procedure for C(sp^3)–H borylation in the presence of Cl_2

2.10. Cyclic voltammetry experiments

All cyclic voltammetry experiments were performed under a nitrogen atmosphere. The cell setup consisted of a glassy carbon working electrode, Pt coil counter electrode, and Hg/HgCl (in saturated KCl solution) reference electrode. All measurements were performed in dry degassed MeCN, using 0.1 M (n-Bu)$_4$NClO$_4$ as the supporting electrolyte (recrystallised from ethanol). Each measurement consisted of an oxidative scan, followed by the reverse reductive scan, using 100 mV/s scan rate.

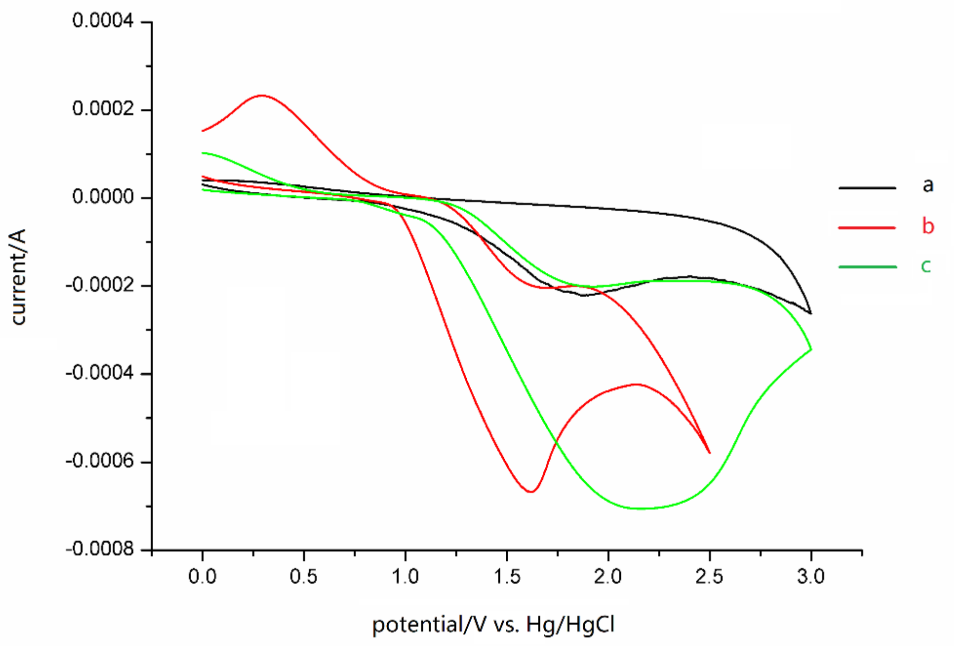

Supplementary Figure 29. Cyclic voltammograms. The cyclic voltammogram was measured in MeCN (nBu$_4$NClO$_4$). **a** HCl (2 mmol·L^{-1}); **b** Et$_4$NCl (2 mmol·L^{-1}); **c** B$_2$(cat)$_2$ **2** (10 mmol·L^{-1}).

2.11. EPR studies

General procedure for EPR studies

To a 10 mL vial equipped with a stir bar was added $B_2(cat)_2$ (48mg, 0.2 mmol, 1.0 equiv.), Et_4NCl (0.1 mmol, 0.5 equiv.), and HCl (concentrated, 0.8 mmol, 4.0 equiv.). MeCN (3 mL) was then added followed by the pentane (2.0 mmol, 10 equiv.). The reaction mixture was stirred at 680 rpm, irradiated with 390 nm LED lamps and 2.0 mA electrolysis. The reaction temperature was maintained at approximately room temperature by cooling with a desk fan. which using a constant current for 0.5 h. Add DMPO (15 mmol) to the reaction bottle and continue stirring for 10min, The solution sample was taken out into a small tube for EPR test. EPR spectra was recorded at room temperature on EPR Elexsys E500 spectrometer operated at 9.855 GHz. Typical spectrometer parameters were shown as follows, sweep width: 200.00 G; center field set: 3500.00 G; conversion time: 58.59 ms; sweep time: 59.99 s; modulation amplitude: 1.0 G; modulation frequency: 100 kHz; PowerAtten10.0 dB; microwave power: 20.00 mW.

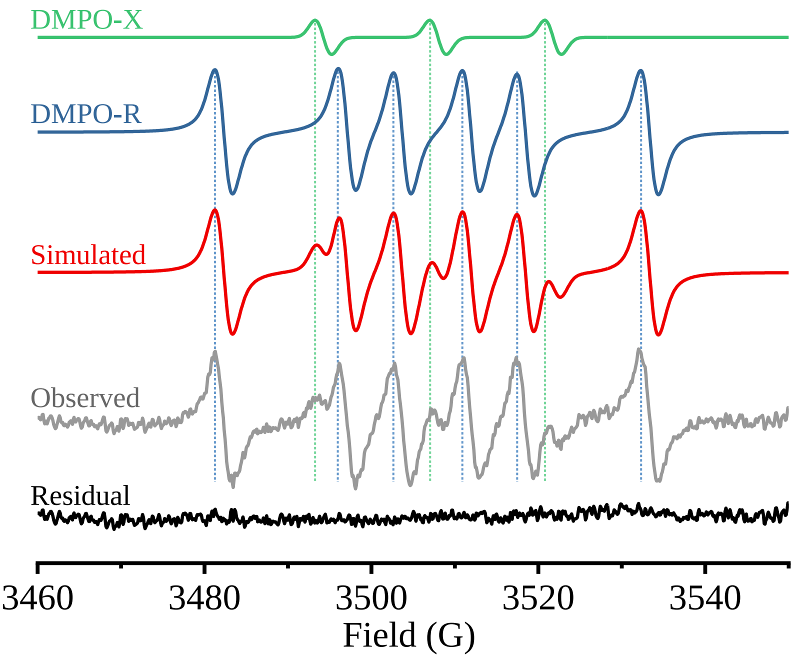

Supplementary Figure 30. Electron paramagnetic resonance (EPR) spectrum

Supplementary Table 7: Electron paramagnetic resonance (EPR) data

Types of free radicals	gFactor	Hyperfine coupling constant			Content (%)
		A_N	$A_{H\beta}$	$A_{H\gamma}$	
DMPO-R	2.007	14.8197	21.3883	-	95.71
DMPO-X		13.7637	-	-	4.29

2.12. Regioselectivity studies with *n*-pentane

(1) Effect of $B_2(cat)_2$ stoichiometry

Procedure: To a 10 mL vial equipped with a stir bar was added $B_2(cat)_2$ (0.05-0.40 mmol, 0.5-2.0 equiv.), Et_4NCl (0.1 mmol, 0.5 equiv.), and HCl (concentrated, 0.8 mmol, 4.0 equiv.). MeCN (6 mL) was then added followed by the addition of *n*-pentane (2.0 mmol, 10 equiv.). The reaction mixture was stirred at 680 rpm, irradiated with 390 nm LED lamps and 2.0 mA electrolysis for 12 h. The reaction temperature was maintained at approximately room temperature by cooling with a desk fan. After irradiation, a solution of pinacol (71 mg, 0.60 mmol, 3.0 equiv.) and Et_3N (0.84 mL, 6.0 mmol, 30 equiv.) in CH_2Cl_2 (1 mL) was added and stirring was continued for 1 h. Add trimethoxybenzene (internal standard) to the reaction mixture. After 1 minute of vigorous oscillation, filter 0.20 mL of the crude reaction mixture through a silica short plug and determine the yield through GC analysis.

Supplementary Table 8: Screening of $B_2(cat)_2$ equivalent

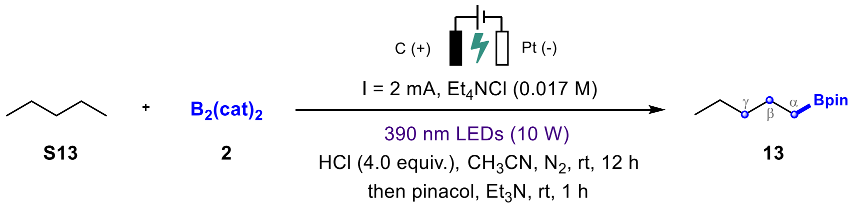

entry	$B_2(cat)_2$ equivalents	product ratio **13α**	**13β**	**13γ**	GC-MS yield (%)	2°/1° selectivity **(13β + 13γ)/13α**
1	0.5	73	22	5	19	0.37
2	1	54	33	13	65	0.85
3	2	37	45	19	74	1.72

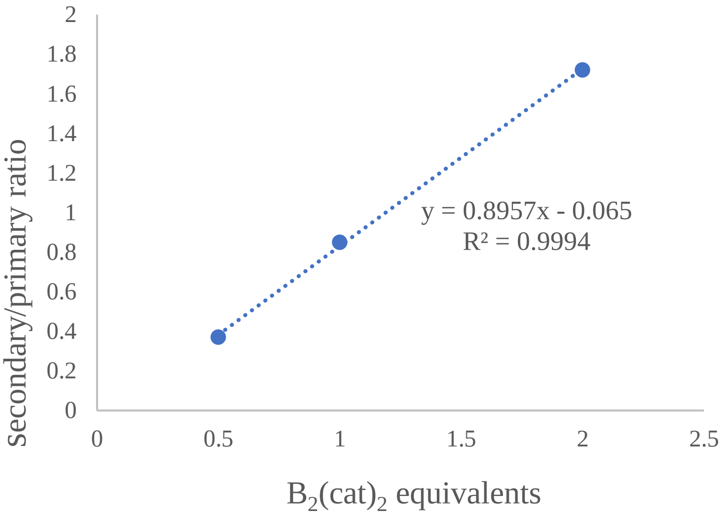

Supplementary Figure 31. Relationship of $B_2(cat)_2$ equivalent with regioselectivity ratio for *n*-pentane

(2) Effect of HCl stoichiometry

Procedure: To a 10 mL vial equipped with a stir bar was added $B_2(cat)_2$ (48mg, 0.2 mmol, 1.0 equiv.), Et_4NCl (0.1 mmol, 0.5 equiv.), and HCl (concentrated, 0.1-1.2 mmol, 0.5-6.0 equiv.). MeCN (6 mL) was then added followed by the addition of *n*-pentane (2.0 mmol, 10 equiv.). The reaction mixture was stirred at 680 rpm, irradiated with 390 nm LED lamps and 2.0 mA electrolysis for 12 h. The reaction temperature was maintained at approximately room temperature by cooling with a desk fan. After irradiation, a solution of pinacol (71 mg, 0.60 mmol, 3.0 equiv.) and Et_3N (0.84 mL, 6.0 mmol, 30 equiv.) in CH_2Cl_2 (1 mL) was added and stirring was continued for 1 h. Add trimethoxybenzene (internal standard) to the reaction mixture. After 1 minute of vigorous oscillation, filter 0.20 mL of the crude reaction mixture through a silica short plug and determine the yield through GC analysis.

Supplementary Table 9: Screening of HCl equivalent

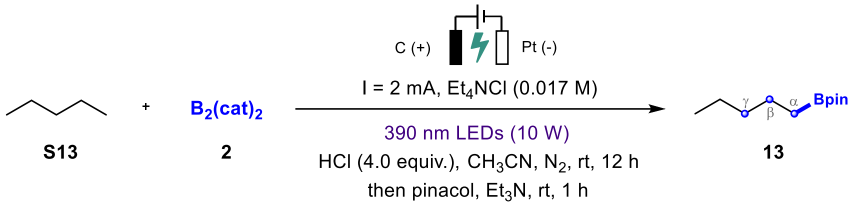

entry	HCl equivalents	product ratio **13α**	**13β**	**13γ**	GC-MS yield (%)	2°/1° selectivity (**13β** + **13γ**)/**13α**
1	0.5	66	23	11		0.52
2	1	60	28	12	33	0.67
3	2	55	32	13	41	0.72
4	4	54	33	13	65	0.85
5	6	48	35	17	58	1.08

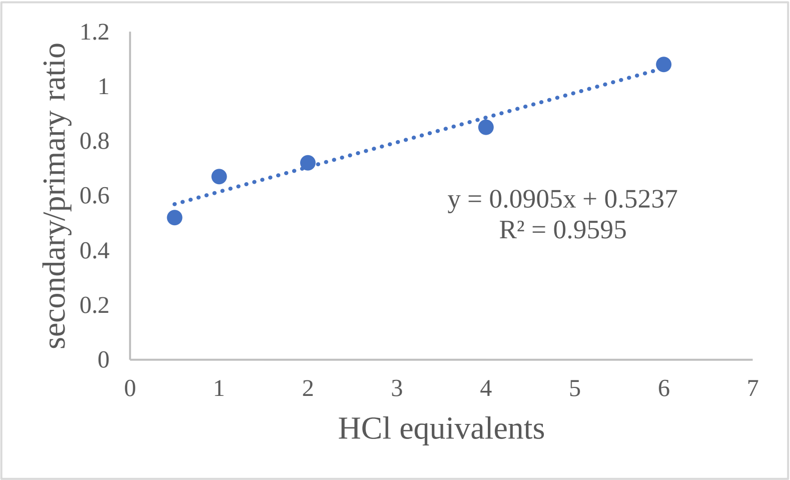

Supplementary Figure 32. Relationship of HCl equivalent with regioselectivity ratio for *n*-pentane

(3) Effect of n-pentane stoichiometry

Procedure: To a 10 mL vial equipped with a stir bar was added $B_2(cat)_2$ (48mg, 0.2 mmol, 1.0 equiv.), Et_4NCl (0.1 mmol, 0.5 equiv.), and HCl (concentrated, 0.8 mmol, 4.0 equiv.). MeCN (6 mL) was then added followed by the addition of *n*-pentane (0.6-10.0 mmol, 3.0-50.0 equiv.). The reaction mixture was stirred at 680 rpm, irradiated with 390 nm LED lamps and 2.0 mA electrolysis for 12 h. The reaction temperature was maintained at approximately room temperature by cooling with a desk fan. After

irradiation, a solution of pinacol (71 mg, 0.60 mmol, 3.0 equiv.) and Et_3N (0.84 mL, 6.0 mmol, 30 equiv.) in CH_2Cl_2 (1 mL) was added and stirring was continued for 1 h. Add trimethoxybenzene (internal standard) to the reaction mixture. After 1 minute of vigorous oscillation, filter 0.20 mL of the crude reaction mixture through a silica short plug and determine the yield through GC analysis.

Supplementary Table 10: Screening of *n*-pentane equivalent

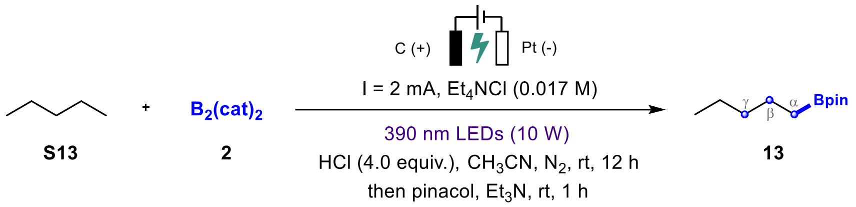

S13 2 13

entry	pentane equivalents	product ratio 13α	13β	13γ	GC-MS yield (%)a	2°/1° selectivity (13β + 13γ)/13α
1	3	61	29	10	21	0.64
2	5	56	32	12	47	0.78
3	10	54	33	13	63	0.85
4	20	46	39	15	65	1.17
5	50	44	40	16	59	1.27

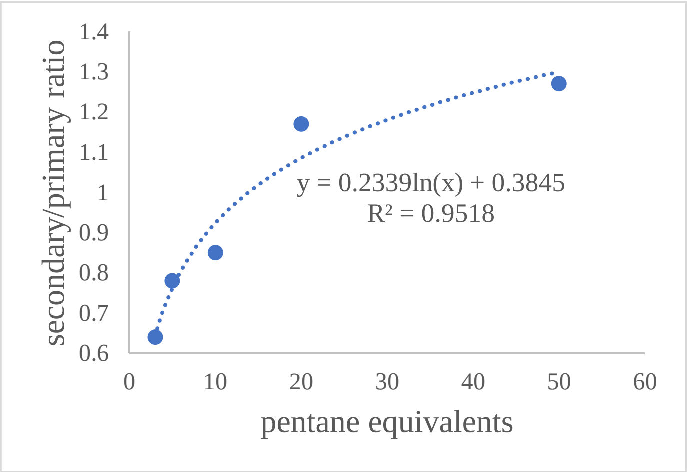

Supplementary Figure 33. Relationship of *n*-pentane equivalent with regioselectivity ratio for *n*-pentane

2.13. Explanation of the different results with *n*Bu_4NCl and Et_4NCl

The use of *n*Bu_4NCl instead of Et_4NCl would promote the hydrolysis of $B_2(cat)_2$ as well as other side reaction process. We have conducted some research to investigate the origin of the drastic difference on yield with the use of *n*Bu_4NCl and Et_4NCl. When using *n*Bu_4NCl to replace Et_4NCl for reaction, no target borylated product was detected. We have found significant differences in the ^1H NMR spectra obtained by studying two different electrolytes. When using Et_4NCl for the photoelectrochemical reaction, the ^1H NMR spectrum was very clean and no obvious by-products were detected. However, when *n*Bu_4NCl was used for the reaction, the ^1H NMR spectrum showed the formation of hydrolysis product (catechol) of B_2cat_2 together with some other unknown by-products. Supplementary Figure 34 shows the ^1H NMR spectra of $B_2(cat)_2$ and catechol in $CDCl_3$.

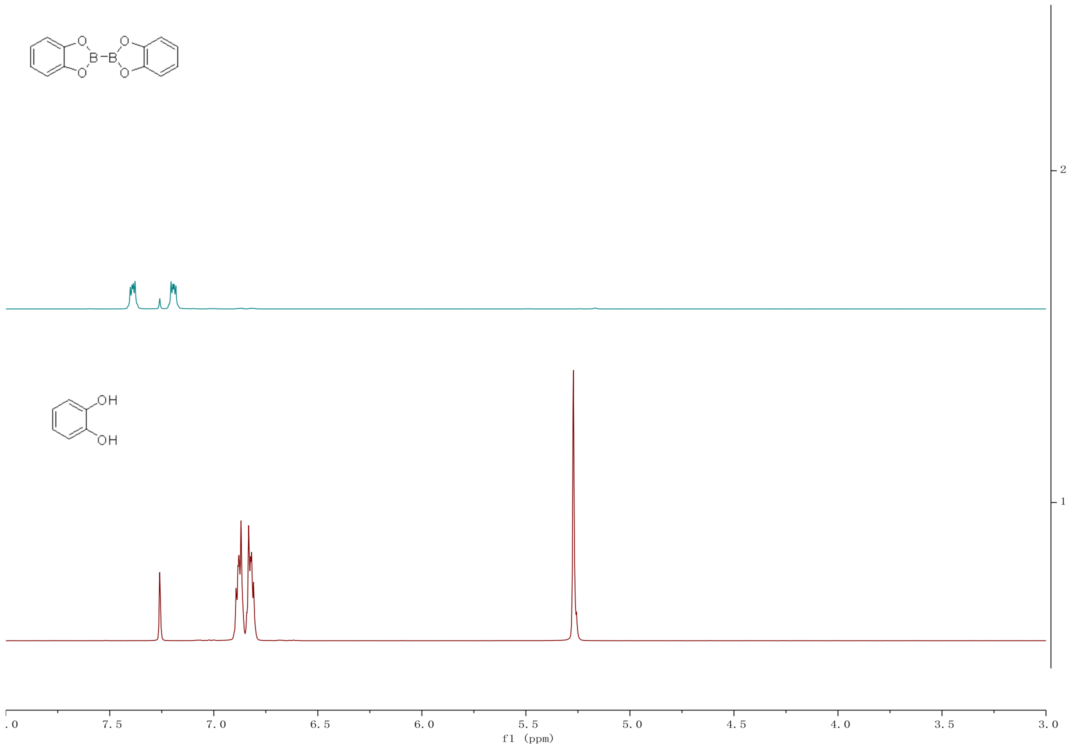

Supplementary Figure 34 ^1H NMR spectra of $B_2(cat)_2$ and catechol in $CDCl_3$

2.14. Time profile of the transformation with the ON-OFF over time

(1) ON-OFF for both light and electricity

The standard reaction is based on the steps of preparing **3** at a scale of 0.2 mmol, and an additional 1.0 equivalent of 1,3,5-$(OMe)_3C_6H_3$ is added as the internal standard. The reaction begins with continuous irradiation and black cycle research on the effects of continuous irradiation of visible light and electricity on the progress of the reaction. After irradiation with 10 W 390nm LED for 2 hours, the reaction mixture was sampled for GC analysis to determine the yield of product **3**. Then, the mixture was stirred for 2 hours with the light and electricity both turned off. After 2 hours of light and electricity on or off, in the same way.

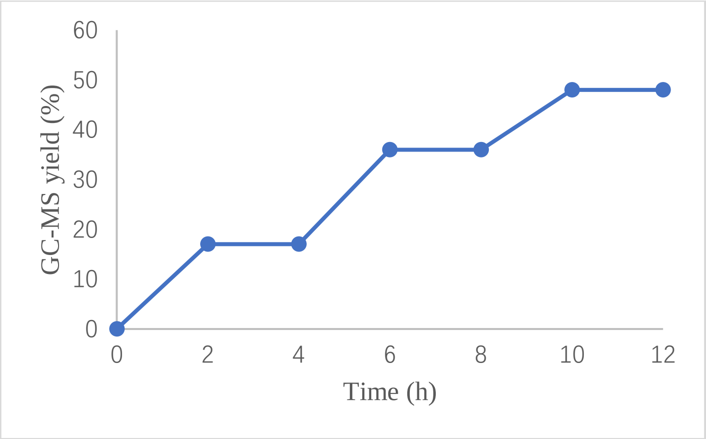

Supplementary Figure 35 ON-OFF results (for both light and electricity)

(2) ON-OFF for electricity

The standard reaction is based on the steps of preparing **3** at a scale of 0.2 mmol, and an additional 1.0 equivalent of 1,3,5-$(OMe)_3C_6H_3$ is added as the internal standard. The reaction begins with continuous photo irradiation and black cycle research on the effects of electrolysis. After irradiation with 10 W 390nm LED for 2 hours, the reaction mixture was sampled for GC analysis to determine the yield of product **3**. Then, stir the mixture for 2 hours without turning off the light but only cutting off the electricity power. In the same way, after 2 hours of power outage or power on. These results indicate that electricity is a necessary component of the reaction.

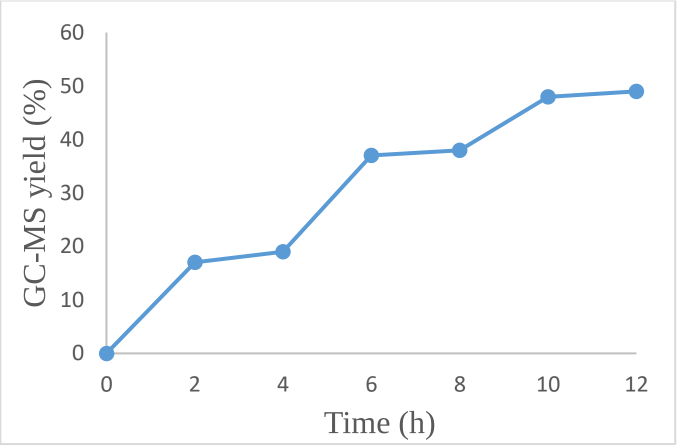

Supplementary Figure 36 ON-OFF results (for electricity)

(3) ON-OFF for photo irradiation

The standard reaction is based on the steps of preparing **3** at a scale of 0.2 mmol, and an additional 1.0 equivalent of 1,3,5-$(OMe)_3C_6H_3$ is added as the internal standard. The reaction begins with continuous electrolysis and black cycle research on the effects of photo irradiation. After irradiation with 10 W 390nm LED for 2 hours, the reaction mixture was sampled for GC analysis to determine the yield of product **3**. Then, stir the mixture for 2 hours with only the light off and power on, without turning off the electricity for electrolysis. In the same way, after turning off or on the lights for 2 hours. These results indicate that light irradiation is a necessary component of the reaction.

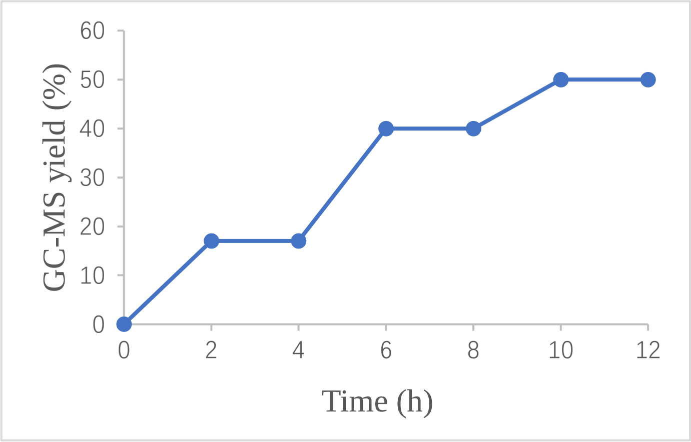

Supplementary Figure 37 ON-OFF results (for photo irradiation)

2.15. Study on the HAT species

(1) Evidence for free Cl radical as HAT species

To probe whether there is a direct HAT process of free chlorine radical in the photoelectrochemical reaction, we used other type of radical receptors instead of B_2cat_2 under the standard conditions. The use of benzylidenemalononitrile (**64**) and phenyl acrylate (**66**) as typical radical acceptors successfully gave the C–H alkylated products **65** and **67** in 70% and 74% yields, respectively. Consistent with other reactions via free Cl radical HAT process, the regioselectivities of these two reactions follow the trend of 3° > 2° > 1°, giving the desired products with 1:3 and 1:6 ratio of intrinsic selectivity (1°/3°), respectively. If B_2cat_2 is also added into the photoelectrochemical system, a Cl-boron complex may be rapidly in situ generated and affects the site selectivity of C–H alkylation due to the sterically hindered feature of the Cl-boron complex in HAT process (see Figure 7c & 7d in Aggarwal's paper: *J. Am. Chem. Soc.* **2023**, *145*, 15207.). In the presence of B_2cat_2, control reaction revealed that the addition of benzylidenemalononitrile (**64**) as a typical radical acceptor greatly inhibited the desired C–H borylation reaction, while generating C–H alkylated product **65** preferentially at the weaker 3° C–H bond (the intrinsic selectivity of 1°/3° is still 1:3). Consistent with this finding, the addition of phenyl acrylate (**66**) into the photoelectrochemical system with DMB (**S9**) and B_2cat_2 (**2**) produced C–H alkylated product **67** in 65% yield with the same intrinsic selectivity of 1°/3° (1:6) as the reaction in the absence of B_2cat_2. These results suggested that free Cl radical HAT process could be much faster than the in situ generation of a Cl-boron complex under our photoelectrochemical conditions. Therefore, unlike Aggarwal's copper-mediated C(sp^3)−H borylation protocol in which the presence of copper species greatly facilitates the generation of a Cl-boron complex, under our photoelectrochemical conditions the free Cl radical HAT may dominate the HAT-mediated functionalization reactions.

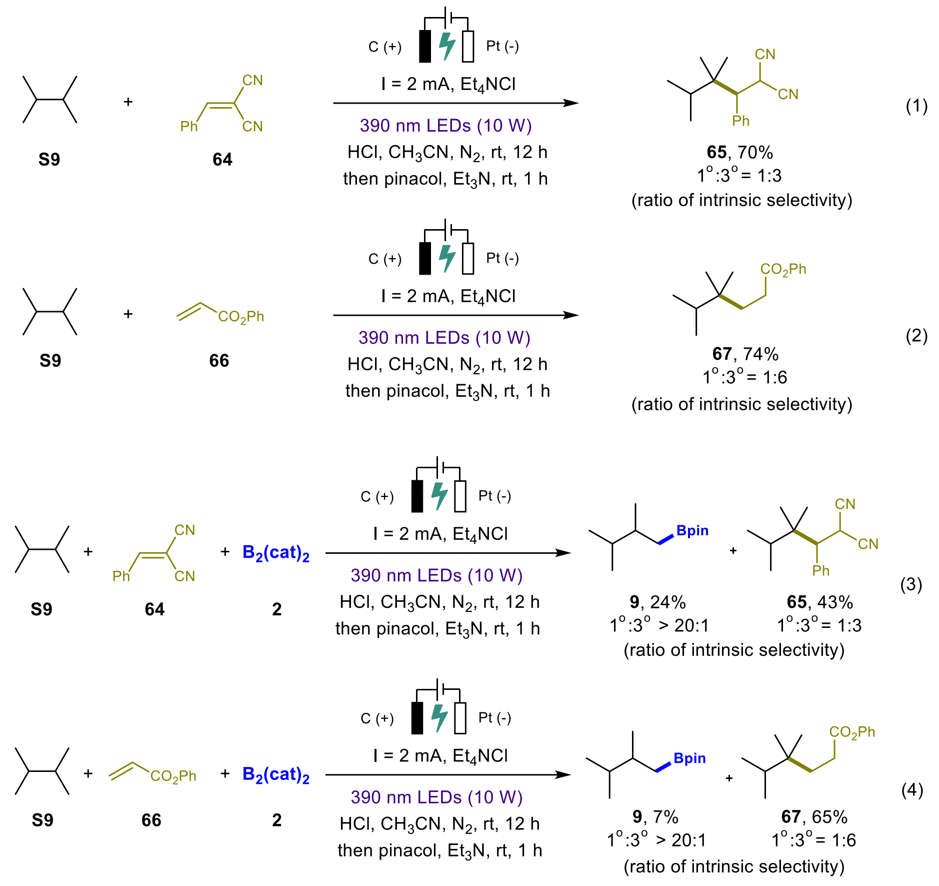

Supplementary Figure 38 Trapping reaction with alkenes

(2) Evidence for Cl-boron complexes as HAT species

As mentioned in Aggarwal's recent paper (*J. Am. Chem. Soc.* **2023**, *145*, 15207–15217), a key reaction intermediate, $O(Bcat)_2$, as a novel electrophilic borylating agent is proposed to react with free Cl radical and generate Cl-radical-boron "ate" complex, which is supposed to be the HAT species in that research work. The $O(Bcat)_2$ species is derived from the copper-catalyzed reaction of B_2cat_2 with H_2O (see the figure below).

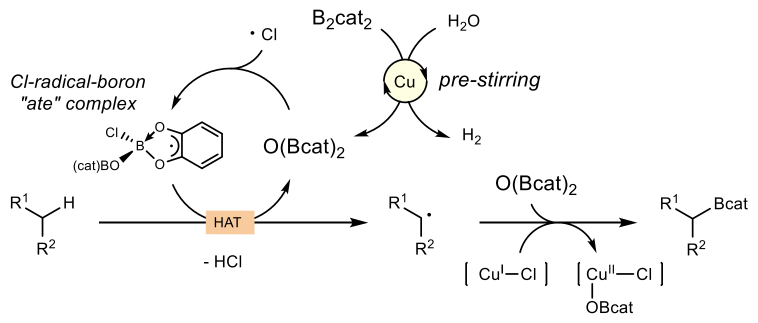

Supplementary Figure 39 Aggarwal's Cl-boron complex-mediated HAT process

Under our conditions, we also tried hard to capture these key intermediates, $O(Bcat)_2$ and HO-Bcat, in order to get evidence of Cl-boron complex as HAT species. In the presence of HCl (4 equiv.) and Et_4NCl (0.5 equiv.), weak signals of HO-Bcat in ^1H NMR spectra are likely to be observed (see Fig. S9). In comparison to Aggarwal's results where clear NMR signals of $O(Bcat)_2$ and HO-Bcat are successfully captured, we only observed weak signals of HO-Bcat probably due to the fact that the presence of metal species plays a key role in the cleavage of boron-boron bond and promotes the formation of $O(Bcat)_2$ species (also see: *J. Chem. Soc., Dalton Trans.* **1998**, 301–309).

Procedure: To three 10 mL vials equipped with stir bars was added $B_2(cat)_2$ (48mg, 0.2 mmol, 1.0 equiv.), Et_4NCl (0.1 mmol, 0.5 equiv.), and HCl (concentrated, 0.8 mmol, 4.0 equiv.) in the glovebox. MeCN (6 mL) was then added to the reaction system followed by the addition of cyclohexane (2.0 mmol, 10 equiv.). The reaction mixture was stirred for one hour and then directly take samples for NMR analysis. Supplementary Figure 40 shows that a small part of $B_2(cat)_2$ was hydrolyzed into HO-Bcat by ^1H NMR detection.

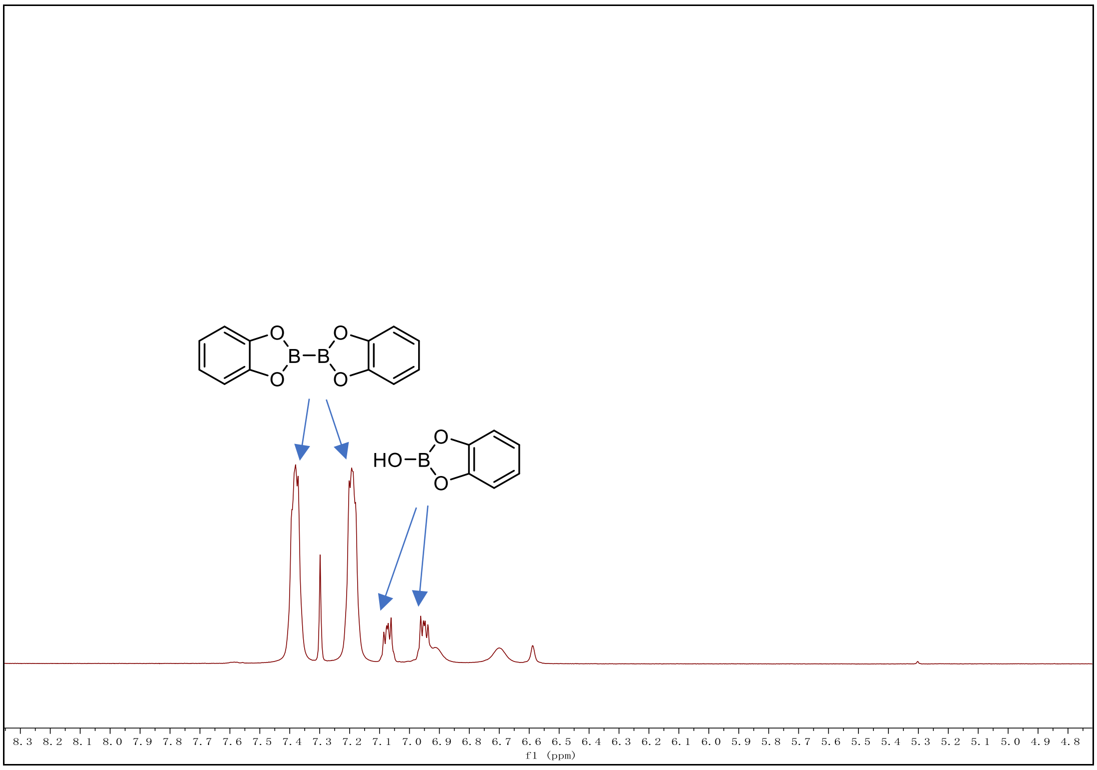

Supplementary Figure 40 ^1H NMR spectra of reaction mixtures (400 MHz, Chloroform-*d*)

To verify whether a Cl-boron complex can be generated in the photoelectrochemical system, a divided-cell experiment was carried out, as shown in the figure below. Substrates (**1**) and B_2cat_2 (**2**), along with Et_4NBF_4, ClBcat, and CH_3CN, was placed into the anode chamber, while Et_4NBF_4 and CH_3CN were added into the cathode chamber. As expected, upon light irradiation and electrolysis for 12 h the desired alkyl boronate **3** was detected in the anode chamber with 8% yield, suggesting that the photoelectrochemical $C(sp^3)$–H borylation occurred surrounding the graphite rod anode. Interestingly, an increased yield (24%) of alkyl boronate **3** was afforded in the presence of H_2O (5 μL) at the anode chamber, probably because the presence of H_2O promotes the generation of HO-Bcat species from Cl-Bcat, and HO-Bcat is the key precursor for the production of Cl-boron complex (Path A; generation of Cl-boron complex from HO-Bcat and Cl radical; Path B; generation of Cl-boron complex from $O(Bcat)_2$ and Cl radical) (Supplementary Figure 41).

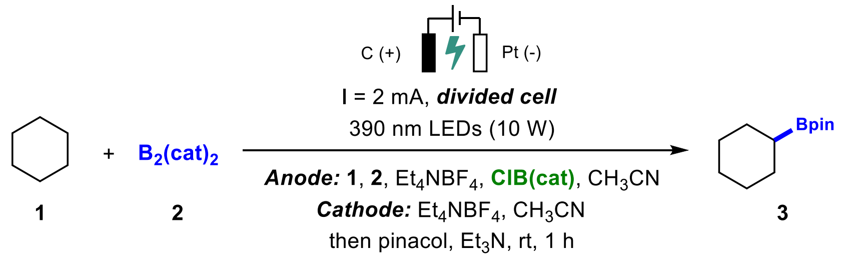

Entry	Deviation from standard conditions	Yield (%)
1	None	8
2	Adding H_2O (5 uL)	24

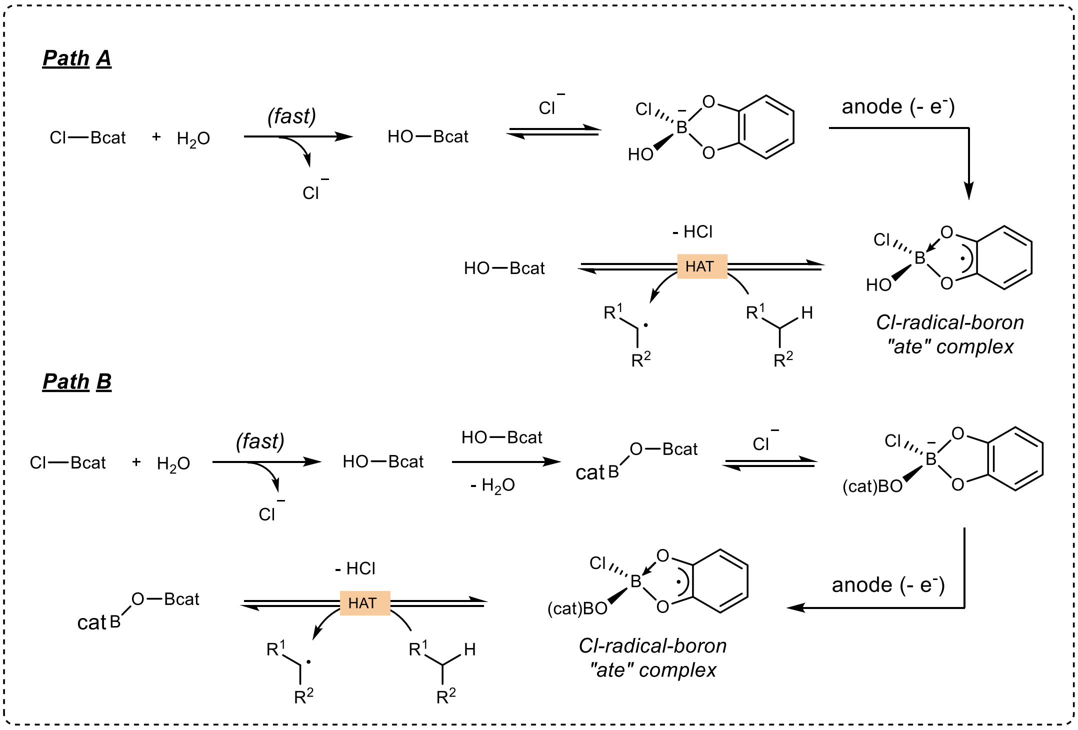

Supplementary Figure 41 Proposed reaction mechanism of HAT process mediated by Cl-boron complexes

Another evidence for the Cl-boron complex as HAT species is afforded by using squalane (**S59**) as substrate. The reaction of squalane (**S59**) with B_2cat_2 (**2**) under the standard photoelectrochemical conditions successfully delivered the desired C(sp^3)–H borylation product **59** in 43% yield with exclusive site selectivity. In the meantime we were also able to isolate a terminal alkene **59'** as a major side product, which is likely to be generated from 1° radical species followed by anodic oxidation and E1 elimination (see the figure below), while the generation of internal alkene **59''** was not observed.

Considering the fact that Cl radical preferentially undergoes HAT at the 3° C(sp^3)–H bonds and a Cl-boron complex preferentially undergoes HAT at the sterically unhindered 1° C(sp^3)–H bonds, the formation of this terminal alkene **59’** proves the existence of Cl-boron complex as HAT species in our photoelectrochemical system (Supplementary Figure 42).

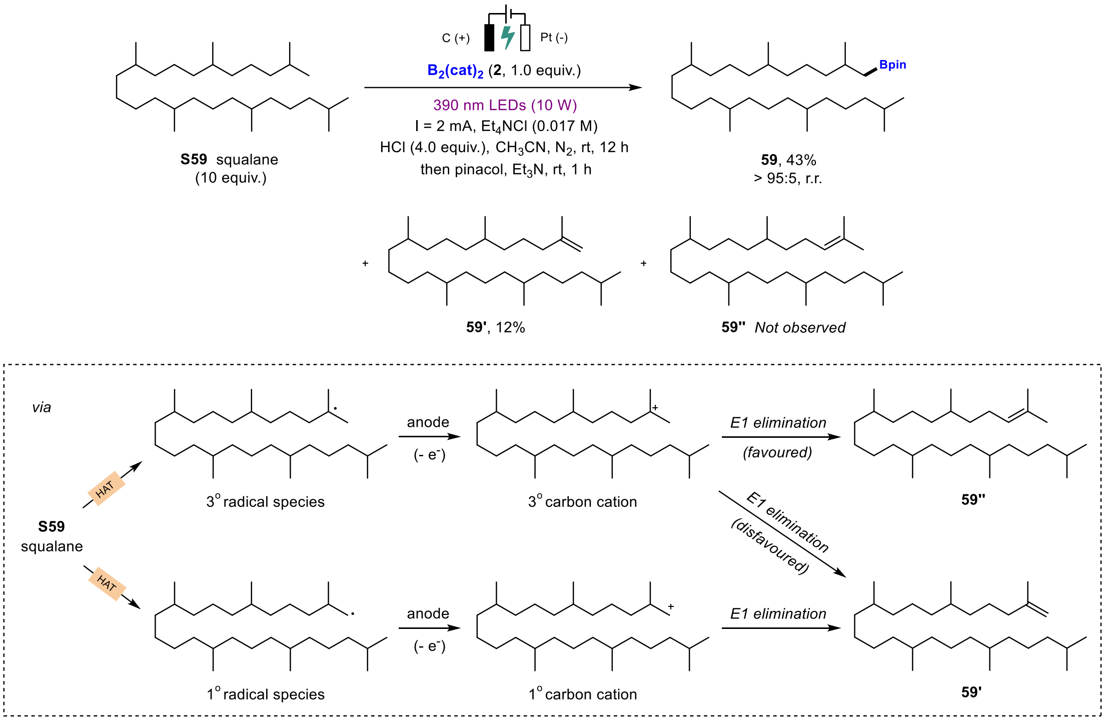

Supplementary Figure 42 Photoelectrochemical reaction of squalane

2,6,10,15,19,23-hexamethyltetracos-1-ene (59’)

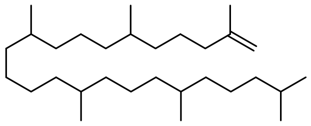

^{1}H NMR (400 MHz, Chloroform-*d*) δ 6.82-6.70 (m, 2H), 1.58 (s, 3H), 1.52 (td, *J* = 6.7, 2.4 Hz, 2H), 1.33 – 1.08 (m, 35H), 0.86-0.82 (m, 18H).

^{13}C NMR (101 MHz, Chloroform-*d*) δ 121.2, 108.6, 39.5, 37.5, 37.4, 32.9, 28.1, 27.6, 26.0, 24.9, 24.6, 22.9, 22.8, 19.9.

^{1}H NMR spectrum (400 MHz, $CDCl_3$)

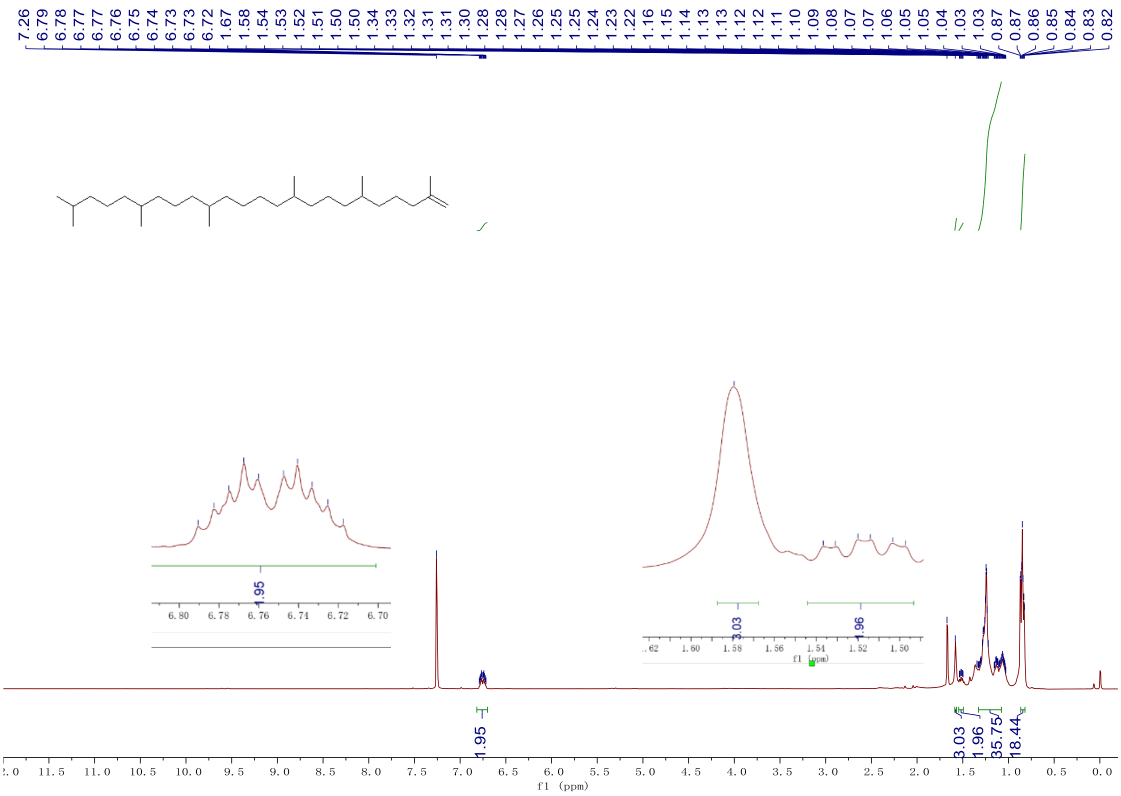

^{13}C NMR spectrum (101 MHz, $CDCl_3$)

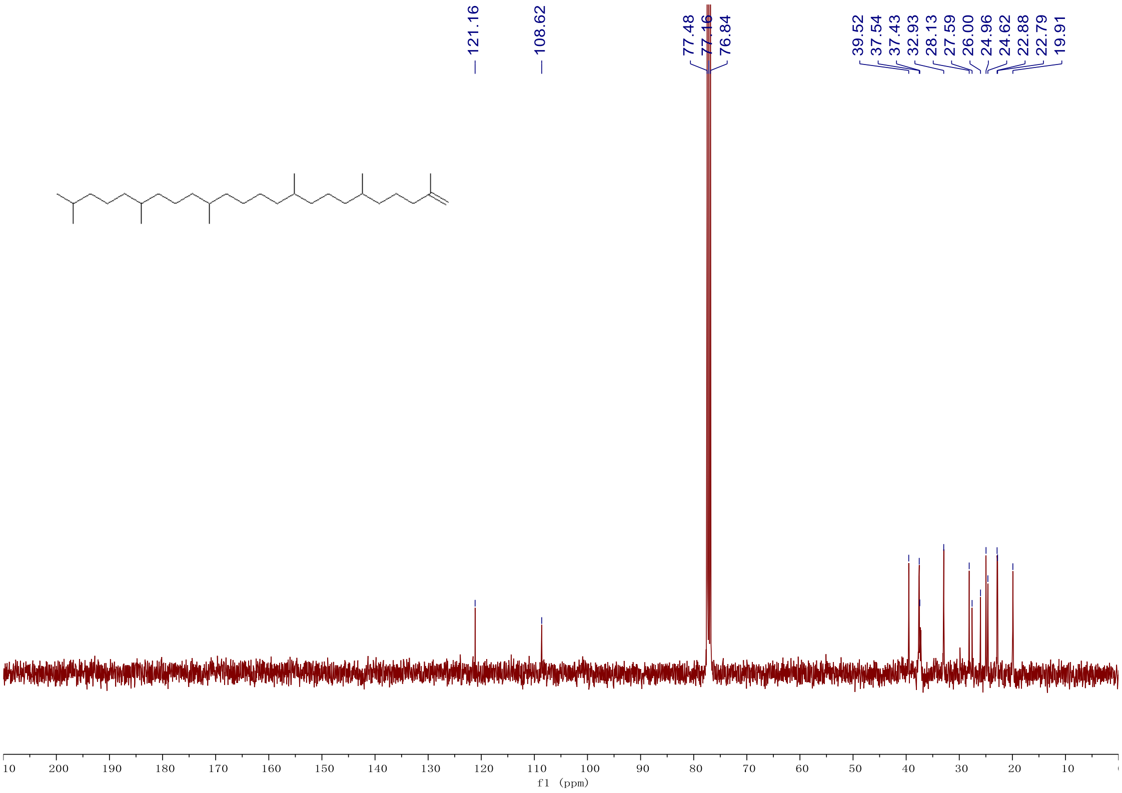

(3) Radical-mediated C(sp^3)−H borylation of *n*-pentane

Using benzylidenemalononitrile (**64**) and phenyl acrylate (**66**) as typical radical acceptors instead of B_2cat_2 (**2**) under our photoelectrochemical conditions, the preferential formation of 3° C–H alkylated products **65** and **67** has already revealed that site selectivity of C(sp^3)−H functionalization reaction is radical acceptor depended. In order to answer the reviewer's question about whether regioselectivity of C−H borylation is also determined by the HAT species, we further investigate C(sp^3)−H borylation reaction of *n*-pentane. Considering the differential features of Cl radical (secondary C−H seletivity) and Cl-boron complex (primary C−H seletivity) in the HAT process, we compare the regioselectivity results of currently existing protocols for C(sp^3)−H borylation of *n*-pentane. From the scheme below we can see that all the reactions show good distal methyl selectivity, which is mainly caused by the radical acceptor depended nature. If we look more closely, the C(sp^3)−H borylation reactions show better α-C(sp^3)−H selectivity in the presence of metal species. It may be due to the fact that the presence of metal species plays a key role in the cleavage of boron-boron bond of B_2cat_2 and promotes the formation of key Cl-boron complex. Considering that HAT is a fast process in our photoelectrochemical system (KIE: k_H/k_D = 1.0) as well as the fact that 33% yield of secondary boronate is generated, this result suggests that both free Cl radical and Cl-boron complex as HAT species could exist in our reaction system.

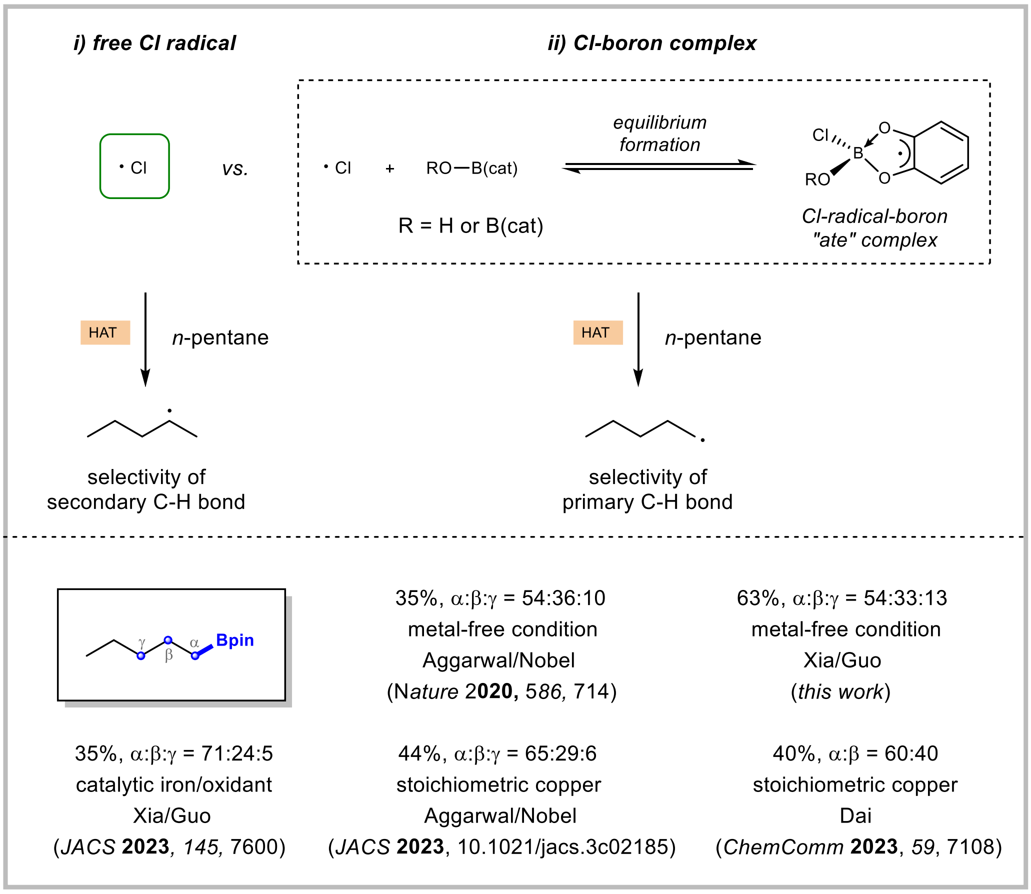

Supplementary Figure 43 Discussion of regioselectivity for *n*-pentane

In addition, we found that the unconventional selectivity of C(sp^3)–H borylation reaction is not only determined by HAT species or radical acceptor. There are many other factors that could greatly affect the site selectivity of the photoelectrochemical reaction, such as equivalents of borylating agent, alkane substrate, HCl, and concentration of the reaction. Please see the figure below, as well as 2.12. Regioselectivity studies with n-pentane.

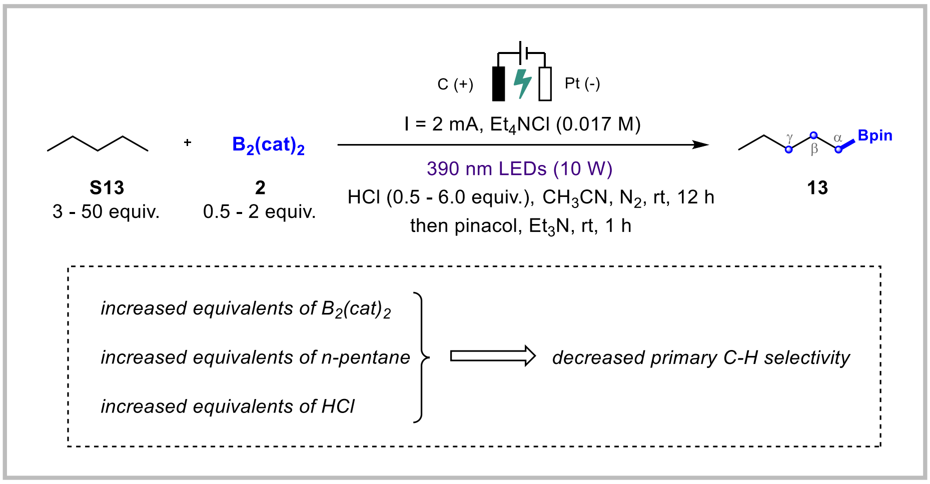

Supplementary Figure 44 Trends in regioselectivity for *n*-pentane

(4) Conclusion

Based on the above results, we propose that free Cl radical-mediated and Cl-boron complex-mediated HAT processes are both likely to exist in our photoelectrochemical system. We have corrected the proposed mechanism, as shown in the scheme below as well as in the revised manuscript (see Fig. 6). On the basis of fast and reversible HAT and the trapping experiments with benzylidenemalononitrile and phenyl acrylate, we believe that the free Cl radical-mediated HAT is the dominant during our photoelectrochemical reaction process.

Based on the above mechanistic studies and relevant literature reports, we propose the plausible mechanism outlined in Fig. S10. First, the Cl^- is electrochemically oxidized to Cl_2 at the anode surface, and then the chlorine radical species (**A**) is generated via light-promoted homolytic cleavage of Cl_2. According to its inherent selectivity, the chlorine radical undergoes HAT process with $C(sp^3)$–H compounds (taking **S9** as an example) to initially release a more substituted carbon-centered radical (**B**), which could not proceed with the C–H borylation reaction at the tertiary site, probably due to steric hindrance. Since the alkyl radical formation is reversible (or the alkyl radical can "isomerize" by performing the subsequent reversible HAT with other molecules of the substrate) and fast, a sterically unhindered primary radical (**D**) is generated and trapped by $B_2(cat)_2$ to give an alkyl boronate ester (**E**) as well as the ligated boryl radical (**F**)

(*Path A*). Alternatively, the HO-Bcat (**G**) generated by hydrolysis and anodic oxidation in the reaction system can be complexed with Cl radical species (**A**) to obtain Cl-radical-boron “ate” complex (**H**), followed by the HAT process to obtain carbon center radical (**D**) (*Path B*). Treating intermediate **E** with pinacol and triethylamine finally delivers the desired product **9**. At the cathode surface, protons undergo cathodic reduction to generate H_2, obviating the need for sacrificial oxidants.

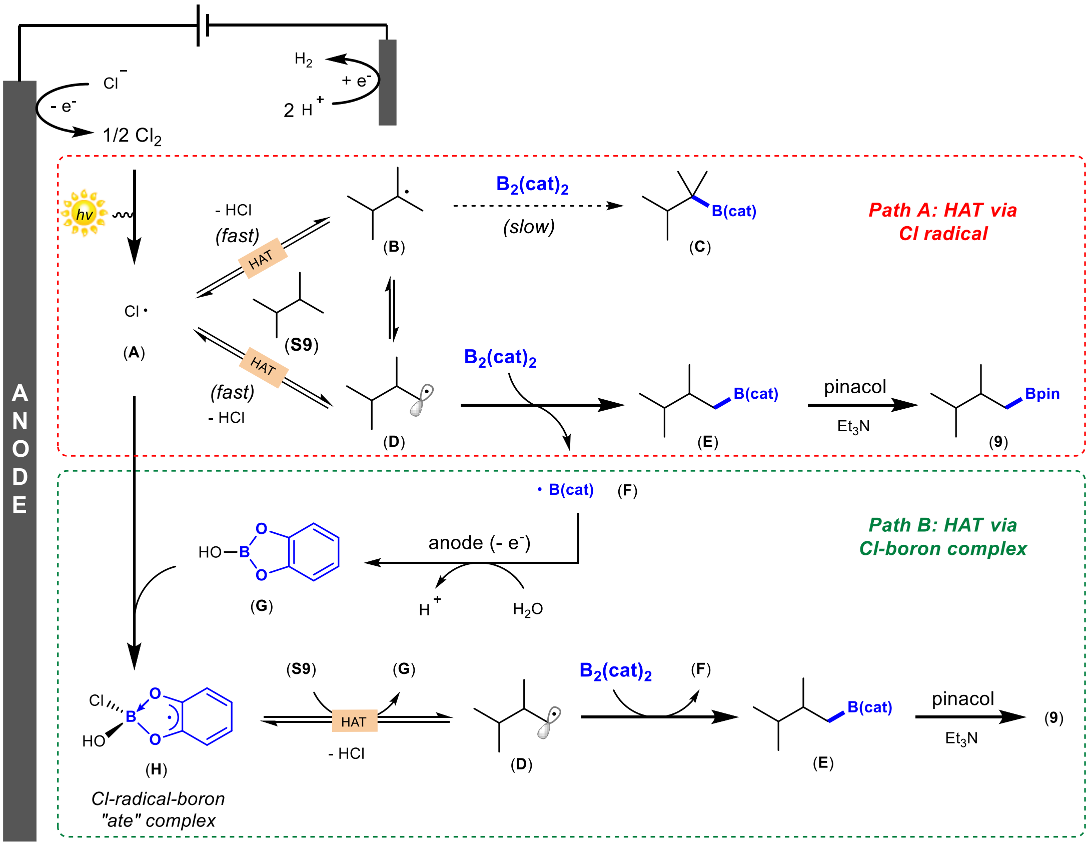

Supplementary Figure 45 Proposed reaction mechanism

3. Product Characterization

2-Cyclohexyl-4,4,5,5-tetramethyl-1,3,2-dioxaborolane (3)

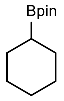

Prepared following **General Procedure A** using cyclohexane (169 mg, 2.00 mmol, 10.0 equiv.). Flash column chromatography (5% EtOAc/ Petroleum ether) gave **3** (28.5 mg, 0.14 mmol, 68%) as a colourless liquid. All recorded spectroscopic data matched those previously reported in the literature[8].

^{1}H NMR (400 MHz, Chloroform-*d*) δ 1.69 – 1.53 (m, 5H), 1.37 – 1.25 (m, 5H), 1.21 (d, *J* = 4.5 Hz, 12H), 0.1.02 – 0.89 (m, 1H). See spectrum

^{13}C NMR (101 MHz, Chloroform-*d*) δ 82.9, 28.1, 27.3, 26.9, 24.9. *The carbon attached to boron could not be observed due to quadrupolar relaxation*. See spectrum

2-Cyclopentyl-4,4,5,5-tetramethyl-1,3,2-dioxaborolane (4)

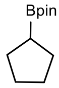

Prepared following **General Procedure A** using cyclopentane (140 mg, 2.00 mmol, 10.0 equiv.). Flash column chromatography (5% EtOAc/ Petroleum ether) gave **4** (26.3 mg, 0.13 mmol, 67%) as a colourless liquid. All recorded spectroscopic data matched those previously reported in the literature[8].

^{1}H NMR (400 MHz, Chloroform-*d*) δ 1.82 – 1.68 (m, 2H), 1.64 – 1.56 (m, 2H), 1.55 – 1.39 (m, 4H), 1.23 (d, J = 0.9 Hz, 12H), 1.20 – 1.13 (m, 1H). See spectrum

^{13}C NMR (101 MHz, Chloroform-*d*) δ 82.9, 28.7, 27.0, 24.9. *The carbon attached to boron could not be observed due to quadrupolar relaxation*. See spectrum

2-Cycloheptyl-4,4,5,5-tetramethyl-1,3,2-dioxaborolane (5)

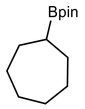

Prepared following **General Procedure A** using cycloheptane (193 mg, 2.00 mmol, 10.0 equiv.). Flash column chromatography (5% EtOAc/ Petroleum ether) gave **5** (28.7 mg, 0.13 mmol, 64%) as a colourless liquid. All recorded spectroscopic data matched those previously reported in the literature[8].

^{1}H NMR (400 MHz, Chloroform-*d*) δ 1.77 - 1.41 (m, 12H), 1.22 (d, *J* = 5.8 Hz, 12H), 1.10 – 1.02 (m, 1H). See spectrum

^{13}C NMR (101 MHz, Chloroform-*d*) δ 82.9, 29.7, 29.1, 28.5, 24.8. *The carbon attached to boron could not be observed due to quadrupolar relaxation*. See spectrum

2-Cyclooctyl-4,4,5,5-tetramethyl-1,3,2-dioxaborolane (6)

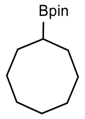

Prepared following **General Procedure A** using cyclooctane (225 mg, 2.00 mmol, 10.0 equiv.). Flash column chromatography (5% EtOAc/ Petroleum ether) gave **6** (26.7 mg, 0.11 mmol, 56%) as a colourless liquid. All recorded spectroscopic data matched those previously reported in the literature[8].

^{1}H NMR (400 MHz, Chloroform-*d*) δ 1.77 – 1.64 (m, 3H), 1.59 – 1.43 (m, 11H), 1.23 (s, 12H), 1.14 – 1.06 (m, 1H). See spectrum

^{13}C NMR (101 MHz, Chloroform-*d*) δ 82.9, 27.7, 27.1, 26.98 , 26.8, 24.9. *The carbon attached to boron could not be observed due to quadrupolar relaxation*. See spectrum

2-Cyclododecyl-4,4,5,5-tetramethyl-1,3,2-dioxaborolane (7)

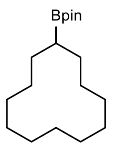

Prepared following **General Procedure A** using cyclododecane (336 mg, 2.00 mmol, 10.0 equiv.). Flash column chromatography (5% EtOAc/ Petroleum ether) gave **7** (29.5 mg, 0.10 mmol, 50%) as a colourless liquid.

^{1}H NMR (400 MHz, Chloroform-*d*) δ 1.43 – 1.32 (m, 22H), 1.23 (s, 12H), 1.10 – 1.01 (m, 1H). See spectrum

^{13}C NMR (101 MHz, Chloroform-*d*) δ 82.9, 25.1 24.9, 24.4, 24.3, 23.8, 23.6, 23.6, 23.5. *The carbon attached to boron could not be observed due to quadrupolar relaxation*. See spectrum

HRMS (ESI^{+}) calcd. for $C_{18}H_{36}BO_2^{+}$ [M+H]$^{+}$,M/Z: 295.2803, found 295.2802.

2-((1*r*,3*r*,5*r*,7*r*)-Adamantan-2-yl)-4,4,5,5-tetramethyl-1,3,2-dioxaborolane (8-α) and 2-((3*r*,5*r*,7*r*)-adamantan-1-yl)-4,4,5,5-tetramethyl-1,3,2-dioxaborolane (8-β)

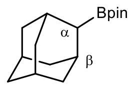

8-α
(major)

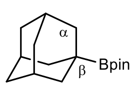

8-β
(minor)

Prepared following **General Procedure A** using adamantane (270 mg, 2.00 mmol, 10.0 equiv.). Flash column chromatography (5% EtOAc/ Petroleum ether) gave **8** (24.7 mg, 0.09 mmol, 47%, **8-α** : **8-β** = 86 : 14) as a colourless liquid. The r.r. was determined by GC analysis of the crude reaction mixture (see spectrum).

^{1}H NMR (400 MHz, Chloroform-*d*) δ 2.05 (s, 2H), 1.91 – 1.64 (m, 14H), 1.37 (t, J = 2.3 Hz, 1H), 1.25 (d, *J* = 2.6 Hz, 12H), 1.21 (d, *J* = 2.7 Hz, 2H). See spectrum

^{13}C NMR (101 MHz, Chloroform-*d*) δ 82.9, 39.5, 38.1, 37.9, 37.6, 36.4, 29.5, 28.4, 28.3, 27.7, 25.0, 24.8. *The carbon attached to boron could not be observed due to quadrupolar relaxation*. See spectrum

HRMS (ESI^{+}) calcd. for $C_{16}H_{26}BO_2^{+}$[M+H]$^{+}$,M/Z:263.2177, found 263.2173.

2-(2,3-Dimethylbutyl)-4,4,5,5-tetramethyl-1,3,2-dioxaborolane (9)

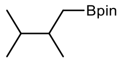

Prepared following **General Procedure A** using 2,3-dimethylbutane (173 mg, 2.00 mmol, 10.0 equiv.). Flash column chromatography (5% EtOAc/ Petroleum ether) gave **9** (22.9 mg, 0.11 mmol, 54%, > 95:5 r.r.) as a colourless liquid. The r.r. was determined by ^{1}H NMR analysis of the crude reaction mixture. The r.r. was determined by GC analysis of the crude reaction mixture (*see spectrum*). All recorded spectroscopic data matched those previously reported in the literature[8].

^{1}H NMR (400 MHz, Chloroform-*d*) δ 1.60 – 1.54 (m, 1H), 1.52 – 1.41 (m, 1H), 1.24 (d, *J* = 1.7 Hz, 12H), 0.87 – 0.79 (m, 10H), 0.67 – 0.53 (m, 1H). *See spectrum*

^{13}C NMR (101 MHz, Chloroform-*d*) δ 83.0, 35.3, 34.4, 25.1, 24.9, 19.9, 18.8, 18.7. *The carbon attached to boron could not be observed due to quadrupolar relaxation*. *See spectrum*

HRMS (ESI$^+$) calcd. for $C_{12}H_{26}BO_2^+$ [M+H]$^+$,M/Z: 213.2020, found 213.2018.

2-(2,4-Dimethylpentyl)-4,4,5,5-tetramethyl-1,3,2-dioxaborolane (10)

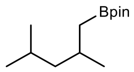

Prepared following **General Procedure A** using 2,4-dimethylpentane (200 mg, 2.00 mmol, 10.0 equiv.). Flash column chromatography (5% EtOAc/ Petroleum ether) gave **10** (24.1 mg, 0.11 mmol, 53%, > 95:5 r.r.) as a colourless liquid. The r.r. was determined by ^{1}H NMR analysis of the crude reaction mixture. All recorded spectroscopic data matched those previously reported in the literature[8].

^{1}H NMR (400 MHz, Chloroform-*d*) δ 1.81 – 1.70 (m, 1H), 1.63 – 1.55 (m, 1H), 1.24 (s, 12H), 1.10 – 1.01 (m, 2H), 0.88 (d, *J* = 6.6 Hz, 3H), 0.84 (dd, *J* = 6.6, 2.5 Hz, 6H), 0.82 – 0.76 (m, 1H), 0.66 – 0.58 (m, 1H). *See spectrum*

^{13}C NMR (101 MHz, Chloroform-*d*) δ 82.9, 49.5, 27.2, 25.6, 25.0, 24.9, 23.4, 22.6. *The carbon attached to boron could not be observed due to quadrupolar relaxation*. *See spectrum*

HRMS (ESI^+) calcd. for $C_{13}H_{28}BO_2^+$ $[M+H]^+$,M/Z: 227.2177, found 227.2178.

4,4,5,5-Tetramethyl-2-(2,4,4-trimethylpentyl)-1,3,2-dioxaborolane (11)

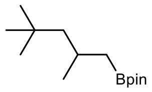

Prepared following **General Procedure A** using 2,2,4-trimethylpentane (228 mg, 2.00 mmol, 10.0 equiv.). Flash column chromatography (5% EtOAc/ Petroleum ether) gave **11** (25.9 mg, 0.11 mmol, 54%, > 95:5 r.r.) as a colourless liquid. The r.r. was determined by ^1H NMR analysis of the crude reaction mixture. The r.r. was determined by GC analysis of the crude reaction mixture (*See spectrum*). All recorded spectroscopic data matched those previously reported in the literature[8].

^1H NMR (400 MHz, Chloroform-*d*) δ 1.88 – 1.76 (m, 1H), 1.24 (s, 12H), 1.21 – 1.17 (m, 1H), 1.15 – 1.09 (m, 1H), 0.95 (d, J = 6.8 Hz, 3H), 0.89 (s, 9H), 0.84 – 0.79 (m, 1H), 0.74 – 0.66 (m, 1H). *See spectrum*

^{13}C NMR (101 MHz, Chloroform-*d*) δ 82.9, 53.9, 31.3, 30.4, 26.2, 25.3, 25.1, 25.0, 24.9. *The carbon attached to boron could not be observed due to quadrupolar relaxation*. *See spectrum*

HRMS (ESI^+) calcd. for $C_{14}H_{30}BO_2^+$ $[M+H]^+$,M/Z: 241.2333, found 241.2334.

2-(3,3-Dimethylbutyl)-4,4,5,5-tetramethyl-1,3,2-dioxaborolane (12)

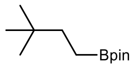

Prepared following **General Procedure A** using 2,2-dimethylbutane (176 mg, 2.00 mmol, 10.0 equiv.). Flash column chromatography (5% EtOAc/Petroleum ether) gave

12 (20.8 mg, 0.10 mmol, 49%, > 95:5 r.r.) as a colourless liquid. The r.r. was determined by ^{1}H NMR analysis of the crude reaction mixture.

^{1}H NMR (400 MHz, Chloroform-*d*) δ 1.32 – 1.27 (m, 2H), 1.24 (s, 12H), 0.84 (s, 9H), 0.75 – 0.67 (m, 2H). *See spectrum*

^{13}C NMR (101 MHz, Chloroform-*d*) δ 82.9, 37.8, 30.9, 28.9, 24.9. *The carbon attached to boron could not be observed due to quadrupolar relaxation. See spectrum*

HRMS (ESI^{+}) calcd. for $C_{12}H_{26}BO_2^{+}$ $[M+H]^{+}$,M/Z: 213.2020, found 213.2017.

4,4,5,5-Tetramethyl-2-pentyl-1,3,2-dioxaborolane (13-α), 4,4,5,5-tetramethyl-2-(pentan-2-yl)-1,3,2-dioxaborolane (13-β), and 4,4,5,5-tetramethyl-2-(pentan-3-yl)-1,3,2-dioxaborolane (13-γ)

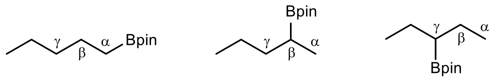

13-α (major) **13**-β **13**-γ

Prepared following **General Procedure A** using pentane (144 mg, 2.00 mmol, 10.0 equiv.). Flash column chromatography (5% EtOAc/ Petroleum ether) gave **13** (24.9 mg, 0.13 mmol, 63%, **13-α** : **13-β** : **13-γ** = 54:33:13) as a colourless liquid. The r.r. was determined by GC analysis of the crude reaction mixture (*See spectrum*). All recorded spectroscopic data matched those previously reported in the literature[8].

^{1}H NMR (400 MHz, Chloroform-*d*) δ 1.45 – 1.36 (m, 4H), 1.33 – 1.26 (m, 8H), 1.24 (s, 22H), 0.95 (d, *J* = 6.4 Hz, 3H), 0.92 – 0.82 (m, 12H), 0.76 (t, *J* = 7.8 Hz, 2H). *See spectrum*

^{13}C NMR (101 MHz, Chloroform-*d*) δ 83.0, 82.9, 82.9, 35.6, 34.8, 25.0, 24.9, 24.8, 24.1, 23.8, 22.8, 22.6, 22.2, 15.6, 14.5, 14.3, 14.2, 13.8. *The carbon attached to boron could not be observed due to quadrupolar relaxation. See spectrum*

HRMS (ESI^{+}) calcd. for $C_{11}H_{24}BO_2^{+}$ $[M+H]^{+}$,M/Z: 199.1864, found 199.1861.

2-((1*R*,2*R*,4*R*)-Bicyclo[2.2.1]heptan-2-yl)-4,4,5,5-tetramethyl-1,3,2-dioxaborolane (14)

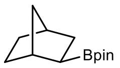

Prepared following **General Procedure A** using (1s,4s)-bicyclo[2.2.1]heptane (192 mg, 2.00 mmol, 10.0 equiv.). Flash column chromatography (5% EtOAc/Petroleum ether) gave **14** (26.7 mg, 0.12 mmol, 60%, > 95:5 r.r., > 95:5 *exo*:*endo*) as a colourless liquid. The r.r. was determined by GC analysis of the crude reaction mixture (See spectrum). All recorded spectroscopic data matched those previously reported in the literature[8].

^{1}H NMR (400 MHz, Chloroform-*d*) δ 2.28 – 2.25 (m, 1H), 2.22 – 2.17 (m, 1H), 1.55 – 1.45 (m, 3H), 1.36 – 1.30 (m, 1H), 1.23 – 1.12 (m, 16H), 0.89 – 0.84 (m, 1H). *See spectrum*

^{13}C NMR (101 MHz, Chloroform-*d*) δ 82.9, 38.8, 38.3, 36.8, 32.4, 32.3, 29.4, 24.8. *The carbon attached to boron could not be observed due to quadrupolar relaxation*. *See spectrum*

HRMS (ESI^{+}) calcd. for $C_{13}H_{24}BO_2^{+}$ [M+H]$^{+}$,M/Z: 223.1864, found 223.1867.

2-(4-Chloro-2-methylbutyl)-4,4,5,5-tetramethyl-1,3,2-dioxaborolane (15)

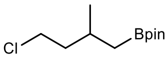

Prepared following **General Procedure A** using 1-chloro-3-methylbutane (210 mg, 2.00 mmol, 10.0 equiv.). Flash column chromatography (5% EtOAc/Petroleum ether) gave **15** (26.4 mg, 0.12 mmol, 57%, > 95:5 r.r.) as a colourless liquid. The r.r. was determined by ^{1}H NMR analysis of the crude reaction mixture. All recorded spectroscopic data matched those previously reported in the literature[8].

^{1}H NMR (400 MHz, Chloroform-*d*) δ 3.61 – 3.48 (m, 2H), 1.97 – 1.85 (m, 1H), 1.83 – 1.71 (m, 1H), 1.71 – 1.61 (m, 1H), 1.24 (s, 12H), 0.95 (d, *J* = 6.6 Hz, 3H), 0.87 – 0.83 (m, 1H), 0.73 – 0.65 (m, 1H). *See spectrum*

^{13}C NMR (101 MHz, Chloroform-*d*) δ 83.1, 43.4, 42.1, 27.2, 24.9, 24.8, 21.8. *The carbon attached to boron could not be observed due to quadrupolar relaxation*. <u>*See spectrum*</u>

HRMS (ESI$^+$) calcd. for $C_{11}H_{23}BClO_2^+$ [M+H]$^+$,M/Z: 233.1474, found 233.1470.

2-(3-Chloro-2-methylpropyl)-4,4,5,5-tetramethyl-1,3,2-dioxaborolane (16)

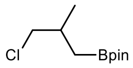

Prepared following **General Procedure A** using 1-chloro-2-methylpropane (184 mg, 2.00 mmol, 10.0 equiv.). Flash column chromatography (5% EtOAc/Petroleum ether) gave **16** (21.8 mg, 0.10 mmol, 50%, > 95:5 r.r.) as a colourless liquid. The r.r. was determined by ^1H NMR analysis of the crude reaction mixture. All recorded spectroscopic data matched those previously reported in the literature[8].

^1H NMR (400 MHz, Chloroform-*d*) δ 3.51 – 3.38 (m, 2H), 2.14 – 2.04 m, 1H), 1.24 (s, 12H), 1.04 (d, *J* = 6.7 Hz, 3H), 1.01 – 0.93 (m, 1H), 0.82 – 0.72 (m, 1H). <u>*See spectrum*</u>

^{13}C NMR (101 MHz, Chloroform-*d*) δ 83.3, 53.1, 32.4, 25.0, 24.9, 20.2. *The carbon attached to boron could not be observed due to quadrupolar relaxation*. <u>*See spectrum*</u>

HRMS (ESI$^+$) calcd. for $C_{10}H_{21}BClO_2^+$ [M+H]$^+$,M/Z: 219.1318, found 219.1317.

2-(3-Chlorocyclopentyl)-4,4,5,5-tetramethyl-1,3,2-dioxaborolane (17)

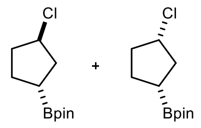

17-*trans* **17**-*cis*

Prepared following **General Procedure A** using chlorocyclopentane (206 mg, 2.00 mmol, 10.0 equiv.). Flash column chromatography (5% EtOAc/Petroleum ether) gave **17** (24.9 mg, 0.11 mmol, 54%) as a colourless liquid. The r.r. (> 95:5 r.r.) and d.r.

(*trans:cis* = 67:33) was determined by ^{1}H NMR and GC analysis of the crude reaction mixture (See spectrum).

^{1}H NMR (400 MHz, Chloroform-*d*) δ (*67:33 trans:cis*) 4.45 (m, 0.67H), 4.26 – 4.33 (m, 0.33H), 2.32 – 2.24 (m, 0.33 H), 2.11 – 1.86 (m, 5H), 1.77 – 1.68 (m, 0.67H), 1.63 – 1.56 (m, 0.67H), 1.42 – 1.31 (m, 0.67H), 1.24 (s, 4H), 1.23 (s, 8H). *See spectrum*

^{13}C NMR (101 MHz, Chloroform-*d*) δ(*67:33 trans:cis*) 83.4 *(cis)*, 83.3*(trans)*, 63.3*(trans)*, 62.0*(cis)*, 39.6*(trans)*, 39.5*(cis)*, 37.8*(cis)*, 37.7*(trans)*, 25.7*(trans)*, 25.3*(cis)*, 24.9(*67:33 trans:cis*). *The carbon attached to boron could not be observed due to quadrupolar relaxation. See spectrum*

HRMS (ESI^{+}) calcd. for $C_{11}H_{21}BClO_2^{+}$ $[M+H]^{+}$ 231.1318, found 231.1317.

2-(2-(3,5-Diisopropylphenyl)propyl)-4,4,5,5-tetramethyl-1,3,2-dioxaborolane (18)

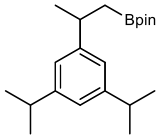

Prepared following **General Procedure A** using 1,3,5-triisopropylbenzene (406 mg, 2.00 mmol, 10.0 equiv.). Flash column chromatography (5% EtOAc/Petroleum ether) gave **18** (24.4 mg, 0.10 mmol, 52%, > 95:5 r.r.) as a colourless liquid. The r.r. was determined by ^{1}H NMR analysis of the crude reaction mixture.

^{1}H NMR (400 MHz, Chloroform-*d*) δ 6.93 (d, *J* = 1.7 Hz, 2H), 6.88 (t, *J* = 1.7 Hz, 1H), 3.05 – 2.95 (m, 1H), 2.92 – 2.80 (m, 2H), 1.27 (dd, *J* = 6.9, 3.6 Hz, 3H), 1.30 – 1.25 (m, 1H), 1.24 (d, *J* = 6.9 Hz, 12H), 1.19 – 1.14 (m, 1H),1.16 (d, *J* = 1.9 Hz, 12H). *See spectrum*

^{13}C NMR (101 MHz, Chloroform-*d*) δ 149.1, 148.5, 122.3, 122.1, 82.9, 36.0, 34.3, 25.1, 24.8, 24.7, 24.2, 24.2. *The carbon attached to boron could not be observed due to quadrupolar relaxation. See spectrum*

HRMS (ESI^{+}) calcd. for $C_{12}H_{36}BO_2^{+}$ $[M+H]^{+}$,M/Z: 331.2803, found 331.2801.

4,4,5,5-Tetramethyl-2-(2-phenylpropyl)-1,3,2-dioxaborolane (19)

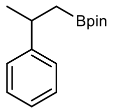

Prepared following **General Procedure A** using cumene (238 mg, 2.00 mmol, 10.0 equiv.). Flash column chromatography (5% EtOAc/Petroleum ether) gave **19** (19.2 mg, 0.08 mmol, 39%, > 95:5 r.r.) as a colourless liquid. The r.r. was determined by ^{1}H NMR analysis of the crude reaction mixture.

^{1}H NMR (400 MHz, Chloroform-*d*) δ 7.29 – 7.21 (m, 4H), 7.17 – 7.11 (m, 1H), 3.09 – 2.96 (m, 1H), 1.32 – 1.25 (m, 1H), 1.27 (d, *J* = 7.0 Hz, 3H), 1.16 (s, 12H). <u>*See spectrum*</u>

^{13}C NMR (101 MHz, Chloroform-*d*) δ 149.4, 128.3, 126.8, 125.8, 83.1, 35.9, 25.0, 24.9, 24.8. *The carbon attached to boron could not be observed due to quadrupolar relaxation*. <u>*See spectrum*</u>

HRMS (ESI^{+}) calcd. for $C_{15}H_{24}BO_2^{+}$ [M+H]$^{+}$,M/Z: 247.1864, found 247.1867.

Dimethyl(phenyl)((4,4,5,5-tetramethyl-1,3,2-dioxaborolan-2-yl)methyl)silane (20)

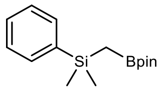

Prepared following **General Procedure A** using trimethyl(phenyl)silane (298 mg, 2.00 mmol, 10.0 equiv.). Flash column chromatography (5% EtOAc/Petroleum ether) gave **20** (24.3 mg, 0.09 mmol, 44%) as a colourless liquid. All recorded spectroscopic data matched those previously reported in the literature[8].

^{1}H NMR (400 MHz, Chloroform-*d*) δ 7.58 – 7.51 (m, 2H), 7.37 – 7.30 (m, 3H), 1.18 (s, 12H), 0.36 (s, 2H), 0.33 (s, 6H). <u>*See spectrum*</u>

^{13}C NMR (101 MHz, Chloroform-*d*) δ 140.3, 133.6, 128.9, 127.8, 83.0, 25.0, 0.7. *The carbon attached to boron could not be observed due to quadrupolar relaxation*. <u>*See spectrum*</u>

HRMS (ESI^+) calcd. for $C_{15}H_{26}BO_2Si^+$ $[M+H]^+$,M/Z: 277.1790, found 277.1795.

Benzyldimethyl((4,4,5,5-tetramethyl-1,3,2-dioxaborolan-2-yl)methyl)silane (21)

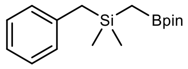

Prepared following **General Procedure A** using benzyltrimethylsilane (326 mg, 2.00 mmol, 10.0 equiv.). Flash column chromatography (5% EtOAc/Petroleum ether) gave **21** (36.5 mg, 0.13 mmol, 63%, > 95:5 r.r.) as a colourless liquid. The r.r. was determined by ^{1}H NMR analysis of the crude reaction mixture. All recorded spectroscopic data matched those previously reported in the literature[8].

^{1}H NMR (400 MHz, Chloroform-*d*) δ 7.20 (t, *J* = 7.6, 2H), 7.07 (d, *J* = 7.4, 1H), 7.04 – 7.00 (m, 2H), 2.13 (s, 2H), 1.25 (s, 12H), 0.10 (s, 2H), 0.03 (s, 6H). See spectrum

^{13}C NMR (101 MHz, Chloroform-*d*) δ 140.4, 128.3, 128.3, 124.0, 83.0, 27.4, 25.1, -1.7. *The carbon attached to boron could not be observed due to quadrupolar relaxation*. See spectrum

HRMS (ESI^+) calcd. for $C_{16}H_{26}BO_2Si^+$ $[M+H]^+$,M/Z: 291.1946, found 291.1945.

(4-(Dimethyl((4,4,5,5-tetramethyl-1,3,2-dioxaborolan-2-yl)methyl)silyl)phenyl)trimethylsilane (22)

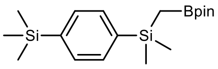

Prepared following **General Procedure A** using 1,4-bis(trimethylsilyl)benzene (442 mg, 2.00 mmol, 10.0 equiv.). Flash column chromatography (5% EtOAc/Petroleum ether) gave **22** (37.9 mg, 0.09 mmol, 46%) as a colourless liquid.

^{1}H NMR (400 MHz, Chloroform-*d*) δ 7.59 – 7.51 (m, 4H), 1.21 (s, 12H), 0.39 (s, 2H), 0.36 (s, 6H), 0.29 (s, 9H). See spectrum

^{13}C NMR (101 MHz, Chloroform-*d*) δ 141.0, 140.8, 132.8, 132.6, 82.9, 25.0, -1.1. *The carbon attached to boron could not be observed due to quadrupolar relaxation*. *See spectrum*

HRMS (ESI$^+$) calcd. for $C_{18}H_{34}BO_2Si_2^+$ [M+H]$^+$,M/Z: 349.2185, found 349.2185.

1,1,1,2,2-Pentamethyl-2-((4,4,5,5-tetramethyl-1,3,2-dioxaborolan-2-yl)methyl)disilane (23)

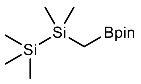

Prepared following **General Procedure A** using 1,1,1,2,2,2-hexamethyldisilane (293 mg, 2.00 mmol, 10.0 equiv.). Flash column chromatography (5% EtOAc/Petroleum ether) gave **23** (34.3 mg, 0.13 mmol, 66%) as a colourless liquid. All recorded spectroscopic data matched those previously reported in the literature[8].

^1H NMR (400 MHz, Chloroform-*d*) δ 1.23 (d, *J* = 2.1 Hz, 12H), 0.20 (s, 1H), 0.10 (s, 1H), 0.10 (d, *J* = 9.0 Hz, 6H), 0.06 (d, *J* = 6.5 Hz, 9H). *See spectrum*

^{13}C NMR (101 MHz, Chloroform-*d*) δ 82.9, 82.89, 25.2, 25.0, 2.4, 2.1, -2.2, -2.3. *The carbon attached to boron could not be observed due to quadrupolar relaxation*. *See spectrum*

HRMS (ESI$^+$) calcd. for $C_{12}H_{30}BO_2Si_2^+$ [M+H]$^+$,M/Z: 273.1872, found 273.1876.

((Dimethyl((4,4,5,5-tetramethyl-1,3,2-dioxaborolan-2-yl)methyl)silyl)methyl)trimethylsilane (24)

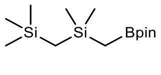

Prepared following **General Procedure A** using bis(trimethylsilyl)methane (322 mg, 2.00 mmol, 10.0 equiv.). Flash column chromatography (5% EtOAc/Petroleum ether) gave **24** (39.6 mg, 0.14 mmol, 69%, > 95:5 r.r.) as a colourless liquid. The r.r. was

determined by ^{1}H NMR analysis of the crude reaction mixture. All recorded spectroscopic data matched those previously reported in the literature[8].

^{1}H NMR (400 MHz, Chloroform-*d*) δ 1.23 (s, 12H), 0.11 (s, 2H), 0.08 (s, 6H), 0.02 (s, 9H), -0.21 (s, 2H). See spectrum

^{13}C NMR (101 MHz, Chloroform-*d*) δ 82.7, 25.0, 4.8, 1.5, 1.4. *The carbon attached to boron could not be observed due to quadrupolar relaxation*. See spectrum

HRMS (ESI$^+$) calcd. for $C_{13}H_{32}BO_2Si_2^+$ $[M+H]^+$,M/Z: 287.2028, found 287.2029.

(Chloromethyl)dimethyl((4,4,5,5-tetramethyl-1,3,2-dioxaborolan-2-yl)methyl)silane (25)

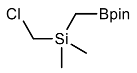

Prepared following **General Procedure A** using (chloromethyl)trimethylsilane (242 mg, 2.00 mmol, 10.0 equiv.). Flash column chromatography (5% EtOAc/Petroleum ether) gave **25** (30.3 mg, 0.12 mmol, 61%, > 95:5 r.r.) as a colourless liquid. The r.r. was determined by ^{1}H NMR analysis of the crude reaction mixture. All recorded spectroscopic data matched those previously reported in the literature[8].

^{1}H NMR (400 MHz, Chloroform-*d*) δ 2.81 (s, 2H), 1.24 (s, 12H), 0.20 (s, 2H), 0.17 (s, 6H). See spectrum

^{13}C NMR (101 MHz, Chloroform-*d*) δ 83.2, 31.7, 25.1, -2.7. *The carbon attached to boron could not be observed due to quadrupolar relaxation*. See spectrum

HRMS (ESI$^+$) calcd. for $C_{10}H_{23}BClO_2Si^+$ $[M+H]^+$,M/Z: 249.1243, found 249.1245.

(Chloromethyl)(methyl)(phenyl)((4,4,5,5-tetramethyl-1,3,2-dioxaborolan-2-yl)methyl)silane (26)

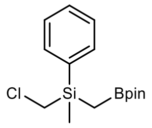

Prepared following **General Procedure A** using (chloromethyl)dimethyl(phenyl)silane (366 mg, 2.00 mmol, 10.0 equiv.). Flash column chromatography (5% EtOAc/Petroleum ether) gave **26** (31.0 mg, 0.10 mmol, 50%, > 95:5 r.r.) as a colourless liquid. The r.r. was determined by ^{1}H NMR and GC analysis of the crude reaction mixture (*See spectrum*). All recorded spectroscopic data matched those previously reported in the literature[8].

^{1}H NMR (400 MHz, Chloroform-*d*) δ 7.61 – 7.57 (m, 2H), 7.39 – 7.35 (m, 3H), 3.02 (d, *J* = 1.7 Hz, 2H), 1.17 (d, *J* = 2.5 Hz, 12H), 0.49 (s, 2H), 0.48 (s, 3H). *See spectrum*

^{13}C NMR (101 MHz, Chloroform-*d*) δ 134.0, 129.7, 127.9, 83.2, 30.6, 24.9, 24.9, -4.3. *The carbon attached to boron could not be observed due to quadrupolar relaxation. See spectrum*

HRMS (ESI^{+}) calcd. for $C_{15}H_{25}BClO_2Si^{+}$ $[M+H]^{+}$,M/Z: 311.1400, found 311.1403.

(3-Chloropropyl)dimethyl((4,4,5,5-tetramethyl-1,3,2-dioxaborolan-2-yl)methyl)silane (27)

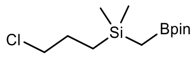

Prepared following **General Procedure A** using (3-chloropropyl)trimethylsilane (298 mg, 2.00 mmol, 10.0 equiv.). Flash column chromatography (5% EtOAc/Petroleum ether) gave **27** (34.9 mg, 0.13 mmol, 63%, > 95:5 r.r.) as a colourless liquid. The r.r. was determined by G^{1}H NMR analysis of the crude reaction mixture. All recorded spectroscopic data matched those previously reported in the literature[8].

^{1}H NMR (400 MHz, Chloroform-*d*) δ 3.49 (t, *J* = 7.0 Hz, 2H), 1.83 – 1.72 (m, 2H), 1.23 (s, 12H), 0.68 – 0.56 (m, 2H), 0.09 (s, 2H), 0.05 (s, 6H). *See spectrum*

^{13}C NMR (101 MHz, Chloroform-*d*) δ 82.9, 48.2, 27.7, 25.1, 14.9, -1.4. *The carbon attached to boron could not be observed due to quadrupolar relaxation. See spectrum*

HRMS (ESI^{+}) calcd. for $C_{12}H_{27}BClO_2Si^{+}$ $[M+H]^{+}$ 277.1556, found 277.1551.

Triethyl(1-(4,4,5,5-tetramethyl-1,3,2-dioxaborolan-2-yl)ethyl)silane (28-α) and triethyl(2- (4,4,5,5-tetramethyl-1,3,2-dioxaborolan-2-yl)ethyl)silane (28-β)

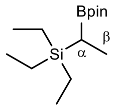

28-α

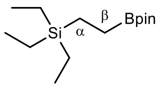

28-β

Prepared following **General Procedure A** using tetraethylsilane (286 mg, 2.00 mmol, 10.0 equiv.). Flash column chromatography (5% EtOAc/Petroleum ether) gave **28** (26.3 mg, 0.13 mmol, 45%, **28-α** : **28-β** = 12:88) as a colourless liquid. The r.r. was determined by ^{1}H NMR and GC analysis of the crude reaction mixture (*See spectrum*). All recorded spectroscopic data matched those previously reported in the literature[8].

(**28-α**) ^{1}H NMR (400 MHz, Chloroform-*d*) δ 1.22 (s, 2H), 0.97 – 0.91 (m, 7H), 0.75 – 0.68 (m, 2H), 0.60 – 0.53 (m, 3H). *See spectrum*

(**28-α**) ^{13}C NMR (101 MHz, Chloroform-*d*) δ 82.6, 25.3, 9.4, 7.7, 3.4. *The carbon attached to boron could not be observed due to quadrupolar relaxation*. *See spectrum*

(**28-β**) ^{1}H NMR (400 MHz, Chloroform-*d*) δ 1.24 (s, 12H), 1.23 – 1.21 (m, 2H), 0.94 – 0.89 (m, 9H), 0.75 – 0.68 (m, 1H), 0.60 – 0.53 (m, 1H), 0.49 (q, *J* = 7.9 Hz, 6H). *See spectrum*

(**28-β**) ^{13}C NMR (101 MHz, Chloroform-*d*) δ 83.1, 25.0, 7.6, 4.0, 3.1. *The carbon attached to boron could not be observed due to quadrupolar relaxation*. *See spectrum*

HRMS (ESI^{+}) calcd. for $C_{14}H_{32}BO_2Si^{+}$ $[M+H]^{+}$,M/Z: 271.2259, found 271.2257.

1,1,1,3,3-Pentamethyl-3-((4,4,5-trimethyl-1,3,2-dioxaborolan-2-yl)methyl)disiloxane (29)

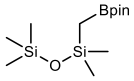

Prepared following **General Procedure B** using 1,1,1,3,3,3-hexamethyldisiloxane (322 mg, 2.00 mmol, 10.0 equiv.). Flash column chromatography (10% EtOAc/Petroleum ether) gave **29** (19.6 mg, 0.07 mmol, 34%) as a colourless liquid.

^{1}H NMR (400 MHz, Chloroform-*d*) δ 1.23 (s, 12H), 0.20 (s, 2H), 0.12 (s, 6H), 0.07 (d, *J* = 0.8 Hz, 9H). See spectrum

^{13}C NMR (101 MHz, Chloroform-*d*) δ 82.9, 25.0, 2.4, 2.1. *The carbon attached to boron could not be observed due to quadrupolar relaxation*. See spectrum

HRMS (ESI$^+$) calcd. for $C_{12}H_{30}BO_3Si_2^+$ [M+H]$^+$ 289.1821, found 289.1823.

2,2'-(2-Methylpropane-1,3-diyl)bis(4,4,5,5-tetramethyl-1,3,2-dioxaborolane) (30)

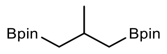

Prepared following **General Procedure A** using 2-isobutyl-4,4,5,5-tetramethyl-1,3,2-dioxaborolane (368 mg, 2.00 mmol, 10.0 equiv.). Flash column chromatography (10% EtOAc/Petroleum ether) gave **30** (28.5 mg, 0.09 mmol, 46%, > 95:5 r.r.) as a colourless liquid. The r.r. was determined by ^{1}H NMR analysis of the crude reaction mixture.

^{1}H NMR (400 MHz, Chloroform-*d*) δ 2.06 – 1.96 (m, 1H), 1.23 (s, 24H), 0.97 (d, *J* = 6.6 Hz, 3H), 0.91 – 0.84 (m, 2H), 0.80 – 0.71 (m, 2H). See spectrum

^{13}C NMR (101 MHz, Chloroform-*d*) δ 82.9, 26.3, 25.0, 24.9, 24.8. *The carbon attached to boron could not be observed due to quadrupolar relaxation*. See spectrum

HRMS (ESI$^+$) calcd. for $C_{16}H_{33}B_2O_4^+$ [M+H]$^+$,M/Z: 311.2559, found 311.2558.

2-(4-(Isopentyloxy)-2-methylbutyl)-4,4,5-trimethyl-1,3,2-dioxaborolane (31)

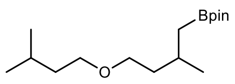

Prepared following **General Procedure B** using 1-(isopentyloxy)-3-methylbutane (140 mg, 2.00 mmol, 10.0 equiv.). Flash column chromatography (10% EtOAc/Petroleum

ether) gave **31** (16.5 mg, 0.06 mmol, 29%, > 95:5 r.r.) as a colourless liquid. The r.r. was determined by ^{1}H NMR analysis of the crude reaction mixture.

^{1}H NMR (400 MHz, Chloroform-*d*) δ 3.46 – 3.38 (m, 4H), 1.87 – 1.77 (m, 1H), 1.73 – 1.65 (m, 1H), 1.63 – 1.53 (m, 1H), 1.51 – 1.41 (m, 3H), 1.24 (s, 12H), 0.93 (d, *J* = 6.7 Hz, 3H), 0.89 (d, *J* = 6.6 Hz, 6H), 0.87 – 0.83 (m, 1H), 0.72 – 0.61 (m, 1H). See spectrum

^{13}C NMR (101 MHz, Chloroform-*d*) δ 83.0, 69.6, 69.4, 39.2, 38.8, 26.9, 25.3, 25.0, 24.9, 22.8, 22.5. *The carbon attached to boron could not be observed due to quadrupolar relaxation*. See spectrum

HRMS (ESI$^+$) calcd. for $C_{16}H_{34}BO_3^+$ [M+H]$^+$ 285.2596, found 285.2597.

4,4,5,5-Tetramethyl-2-(1-propoxypropan-2-yl)-1,3,2-dioxaborolane (32-β) and 4,4,5,5-tetramethyl-2-(3-propoxypropyl)-1,3,2-dioxaborolane (32-γ)

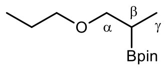

32-β

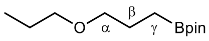

32-γ

Prepared following **General Procedure B** using 1-propoxypropane (202 mg, 2.00 mmol, 10.0 equiv.). Flash column chromatography (10% EtOAc/Petroleum ether) gave **57** (18.3 mg, 0.08 mmol, 40%, **32-β** : **32-γ** = 25:75) as a colourless liquid. The r.r. was determined by ^{1}H and ^{13}C NMR analysis of the crude reaction mixture.

^{1}H NMR (400 MHz, Chloroform-*d*) δ 3.56 – 3.50 (m, 0.33H, compound **32-β**), 3.46 – 3.35 (m, 5H containing compound **32-β**), 1.78 – 1.68 (m, 3H containing compound **32-β** and H_2O), 1.66 – 1.59 (m, 2.5H containing compound **32-β**), 1.28 (d, *J* = 1.1 Hz, 15H containing compound **32-β**), 1.04 (d, *J* = 7.4 Hz, 1H, compound **32-β**), 0.95 (t, *J* = 7.4 Hz, 4H containing compound **32-β**), 0.84 (t, *J* = 7.8 Hz, 2H). See spectrum

(**32-γ**) ^{13}C NMR (101 MHz, Chloroform-*d*) δ 83.0, 72.7, 72.5, 24.9, 24.2, 23.0, 10.7. *The carbon attached to boron could not be observed due to quadrupolar relaxation*. See spectrum

(**32-β**) ^{13}C NMR (101 MHz, Chloroform-*d*) δ 83.1, 73.8, 72.4, 24.8, 23.0, 12.5, 10.7. *The carbon attached to boron could not be observed due to quadrupolar relaxation*. <u>*See spectrum*</u>

HRMS (ESI$^+$) calcd. for $C_{12}H_{26}BO_3^+$ [M+H]$^+$ 229.1970, found 229.1971.

1-(4,4,5-Trimethyl-1,3,2-dioxaborolan-2-yl)pentan-3-one (33)

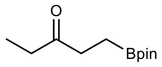

Prepared following **General Procedure A** using pentan-3-one (190 mg, 2.00 mmol, 10.0 equiv.). Flash column chromatography (5% EtOAc/Petroleum ether) gave **33** (17.1 mg, 0.08 mmol, 40%, > 95:5 r.r.) as a colourless liquid. The r.r. was determined by ^1H NMR analysis of the crude reaction mixture.

^1H NMR (400 MHz, Chloroform-*d*) δ 2.55 (t, *J* = 7.1 Hz, 2H), 2.41 (q, *J* = 7.4 Hz, 2H), 1.23 (s, 12H), 1.05 (t, *J* = 7.4 Hz, 3H), 0.91 (t, *J* = 7.1 Hz, 2H). <u>*See spectrum*</u>

^{13}C NMR (101 MHz, Chloroform-*d*) δ 212.2, 83.2, 37.1, 35.5, 24.9, 8.2. *The carbon attached to boron could not be observed due to quadrupolar relaxation*. <u>*See spectrum*</u>

HRMS (ESI$^+$) calcd. for $C_{11}H_{22}BO_3^+$ [M+H]$^+$ 213.1657, found 213.1658.

4-(4,4,5-Trimethyl-1,3,2-dioxaborolan-2-yl)butan-2-one (34)

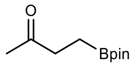

Prepared following **General Procedure B** using butan-2-one (142 mg, 2.00 mmol, 10.0 equiv.). Flash column chromatography (10% EtOAc/Petroleum ether) gave **34** (23.9 mg, 0.12 mmol, 60%, > 95:5 r.r.) as a colourless liquid. The r.r. was determined by GC analysis of the crude reaction mixture (<u>*See spectrum*</u>).

^1H NMR (400 MHz, Chloroform-*d*) δ 2.59 (t, *J* = 7.1 Hz, 2H), 2.13 (s, 3H), 1.23 (s, 12H), 0.90 (t, *J* = 7.1 Hz, 2H). <u>*See spectrum*</u>

^{13}C NMR (101 MHz, Chloroform-*d*) δ 209.4, 83.3, 38.6, 29.5, 24. 9. *The carbon attached to boron could not be observed due to quadrupolar relaxation*. See spectrum
HRMS (ESI^+) calcd. for $C_{10}H_{20}BO_3^+$ $[M+H]^+$ 199.1500, found 199.1504.

3-Methyl-4-(4,4,5-trimethyl-1,3,2-dioxaborolan-2-yl)butan-2-one (35)

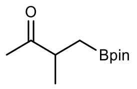

Prepared following **General Procedure B** using 3-methylbutan-2-one (190 mg, 2.00 mmol, 10.0 equiv.). Flash column chromatography (10% EtOAc/Petroleum ether) gave **35** (24.7 mg, 0.12 mmol, 58%, > 95:5 r.r.) as a colourless liquid. The r.r. was determined by GC analysis of the crude reaction mixture (*See spectrum*).

^1H NMR (400 MHz, Chloroform-*d*) δ 2.77 – 2.65 (m, 1H), 2.14 (s, 3H), 1.22 (d, *J* = 4.8 Hz, 12H), 1.14 (d, *J* = 7.1 Hz, 3H), 1.06 – 0.98 (m, 1H), 0.84 –0.76 (m, 1H). *See spectrum*

^{13}C NMR (101 MHz, Chloroform-*d*) δ 213.1, 83.3, 43.4, 27.7, 24.9, 24.9, 18.9. *The carbon attached to boron could not be observed due to quadrupolar relaxation*. *See spectrum*

HRMS (ESI^+) calcd. for $C_{11}H_{22}BO_3^+$ $[M+H]^+$ 213.1657, found 213.1658.

4-Methyl-5-(4,4,5-trimethyl-1,3,2-dioxaborolan-2-yl)pentan-2-one (36)

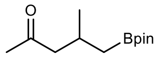

Prepared following **General Procedure B** using 4-methylpentan-2-one (198 mg, 2.00 mmol, 10.0 equiv.). Flash column chromatography (5% EtOAc/Petroleum ether) gave **36** (29.5 mg, 0.13 mmol, 65%, > 95:5 r.r.) as a colourless liquid. The r.r. was determined by GC analysis of the crude reaction mixture(*See spectrum*).

^{1}H NMR (400 MHz, Chloroform-*d*) δ 2.50 – 2.39 (m, 1H), 2.32 – 2.19 (m, 2H), 2.12 (s, 3H), 1.24 (s, 12H), 0.94 (d, *J* = 4.7 Hz, 3H), 0.85 – 0.79 (m, 1H), 0.76 – 0.68 (m, 1H). <u>*See spectrum*</u>

^{13}C NMR (101 MHz, Chloroform-*d*) δ 209.3, 83.2, 53.4, 30.5, 26.2, 25.0, 24.9, 22.6. *The carbon attached to boron could not be observed due to quadrupolar relaxation*. <u>*See spectrum*</u>

HRMS (ESI$^+$) calcd. for $C_{12}H_{24}BO_3^+$ [M+H]$^+$ 227.1813, found 227.1814.

6-Methyl-7-(4,4,5-trimethyl-1,3,2-dioxaborolan-2-yl)heptan-2-one (37)

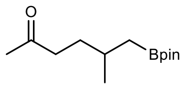

Prepared following **General Procedure B** using 5-methylhexan-2-one (226 mg, 2.00 mmol, 10.0 equiv.). Flash column chromatography (5% EtOAc/Petroleum ether) gave **37** (28.4 mg, 0.12 mmol, 58%, > 95:5 r.r.) as a colourless liquid. The r.r. was determined by ^{1}H NMR analysis of the crude reaction mixture.

^{1}H NMR (400 MHz, Chloroform-*d*) δ 2.41 (t, *J* = 7.9 Hz, 2H), 2.13 (s, 3H), 1.73 – 1.63 (m, 1H), 1.57 – 1.42 (m, 2H), 1.24 (s, 12H), 0.92 (d, *J* = 6.6 Hz, 3H), 0.85 – 0.80 (m, 1H), 0.70 – 0.62 (m, 1H). <u>*See spectrum*</u>

^{13}C NMR (101 MHz, Chloroform-*d*) δ 209.7, 83.1, 42.0, 33.4, 29.9, 29.3, 25.0, 24.9, 22.2. *The carbon attached to boron could not be observed due to quadrupolar relaxation*. <u>*See spectrum*</u>

HRMS (ESI$^+$) calcd. for $C_{13}H_{26}BO_3^+$ [M+H]$^+$ 241.1970, found 241.1970.

2,4-Dimethyl-1-(4,4,5-trimethyl-1,3,2-dioxaborolan-2-yl)pentan-3-one (38)

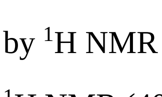

Prepared following **General Procedure B** using 2,4-dimethylpentan-3-one (226 mg, 2.00 mmol, 10.0 equiv.). Flash column chromatography (5% EtOAc/Petroleum ether) gave **38** (32.8 mg, 0.14 mmol, 68%, > 95:5 r.r.) as a colourless liquid. The r.r. was determined by GC analysis of the crude reaction mixture (see spectrum).

^{1}H NMR (400 MHz, Chloroform-*d*) δ 2.94 – 2.73 (m, 2H), 1.20 (d, *J* = 6.2 Hz, 12H), 1.11 (d, *J* = 7.1 Hz, 3H), 1.05 (dd, *J* = 6.9, 5.3 Hz, 6H), 1.02 – 0.91 (m, 1H), 0.81 – 0.74 (m, 1H). See spectrum

^{13}C NMR (101 MHz, Chloroform-*d*) δ 218.7, 83.2, 40.9, 38.5, 24.9, 24.9, 19.2, 19.0, 18.6. *The carbon attached to boron could not be observed due to quadrupolar relaxation*. See spectrum

HRMS (ESI^{+}) calcd. for $C_{13}H_{26}BO_3^{+}$ [M+H]$^{+}$ 241.1970, found 241.1975.

2,6-Dimethyl-1-(4,4,5-trimethyl-1,3,2-dioxaborolan-2-yl)heptan-4-one (39)

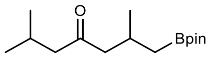

Prepared following **General Procedure B** using 2,6-dimethylheptan-4-one (282 mg, 2.00 mmol, 10.0 equiv.). Flash column chromatography (10% EtOAc/Petroleum ether) gave **39** (30.7 mg, 0.11 mmol, 57%, > 95:5 r.r.) as a colourless liquid. The r.r. was determined by GC analysis of the crude reaction mixture (see spectrum).

^{1}H NMR (400 MHz, Chloroform-*d*) δ 2.44 – 2.35 (m, 1H), 2.27 – 2.21 (m, 3H), 2.15 – 2.11 (m, 1H), 1.24 (s, 12H), 0.93 (d, *J* = 4.9 Hz, 3H), 0.90 (d, *J* = 6.6 Hz, 6H), 0.84 – 0.79 (m, 1H), 0.75 – 0.67 (m, 1H). See spectrum

^{13}C NMR (101 MHz, Chloroform-*d*) δ 211.1, 83.0, 52.8, 52.3, 25.9, 24.9, 24.9, 24.6, 22.7, 22.6, 22.5. *The carbon attached to boron could not be observed due to quadrupolar relaxation*. See spectrum

HRMS (ESI^{+}) calcd. for $C_{15}H_{30}BO_3^{+}$ [M+H]$^{+}$ 269.2283, found 269.2284.

4-(4,4,5-Trimethyl-1,3,2-dioxaborolan-2-yl)pentan-2-one (40-β) and 5-(4,4,5-trimethyl-1,3,2-dioxaborolan-2-yl)pentan-2-one (40-γ)

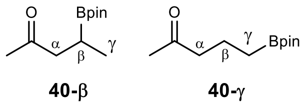

Prepared following **General Procedure B** using pentan-2-one (190 mg, 2.00 mmol, 10.0 equiv.). Flash column chromatography (10% EtOAc/Petroleum ether) gave **40** (20.9 mg, 0.10 mmol, 49%) as a colourless liquid. The r.r. was determined by ^{1}H NMR analysis of the crude reaction mixture.

(40-β) ^{1}H NMR (400 MHz, Chloroform-*d*) δ 2.55 (d, *J* = 7.0 Hz, 2H), 2.11 (s, 3H), 1.27 (q, *J* = 14.4 Hz, 1H), 1.23 (d, *J* = 3.5 Hz, 12H), 0.95 (d, *J* = 7.5 Hz, 3H). *See spectrum*

(40-β) ^{13}C NMR (101 MHz, Chloroform-*d*) δ 209.1, 83.1, 47.7, 29.8, 24.7, 24.7, 15.0. *The carbon attached to boron could not be observed due to quadrupolar relaxation*. *See spectrum*

HRMS (ESI^{+}) calcd. for $C_{11}H_{22}BO_3^{+}$ [M+H]$^{+}$ 213.1657, found 213.1658.

(40-γ) ^{1}H NMR (400 MHz, Chloroform-*d*) δ 2.42 (t, *J* = 7.5 Hz, 2H), 2.11 (s, 3H), 1.68 (p, *J* = 7.6 Hz, 2H), 1.23 (s, 12H), 0.94 (d, *J* = 7.4 Hz, 1H), 0.77 (t, *J* = 7.8 Hz, 2H). *See spectrum*

(40-γ) ^{13}C NMR (101 MHz, Chloroform-*d*) δ 209.5, 83.2, 46.2, 30.0, 25.0, 18.7. *The carbon attached to boron could not be observed due to quadrupolar relaxation*. *See spectrum*

HRMS (ESI^{+}) calcd. for $C_{11}H_{22}BO_3^{+}$ [M+H]$^{+}$ 213.1657, found 213.1659.

2-(4,4,5-Trimethyl-1,3,2-dioxaborolan-2-yl)heptan-4-one (41-β) and 1-(4,4,5-trimethyl-1,3,2-dioxaborolan-2-yl)heptan-4-one (41-γ)

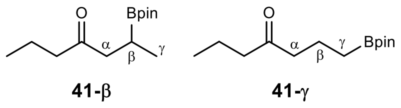

Prepared following **General Procedure B** using heptan-4-one (226 mg, 2.00 mmol, 10.0 equiv.). Flash column chromatography (10% EtOAc/Petroleum ether) gave **41** (30.8 mg, 0.13 mmol, 64%) as a colourless liquid. The r.r. was determined by ^{1}H NMR and GC analysis of the crude reaction mixture (See spectrum).

(41-β) ^{1}H NMR (400 MHz, Chloroform-*d*) δ 2.53 (d, *J* = 7.0 Hz, 2H), 2.34 (t, *J* = 7.4 Hz, 2H), 1.64 – 1.53 (m, 1H), 1.23 (d, *J* = 4.1 Hz, 12H), 0.95 (d, *J* = 7.5 Hz, 3H), 0.91 (t, *J* = 7.3 Hz, 3H), 0.88 – 0.82 (m, 1H). See spectrum

(41-β) ^{13}C NMR (101 MHz, Chloroform-*d*) δ 211.5, 83.1, 46.9, 44.6, 24.9, 24.8, 17.7, 15.2, 13.9. *The carbon attached to boron could not be observed due to quadrupolar relaxation*. See spectrum

HRMS (ESI^{+}) calcd. for $C_{13}H_{26}BO_3^{+}$ [M+H]$^{+}$ 241.1970, found 241.1971.

(41-γ) ^{1}H NMR (400 MHz, Chloroform-*d*) δ 2.42 – 2.30 (m, 4H), 1.73 – 1.64 (m, 2H), 1.62 – 1.51 (m, 2H), 1.22 (s, 12H), 0.89 (t, *J* = 7.4 Hz, 3H), 0.76 (t, *J* = 7.8 Hz, 2H). See spectrum

(41-γ) ^{13}C NMR (101 MHz, Chloroform-*d*) δ 211.6, 83.2, 45.2, 44.8, 24.9, 18.7, 17.4, 13.9. *The carbon attached to boron could not be observed due to quadrupolar relaxation*. See spectrum

HRMS (ESI^{+}) calcd. for $C_{13}H_{26}BO_3^{+}$ [M+H]$^{+}$ 241.1970, found 241.1975.

2-Methyl-1-(4,4,5,5-tetramethyl-1,3,2-dioxaborolan-2-yl)pentan-3-one (42-β) and 4-methyl-1-(4,4,5,5-tetramethyl-1,3,2-dioxaborolan-2-yl)pentan-3-one (42-β')

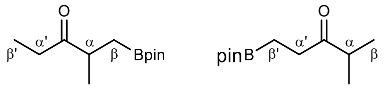

42-β **42-β'**

Prepared following **General Procedure B** using 2-methylpentan-3-one (198 mg, 2.00 mmol, 10.0 equiv.). Flash column chromatography (10% EtOAc/Petroleum ether) gave **42** (30.9 mg, 0.14 mmol, 68%, **42-β** : **42-β'** = 67:33) as a colourless liquid. The r.r. was determined by ^{1}H NMR and GC analysis of the crude reaction mixture (See spectrum).

^{1}H NMR (400 MHz, Chloroform-*d*) δ 2.78 – 2.67 (m, 1H), 2.64 – 2.54 (m, 1.5H, compound **42-β'**), 2.47 (q, *J* = 7.2 Hz, 2H), 1.22 (d, *J* = 4.8 Hz, 21H, containing compound **42-β'**), 1.12 (d, *J* = 7.1 Hz, 3H), 1.08 (d, *J* = 6.9 Hz, 3H, compound **42-β'**), 1.03 (t, *J* = 7.4 Hz, 3H), 0.94 – 0.85 (m, 2H, containing compound **42-β'**), 0.83 – 0.77 (m, 1H). <u>*See spectrum*</u>

(42-β) ^{13}C NMR (101 MHz, Chloroform-*d*) δ 215.6, 83.2, 42.3, 33.5, 24.9, 19.1, 18.6. *The carbon attached to boron could not be observed due to quadrupolar relaxation*. <u>*See spectrum*</u>

(42-β') ^{13}C NMR (101 MHz, Chloroform-*d*) δ 215.4, 83.2, 40.5, 35.3, 24.9, 8.1. *The carbon attached to boron could not be observed due to quadrupolar relaxation*. <u>*See spectrum*</u>

HRMS (ESI$^+$) calcd. for $C_{12}H_{24}BO_3^+$ [M+H]$^+$ 227.1813, found 227.1814.

Methyl 2-methyl-3-(4,4,5,5-tetramethyl-1,3,2-dioxaborolan-2-yl)propanoate (43)

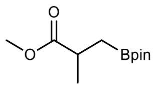

Prepared following **General Procedure B** using methyl isobutyrate (202 mg, 2.00 mmol, 10.0 equiv.). Flash column chromatography (5% EtOAc/Petroleum ether) gave **43** (19.4 mg, 0.09 mmol, 45%, > 95:5 r.r.) as a colourless liquid. The r.r. was determined by ^{1}H NMR analysis of the crude reaction mixture.

^{1}H NMR (400 MHz, Chloroform-*d*) δ 3.65 (s, 3H), 2.68 (q, *J* = 7.2 Hz, 1H), 1.24 (d, *J* = 3.5 Hz, 12H), 1.20 (d, *J* = 7.1 Hz, 3H), 1.12 (dd, *J* = 15.8, 7.5 Hz, 1H), 0.93 (dd, *J* = 15.9, 7.3 Hz, 1H). <u>*See spectrum*</u>

^{13}C NMR (101 MHz, Chloroform-*d*) δ 177.8, 83.3, 51.7, 35.5, 24.9, 19.5. *The carbon attached to boron could not be observed due to quadrupolar relaxation*. <u>*See spectrum*</u>

HRMS (ESI$^+$) calcd. for $C_{11}H_{22}BO_4^+$ [M+H]$^+$ 229.1606, found 229.1612.

Methyl 3-methyl-4-(4,4,5-trimethyl-1,3,2-dioxaborolan-2-yl)butanoate (44)

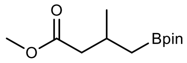

Prepared following **General Procedure B** using methyl 3-methylbutanoate (230 mg, 2.00 mmol, 10.0 equiv.). Flash column chromatography (10% EtOAc/Petroleum ether) gave **44** (32.6 mg, 0.13 mmol, 67%, > 95:5 r.r.) as a colourless liquid. The r.r. was determined by ^{1}H NMR analysis of the crude reaction mixture.

^{1}H NMR (400 MHz, Chloroform-*d*) δ 3.67 (s, 3H), 2.39 – 2.31 (m, 1H), 2.29 – 2.16 (m, 2H), 1.26 (s, 12H), 1.00 (d, *J* = 6.1 Hz, 3H), 0.93 – 0.86 (m, 1H), 0.80 – 0.72 (m, 1H). *See spectrum*

^{13}C NMR (101 MHz, Chloroform-*d*) δ 173.7, 83.1, 51.4, 43.7, 27.1, 25.0, 24.9, 22.4. *The carbon attached to boron could not be observed due to quadrupolar relaxation*. *See spectrum*

HRMS (ESI^{+}) calcd. for $C_{12}H_{24}BO_4^{+}$ [M+H]$^{+}$ 243.1762, found 243.1765.

Ethyl 3-methyl-4-(4,4,5-trimethyl-1,3,2-dioxaborolan-2-yl)butanoate (45)

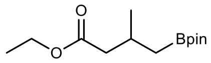

Prepared following **General Procedure B** using ethyl 3-methylbutanoate (258 mg, 2.00 mmol, 10.0 equiv.). Flash column chromatography (10% EtOAc/Petroleum ether) gave **45** (33.4 mg, 0.13 mmol, 66%, > 95:5 r.r.) as a colourless liquid. The r.r. was determined by ^{1}H NMR analysis of the crude reaction mixture.

^{1}H NMR (400 MHz, Chloroform-*d*) δ 4.10 (q, *J* = 7.1 Hz, 2H), 2.34 – 2.27 (m, 1H), 2.26 – 2.11 (m, 2H), 1.23 (s, 12H), 0.97 (d, *J* = 6.3 Hz, 3H), 0.91 – 0.83 (m, 1H), 0.77 – 0.68 (m, 1H). *See spectrum*

^{13}C NMR (101 MHz, Chloroform-*d*) δ 173.3, 83.1, 60.1, 44.0, 27.1, 25.0, 24.9, 22.3, 14.4. *The carbon attached to boron could not be observed due to quadrupolar relaxation*. *See spectrum*

HRMS (ESI^{+}) calcd. for $C_{13}H_{26}BO_4^{+}$ [M+H]$^{+}$ 257.1919, found 257.1920

Isopropyl 3-(4,4,5,5-tetramethyl-1,3,2-dioxaborolan-2-yl)propanoate (46)

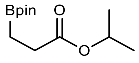

Prepared following **General Procedure B** using isopropyl propionate (230 mg, 2.00 mmol, 10.0 equiv.). Flash column chromatography (10% EtOAc/Petroleum ether) gave **46** (20.9 mg, 0.09 mmol, 43%, > 95:5 r.r.) as a colourless liquid. The r.r. was determined by ^{1}H NMR analysis of the crude reaction mixture.

^{1}H NMR (400 MHz, Chloroform-*d*) δ 4.98 (p, *J* = 6.3 Hz, 1H), 2.39 (t, *J* = 7.5 Hz, 2H), 1.23 (s, 12H), 1.21 (d, *J* = 6.3 Hz, 6H), 1.00 (t, *J* = 7.5 Hz, 2H). See spectrum

^{13}C NMR (101 MHz, Chloroform-*d*) δ 174.4, 83.3, 67.6, 29.3, 24.9, 22.0. *The carbon attached to boron could not be observed due to quadrupolar relaxation*. See spectrum

HRMS (ESI^{+}) calcd. for $C_{12}H_{24}BO_4^{+}$ [M+H]$^{+}$ 243.1762, found 243.1762.

Methyl 4-(4,4,5,5-tetramethyl-1,3,2-dioxaborolan-2-yl)butanoate (47-γ) and methyl 3-(4,4,5,5-tetramethyl-1,3,2-dioxaborolan-2-yl)butanoate (47-β)

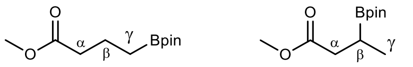

47-γ **47-β**

Prepared following **General Procedure B** using methyl butyrate (202 mg, 2.00 mmol, 10.0 equiv.). Flash column chromatography (10% EtOAc/Petroleum ether) gave **54** (24.7 mg, 0.12 mmol, 54%, **47-γ** : **47-β** = 50:50) as a colourless liquid. The r.r. was determined by ^{1}H NMR analysis of the crude reaction mixture.

(**47-γ**) ^{1}H NMR (400 MHz, Chloroform-*d*) δ 3.63 (s, 3H), 2.30 (t, *J* = 7.6 Hz, 2H), 1.72 (p, *J* = 7.8 Hz, 2H), 1.21 (d, 12H), 0.78 (t, *J* = 7.9 Hz, 2H). See spectrum

(**47-β**) ^{1}H NMR (400 MHz, Chloroform-*d*) δ 3.62 (s, 3H), 2.45 – 2.35 (m, 2H), 1.39 – 1.32 (m, 1H), 1.21 (d, 12H), 0.97 (d, *J* = 7.5 Hz, 3H). See spectrum

^{13}C NMR (101 MHz, Chloroform-*d*) δ 174.4, 174.2, 83.3, 83.2, 51.5, 37.6, 36.4, 24.9, 24.8, 24.7, 19.7, 15.2. *The carbon attached to boron could not be observed due to quadrupolar relaxation*. *See spectrum*

HRMS (ESI$^+$) calcd. for $C_{11}H_{22}BO_4^+$ $[M+H]^+$ 229.1606, found 229.1608.

Methyl 2-methyl-4-(4,4,5,5-tetramethyl-1,3,2-dioxaborolan-2-yl)butanoate (48-γ)

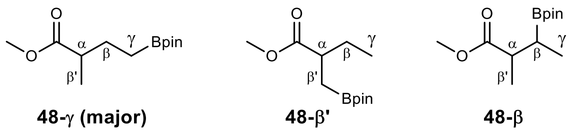

Prepared following **General Procedure B** using methyl 2-methylbutanoate (140 mg, 2.00 mmol, 10.0 equiv.). Flash column chromatography (10% EtOAc/Petroleum ether) gave **48** (19.7 mg, 0.08 mmol, 43%, **48-γ** : **48-β'** : **48-β** = 85:14:1) as a colourless liquid. The r.r. was determined by ^1H NMR and GC analysis of the crude reaction mixture (*See spectrum*).

^1H NMR (400 MHz, Chloroform-*d*) δ 3.65 (s, 3H), 2.46 – 2.37 (m, 1H), 1.80 – 1.69 (m, 1H), 1.59 – 1.47 (m, 1H), 1.23 (s, 15H), 1.13 (d, *J* = 6.9 Hz, 3H), 0.76 (t, *J* = 8.3 Hz, 2H). *See spectrum*

^{13}C NMR (101 MHz, Chloroform-*d*) δ 177.4, 83.2, 51.5, 41.5, 28.3, 24.9, 16.8. *The carbon attached to boron could not be observed due to quadrupolar relaxation*. *See spectrum*

^{13}C NMR (101 MHz, Chloroform-*d*) δ 177.4, 83.2, 51.5, 41.5, 27.4, 24.8, 22.8. *The carbon attached to boron could not be observed due to quadrupolar relaxation*. *See spectrum*

HRMS (ESI$^+$) calcd. for $C_{12}H_{24}BO_4^+$ $[M+H]^+$ 243.1762, found 243.1760.

2-Methyl-3-(4,4,5-trimethyl-1,3,2-dioxaborolan-2-yl)propyl formate (49)

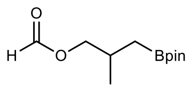

Prepared following **General Procedure B** using isobutyl formate (202 mg, 2.00 mmol, 10.0 equiv.). Flash column chromatography (10% EtOAc/Petroleum ether) gave **49** (11.9 mg, 0.05 mmol, 26%, > 95:5 r.r.) as a colourless liquid. The r.r. was determined by ^{1}H NMR analysis of the crude reaction mixture.

^{1}H NMR (400 MHz, Chloroform-*d*) δ 8.07 (s, 1H), 3.98 (d, *J* = 6.6 Hz, 2H), 2.13 – 2.04 (m, 1H), 1.24 (s, 12H), 0.98 (d, *J* = 6.7 Hz, 3H), 0.91 – 0.85 (m, 1H), 0.75 – 0.67 (m, 1H). <u>*See spectrum*</u>

^{13}C NMR (101 MHz, Chloroform-*d*) δ 161.4, 83.3, 70.4, 29.9, 25.0, 24.9, 19.3. *The carbon attached to boron could not be observed due to quadrupolar relaxation*. <u>*See spectrum*</u>

HRMS (ESI^{+}) calcd. for $C_{11}H_{22}BO_4^+$ [M+H]$^{+}$ 229.1606, found 229.1608.

2-Methyl-3-(4,4,5-trimethyl-1,3,2-dioxaborolan-2-yl)propyl acetate (50)

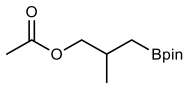

Prepared following **General Procedure B** using isobutyl acetate (230 mg, 2.00 mmol, 10.0 equiv.). Flash column chromatography (5% EtOAc/Petroleum ether) gave **50** (27.2 mg, 0.11 mmol, 56%, > 95:5 r.r.) as a colourless liquid. The r.r. was determined by ^{1}H NMR analysis of the crude reaction mixture.

^{1}H NMR (400 MHz, Chloroform-*d*) δ 3.88 – 3.76 (m, 2H), 2.04 – 1.93(m, 1H), 1.99 (s, 3H), 1.19 (s, 12H), 0.90 (d, *J* = 6.7 Hz, 3H), 0.87 – 0.79 (m, 1H), 0.66 – 0.57 (m, 1H). <u>*See spectrum*</u>

^{13}C NMR (101 MHz, Chloroform-*d*) δ 171.2, 83.1, 70.9, 29.1, 24.9, 24.8, 21.0, 19.2. *The carbon attached to boron could not be observed due to quadrupolar relaxation*. <u>*See spectrum*</u>

HRMS (ESI$^+$) calcd. for $C_{12}H_{24}BO_4^+$ [M+H]$^+$ 243.1762, found 243.1765.

3-Methyl-4-(4,4,5-trimethyl-1,3,2-dioxaborolan-2-yl)butyl acetate (51)

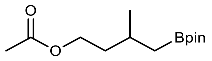

Prepared following **General Procedure B** using isopentyl acetate (238 mg, 2.00 mmol, 10.0 equiv.). Flash column chromatography (5% EtOAc/Petroleum ether) gave **51** (30.8 mg, 0.12 mmol, 60%, > 95:5 r.r.) as a colourless liquid. The r.r. was determined by ^{1}H NMR analysis of the crude reaction mixture.

^{1}H NMR (400 MHz, Chloroform-*d*) δ 4.11 – 3.98 (m, 2H), 1.99 (s, 3H), 1.84 – 1.74 (m, 1H), 1.64 – 1.54 (m, 1H), 1.51 – 1.41 (m, 1H), 1.20 (s, 12H), 0.91 (d, *J* = 6.7 Hz, 3H), 0.86 – 0.78 (m, 1H), 0.71 – 0.61 (m, 1H). See spectrum

^{13}C NMR (101 MHz, Chloroform-*d*) δ 171.3, 83.0, 63.2, 37.9, 26.6, 24.9, 24.9, 22.2, 21.1. *The carbon attached to boron could not be observed due to quadrupolar relaxation*. See spectrum

HRMS (ESI$^+$) calcd. for $C_{13}H_{26}BO_4^+$ [M+H]$^+$ 257.1919, found 257.1920.

3-(4,4,5,5-Tetramethyl-1,3,2-dioxaborolan-2-yl)propyl propionate (52-γ) and propyl 3-(4,4,5,5-tetramethyl-1,3,2-dioxaborolan-2-yl)propanoate (52-β')

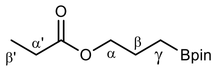

52-γ (major)

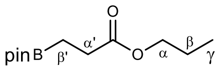

52-β' (minor)

Prepared following **General Procedure B** using propyl propionate (230 mg, 2.00 mmol, 10.0 equiv.). Flash column chromatography (10% EtOAc/Petroleum ether) gave **52** (23.8 mg, 0.11 mmol, 52%, **52-γ** : **52-β'** = 77:23) as a colourless liquid. The r.r. was determined by GC analysis of the crude reaction mixture (See spectrum).

(**52-γ**) ^{1}H NMR (400 MHz, Chloroform-*d*) δ 4.03 (t, *J* = 6.8 Hz, 2H), 2.30 (q, *J* = 7.6 Hz, 2H), 1.77 – 1.69 (m, 2H), 1.24 (s, 12H), 1.12 (t, *J* = 7.6 Hz, 3H), 0.81 (t, *J* = 8.0 Hz, 2H). *See spectrum*

(**52-γ**) ^{13}C NMR (101 MHz, Chloroform-*d*) δ 174.7 , 83.3, 66.1, 27.7, 24.9, 23.3, 9.3. *The carbon attached to boron could not be observed due to quadrupolar relaxation*. *See spectrum*

(**52-β'**) ^{1}H NMR (400 MHz, Chloroform-*d*) δ 4.01 (t, *J* = 6.8 Hz, 0.6H), 2.43 (t, *J* = 7.5 Hz, 0.6H), 1.66 – 1.60 (m, 0.6H), 1.21 (d, *J* = 3.6 Hz, 3.6H), 1.01(t, *J* = 7.5 Hz, 0.6H), 0.92 (t, *J* = 6.8 Hz, 0.9H), *See spectrum*

(**52-β'**) ^{13}C NMR (101 MHz, Chloroform-*d*) δ 174.7, 83.0, 66.0, 28.9, 24.9, 24.6, 22.2, 10.5. *The carbon attached to boron could not be observed due to quadrupolar relaxation*. *See spectrum*

HRMS (ESI$^+$) calcd. for $C_{12}H_{24}BO_4^+$ [M+H]$^+$ 243.1762, found 243.1765.

2-Methyl-3-(4,4,5,5-tetramethyl-1,3,2-dioxaborolan-2-yl)propyl propionate (53-γ) and isobutyl 3-(4,4,5,5-tetramethyl-1,3,2-dioxaborolan-2-yl)propanoate (53-β')

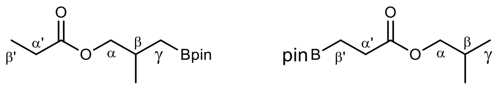

53-γ (major) **53-β' (minor)**

Prepared following **General Procedure B** using isobutyl propionate (238 mg, 2.00 mmol, 10.0 equiv.). Flash column chromatography (10% EtOAc/Petroleum ether) gave **53** (26.3 mg, 0.10 mmol, 51%, **53-γ** : **53-β'** = 91:9) as a colourless liquid. The r.r. was determined by ^{1}H NMR analysis of the crude reaction mixture.

^{1}H NMR (400 MHz, Chloroform-*d*) δ 3.95 – 3.81 (m, 2H), 2.32 (q, *J* = 7.8 Hz, 2H), 2.10 – 1.98 (m, 1H), 1.24 (s, 12H), 1.13 (t, *J* = 7.6 Hz, 3H), 0.96 (d, *J* = 6.7, 3H), 0.93 – 0.87 (m, 1H), 0.72 – 0.63 (m, 1H). *See spectrum*

^{13}C NMR (101 MHz, Chloroform-*d*) δ 174.7, 83.2, 70.8, 29.2, 27.8, 25.0, 24.9, 19.3, 9.3. *The carbon attached to boron could not be observed due to quadrupolar relaxation*. *See spectrum*

HRMS (ESI$^+$) calcd. for $C_{13}H_{26}BO_4^+$ [M+H]$^+$ 257.1919, found 257.1915.

2-Methyl-3-(4,4,5,5-tetramethyl-1,3,2-dioxaborolan-2-yl)propyl isobutyrate (54-γ) and isobutyl 2-methyl-3-(4,4,5,5-tetramethyl-1,3,2-dioxaborolan-2-yl)propanoate (54-β')

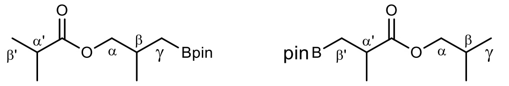

54-γ (major) **54-β' (minor)**

Prepared following **General Procedure B** using isobutyl isobutyrate (286 mg, 2.00 mmol, 10.0 equiv.). Flash column chromatography (10% EtOAc/Petroleum ether) gave **54** (19.3 mg, 0.07 mmol, 34%, **54-γ** : **54-β'** = 75:25) as a colourless liquid. The r.r. was determined by GC analysis of the crude reaction mixture (See spectrum).

(**54-γ, 54-β'**) ^{1}H NMR (400 MHz, Chloroform-*d*) δ 3.94 – 3.81 (m, 2.6H, containing compound **54-β'**), 2.68 (q, *J* = 14.4 Hz, 0.29H, compound **54-β'**) 2.60 – 2.48 (m, 1H), 2.10 – 1.98 (m, 1H), 1.96 – 1.88 (m, 0.3H, compound **54-β'**), 1.24 (s, 12H), 1.20 (d, *J* = 7.1 Hz, 1H, compound **54-β'**), 1.16 (d, *J* = 7.0 Hz, 6H), 0.96 (d, *J* = 6.7 Hz, 3H), 0.92 (d, *J* = 6.7 Hz, 2H, compound **54-β'**), 0.92 – 0.85 (m, 1H), 0.72 – 0.64 (m, 1H). See spectrum

(**54-γ**) ^{13}C NMR (101 MHz, Chloroform-*d*) δ 177.4, 83.2, 70.7, 34.3, 29.3, 25.0, 24.9, 19.3, 19.2. *The carbon attached to boron could not be observed due to quadrupolar relaxation*. See spectrum

(**54-β'**) ^{13}C NMR (101 MHz, Chloroform-*d*) δ 177.5, 83.3, 70.5, 35.7, 29.9, 24.9, 24.9, 19.6. *The carbon attached to boron could not be observed due to quadrupolar relaxation*. See spectrum

HRMS (ESI$^+$) calcd. for $C_{15}H_{30}BO_4^+$ [M+H]$^+$ 285.2232, found 285.2234.

3-methyl-4-(4,4,5,5-tetramethyl-1,3,2-dioxaborolan-2-yl)butyl benzoate (55)

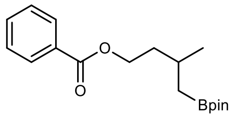

Prepared following **General Procedure B** using isopentyl benzoate (384 mg, 2.00 mmol, 10.0 equiv.). Flash column chromatography (10% EtOAc/Petroleum ether) gave **55** (34.4 mg, 0.12 mmol, 54%) as a colourless liquid. The r.r. was determined by ^{1}H NMR analysis of the crude reaction mixture.

^{1}H NMR (400 MHz, Chloroform-*d*) δ 8.07 – 8.02 (m, 2H), 7.57 – 7.52 (m, 1H), 7.43 (dd, *J* = 8.4, 7.0 Hz, 2H), 4.19 – 4.08 (m, 2H), 2.21 (dp, *J* = 8.9, 6.3 Hz, 1H), 1.25 (s, 12H), 1.06 (d, *J* = 6.7 Hz, 3H), 1.01 (dd, *J* = 15.7, 8.9 Hz, 1H), 0.78 (dd, *J* = 15.7, 8.9 Hz, 1H). See spectrum

^{13}C NMR (101 MHz, Chloroform-*d*) δ 166.8, 132.9, 130.7, 129.7, 128.4, 71.4, 29.4, 25.0, 24.9, 19.4. *The carbon attached to boron could not be observed due to quadrupolar relaxation.* See spectrum

HRMS (ESI^{+}) calcd. for $C_{18}H_{28}BO_4^{+}$ [M+H]$^{+}$ 319.2075, found 319.2078.

2-methyl-3-(4,4,5,5-tetramethyl-1,3,2-dioxaborolan-2-yl)propyl benzoate (56)

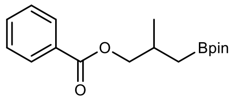

Prepared following **General Procedure B** using isobutyl benzoate (356 mg, 2.00 mmol, 10.0 equiv.). Flash column chromatography (10% EtOAc/Petroleum ether) gave **56** (35.3 mg, 0.12 mmol, 58%) as a colourless liquid. The r.r. was determined by ^{1}H NMR analysis of the crude reaction mixture.

^{1}H NMR (400 MHz, Chloroform-*d*) δ 8.08 – 8.02 (m, 2H), 7.56 – 7.52 (m, 1H), 7.43 (dd, *J* = 8.4, 7.0 Hz, 2H), 4.20 – 4.08 (m, 2H), 2.21 (dp, *J* = 8.9, 6.3 Hz, 1H), 1.25 (s, 12H), 1.06 (d, *J* = 6.7 Hz, 3H), 1.02 – 0.97 (m, 1H), 0.82 – 0.74 (m, 1H). See spectrum

^{13}C NMR (101 MHz, Chloroform-*d*) δ 166.9, 132.9, 130.7, 129.7, 128.4, 83.3, 71.4, 29.4, 25.0, 24.9, 19.4. *The carbon attached to boron could not be observed due to quadrupolar relaxation.* See spectrum

HRMS (ESI^{+}) calcd. for $C_{17}H_{26}BO_4^{+}$ [M+H]$^{+}$ 305.1919, found 305.1918.

phenyl 3-methyl-4-(4,4,5,5-tetramethyl-1,3,2-dioxaborolan-2-yl)butanoate (57)

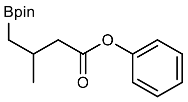

Prepared following **General Procedure B** using phenyl 3-methylbutanoate (356 mg, 2.00 mmol, 10.0 equiv.). Flash column chromatography (10% EtOAc/Petroleum ether) gave **57** (37.1 mg, 0.12 mmol, 61%) as a colourless liquid. The r.r. was determined by ^{1}H NMR analysis of the crude reaction mixture.

^{1}H NMR (400 MHz, Chloroform-*d*) δ 7.37 (t, *J* = 7.8 Hz, 2H), 7.24 – 7.18 (m, 1H), 7.08 (d, *J* = 7.6 Hz, 2H), 2.59 (dd, *J* = 14.2, 5.7 Hz, 1H), 2.47 – 2.30 (m, 2H), 1.25 (s, 12H), 1.10 (d, *J* = 6.4 Hz, 3H), 0.99 (dd, *J* = 15.8, 5.5 Hz, 1H), 0.86 (dd, *J* = 15.8, 5.5 Hz, 1H). *See spectrum*

^{13}C NMR (101 MHz, Chloroform-*d*) δ 171.7, 150.9, 129.5, 125.8, 121.9, 83.2, 43.8, 27.3, 25.0, 24.9, 22.3. *The carbon attached to boron could not be observed due to quadrupolar relaxation*. *See spectrum*

HRMS (ESI$^+$) calcd. for $C_{17}H_{26}BO_4^+$ $[M+H]^+$ 305.1919, found 305.1921.

5-(4,4,5,5-Tetramethyl-1,3,2-dioxaborolan-2-yl)pentanenitrile (58-δ) and 4-(4,4,5,5-tetramethyl-1,3,2-dioxaborolan-2-yl)pentanenitrile (58-γ)

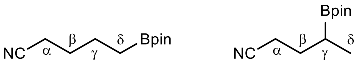

58-δ **58-γ**

Prepared following **General Procedure B** using pentanenitrile (136 mg, 2.00 mmol, 10.0 equiv.). Flash column chromatography (10% EtOAc/Petroleum ether) gave **58** (15.2 mg, 0.08 mmol, 42%, **58-δ** : **58-γ** = 50:50) as a colourless liquid. The r.r. was determined by GC analysis of the crude reaction mixture (*See spectrum*). Only the δ-substituted product (**58-δ**) is listed for characterisation and all recorded spectroscopic data matched those previously reported in the literature[8].

^{1}H NMR (400 MHz, Chloroform-*d*) δ 2.32 (t, *J* = 7.1 Hz, 2H), 1.71 – 1.61 (m, 2H), 1.60 – 1.50 (m, 2H), 1.24 (s, 12H), 0.79 (t, *J* = 7.4 Hz, 2H). *See spectrum*

^{13}C NMR (101 MHz, Chloroform-*d*) δ 120.0, 83.3, 27.9, 25.0, 23.4, 17.1. *The carbon attached to boron could not be observed due to quadrupolar relaxation*. See spectrum

HRMS (ESI$^+$) calcd. for $C_{11}H_{21}BNO_2^+$ $[M+H]^+$ 210.1660, found 210.1663.

2-(2,6,10,15,19,23-hexamethyltetracosyl)-4,4,5,5-tetramethyl-1,3,2-dioxaborolane (59)

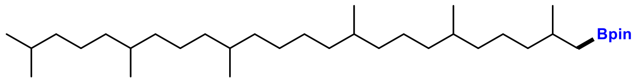

Prepared following **General Procedure B** using squalane (845 mg, 2.00 mmol, 10.0 equiv.). Flash column chromatography (10% EtOAc/Petroleum ether) gave **59** (47.3 mg, 0.086 mmol, 43%) as a colourless liquid. The r.r. was determined by ^1H NMR analysis of the crude reaction mixture.

^1H NMR (400 MHz, Chloroform-*d*) δ 1.63 – 1.48 (m, 3H), 1.45 – 1.32 (m, 6H), 1.32 – 1.26 (m, 9H), 1.24 (s, 31H), 1.19 – 0.97 (m, 7H), 0.92 – 0.81 (m, 15H), 0.76 (t, *J* = 7.8 Hz, 3H). See spectrum

^{13}C NMR (101 MHz, Chloroform-*d*) δ 82.9, 39.4, 37.3, 34.8, 32.8, 28.0, 27.5, 25.9, 24.5, 23.7, 22.8, 22.7, 22.5, 19.8, 19.8, 14.1. *The carbon attached to boron could not be observed due to quadrupolar relaxation*. See spectrum

HRMS (ESI$^+$) calcd. for $C_{36}H_{74}BO_2^+$ $[M+H]^+$ 549.5776, found 549.5777.

4. NMR Spectra

^{1}H NMR (400 MHz, $CDCl_3$) of **3** (see procedure):

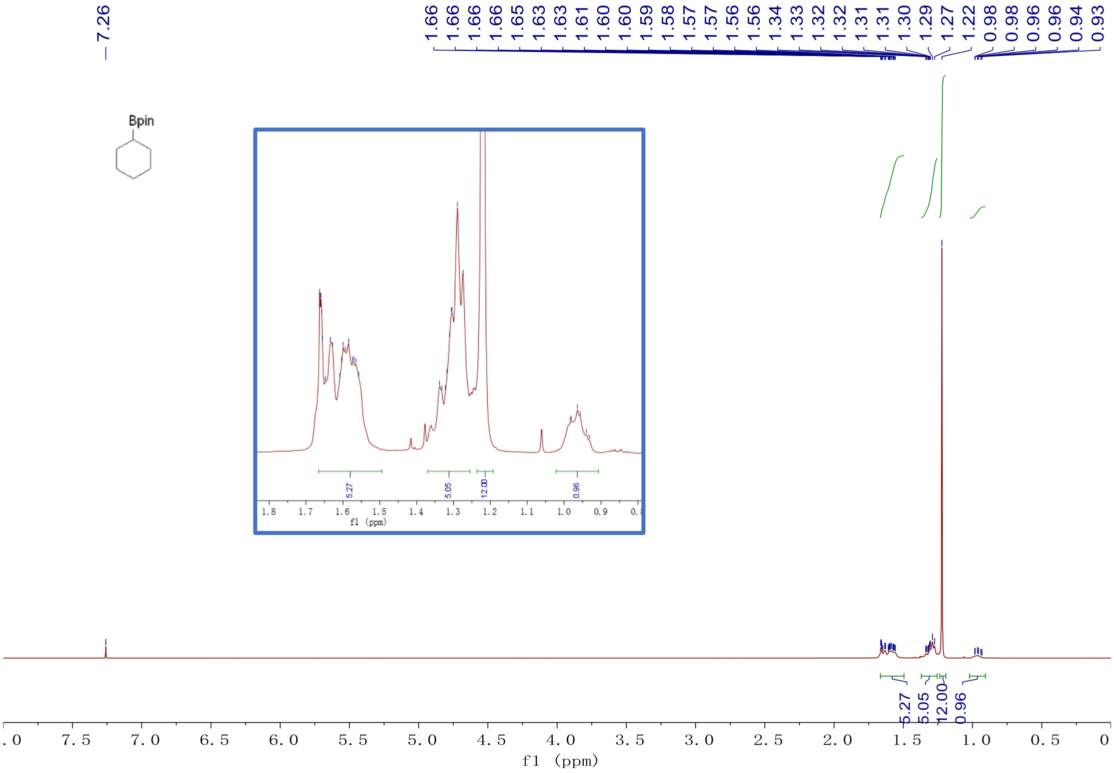

^{13}C NMR (101 MHz, $CDCl_3$) of **3** (see procedure):

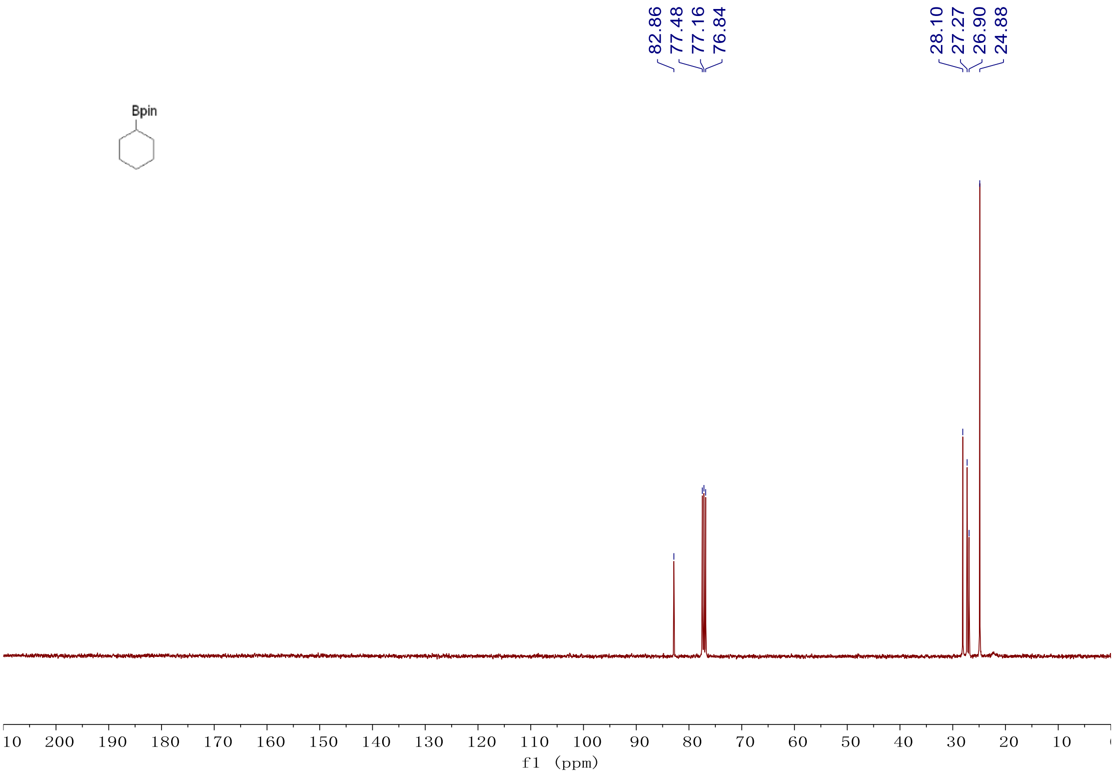

^{1}H NMR (400 MHz, $CDCl_3$) of **4** (see procedure):

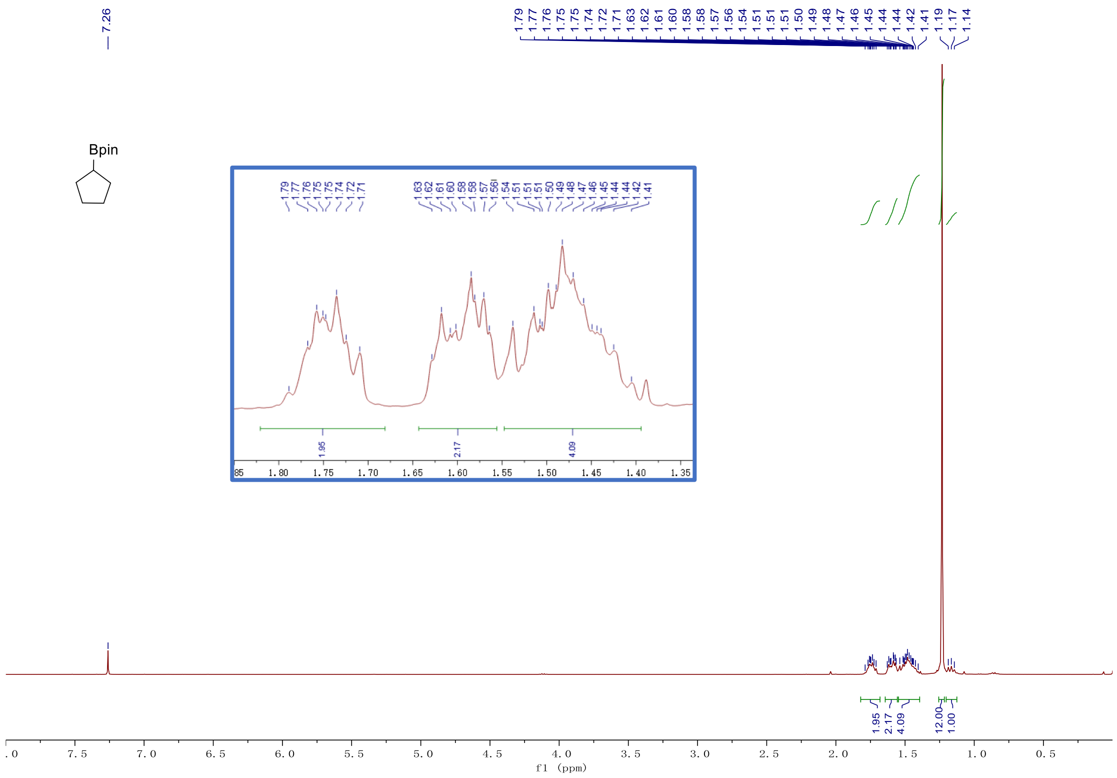

^{13}C NMR (101 MHz, $CDCl_3$) of **4** (see procedure):

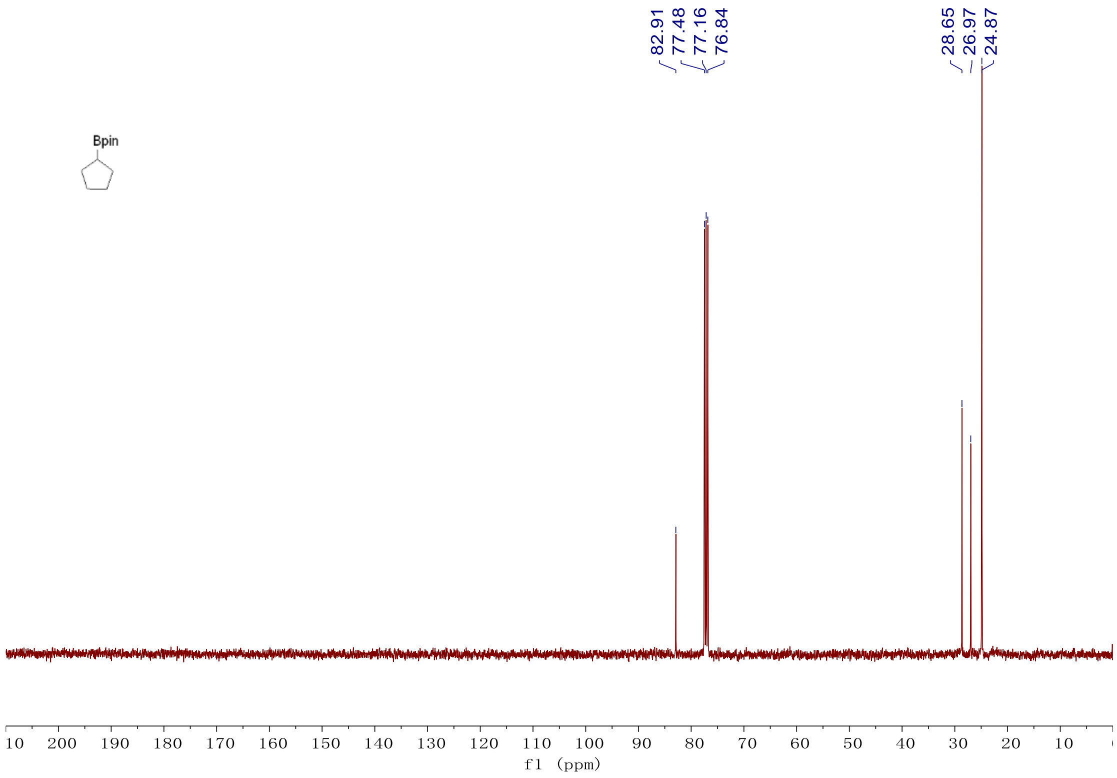

^{1}H NMR (400 MHz, $CDCl_3$) of **5** (*see procedure*):

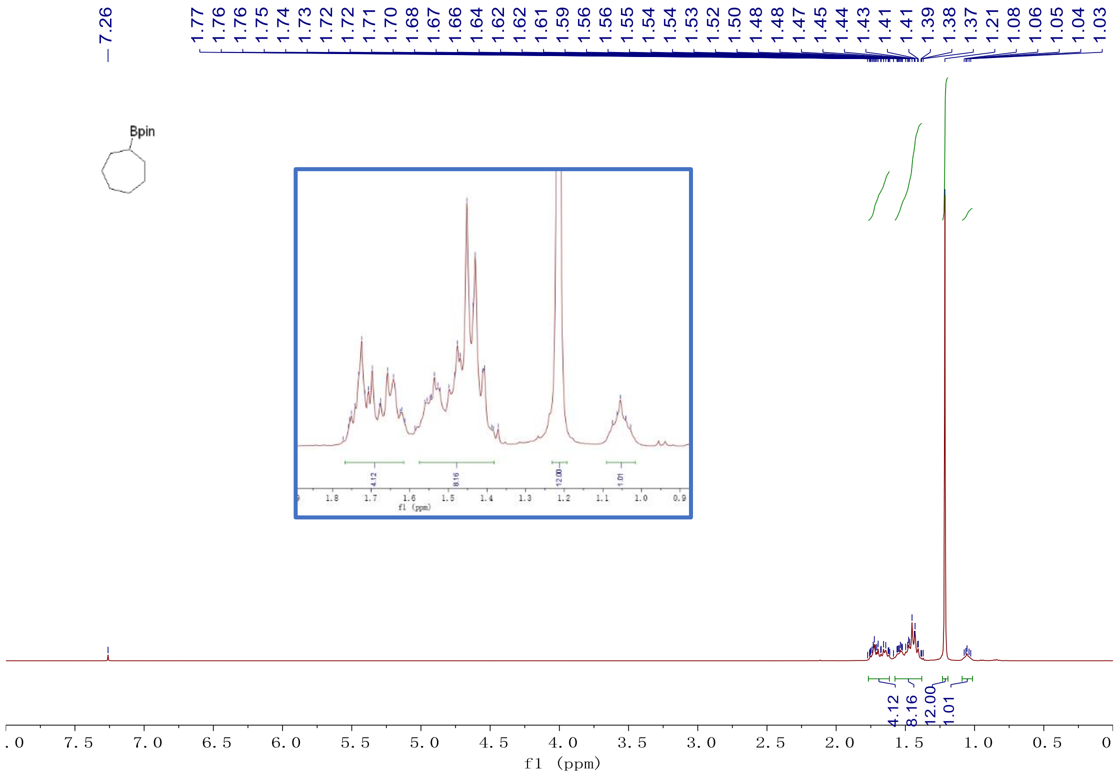

^{13}C NMR (101 MHz, $CDCl_3$) of **5** (*see procedure*):

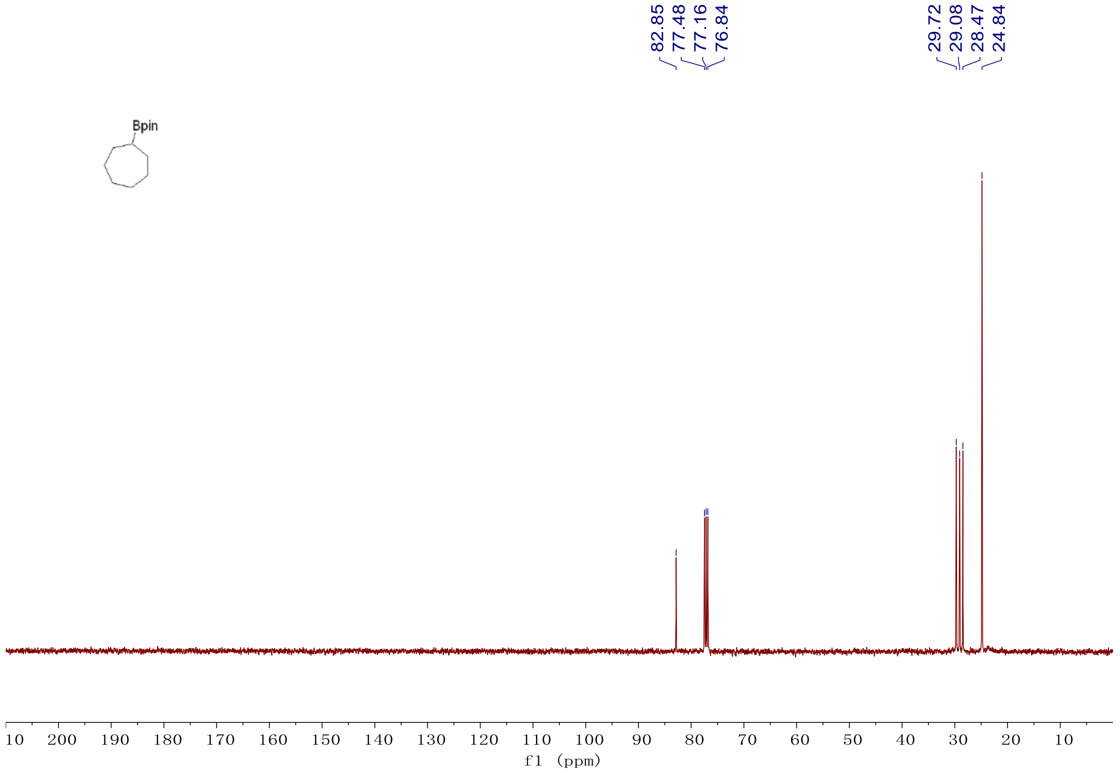

^{1}H NMR (400 MHz, $CDCl_3$) of **6** (see procedure):

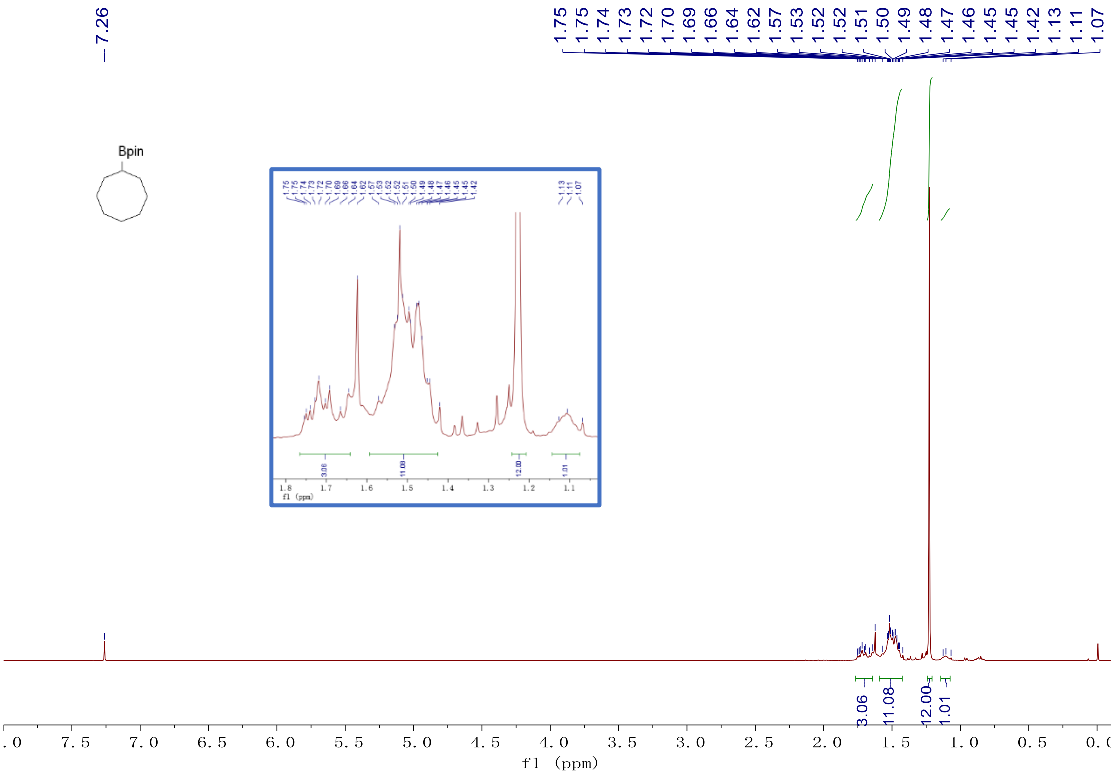

^{13}C NMR (101 MHz, $CDCl_3$) of **6** (see procedure):

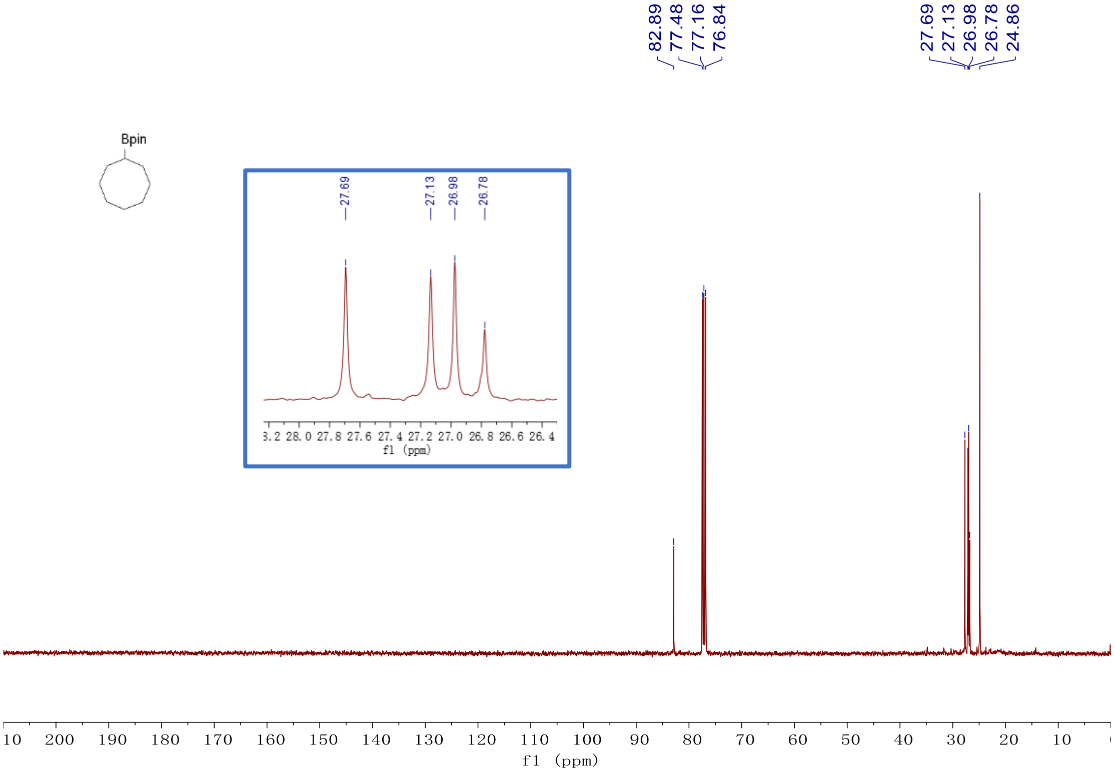

^{1}H NMR (400 MHz, $CDCl_3$) of **7** (*see procedure*):

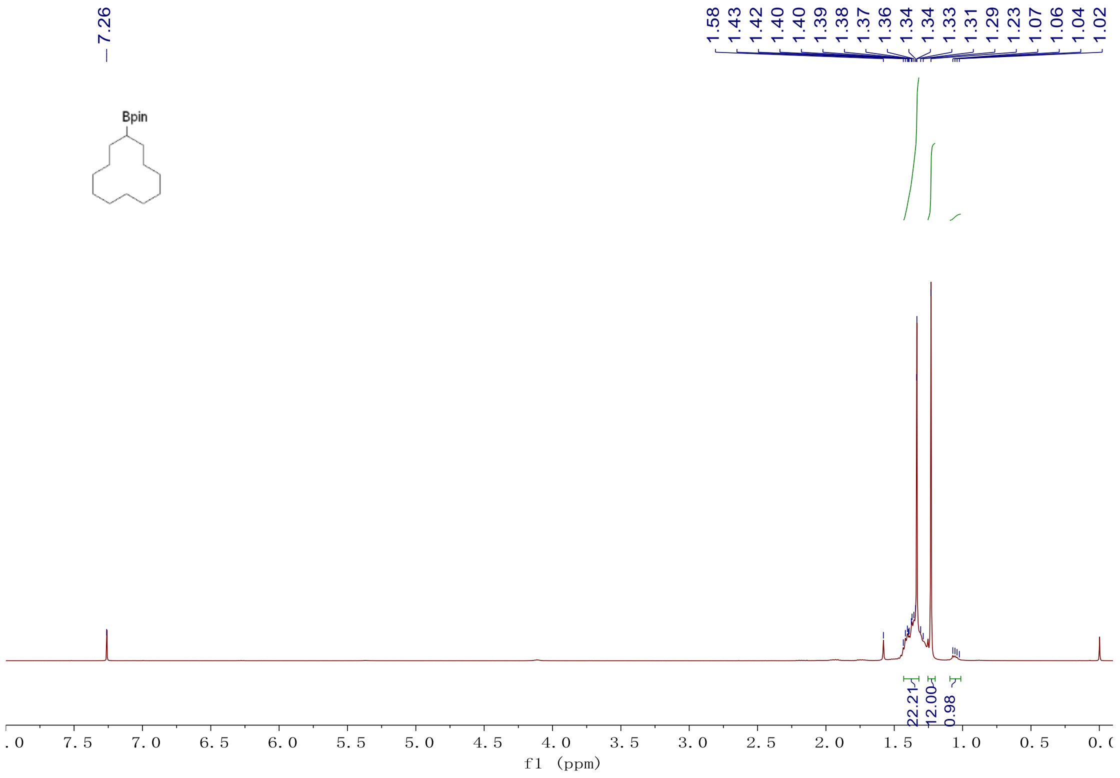

^{13}C NMR (101 MHz, $CDCl_3$) of **7** (*see procedure*):

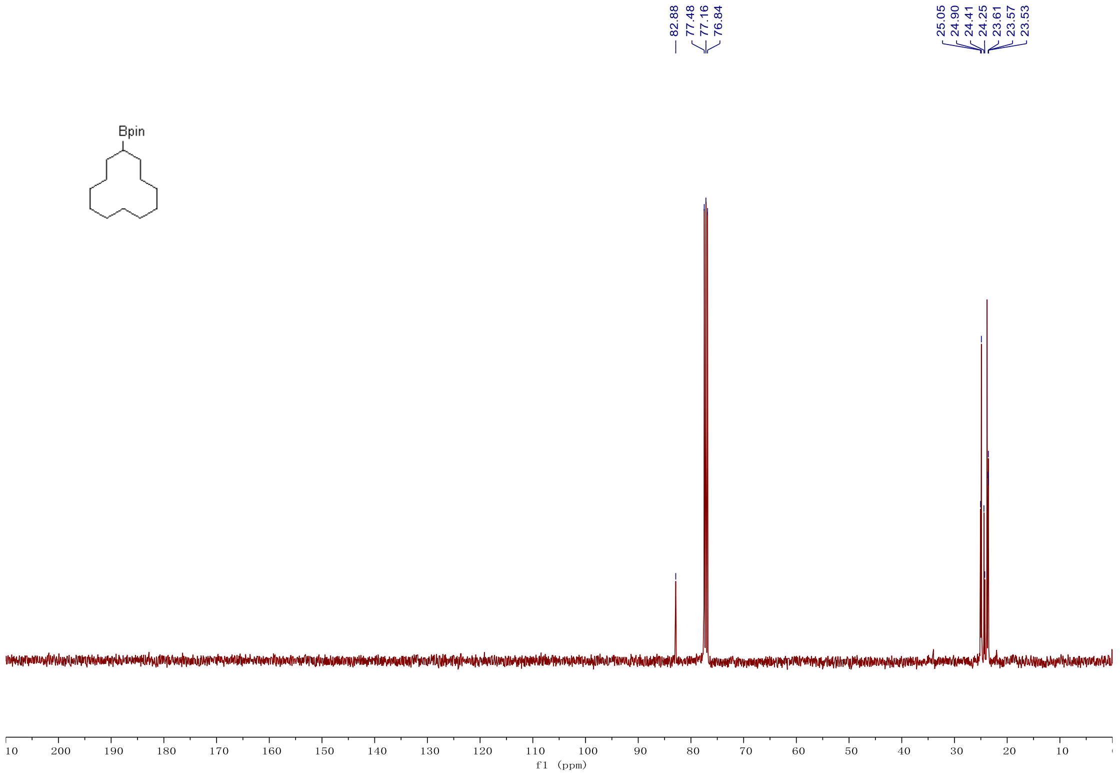

^{1}H NMR (400 MHz, $CDCl_3$) of **8** (see procedure):

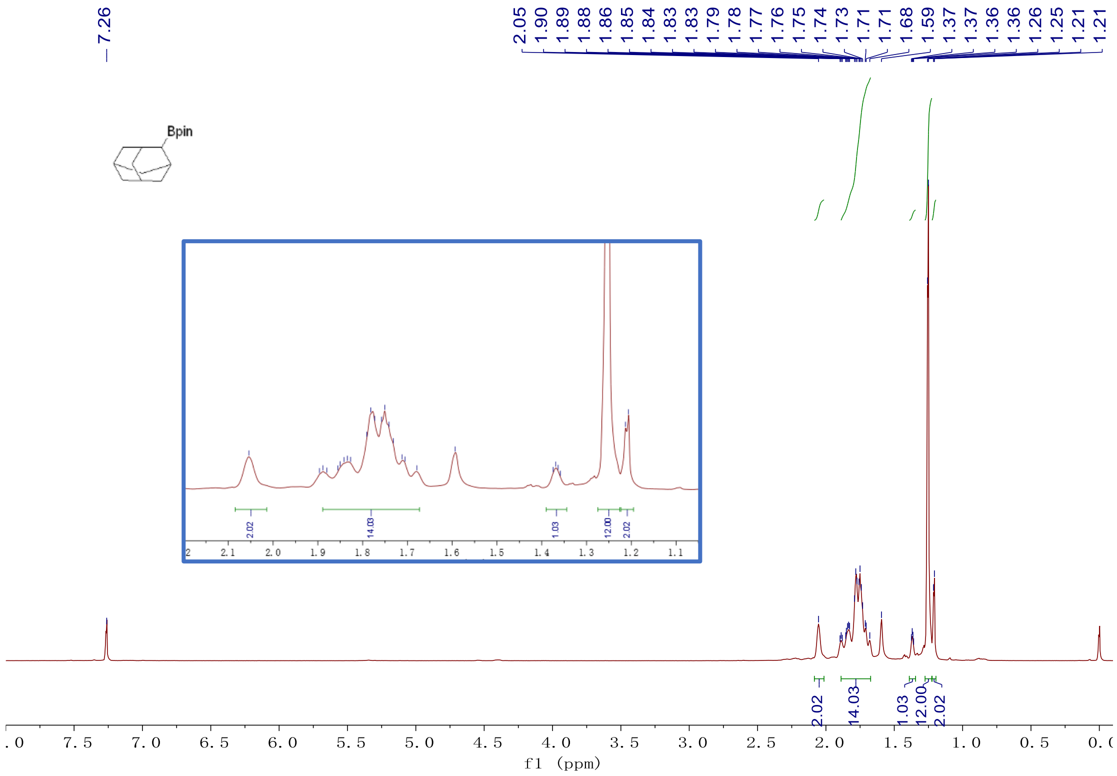

^{13}C NMR (101 MHz, $CDCl_3$) of **8** (see procedure):

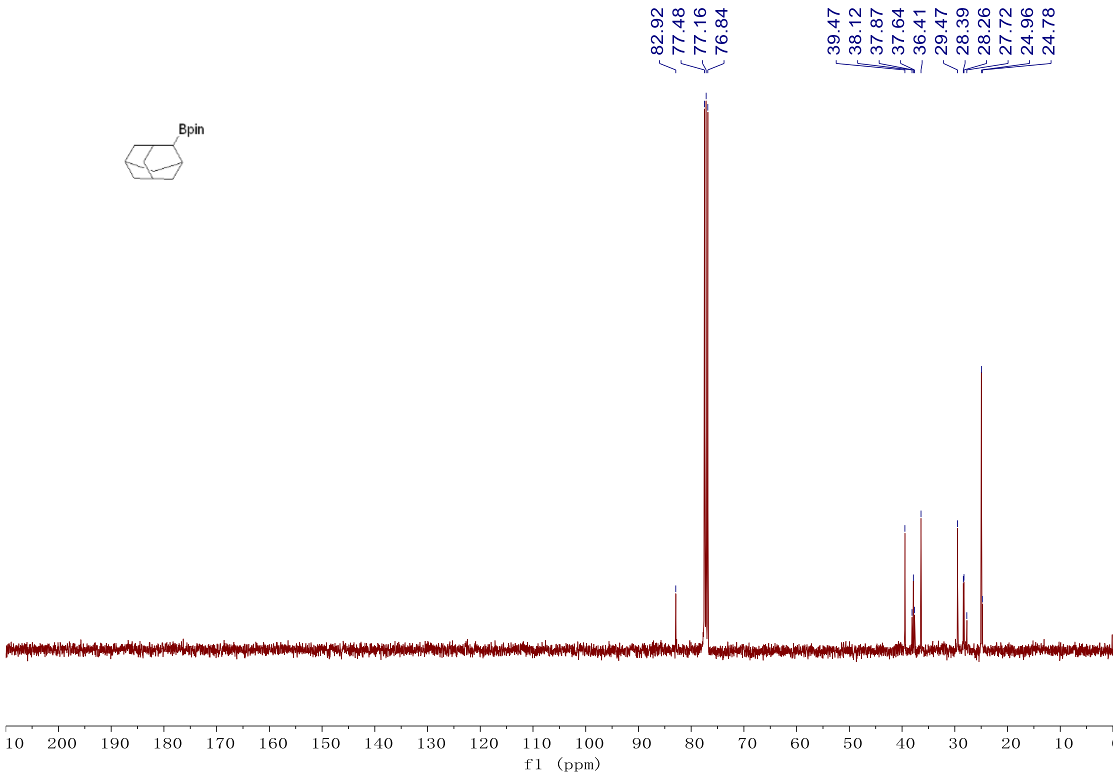

^{1}H NMR (400 MHz, $CDCl_3$) of **9** (*see procedure*):

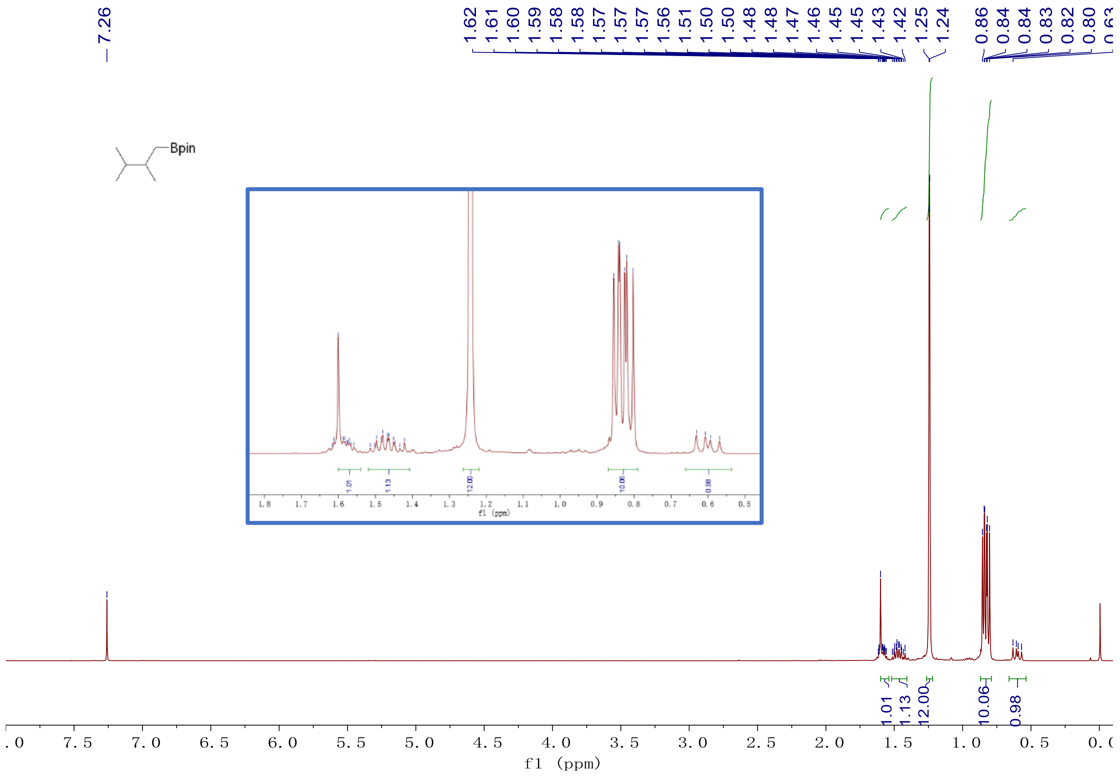

^{13}C NMR (101 MHz, $CDCl_3$) of **9** (*see procedure*):

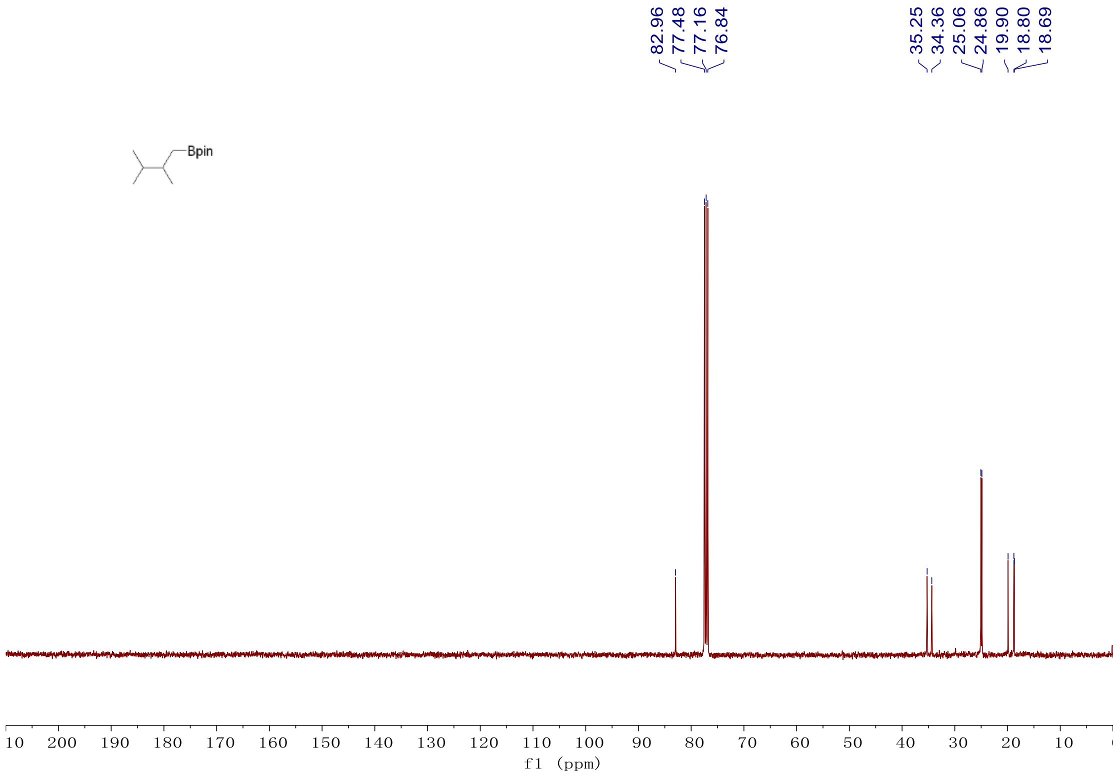

^{1}H NMR (400 MHz, $CDCl_3$) of **10** (see procedure):

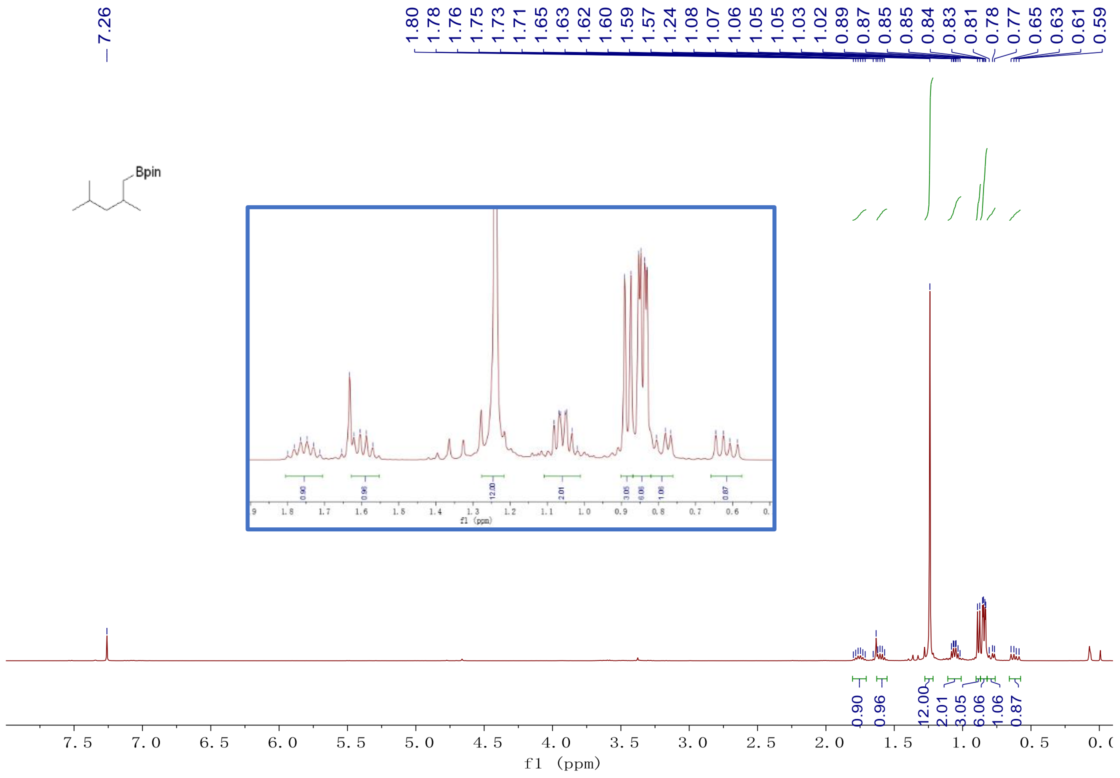

^{13}C NMR (101 MHz, $CDCl_3$) of **10** (see procedure):

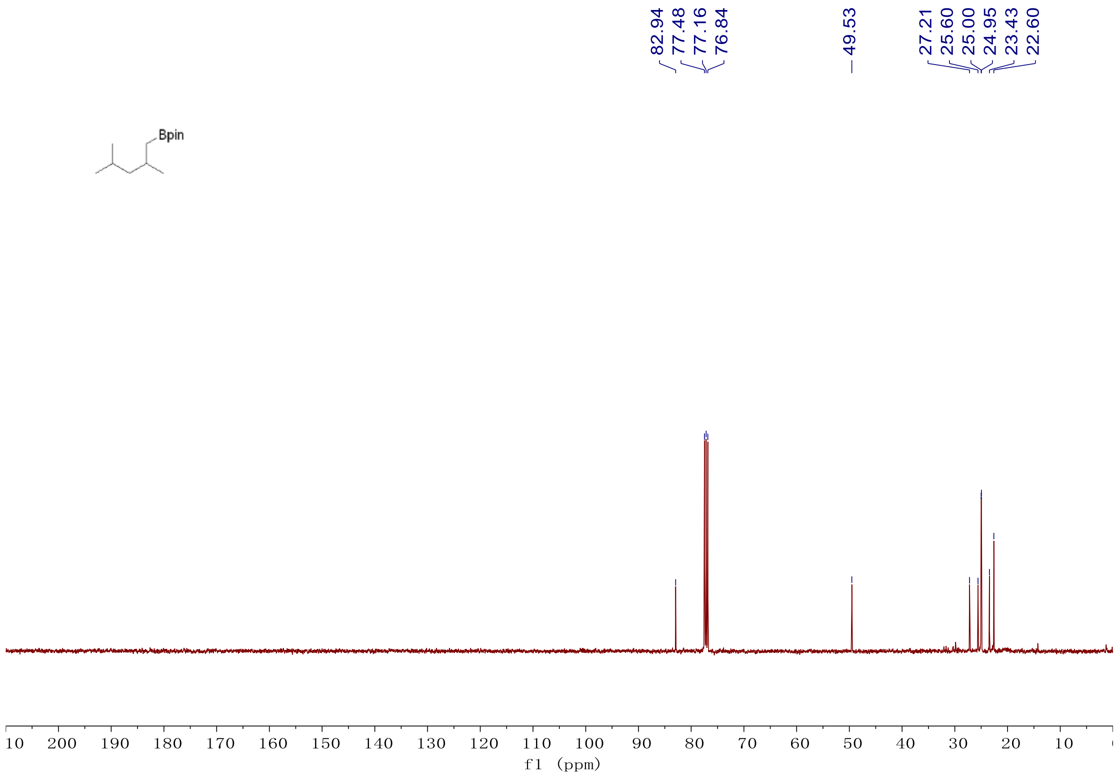

^{1}H NMR (400 MHz, $CDCl_3$) of **11** (*see procedure*):

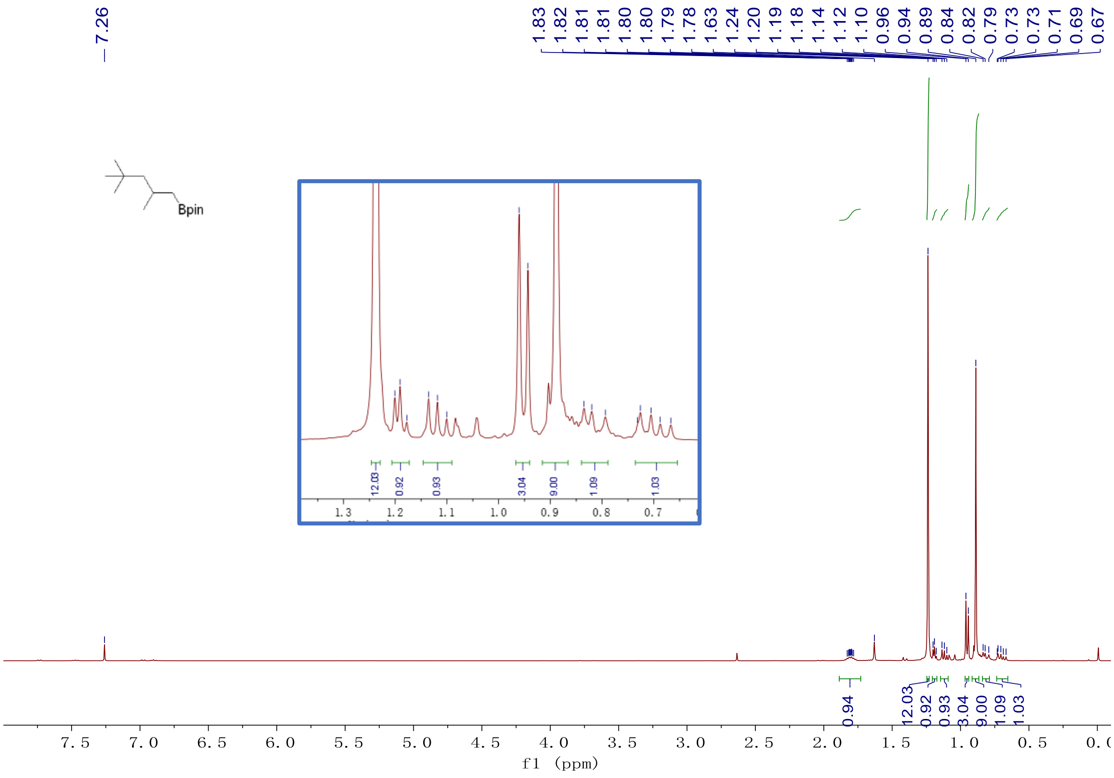

^{13}C NMR (101 MHz, $CDCl_3$) of **11** (*see procedure*):

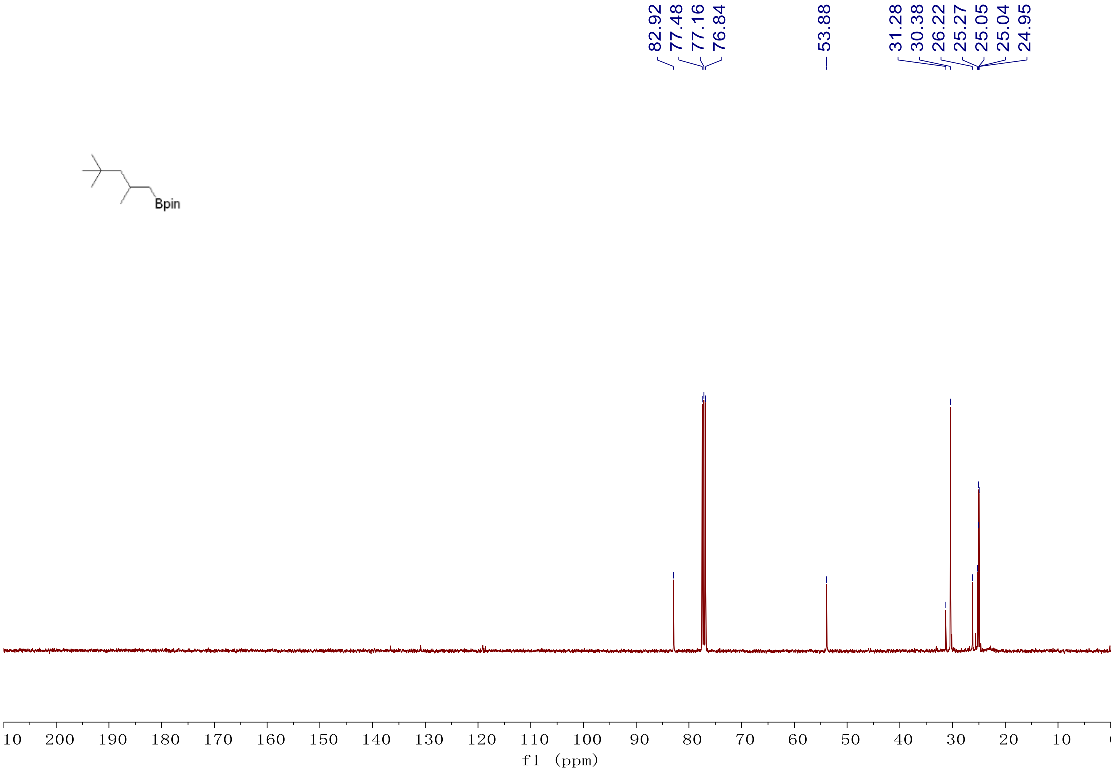

^{1}H NMR (400 MHz, $CDCl_3$) of **12** (*see procedure*):

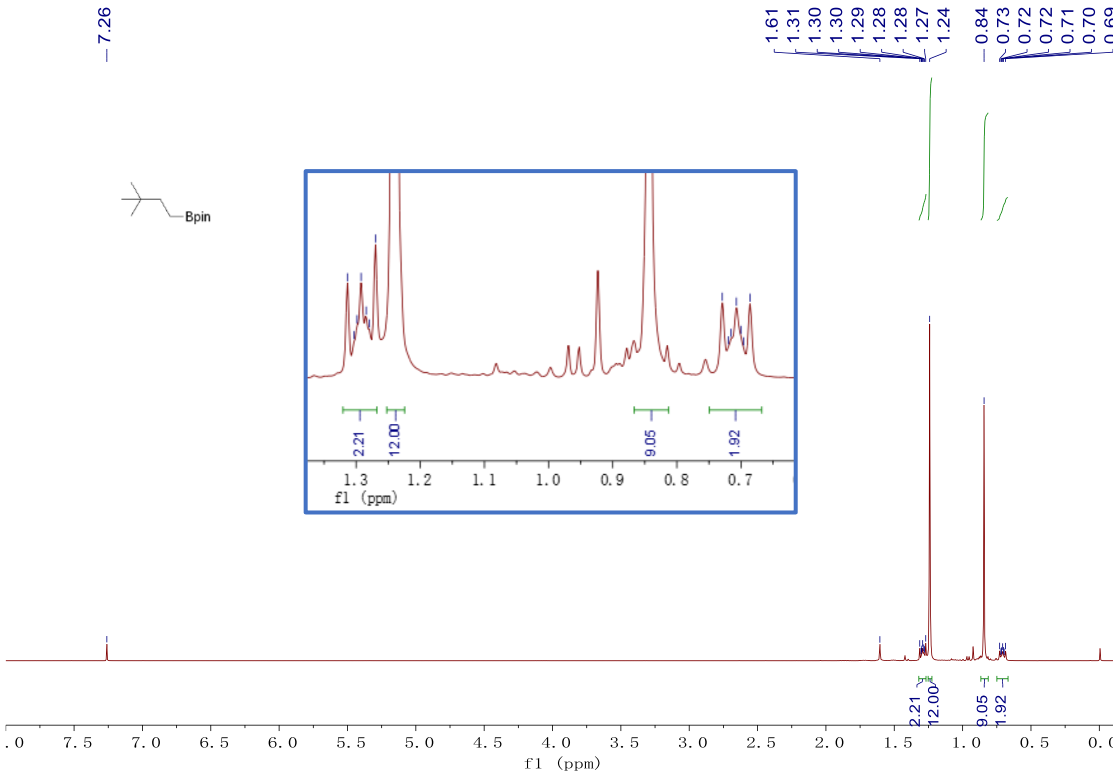

^{13}C NMR (101 MHz, $CDCl_3$) of **12** (*see procedure*):

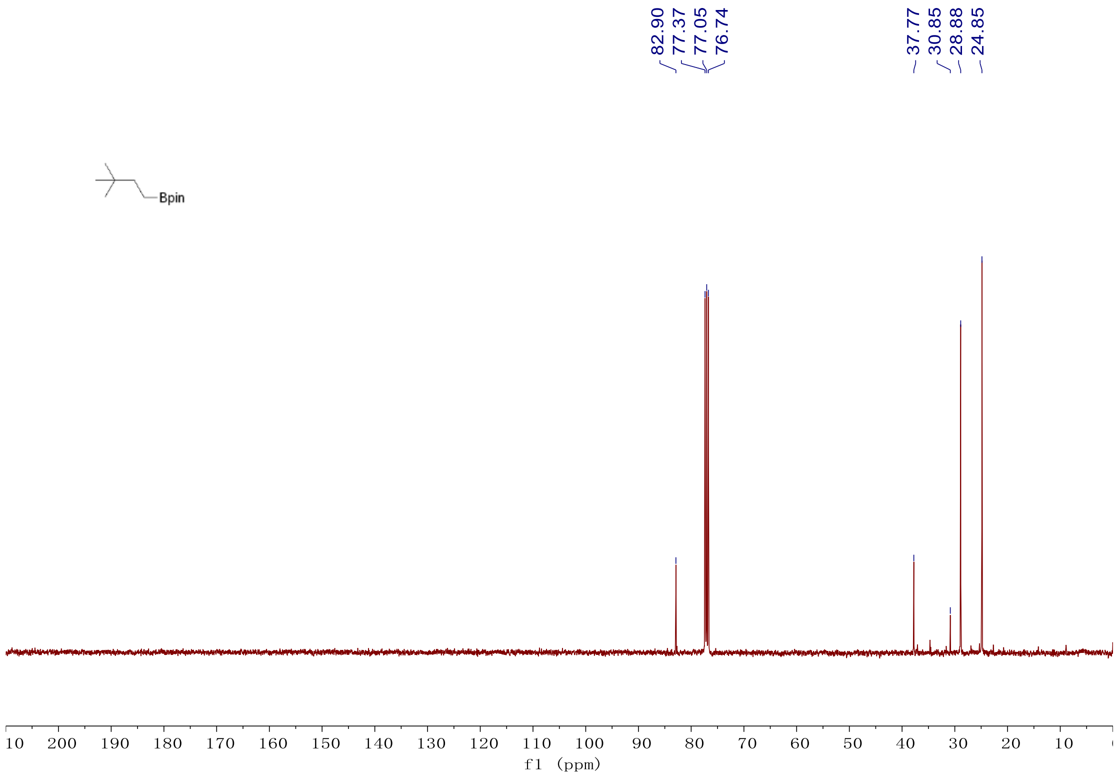

^{1}H NMR (400 MHz, $CDCl_3$) of **13** (*see procedure*):

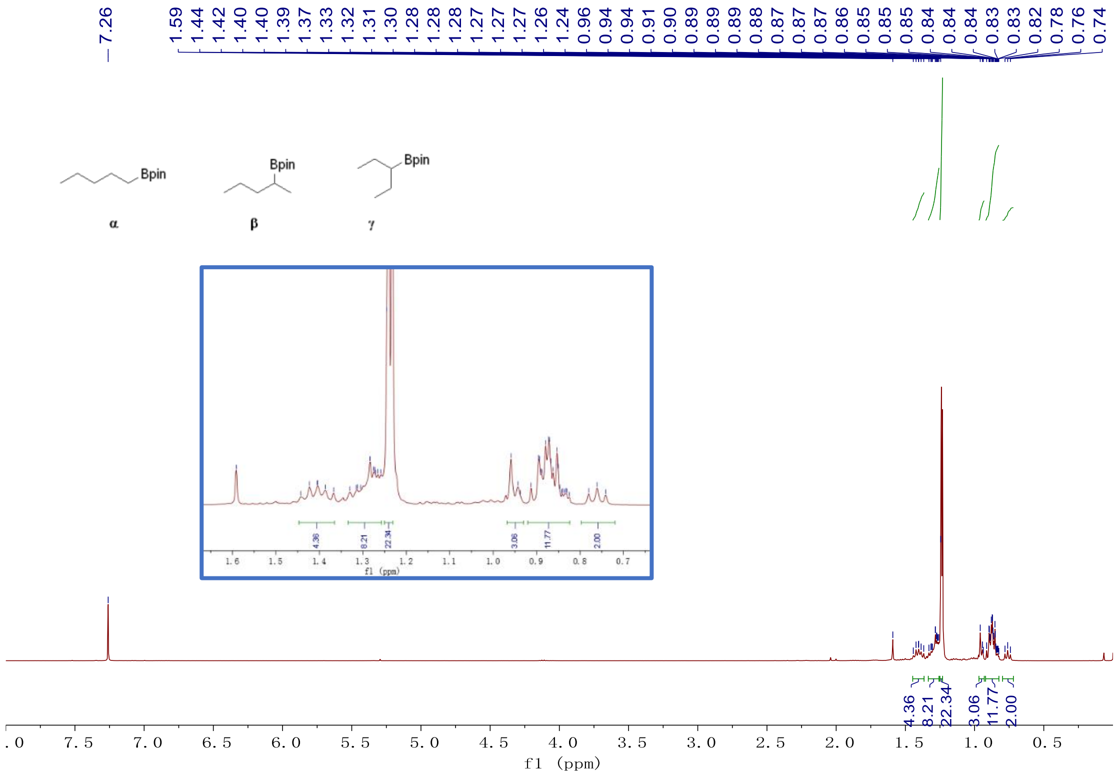

^{13}C NMR (101 MHz, $CDCl_3$) of **13** (*see procedure*):

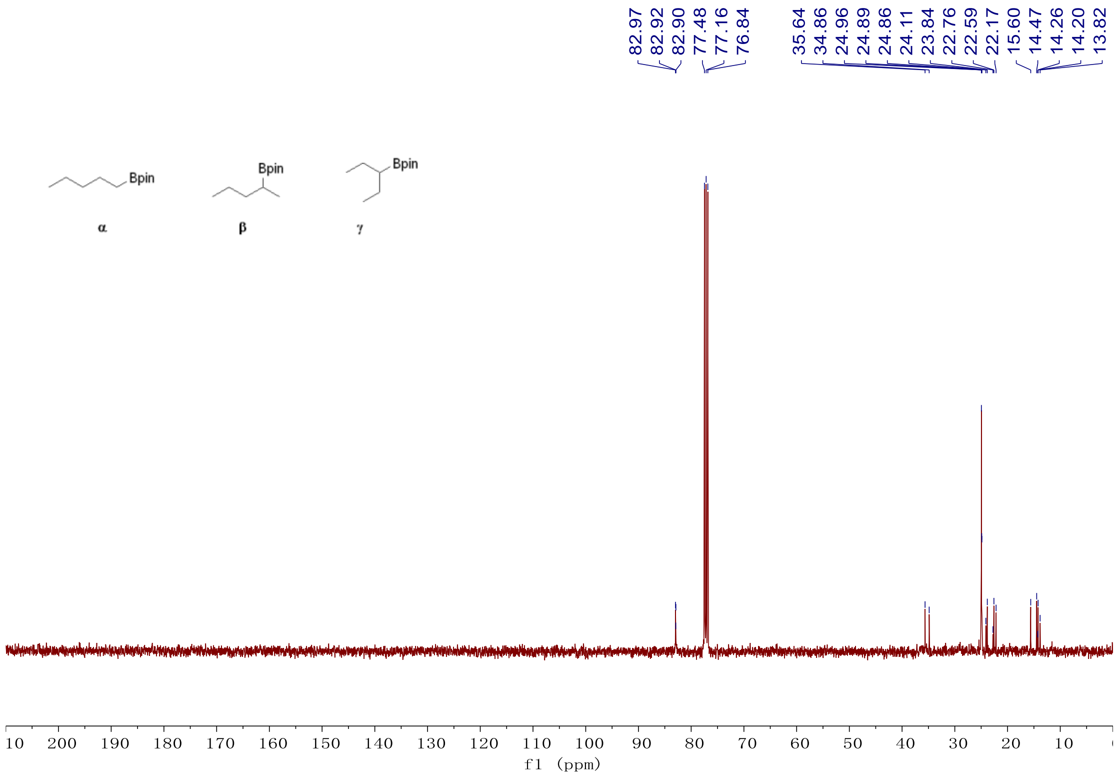

^{1}H NMR (400 MHz, $CDCl_3$) of **14** (*see procedure*):

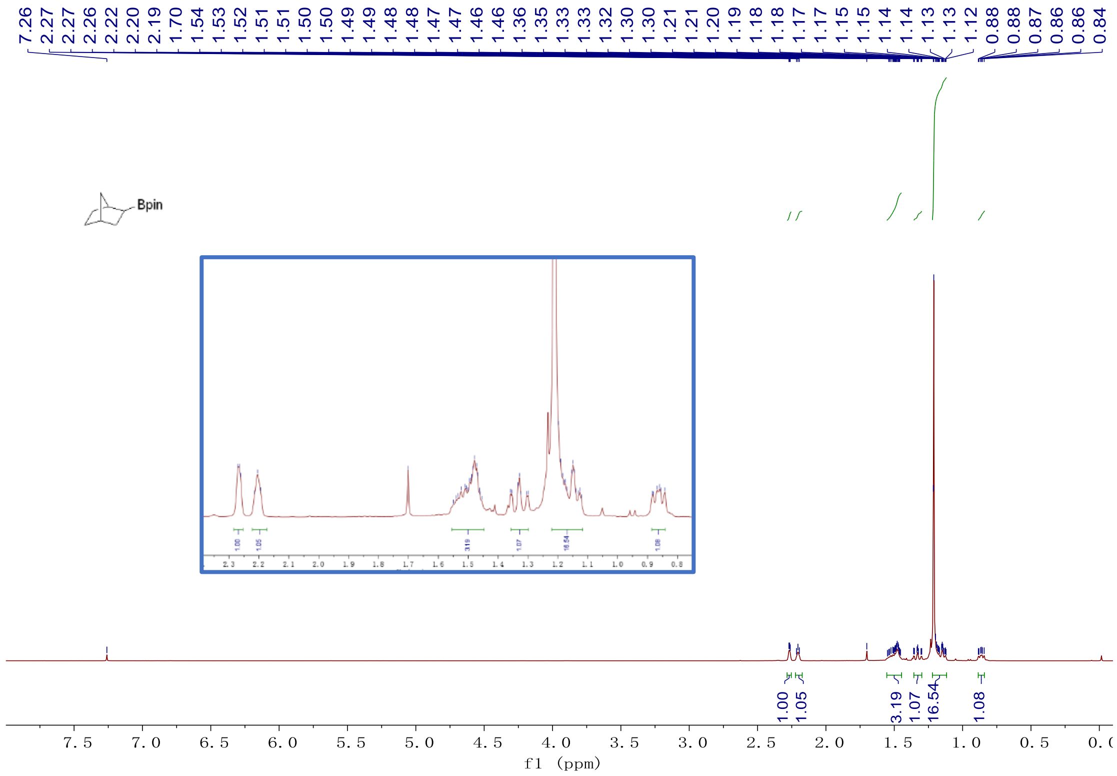

^{13}C NMR (101 MHz, $CDCl_3$) of **14** (*see procedure*):

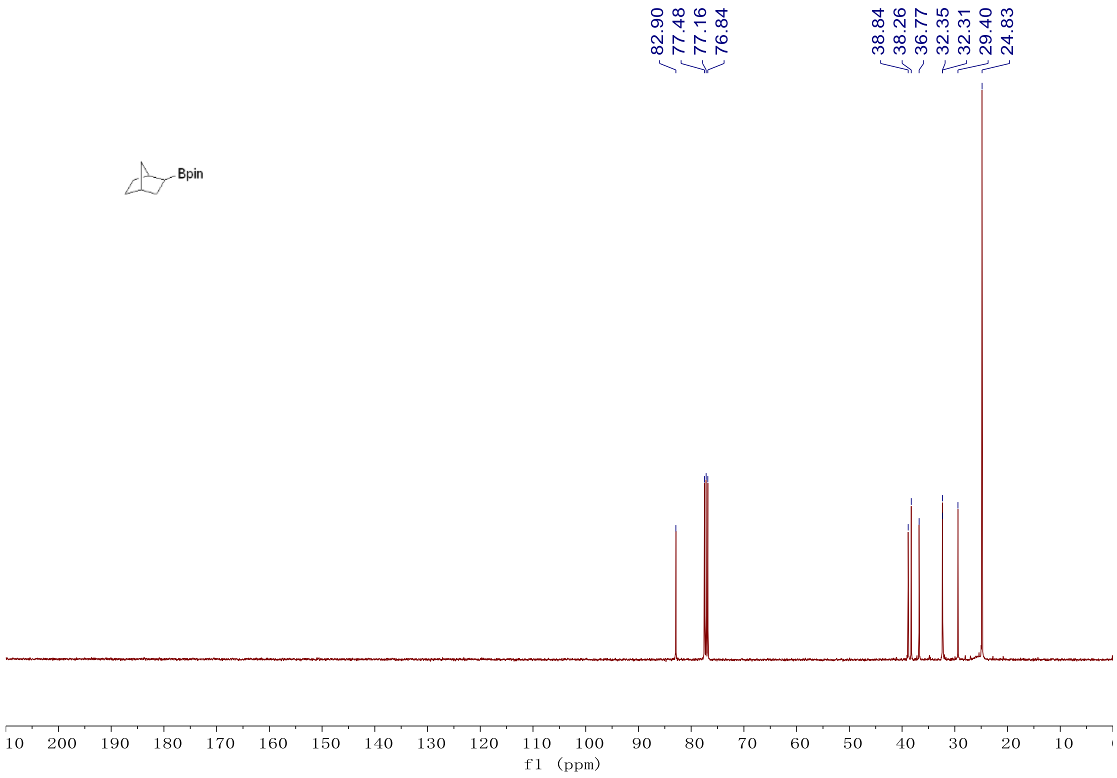

^{1}H NMR (400 MHz, $CDCl_3$) of **15** (*see procedure*):

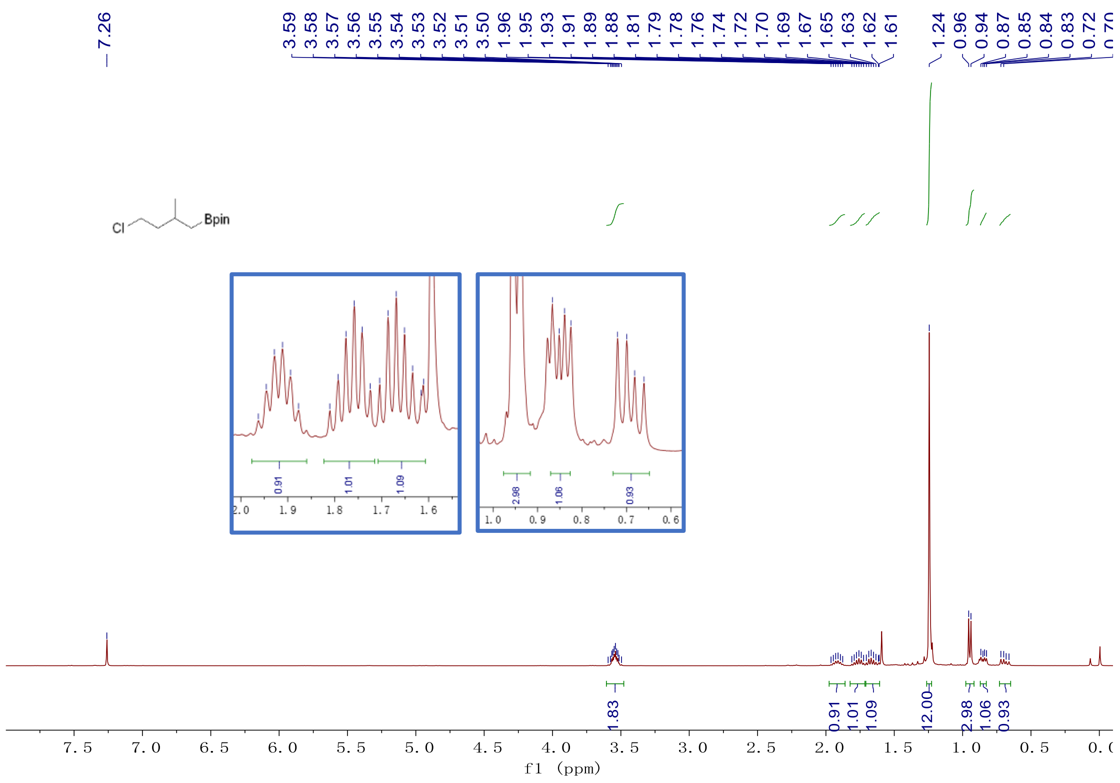

^{13}C NMR (101 MHz, $CDCl_3$) of **15** (*see procedure*):

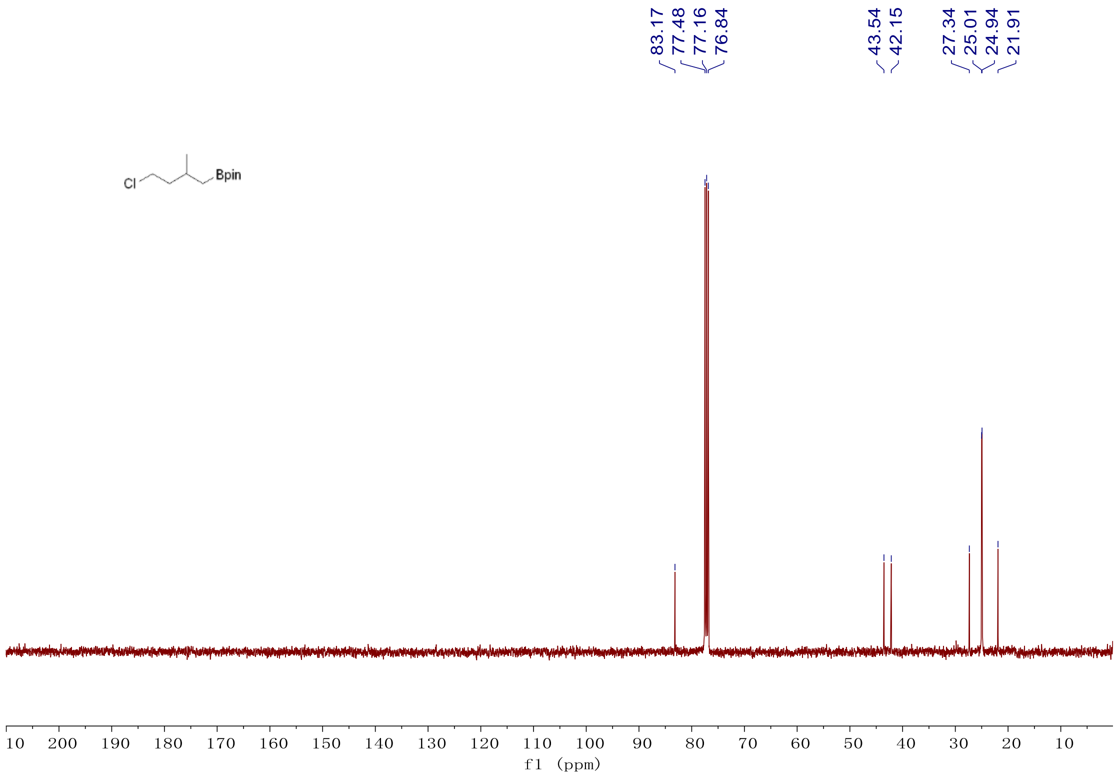

^{1}H NMR (400 MHz, $CDCl_3$) of **16** (see procedure):

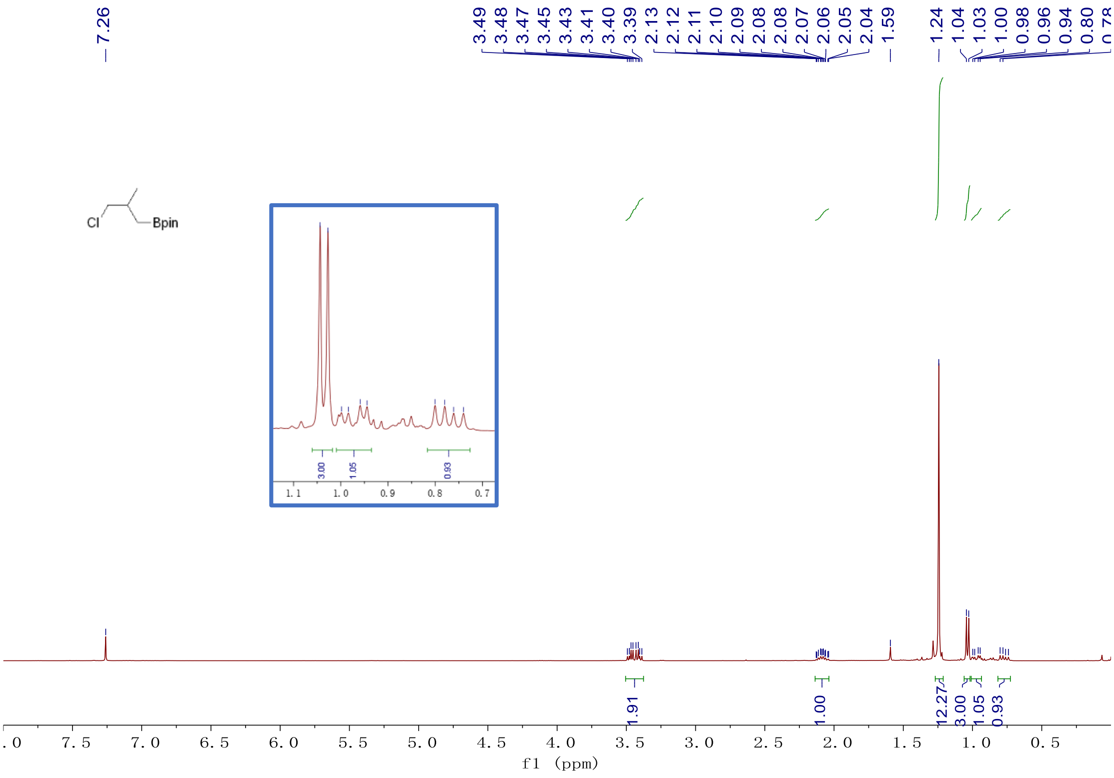

^{13}C NMR (101 MHz, $CDCl_3$) of **16** (see procedure):

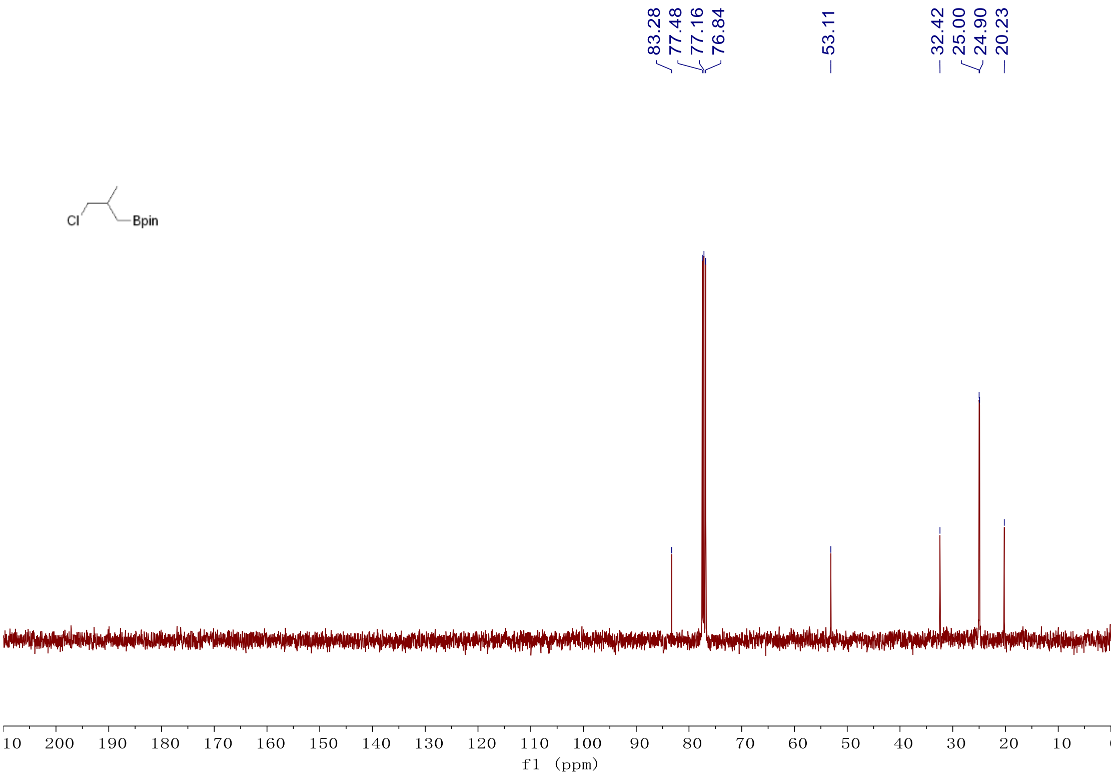

^{1}H NMR (400 MHz, $CDCl_3$) of **17** (*see procedure*):

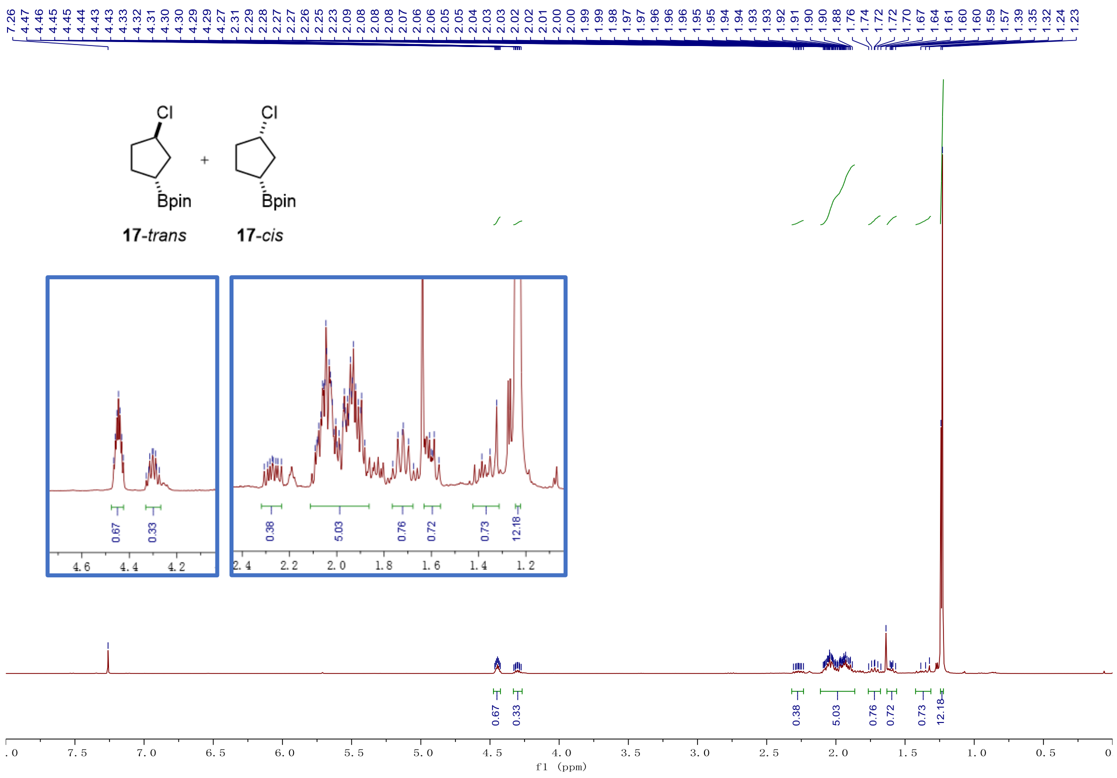

^{13}C NMR (101 MHz, $CDCl_3$) of **17** (*see procedure*):

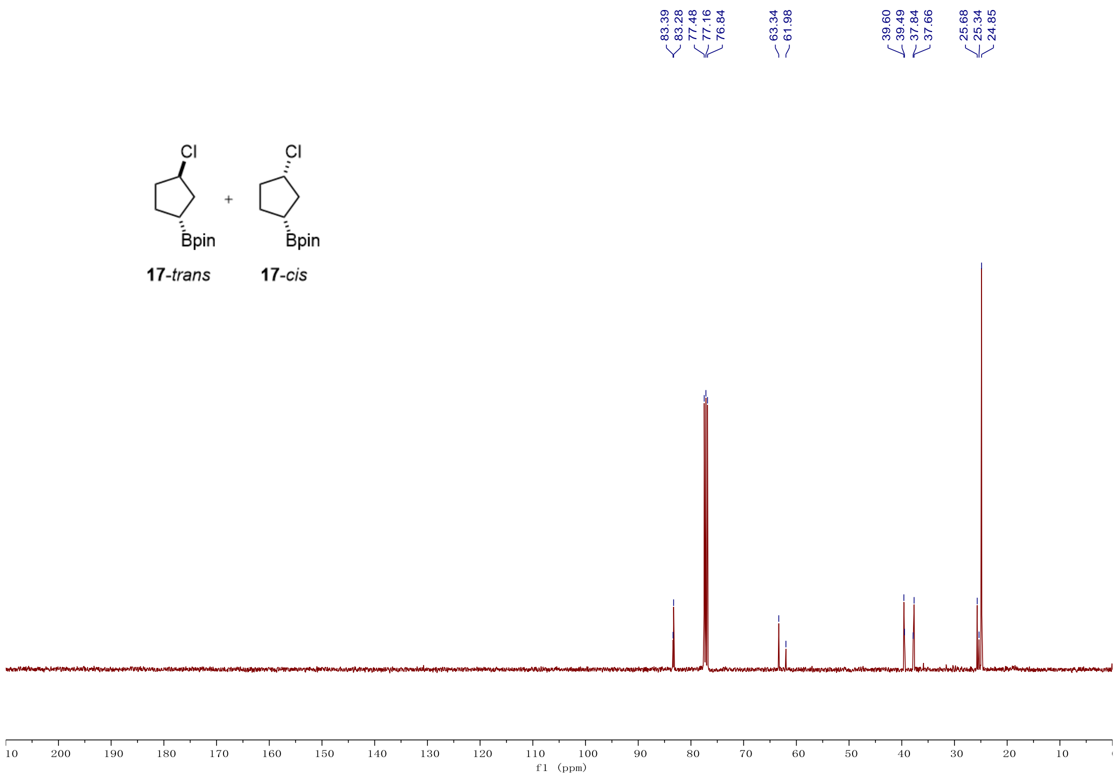

^{1}H NMR (400 MHz, $CDCl_3$) of **18** (see procedure):

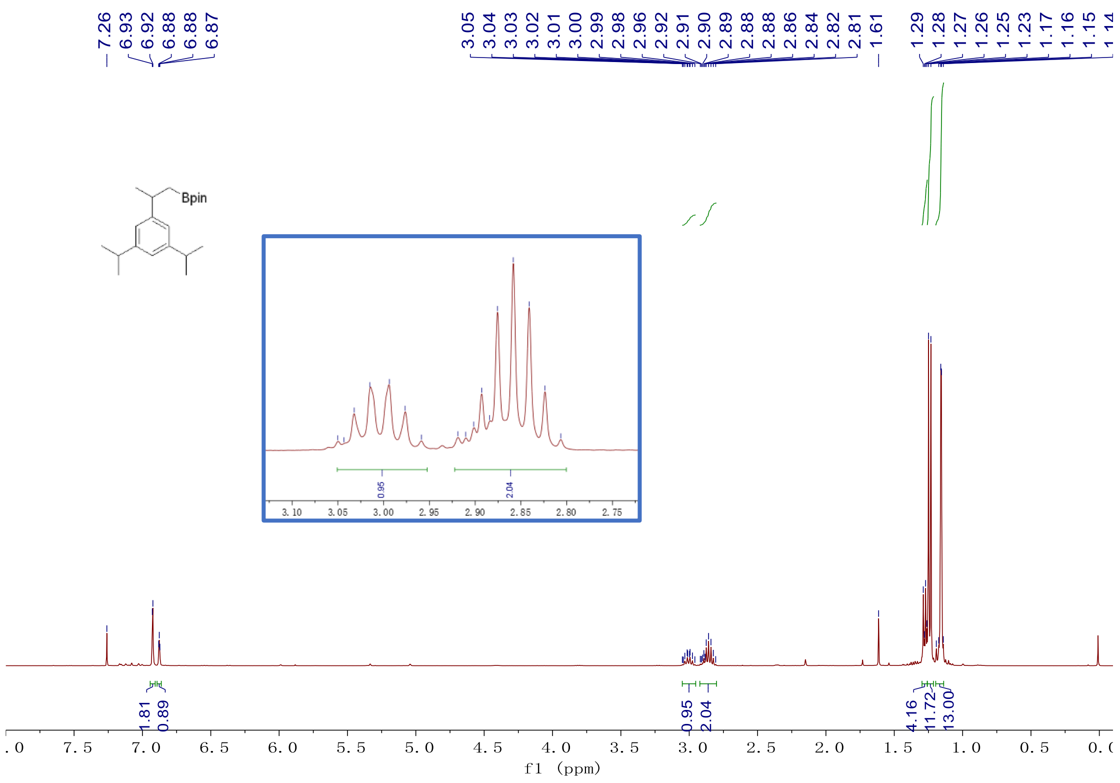

^{13}C NMR (101 MHz, $CDCl_3$) of **18** (see procedure):

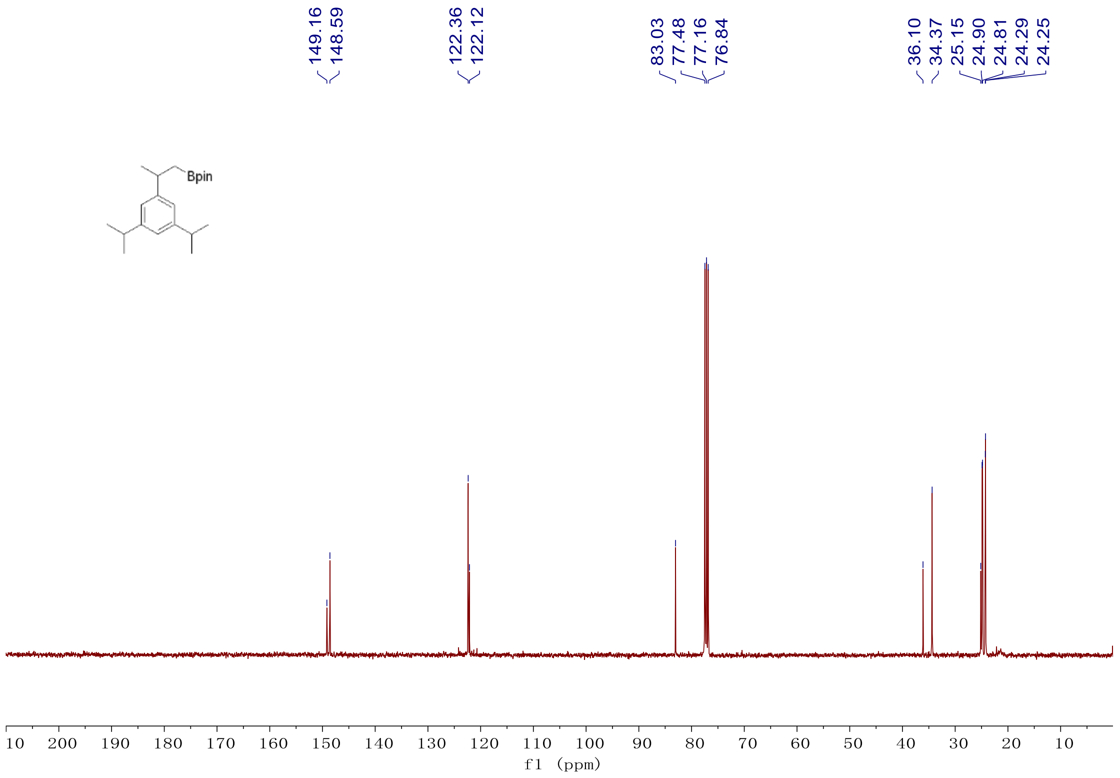

^{1}H NMR (400 MHz, $CDCl_3$) of **19** (*see procedure*):

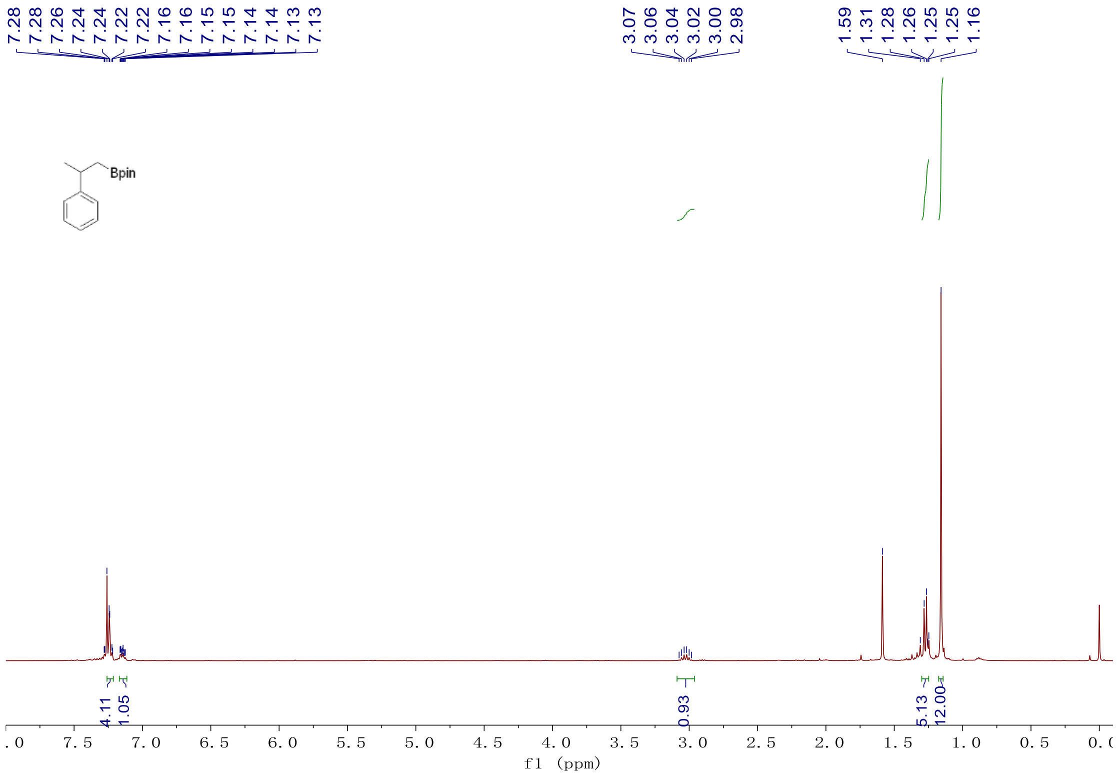

^{13}C NMR (101 MHz, $CDCl_3$) of **19** (*see procedure*):

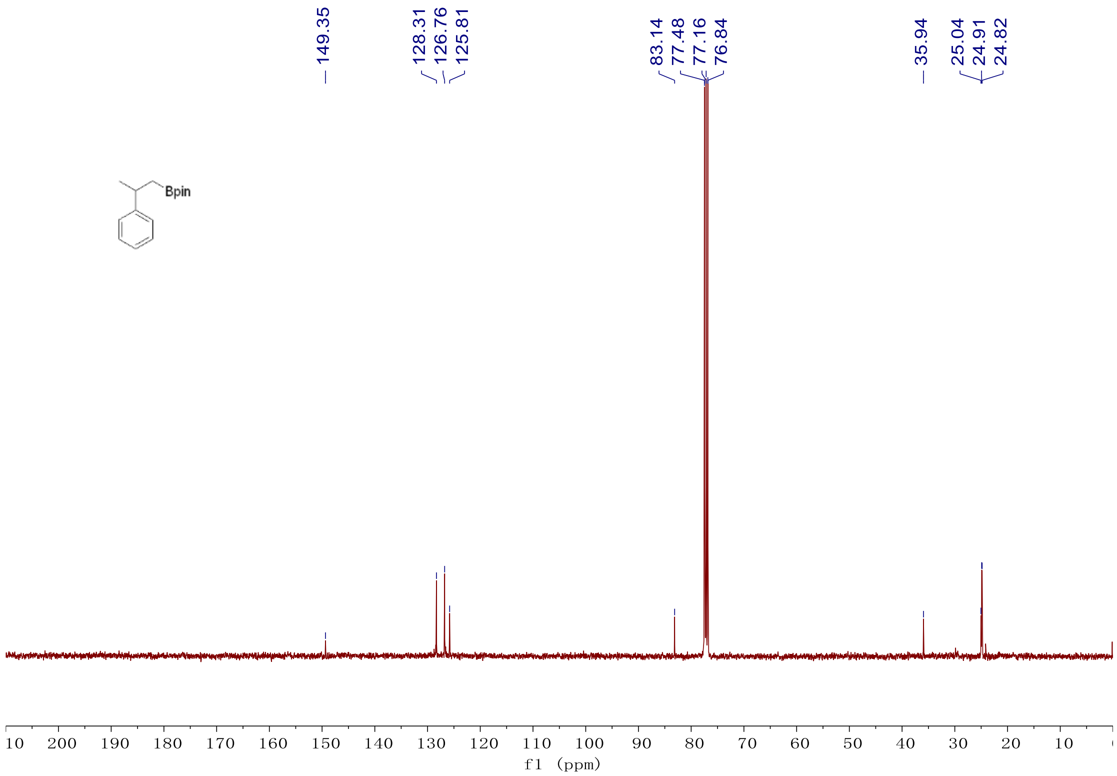

^1H NMR (400 MHz, $CDCl_3$) of **20** (*see procedure*):

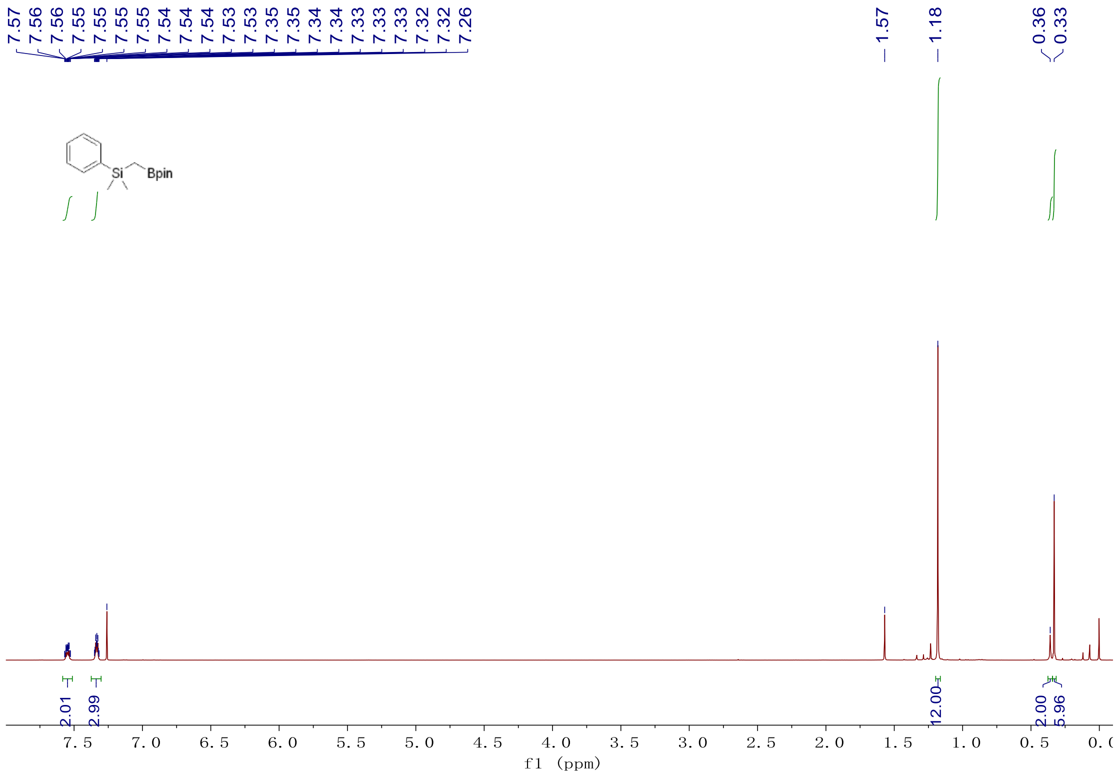

^{13}C NMR (101 MHz, $CDCl_3$) of **20** (*see procedure*):

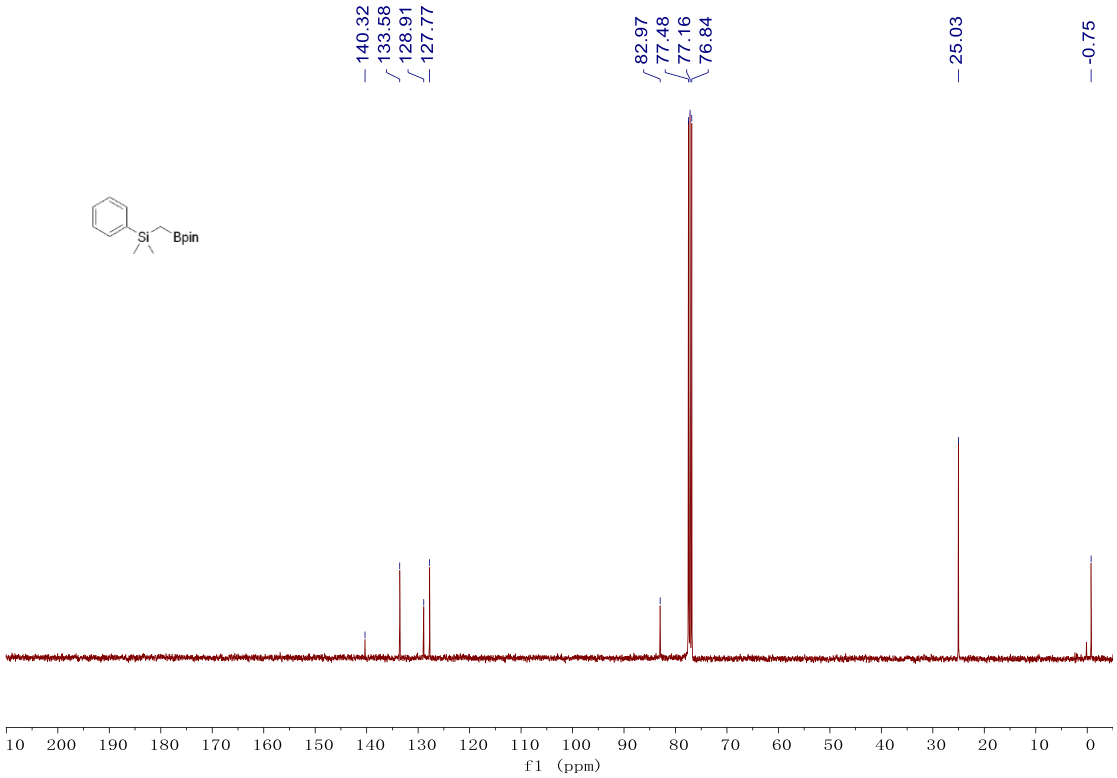

^{1}H NMR (400 MHz, $CDCl_3$) of **21** (*see procedure*):

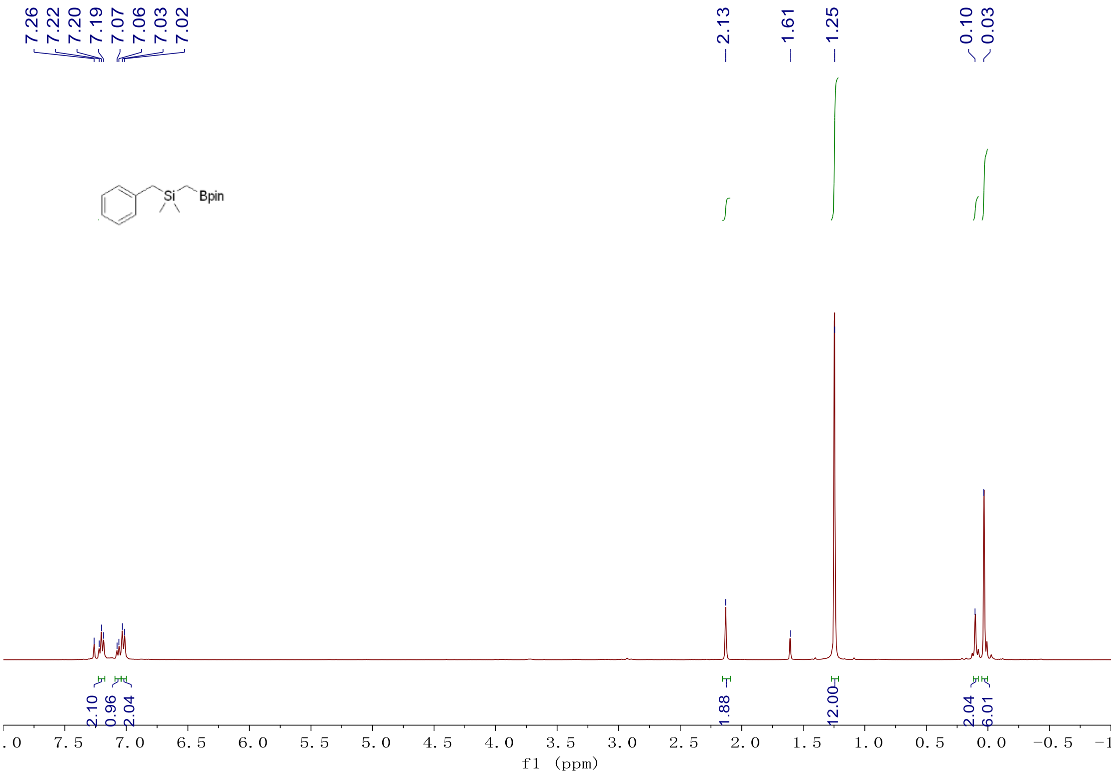

^{13}C NMR (101 MHz, $CDCl_3$) of **21** (*see procedure*):

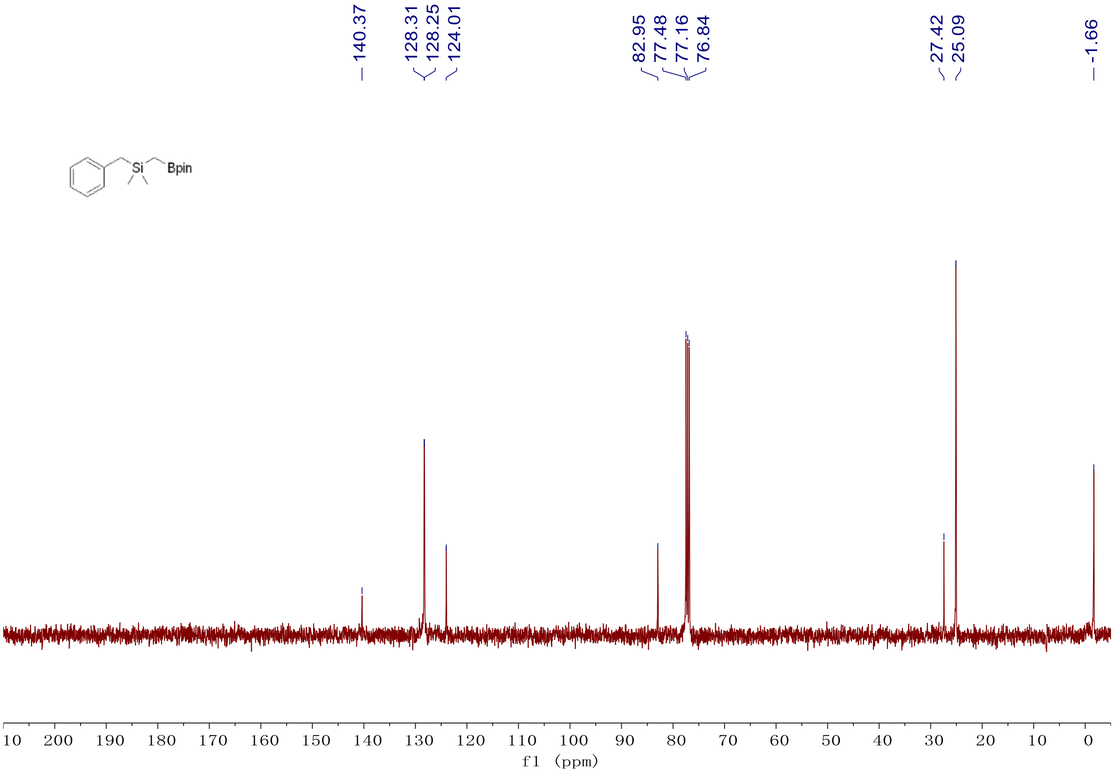

^{1}H NMR (400 MHz, $CDCl_3$) of **22** (*see procedure*):

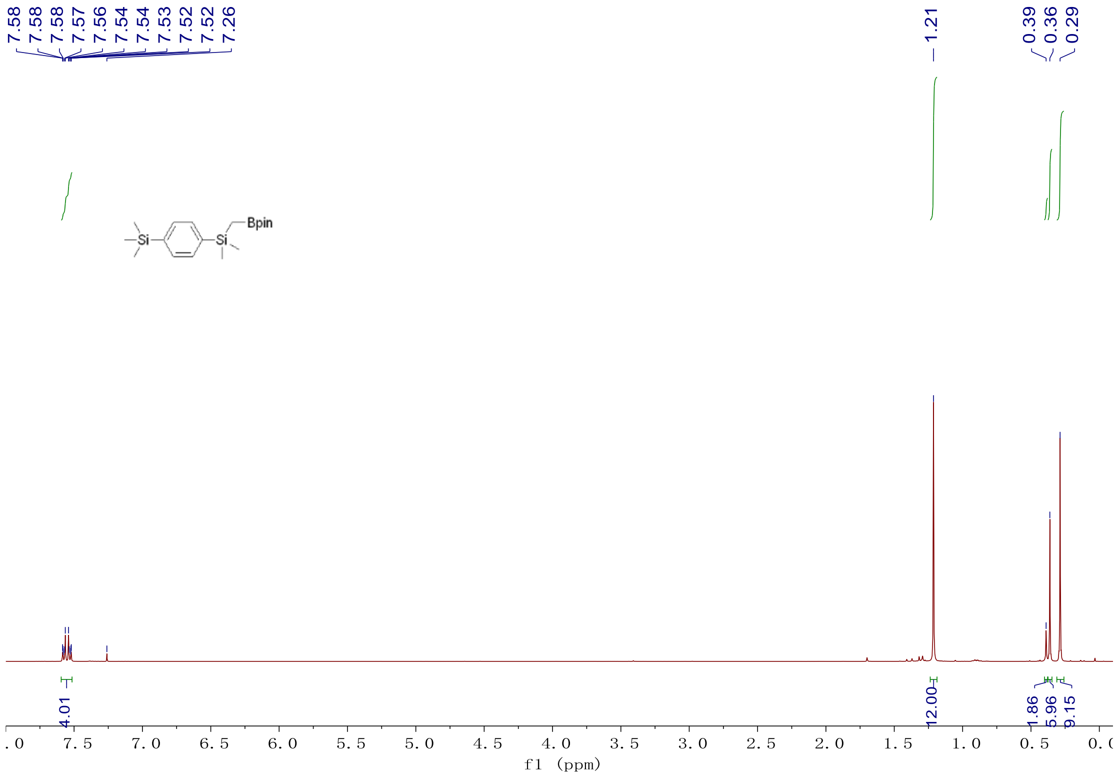

^{13}C NMR (101 MHz, $CDCl_3$) of **22** (*see procedure*):

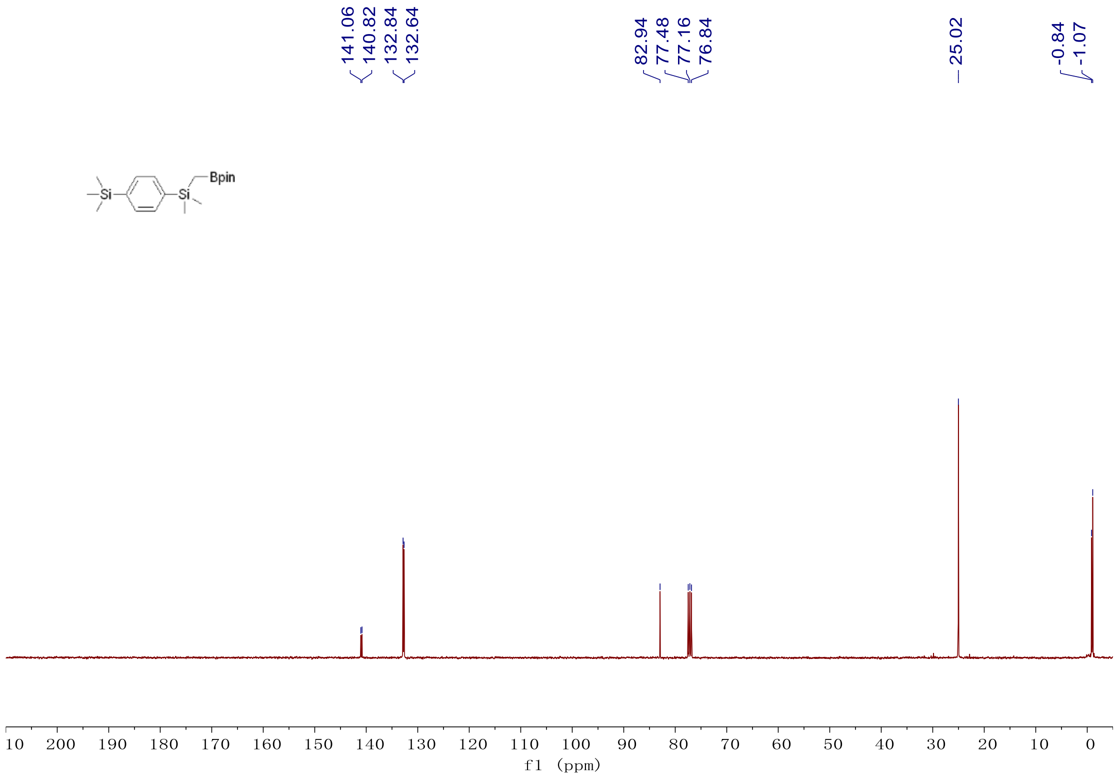

^{1}H NMR (400 MHz, $CDCl_3$) of **23** (*see procedure*):

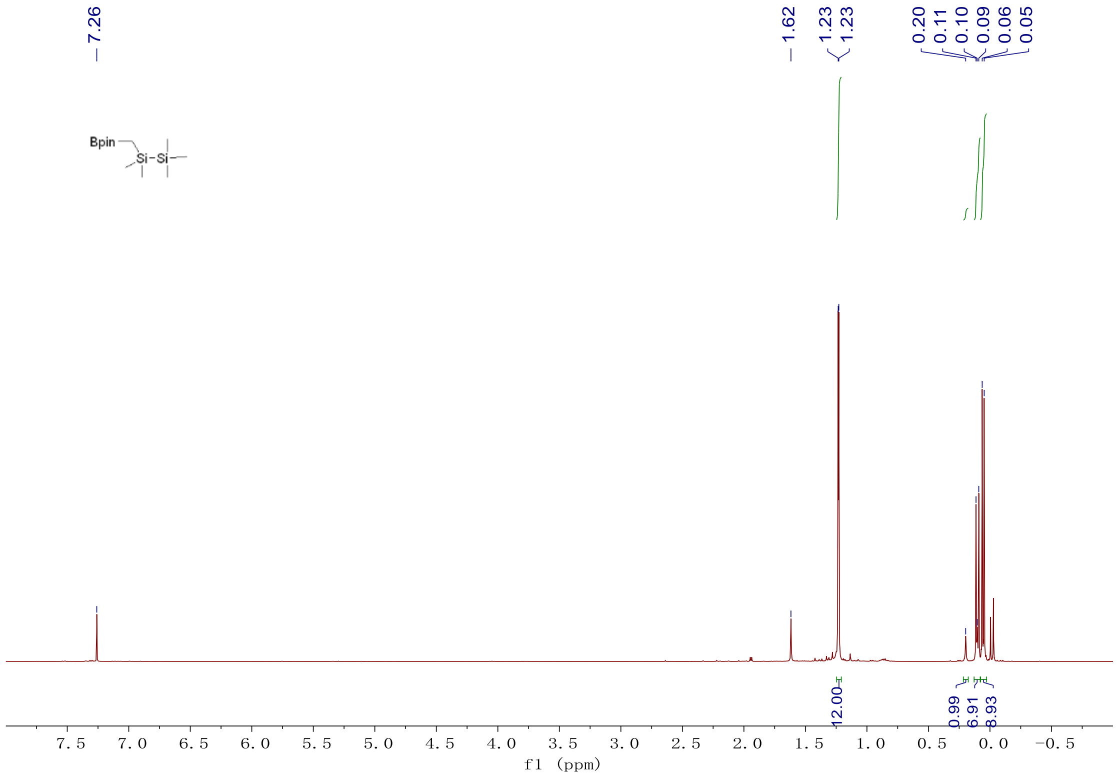

^{13}C NMR (101 MHz, $CDCl_3$) of **23** (*see procedure*):

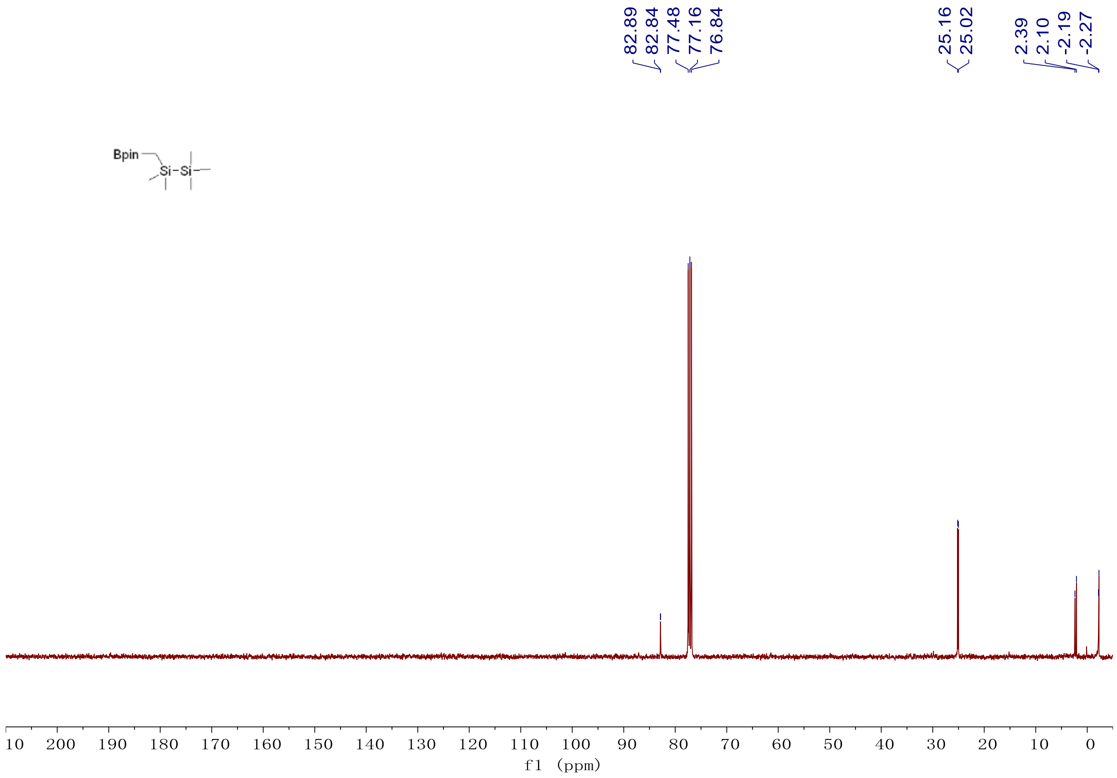

^{1}H NMR (400 MHz, $CDCl_3$) of **24** (*see procedure*):

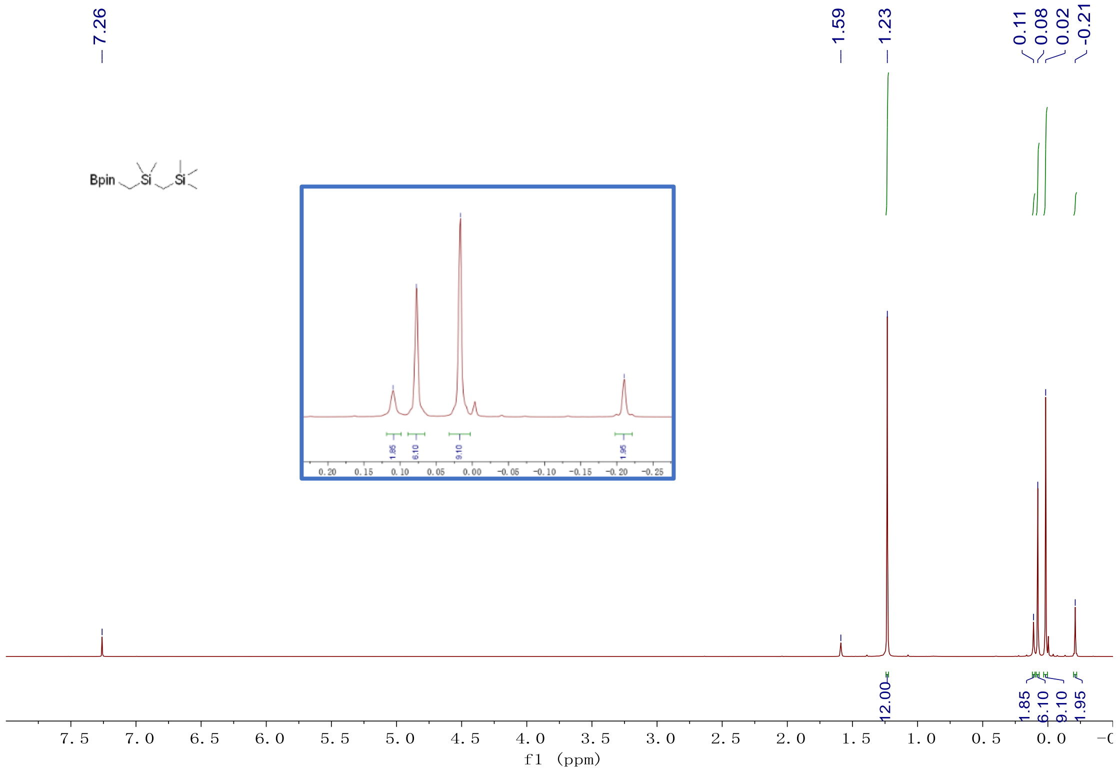

^{13}C NMR (101 MHz, $CDCl_3$) of **24** (*see procedure*):

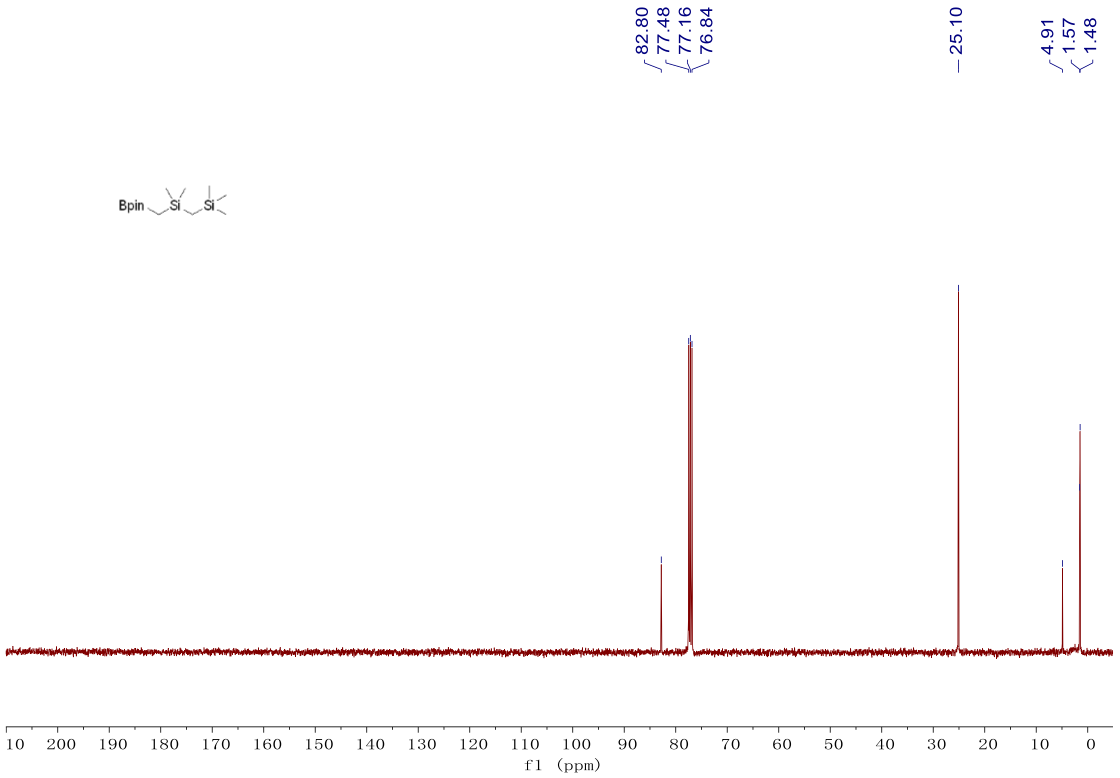

^{1}H NMR (400 MHz, $CDCl_3$) of **25** (*see procedure*):

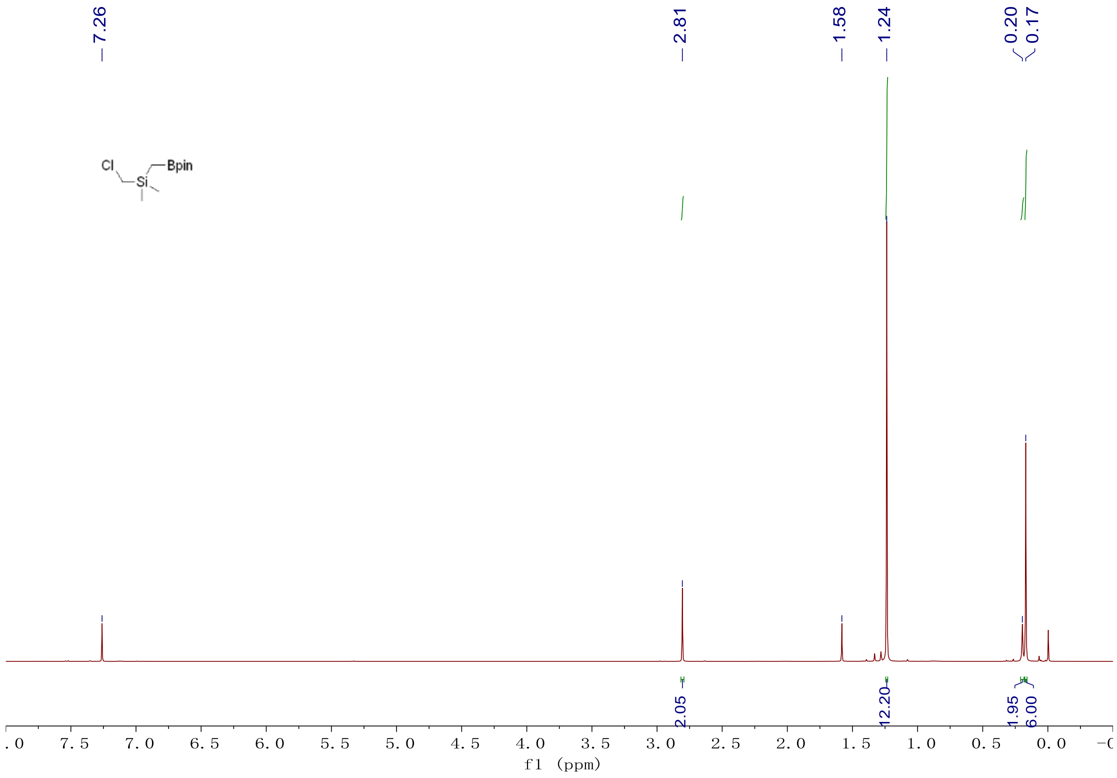

^{13}C NMR (101 MHz, $CDCl_3$) of **25** (*see procedure*):

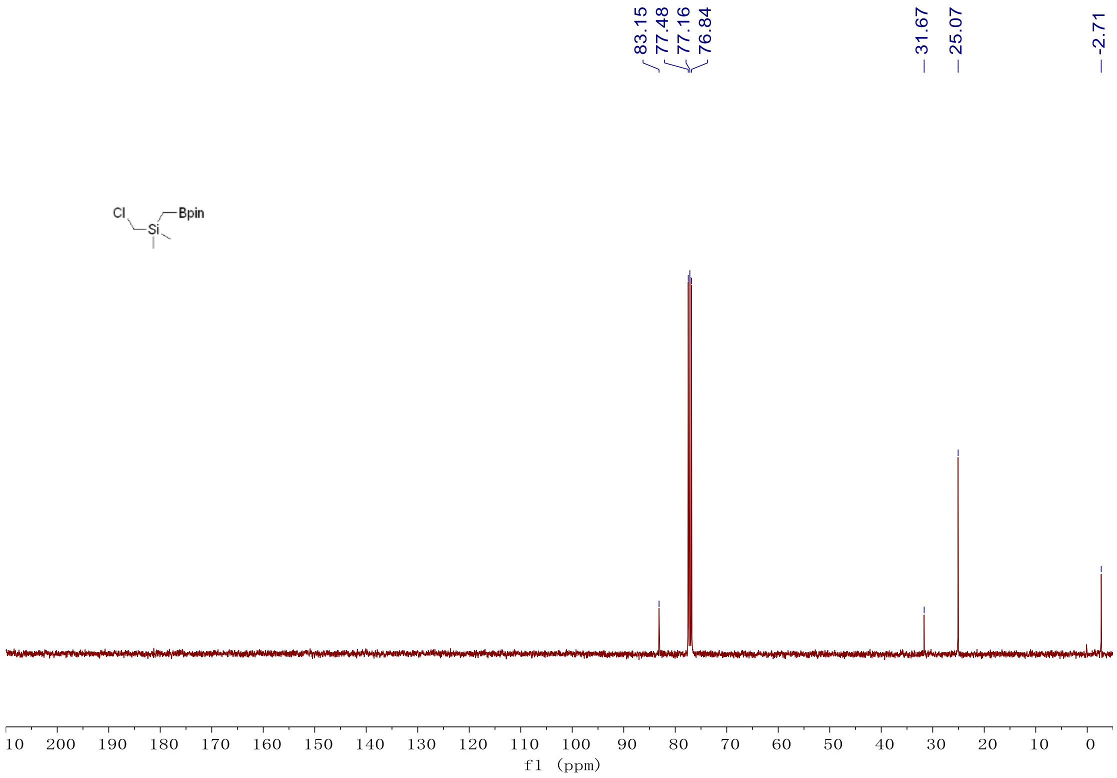

^{1}H NMR (400 MHz, $CDCl_3$) of **26** (<u>*see procedure*</u>):

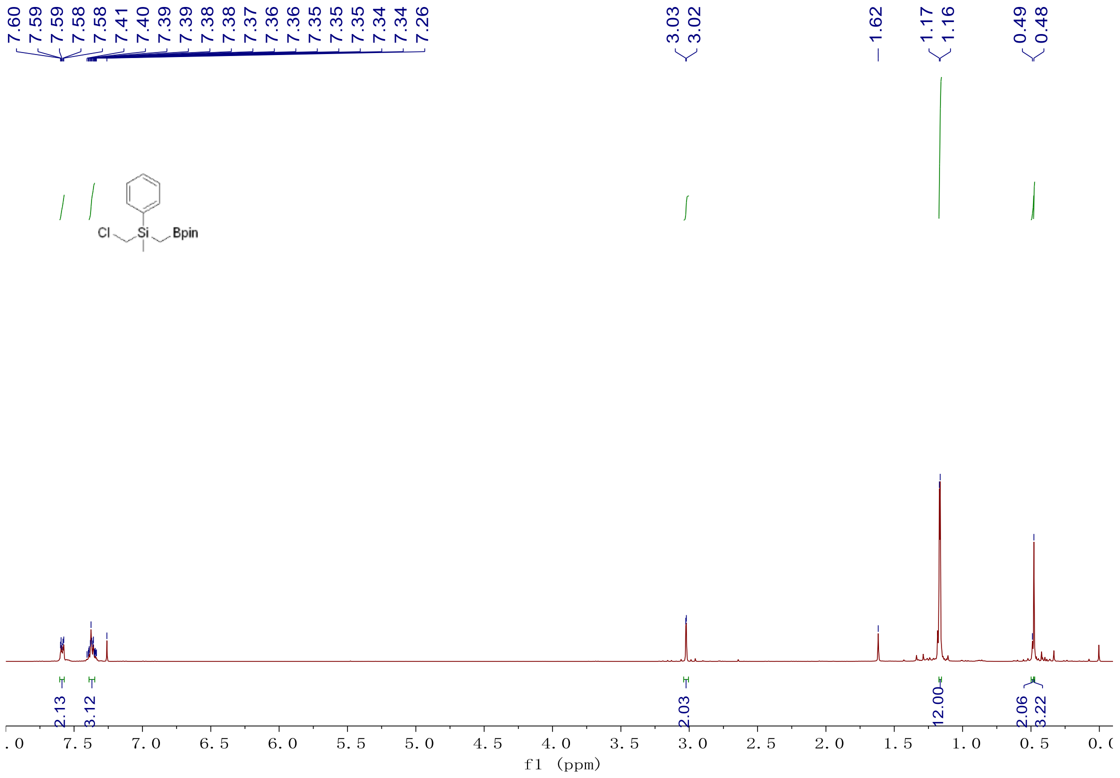

^{13}C NMR (101 MHz, $CDCl_3$) of **26** (<u>*see procedure*</u>):

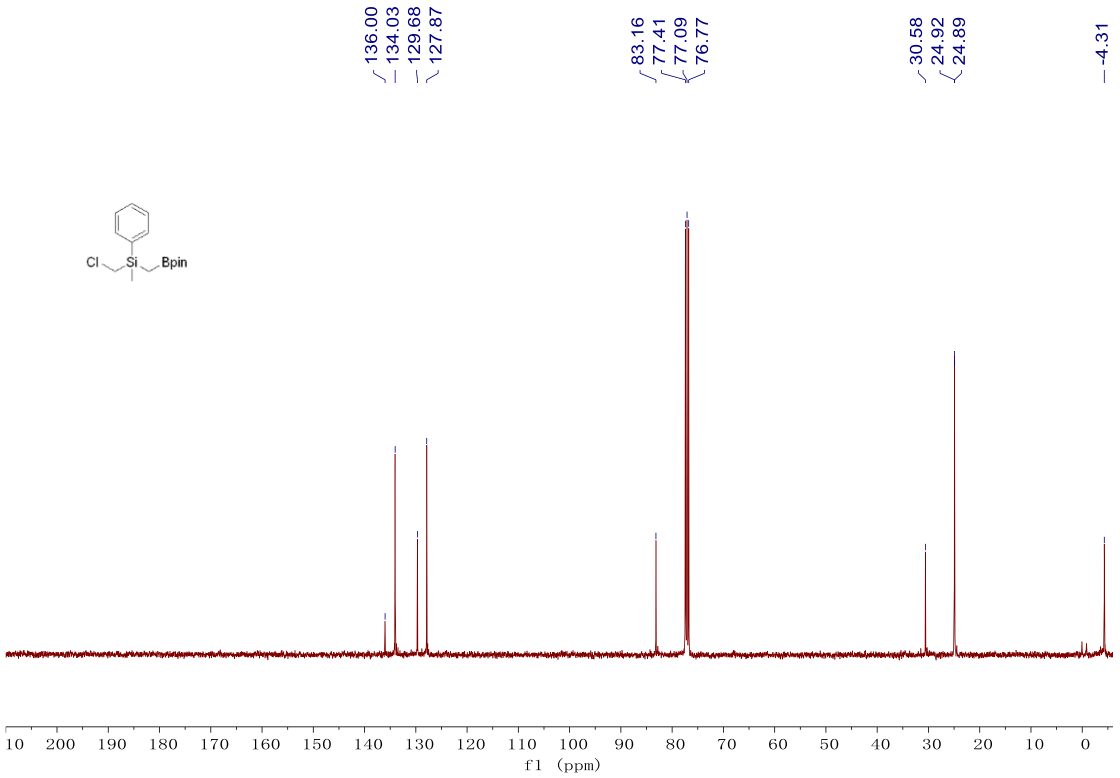

^{1}H NMR (400 MHz, $CDCl_3$) of **27** (*see procedure*):

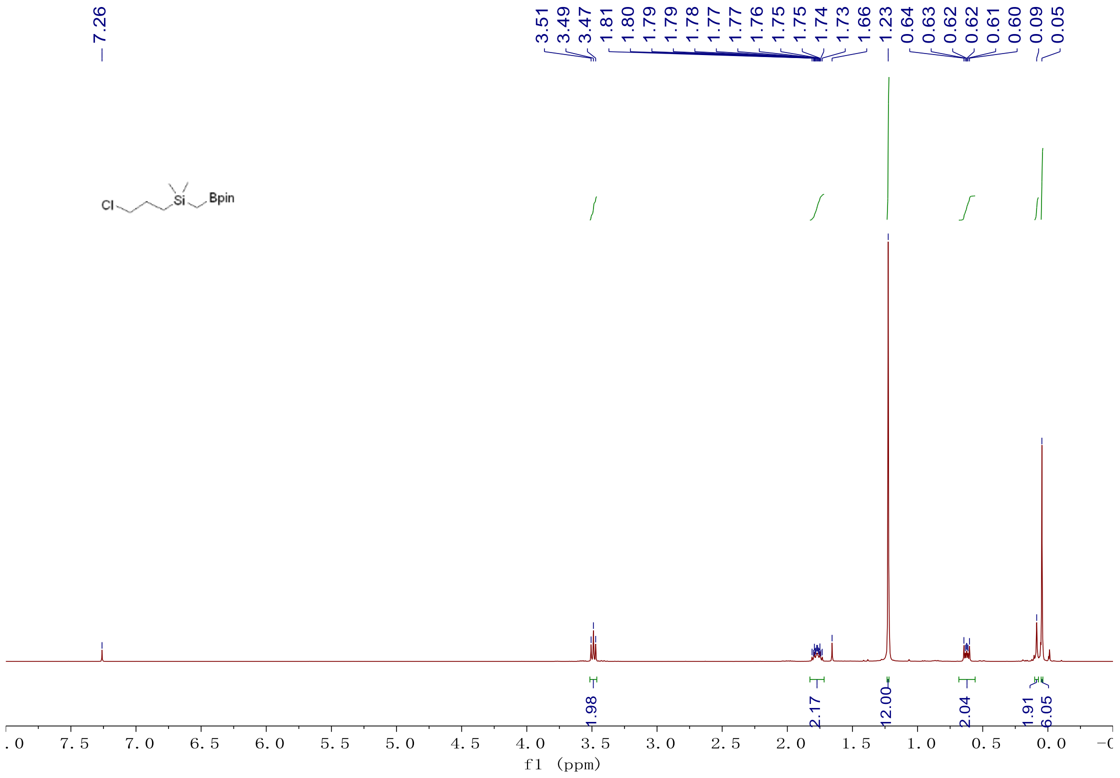

^{13}C NMR (101 MHz, $CDCl_3$) of **27** (*see procedure*):

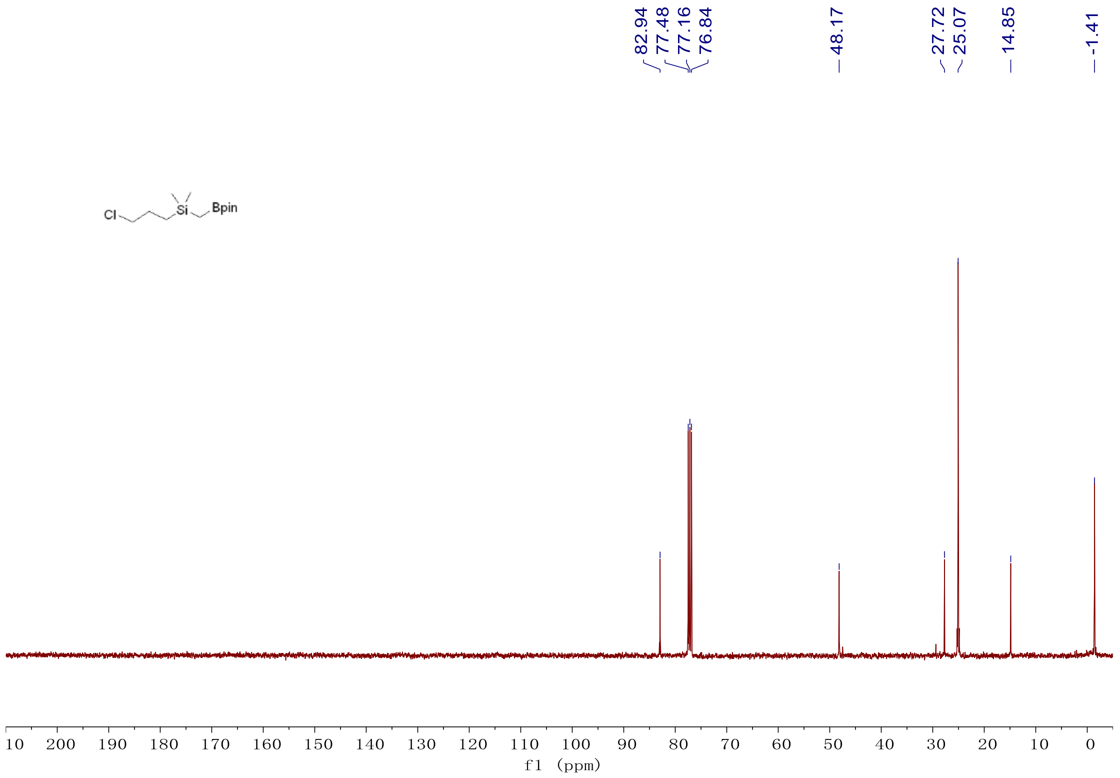

^{1}H NMR (400 MHz, $CDCl_3$) of **28** (*see procedure*):

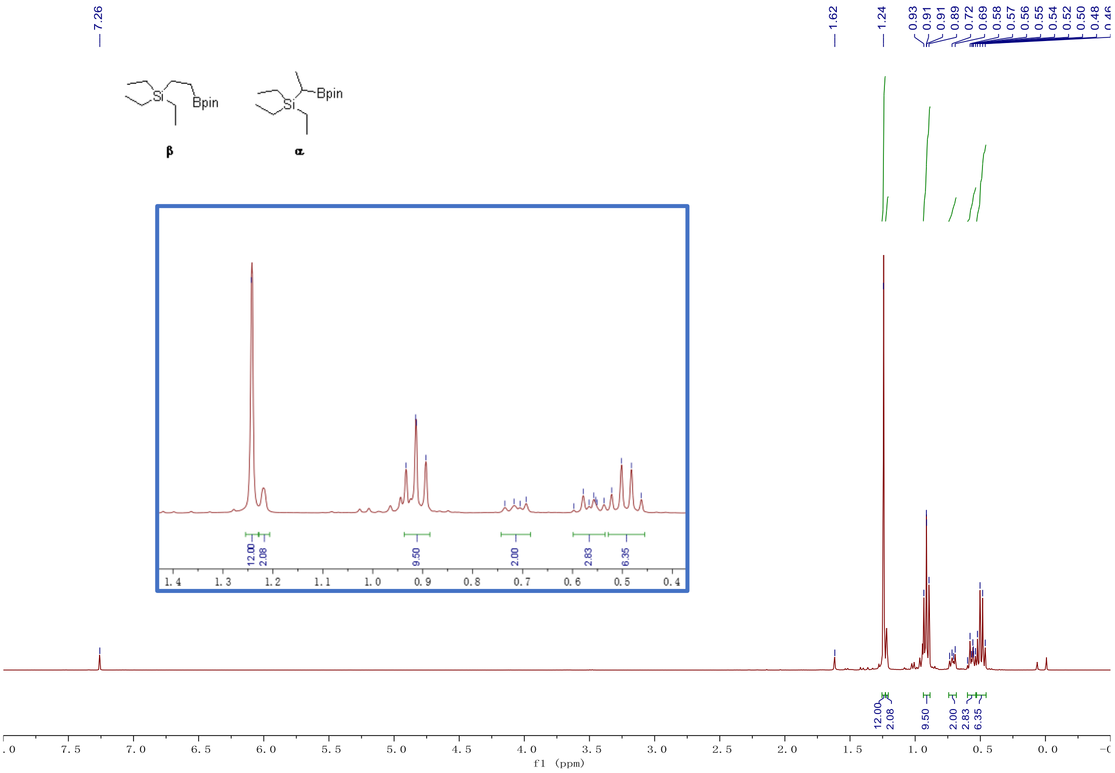

^{13}C NMR (101 MHz, $CDCl_3$) of **28** (*see procedure*):

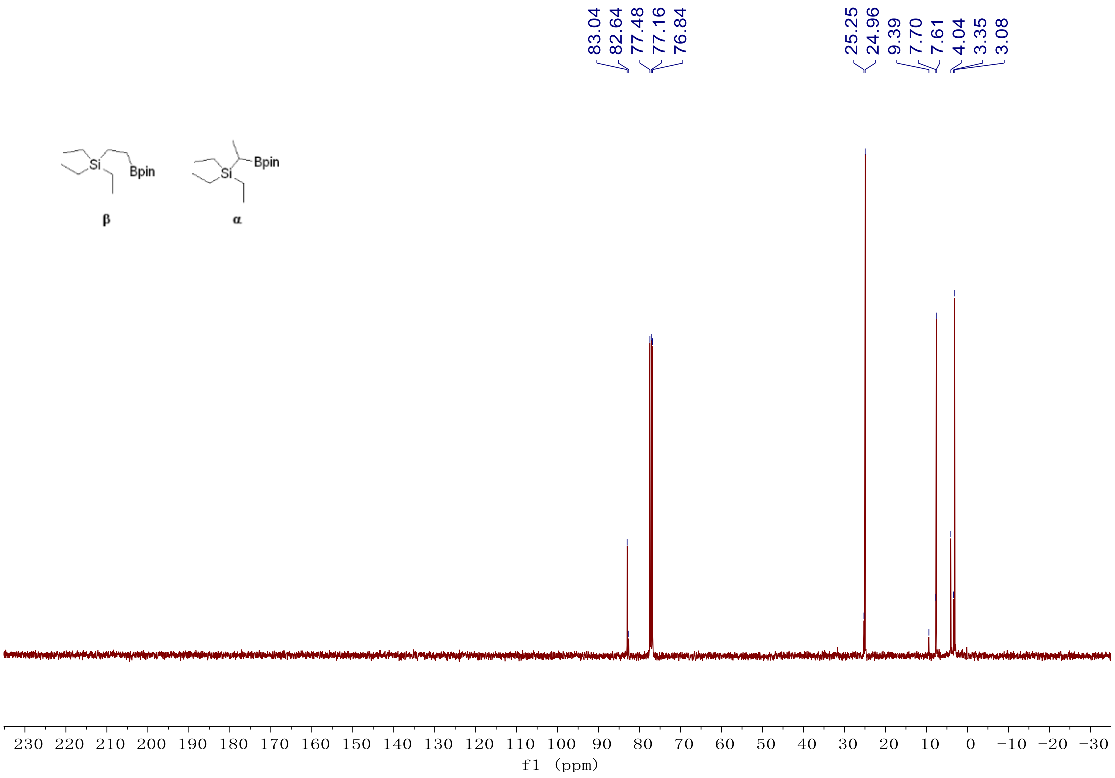

^{1}H NMR (400 MHz, $CDCl_3$) of **29** (*see procedure*):

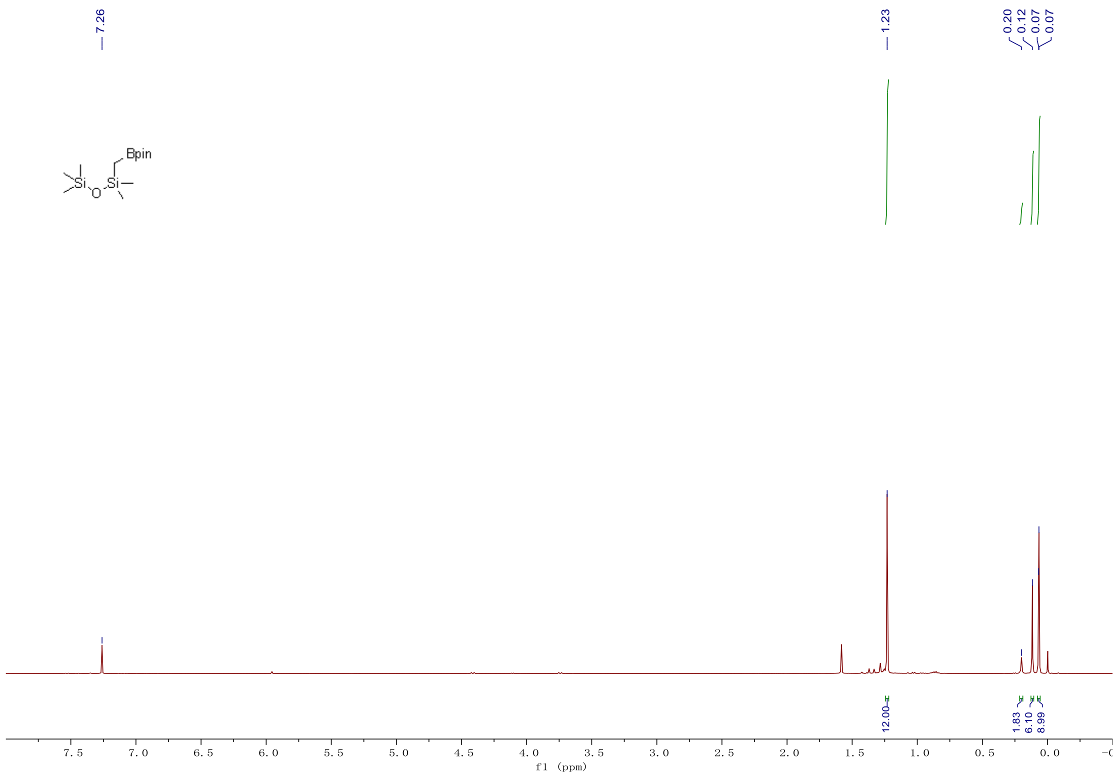

^{13}C NMR (101 MHz, $CDCl_3$) of **29** (*see procedure*):

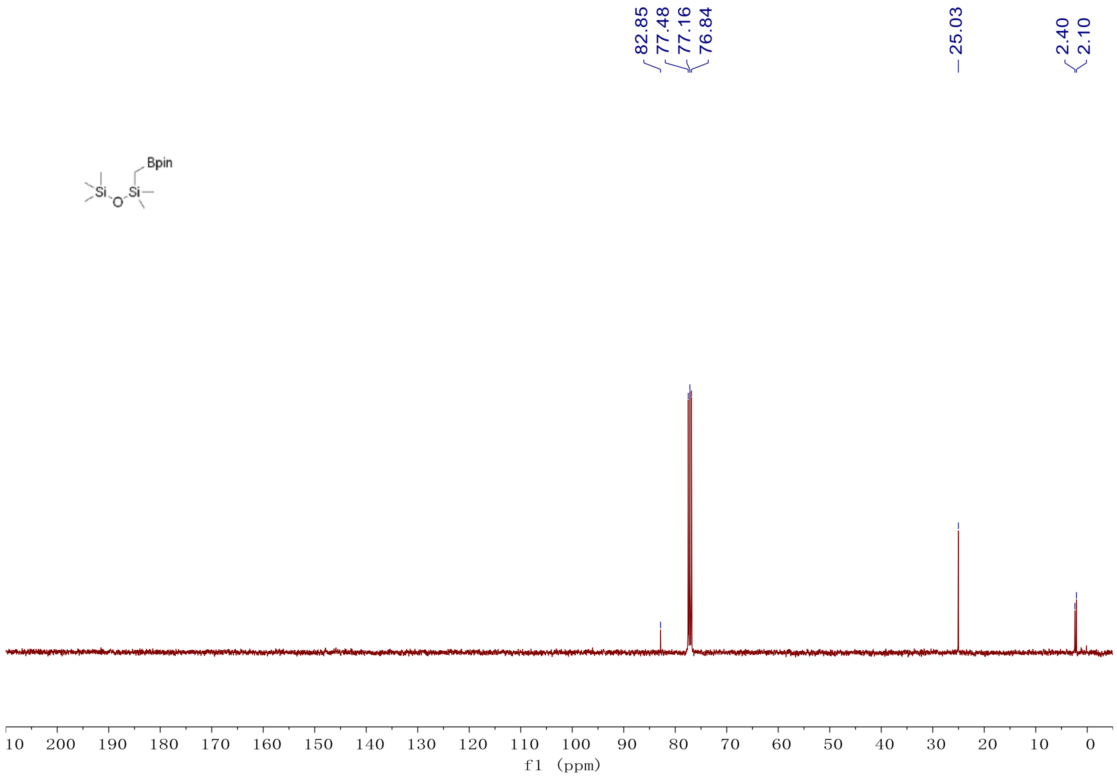

^{1}H NMR (400 MHz, $CDCl_3$) of **30** (*see procedure*):

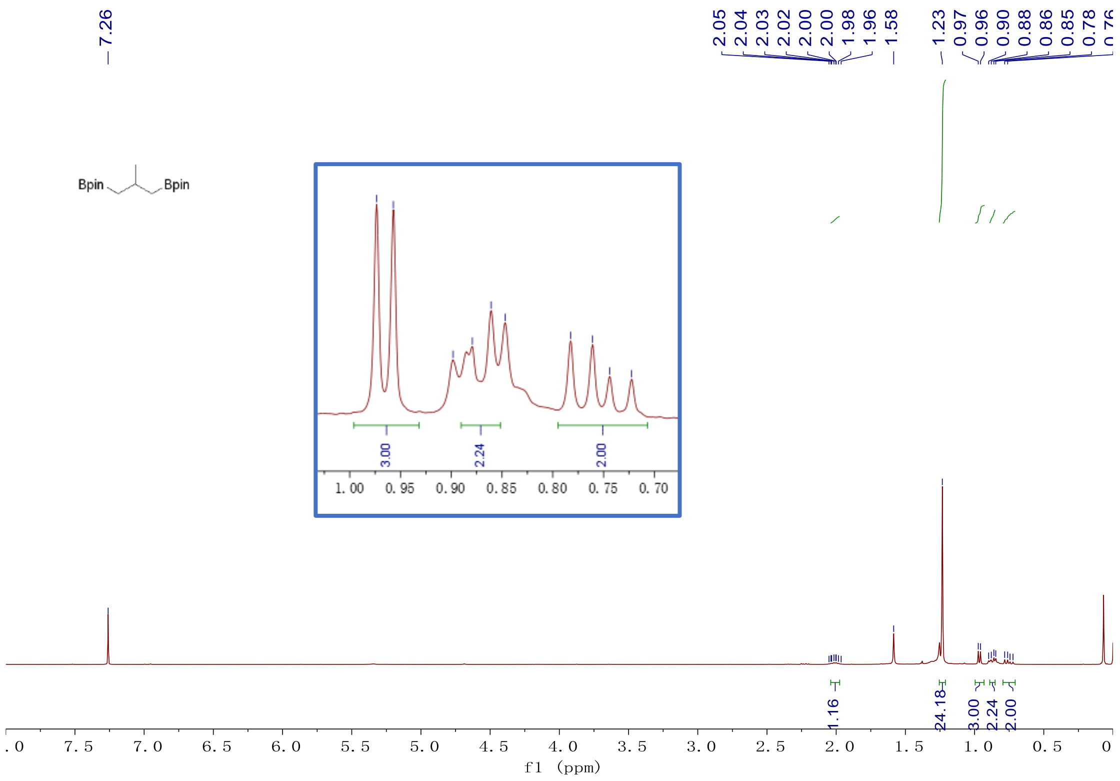

^{13}C NMR (101 MHz, $CDCl_3$) of **30** (*see procedure*):

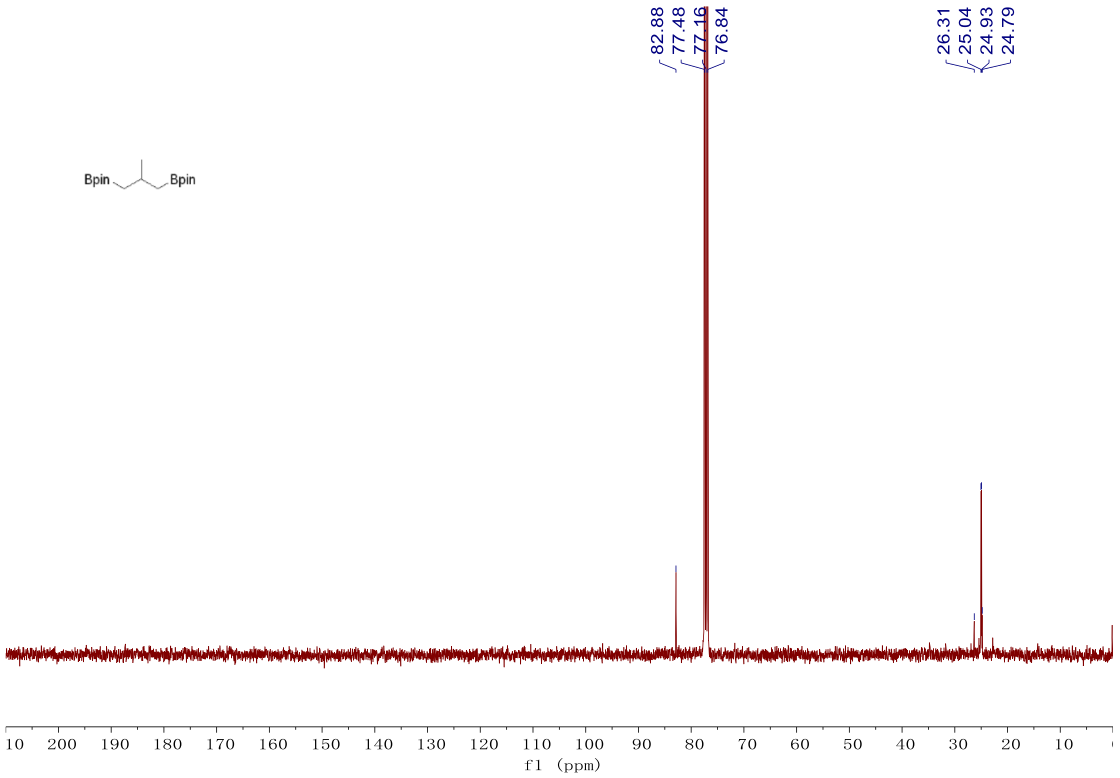

^{1}H NMR (400 MHz, $CDCl_3$) of **31** (*see procedure*):

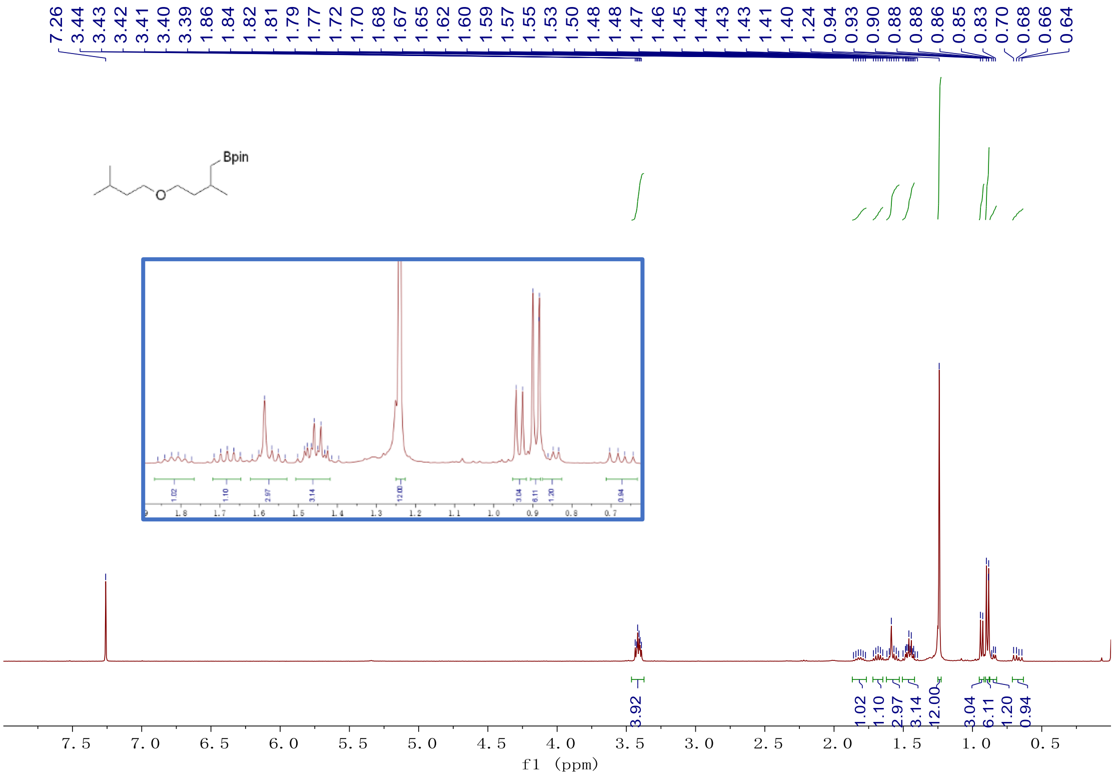

^{13}C NMR (101 MHz, $CDCl_3$) of **31** (*see procedure*):

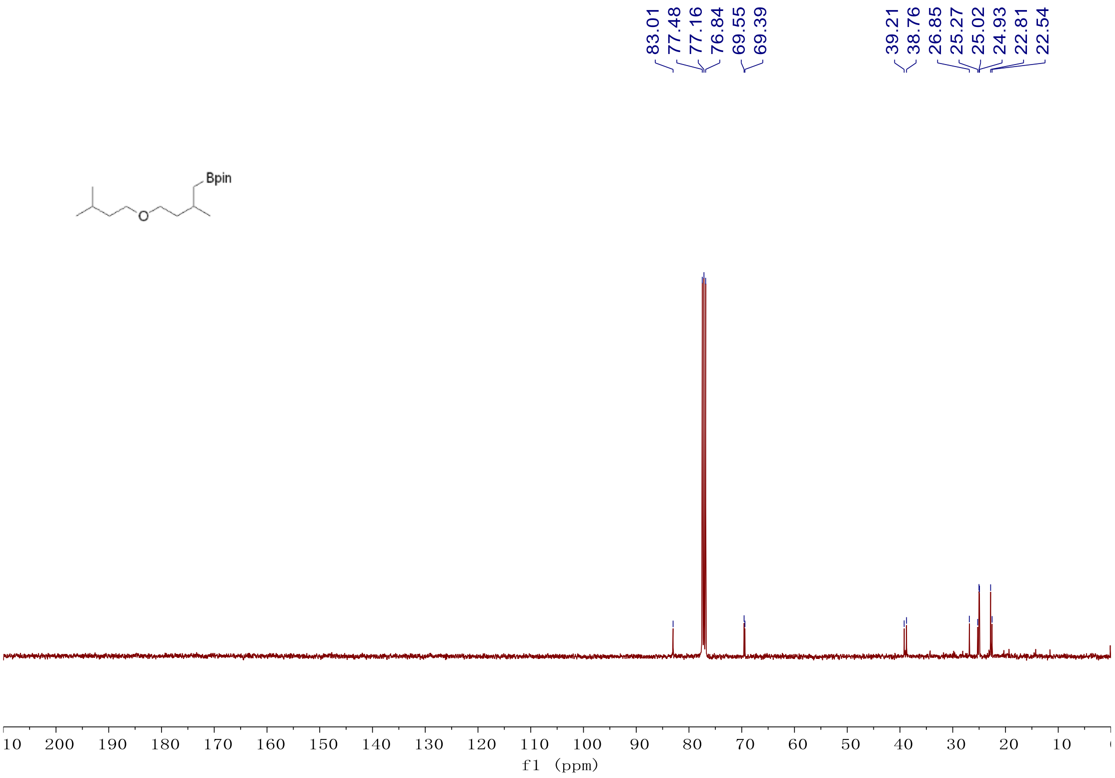

^{1}H NMR (400 MHz, $CDCl_3$) of **32** (*see procedure*):

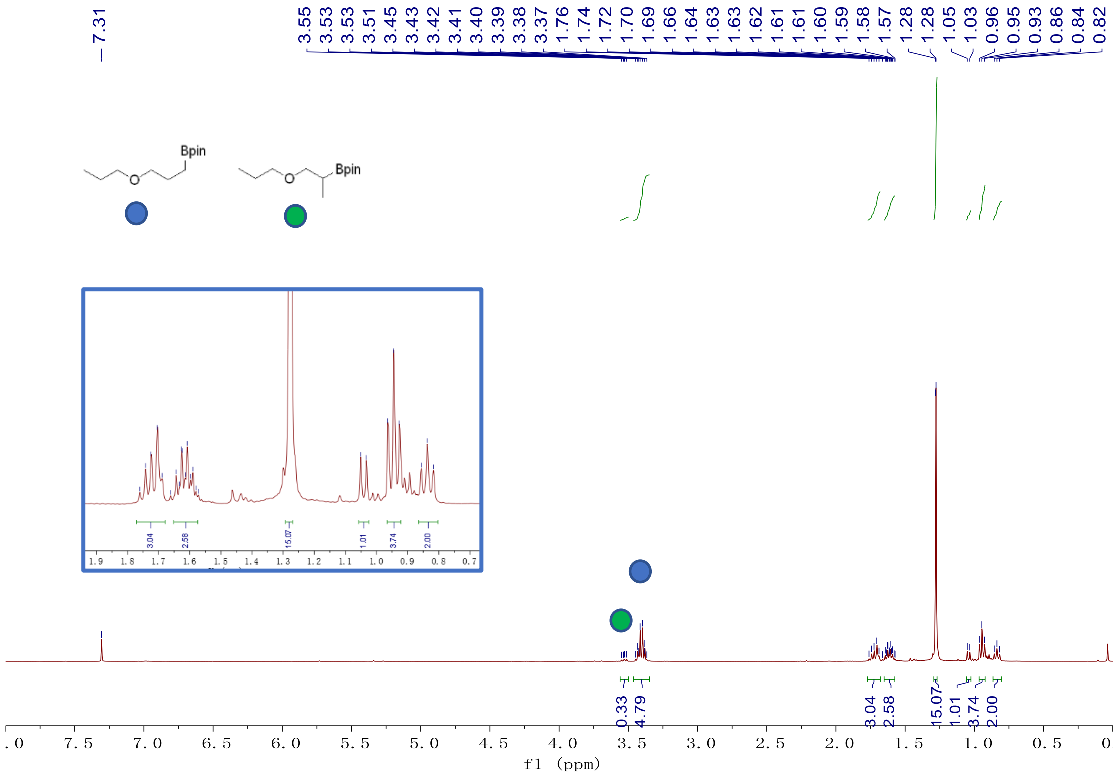

^{13}C NMR (101 MHz, $CDCl_3$) of **32** (*see procedure*):

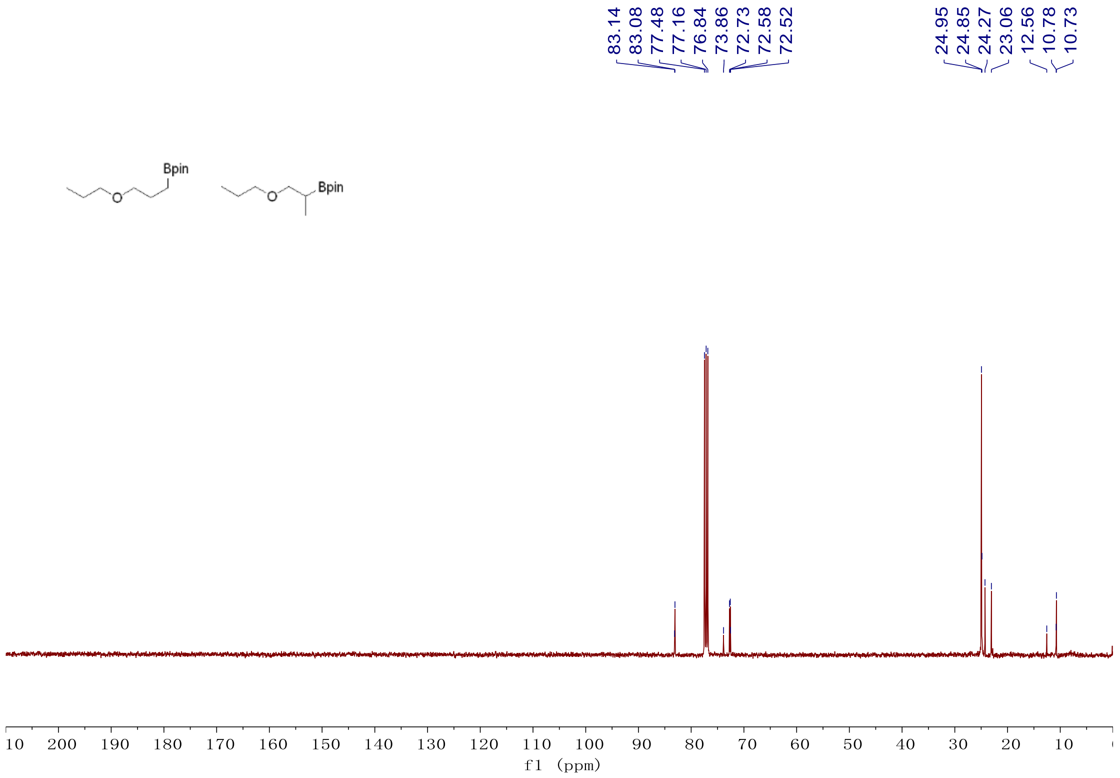

^{1}H NMR (400 MHz, $CDCl_3$) of **33** (*see procedure*):

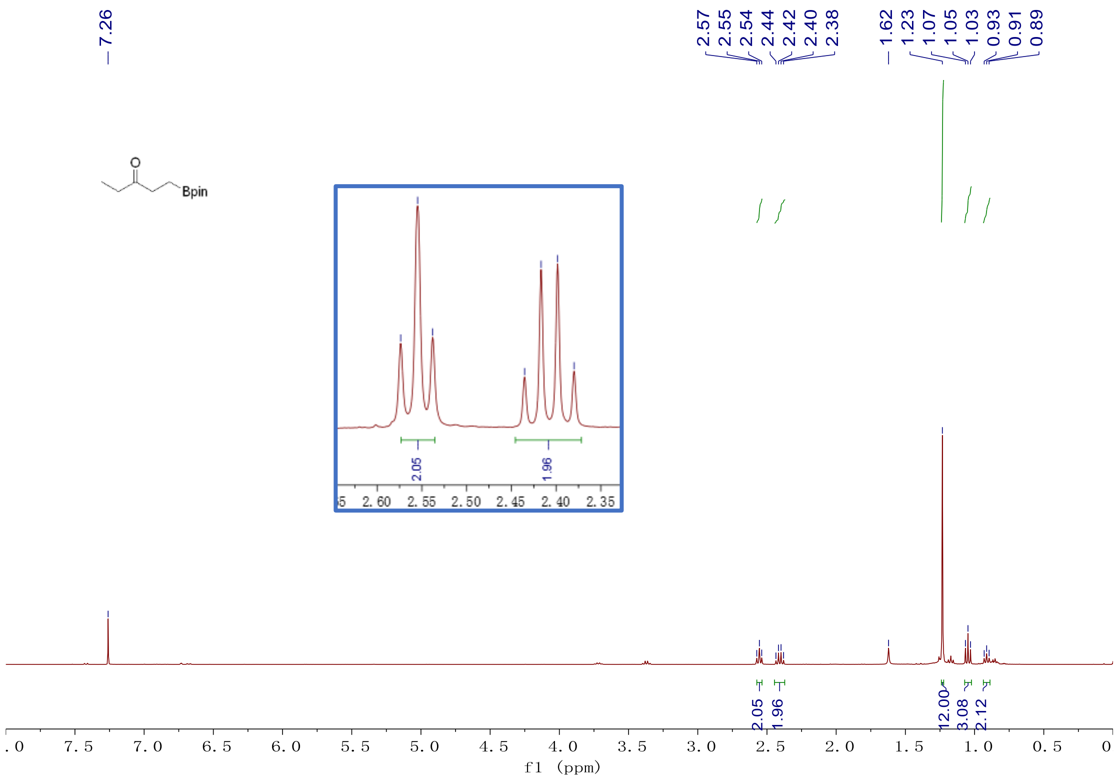

^{13}C NMR (101 MHz, $CDCl_3$) of **33** (*see procedure*):

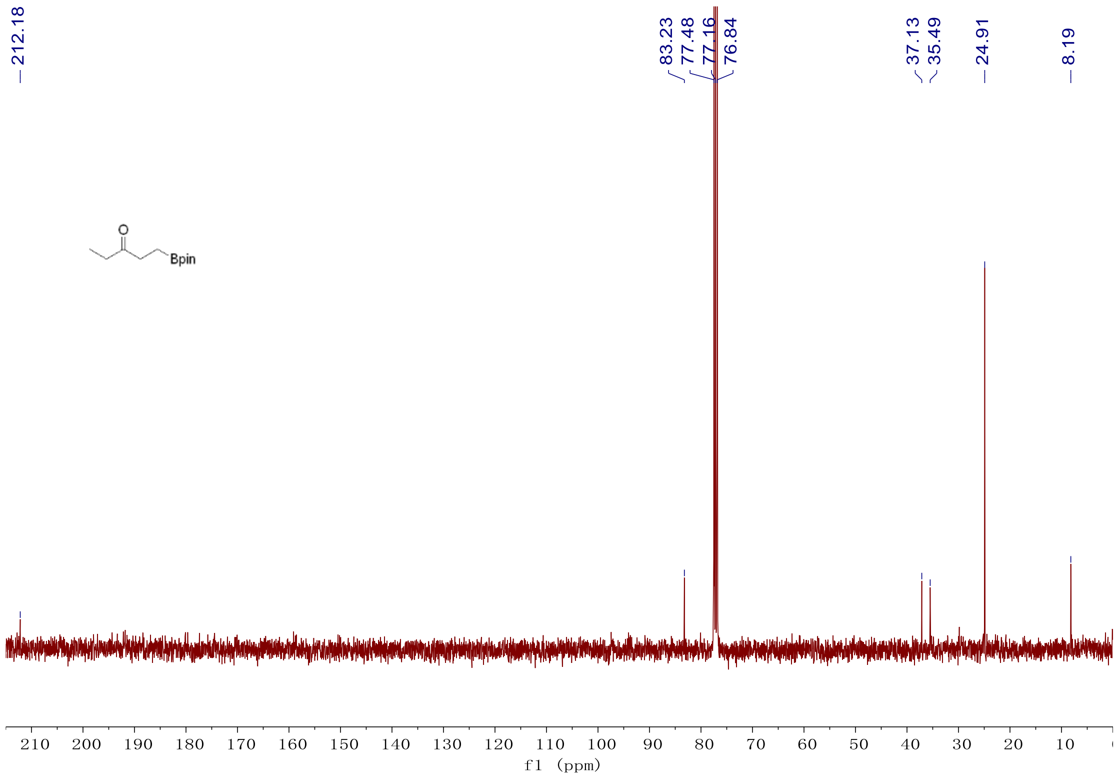

^{1}H NMR (400 MHz, $CDCl_3$) of **34** (*see procedure*):

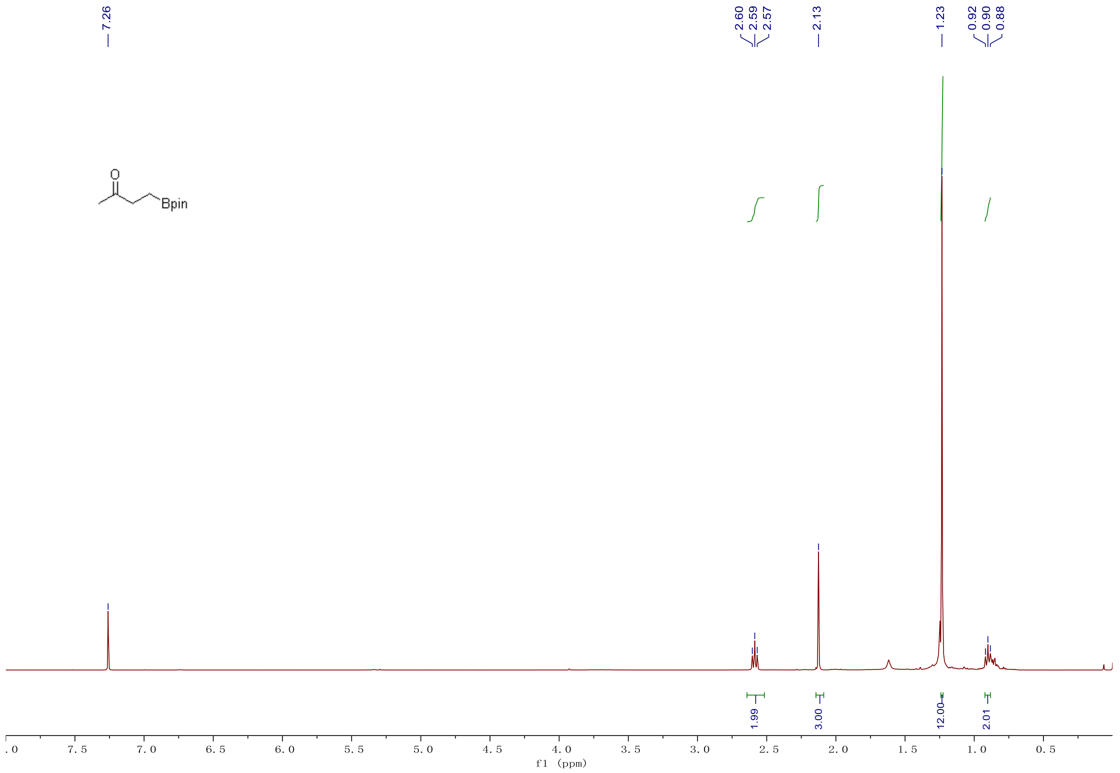

^{13}C NMR (101 MHz, $CDCl_3$) of **34** (*see procedure*):

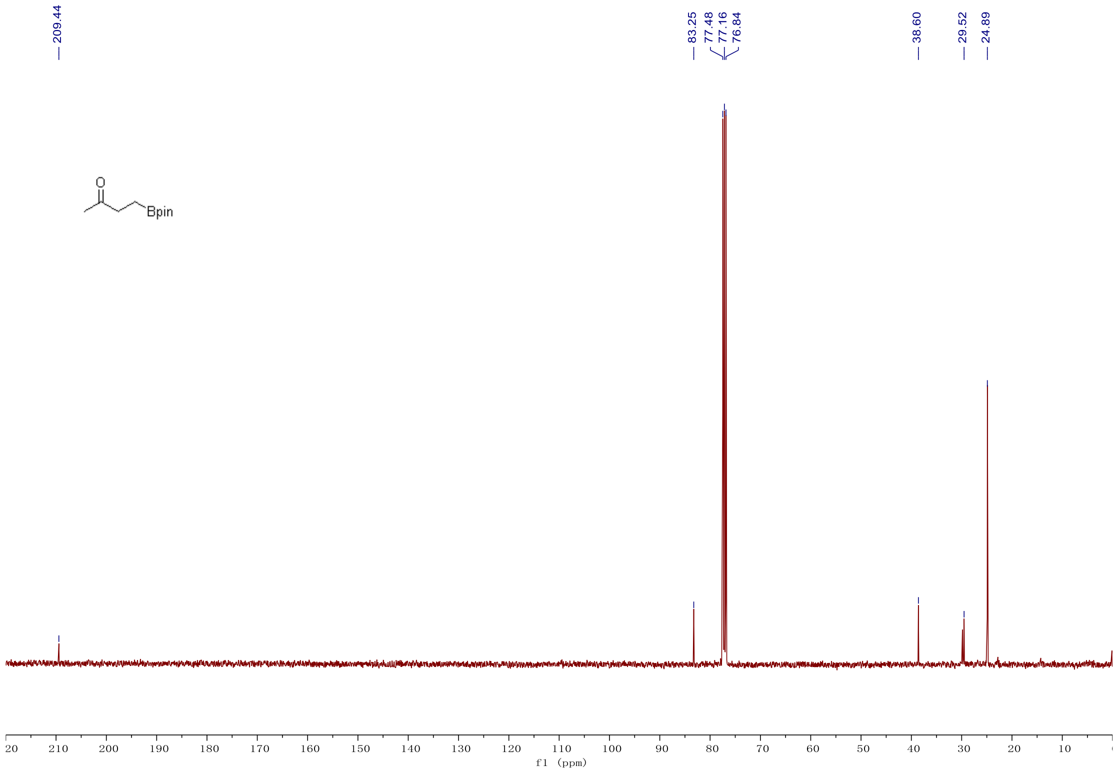

^{1}H NMR (400 MHz, $CDCl_3$) of **35** (*see procedure*):

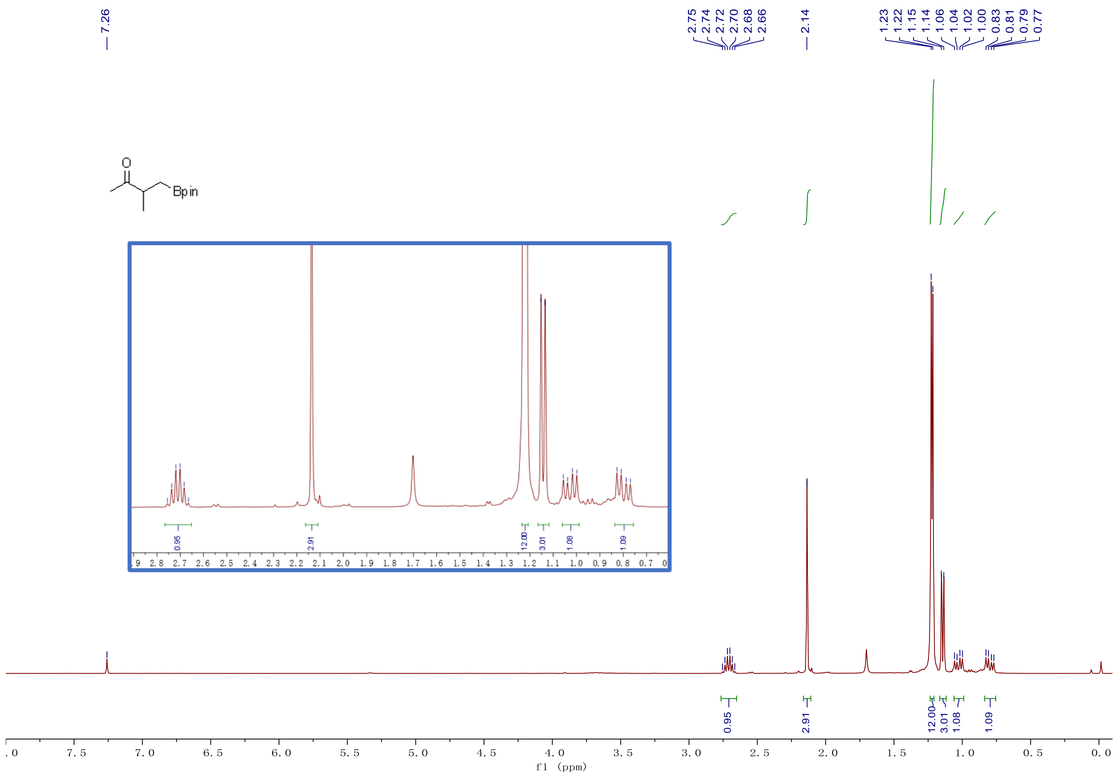

^{13}C NMR (101 MHz, $CDCl_3$) of **35** (*see procedure*):

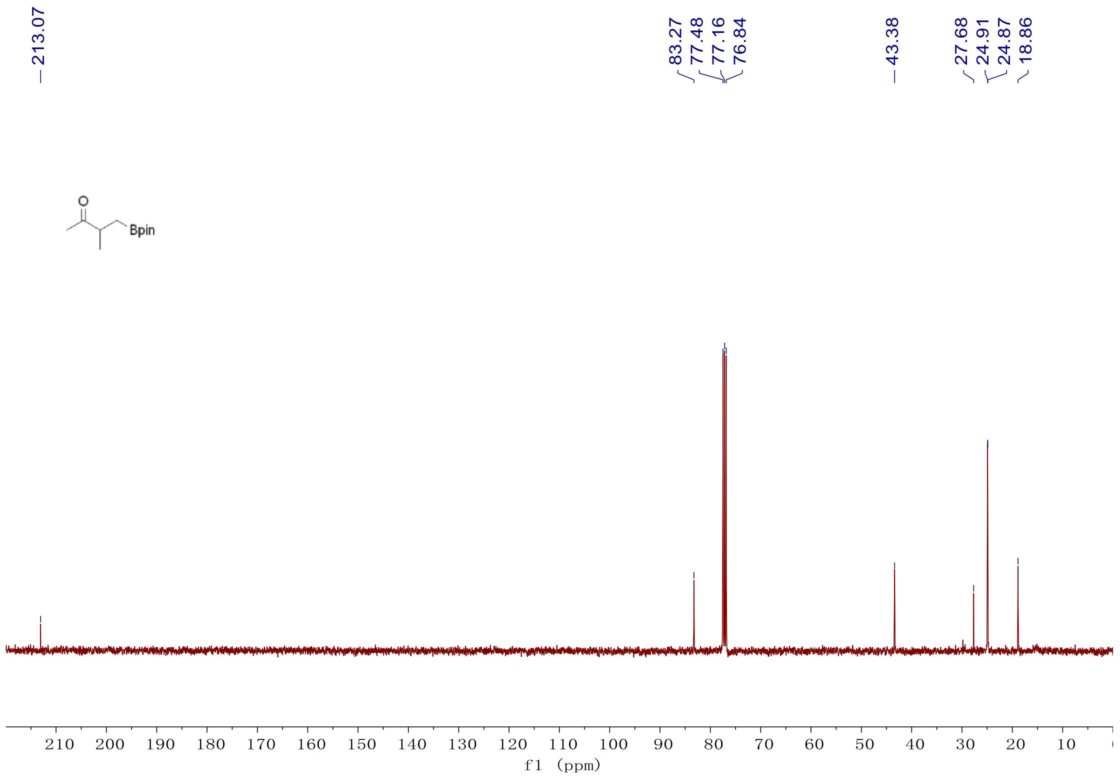

^{1}H NMR (400 MHz, $CDCl_3$) of **36** (*see procedure*):

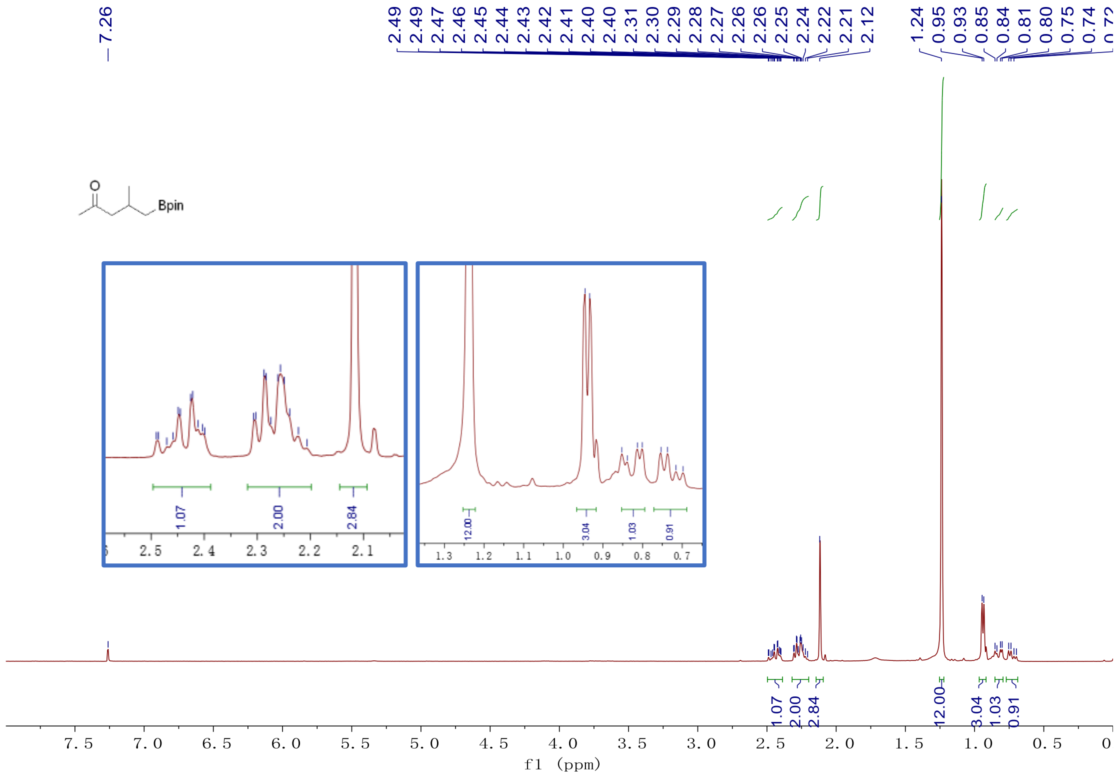

^{13}C NMR (101 MHz, $CDCl_3$) of **36** (*see procedure*):

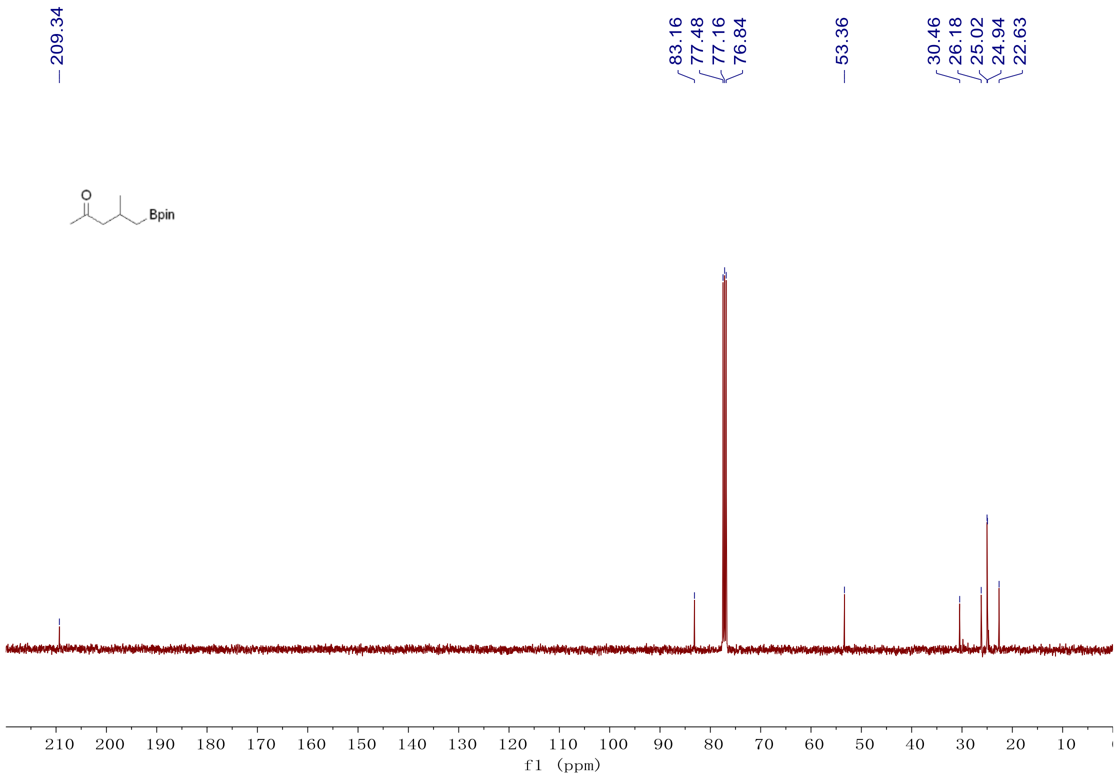

^{1}H NMR (400 MHz, $CDCl_3$) of **37** (*see procedure*):

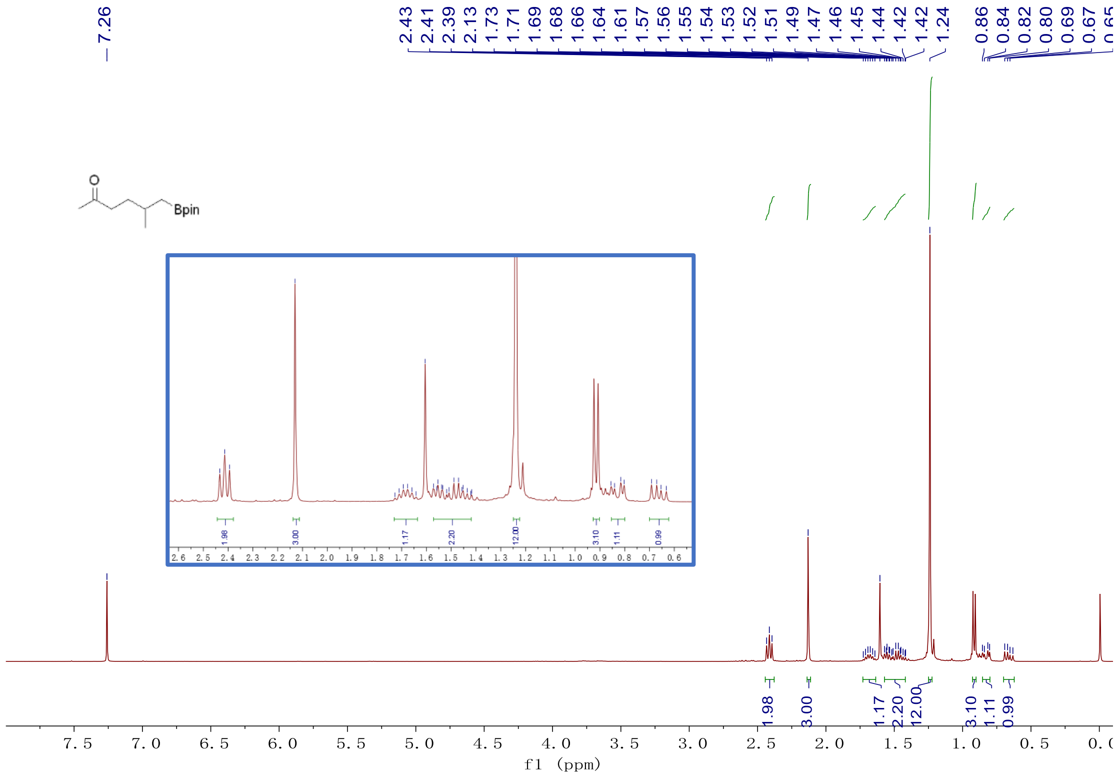

^{13}C NMR (101 MHz, $CDCl_3$) of **37** (*see procedure*):

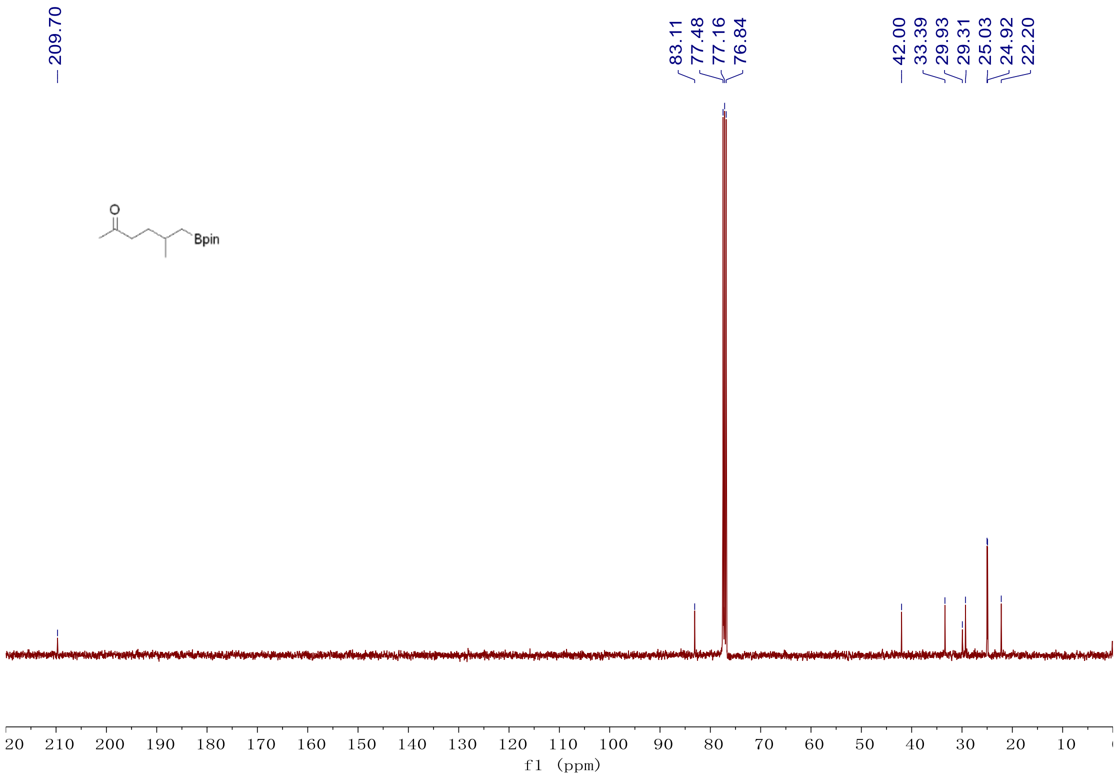

^{1}H NMR (400 MHz, $CDCl_3$) of **38** (*see procedure*):

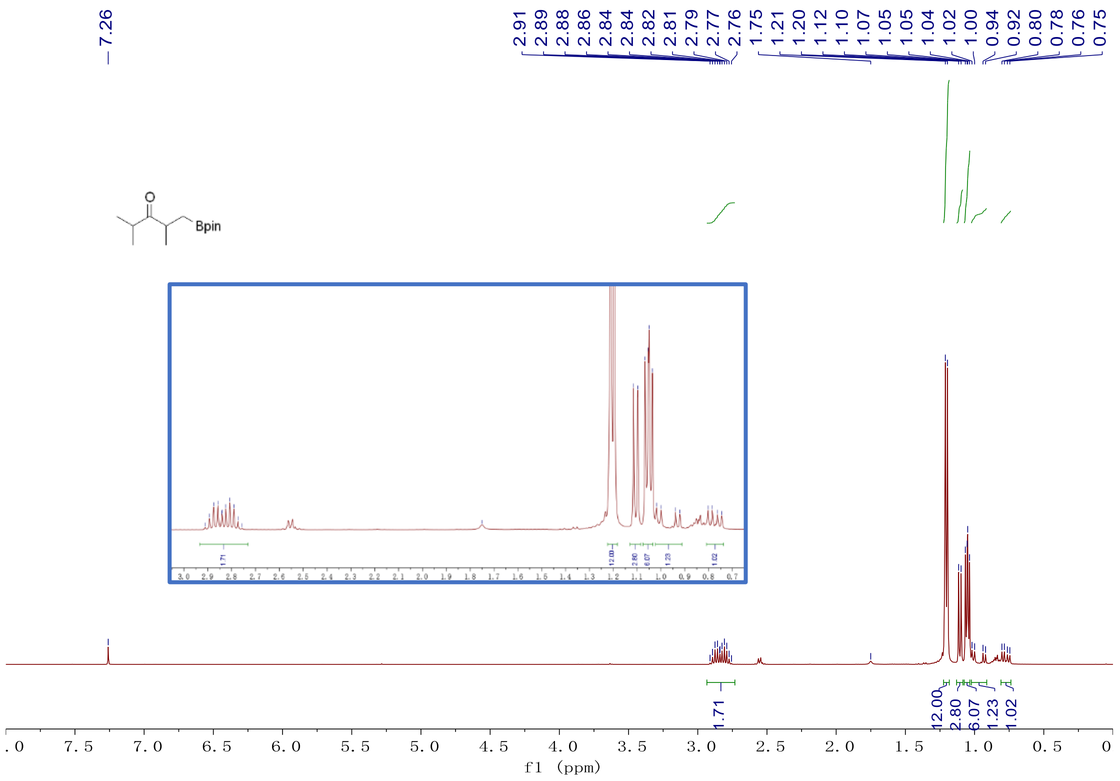

^{13}C NMR (101 MHz, $CDCl_3$) of **38** (*see procedure*):

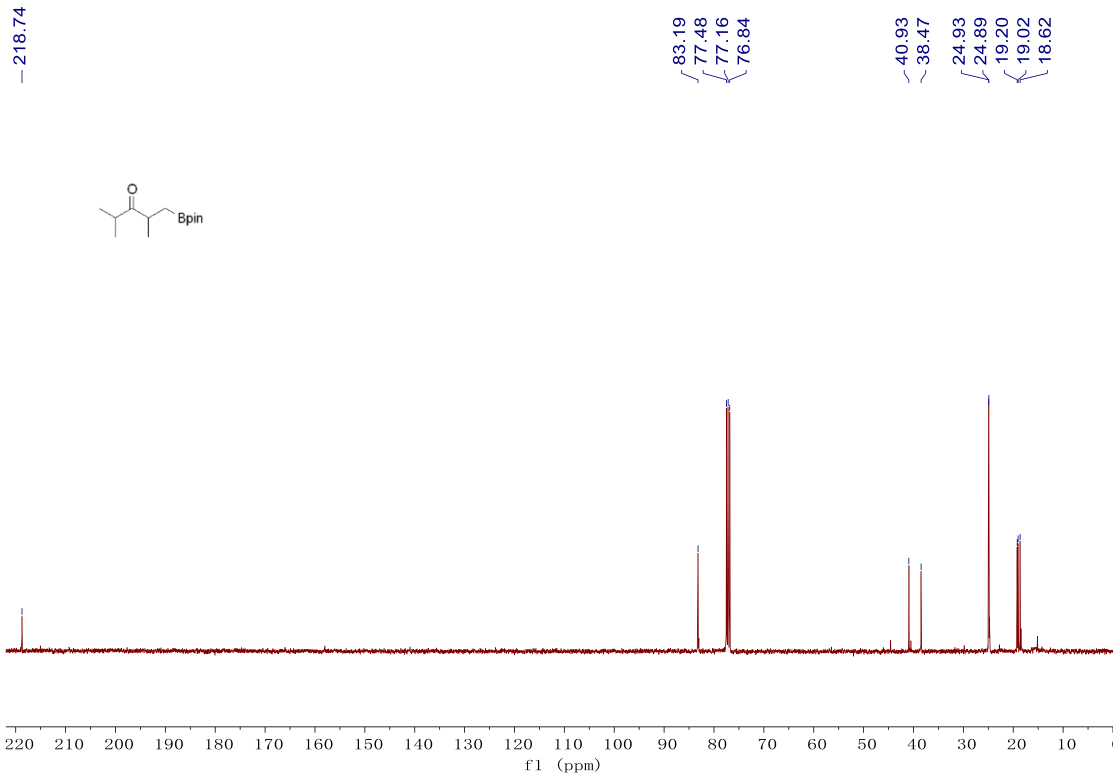

^{1}H NMR (400 MHz, CDCl$_3$) of **39** (*see procedure*):

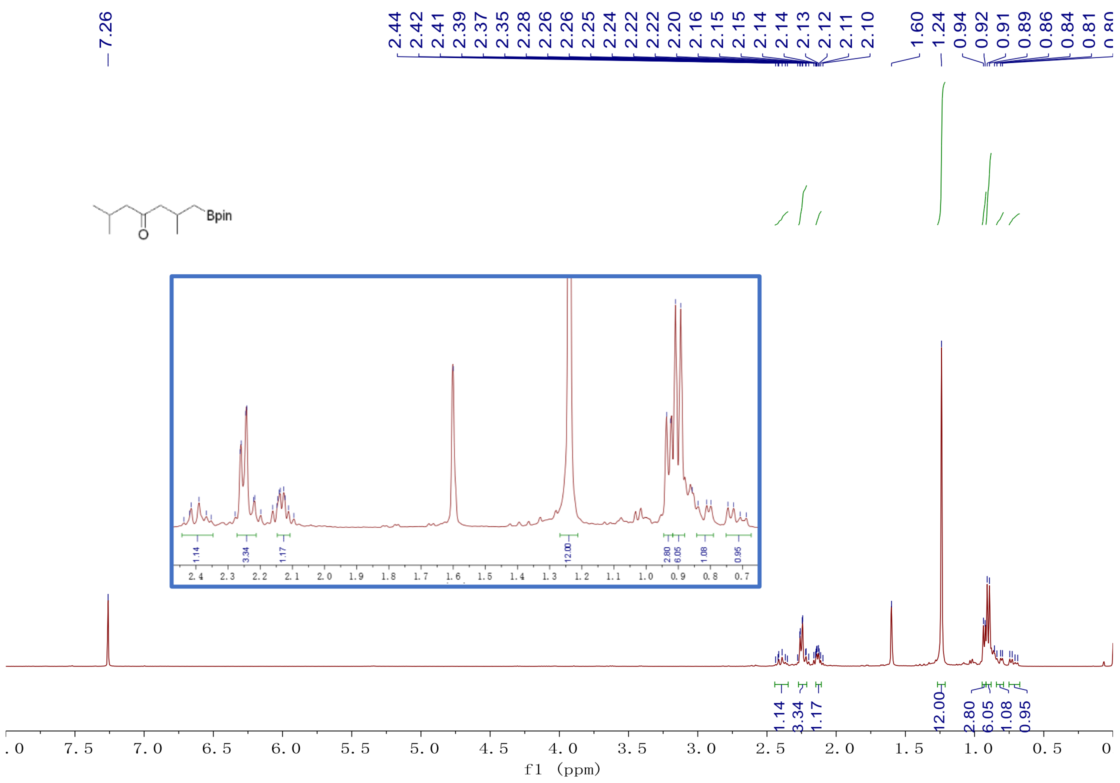

^{13}C NMR (101 MHz, CDCl$_3$) of **39** (*see procedure*):

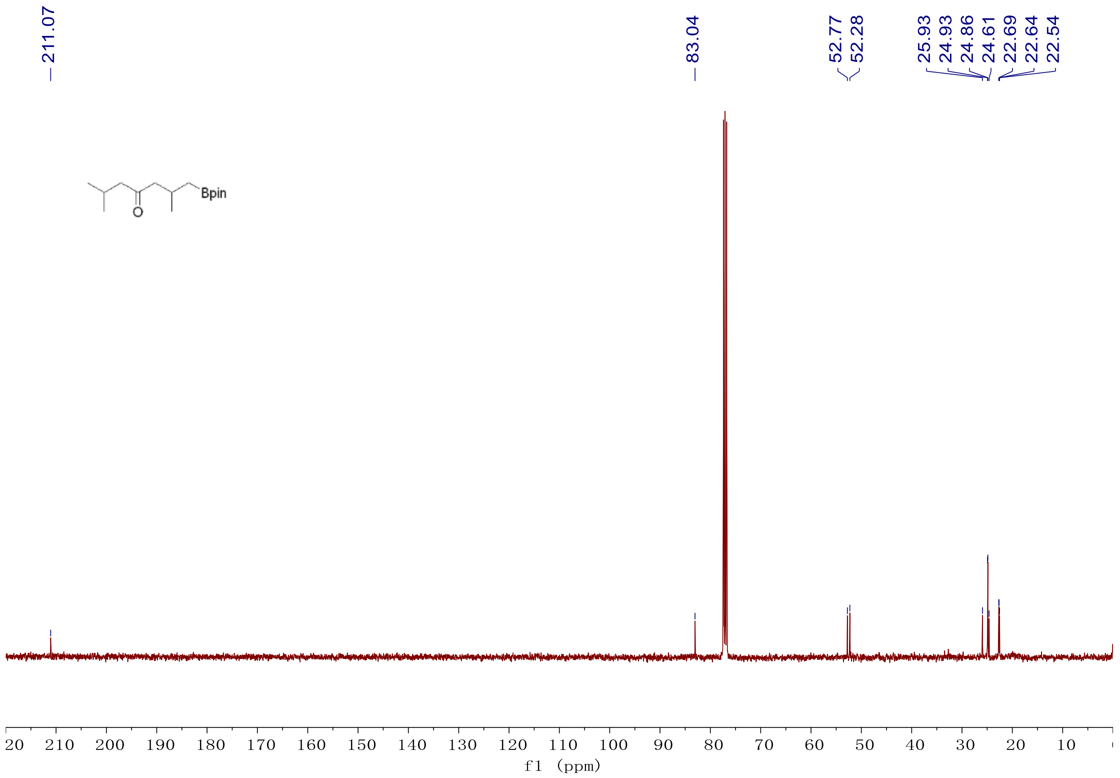

^{1}H NMR (400 MHz, $CDCl_3$) of **40-β** (*see procedure*):

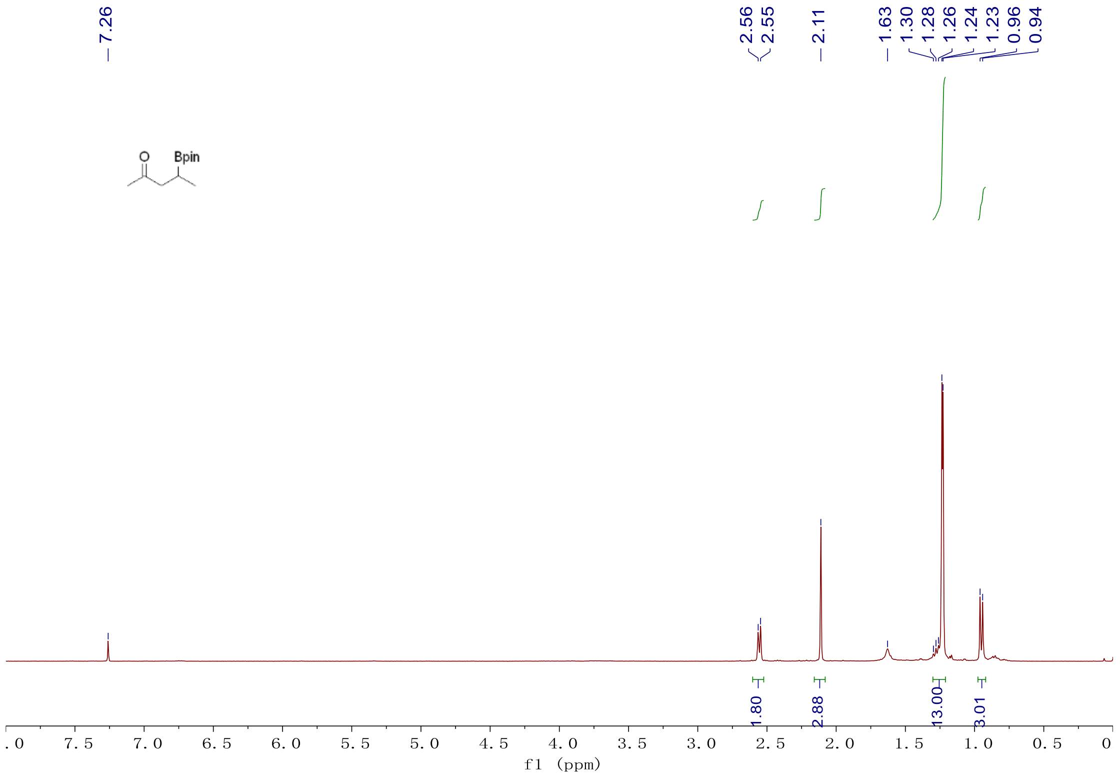

^{13}C NMR (101 MHz, $CDCl_3$) of **40-β** (*see procedure*):

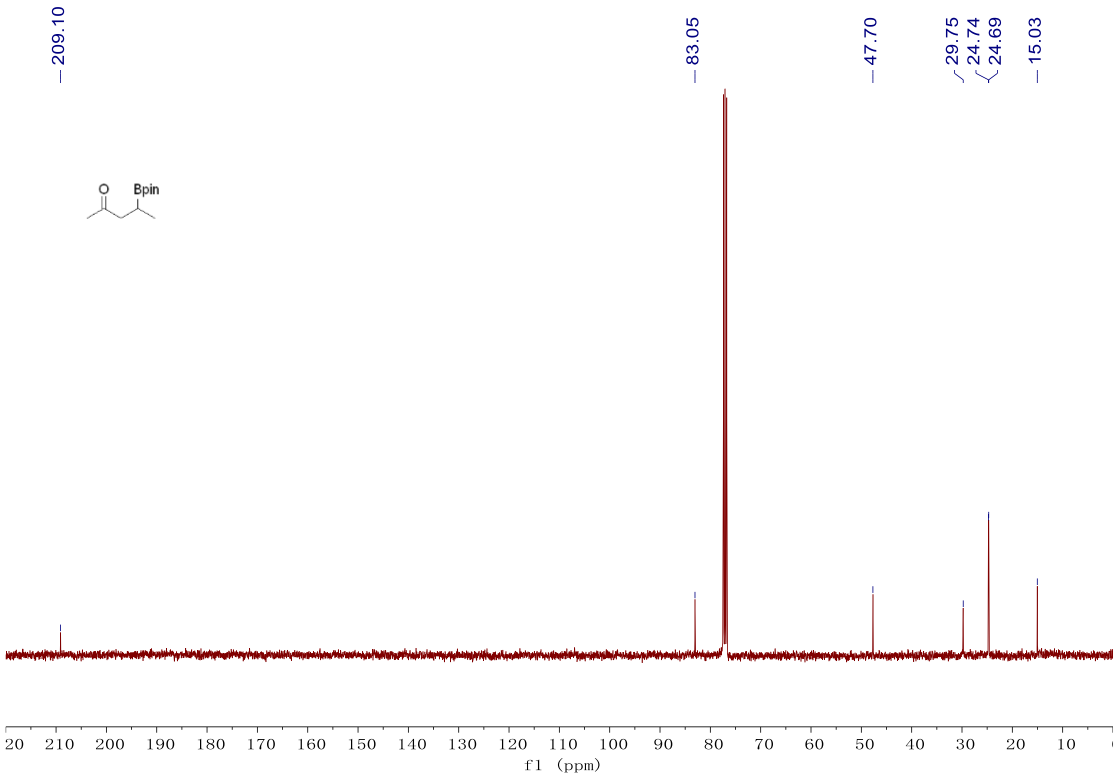

^{1}H NMR (400 MHz, $CDCl_3$) of **40-γ** (*see procedure*):

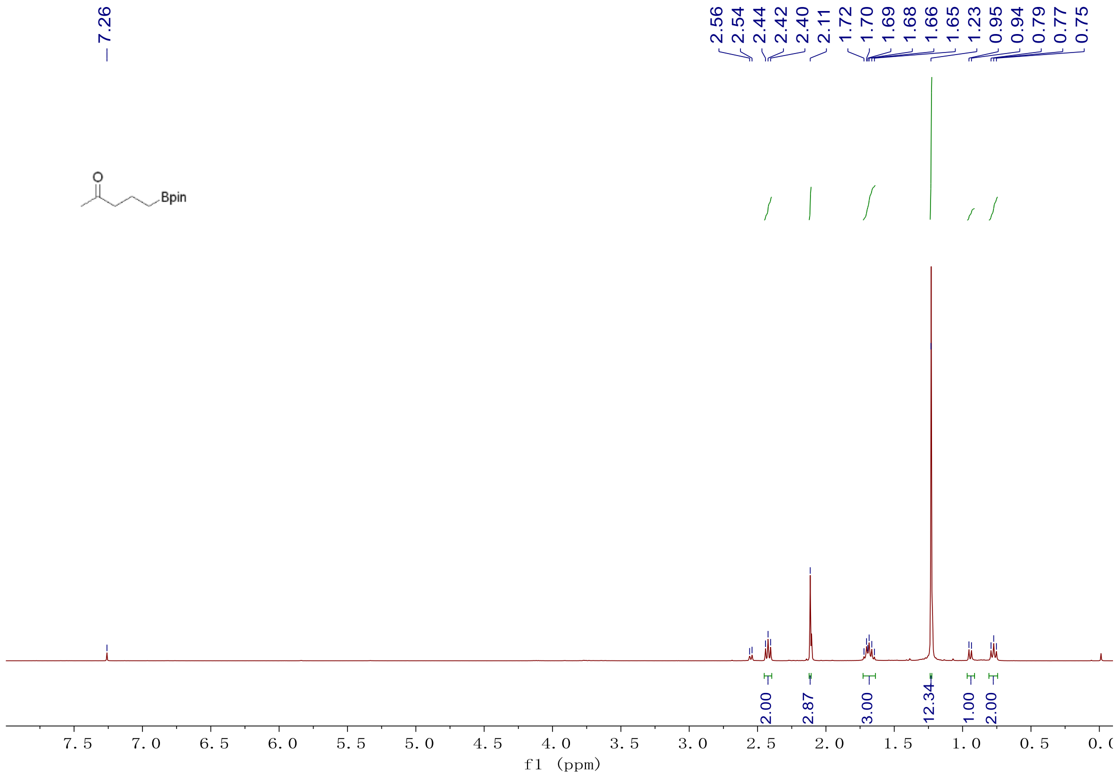

^{13}C NMR (101 MHz, $CDCl_3$) of **40-γ** (*see procedure*):

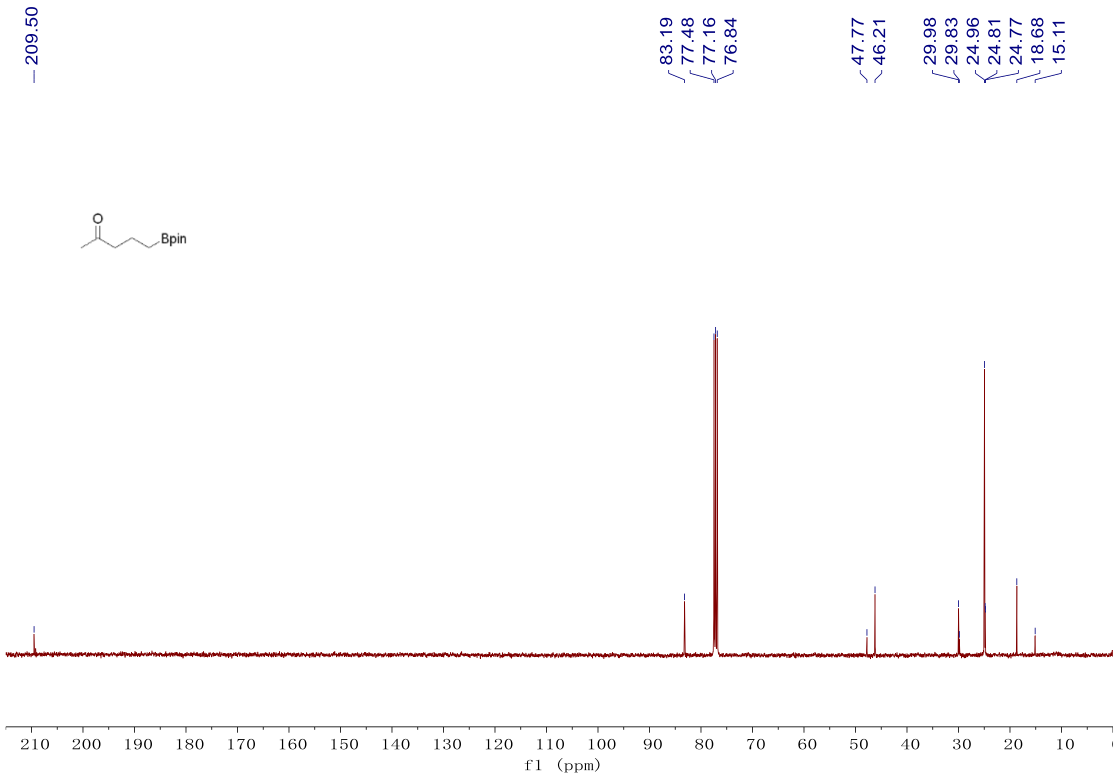

^{1}H NMR (400 MHz, $CDCl_3$) of **41-β** (*see procedure*):

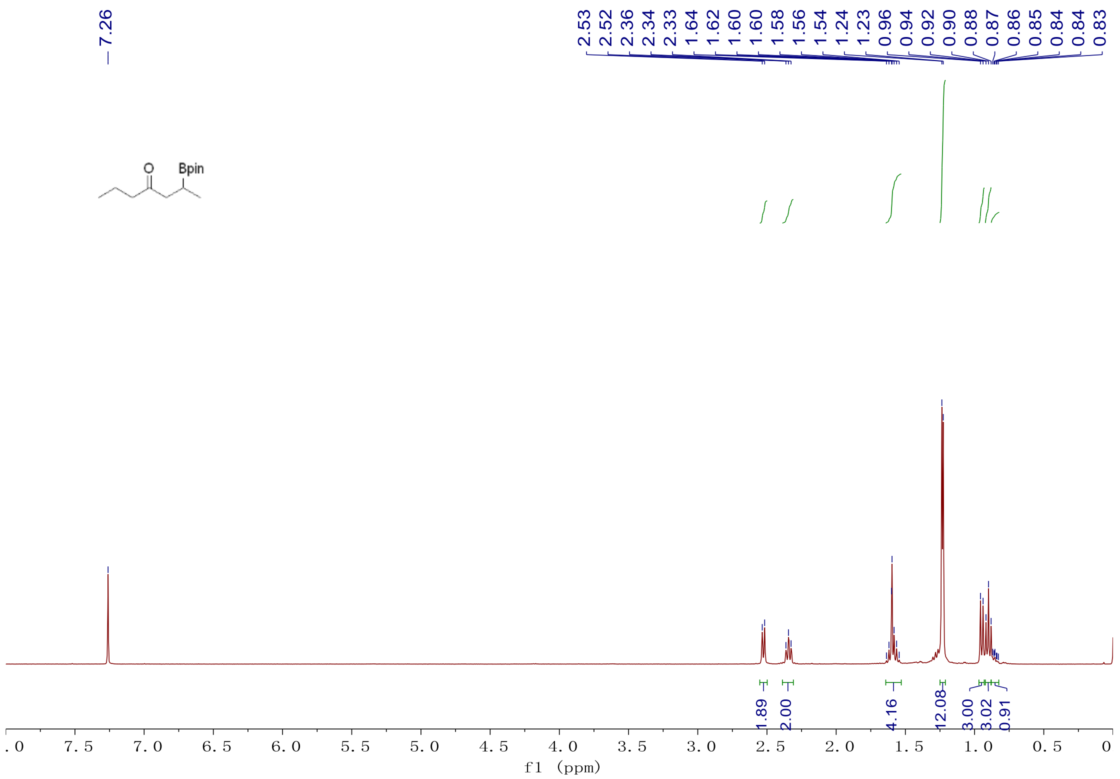

^{13}C NMR (101 MHz, $CDCl_3$) of **41-β** (*see procedure*):

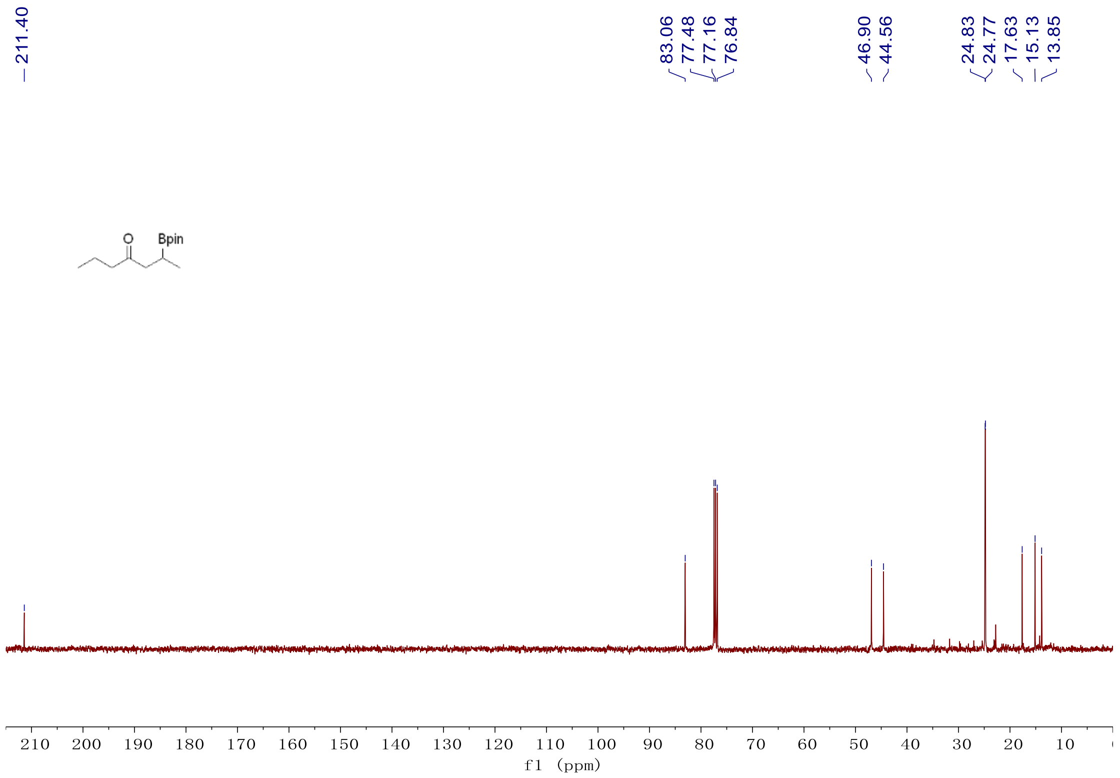

^{1}H NMR (400 MHz, $CDCl_3$) of **41-γ** (see procedure):

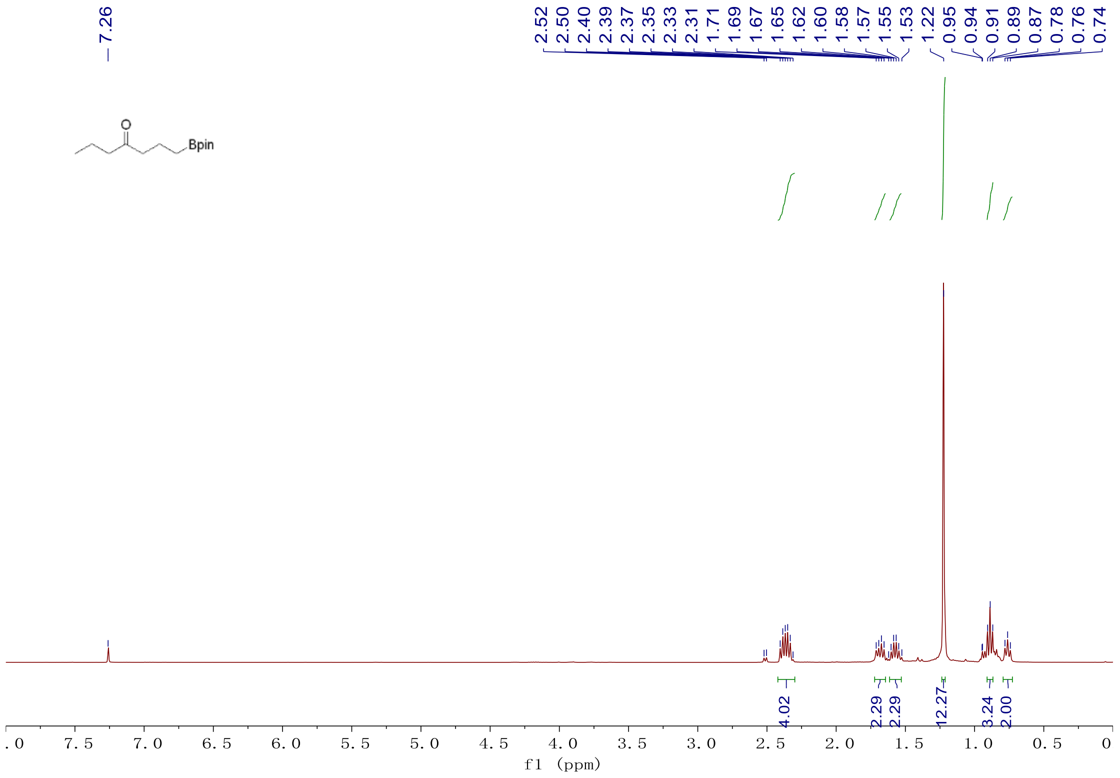

^{13}C NMR (101 MHz, $CDCl_3$) of **41-γ** (see procedure):

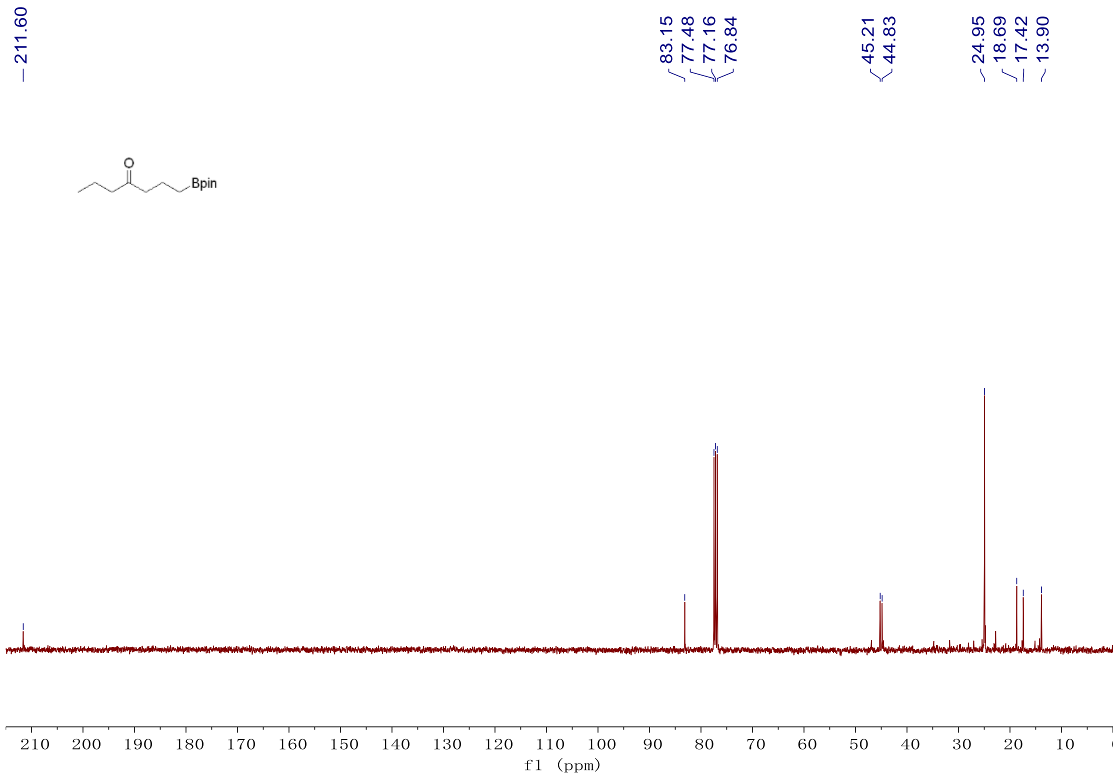

^{1}H NMR (400 MHz, $CDCl_3$) of **42** (*see procedure*):

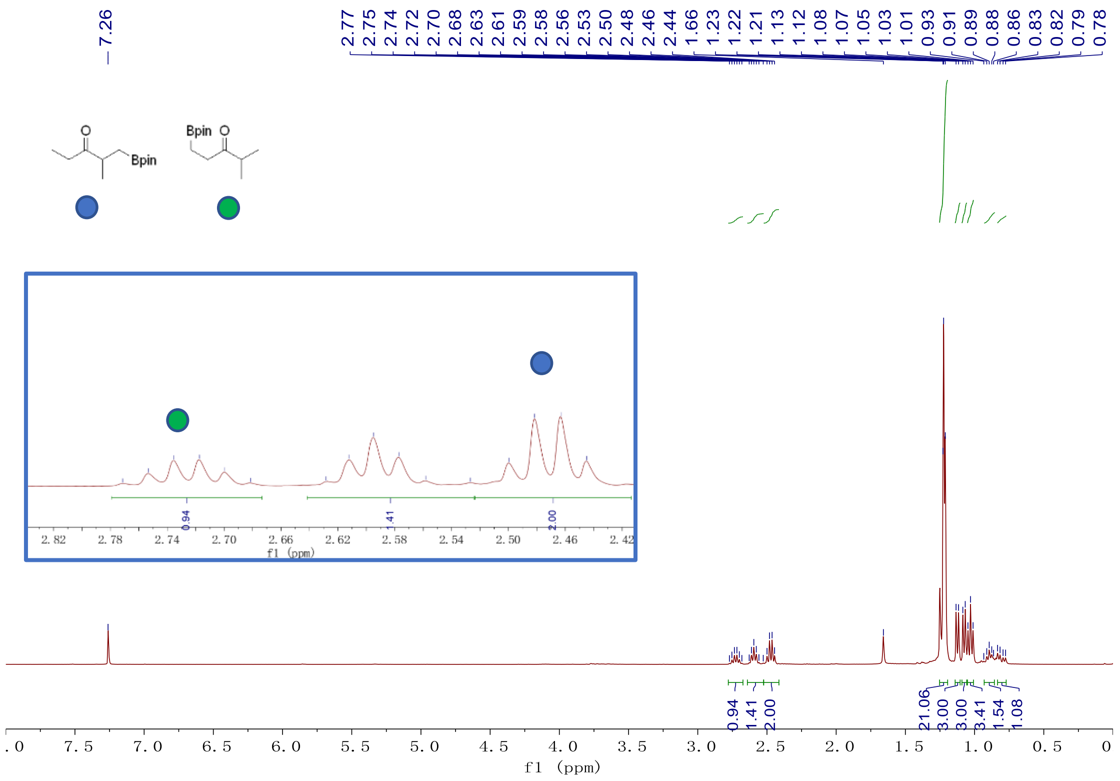

^{13}C NMR (101 MHz, $CDCl_3$) of **42** (*see procedure*):

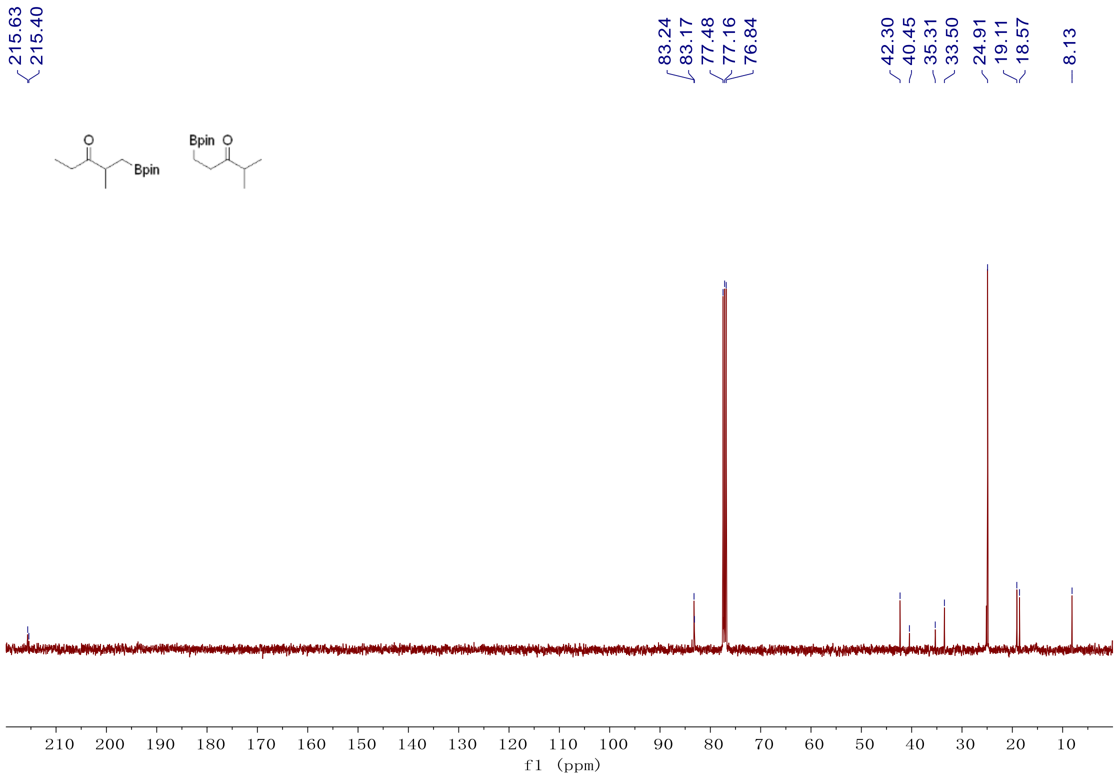

^{1}H NMR (400 MHz, $CDCl_3$) of **43** (*see procedure*):

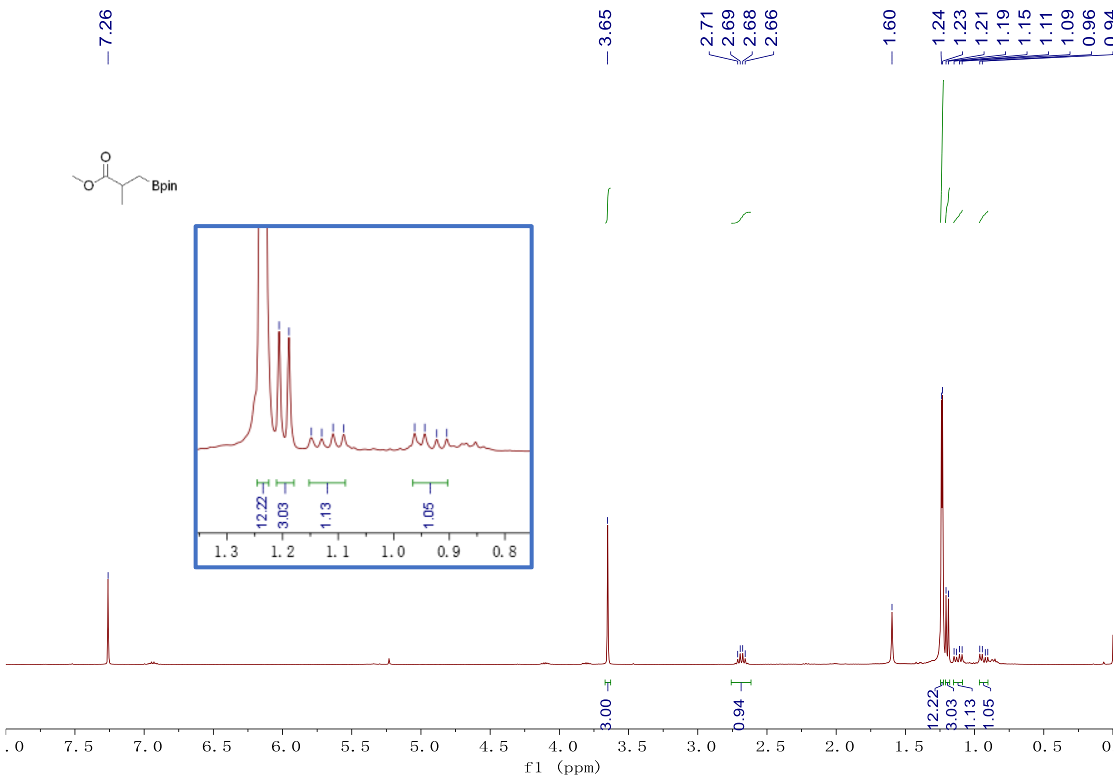

^{13}C NMR (101 MHz, $CDCl_3$) of **43** (*see procedure*):

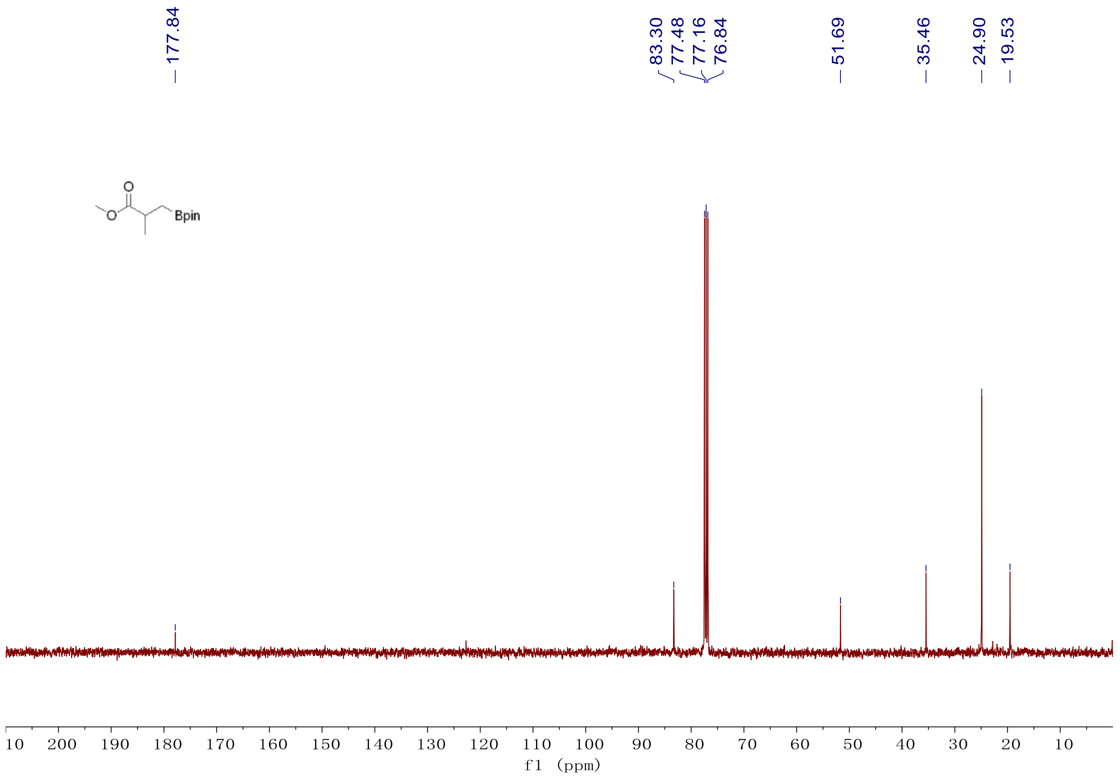

^{1}H NMR (400 MHz, $CDCl_3$) of **44** (*see procedure*):

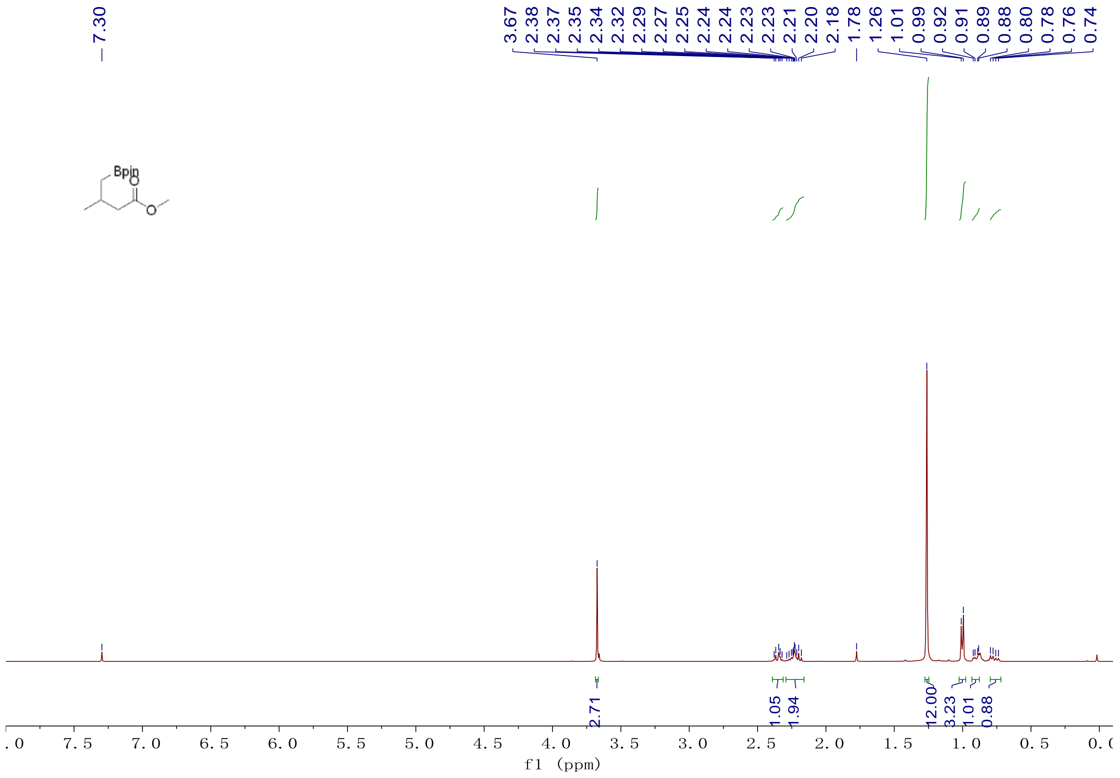

^{13}C NMR (101 MHz, $CDCl_3$) of **44** (*see procedure*):

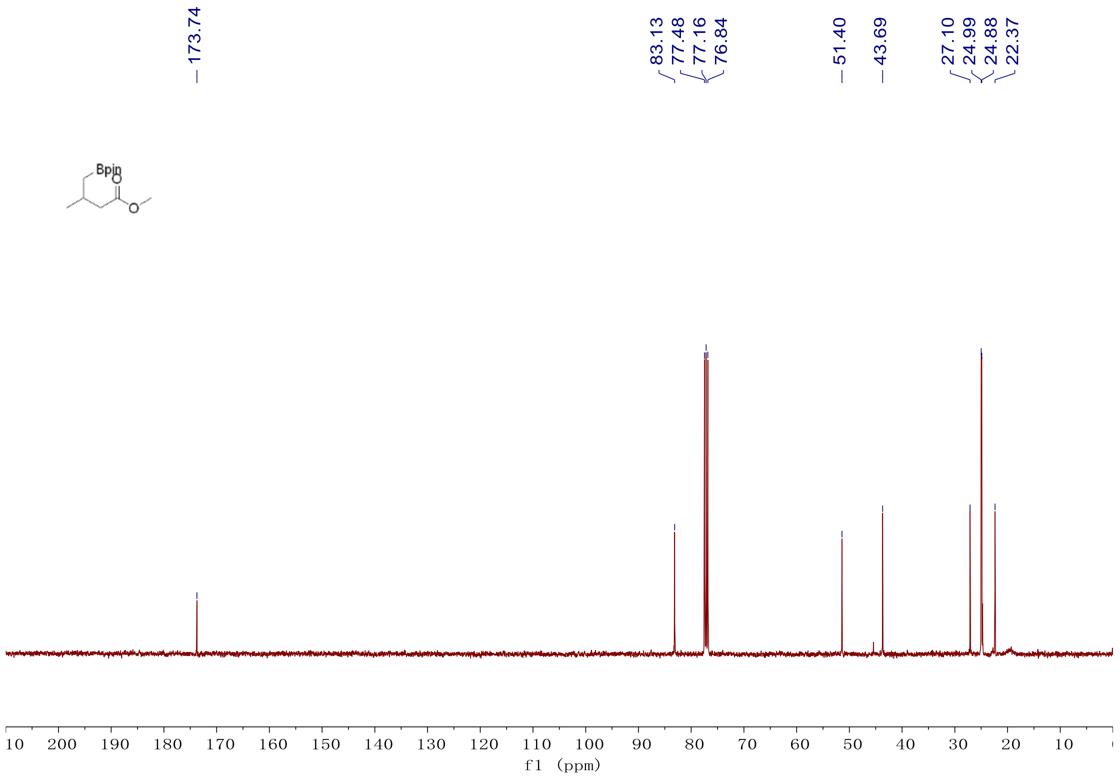

^{1}H NMR (400 MHz, $CDCl_3$) of **45** (*see procedure*):

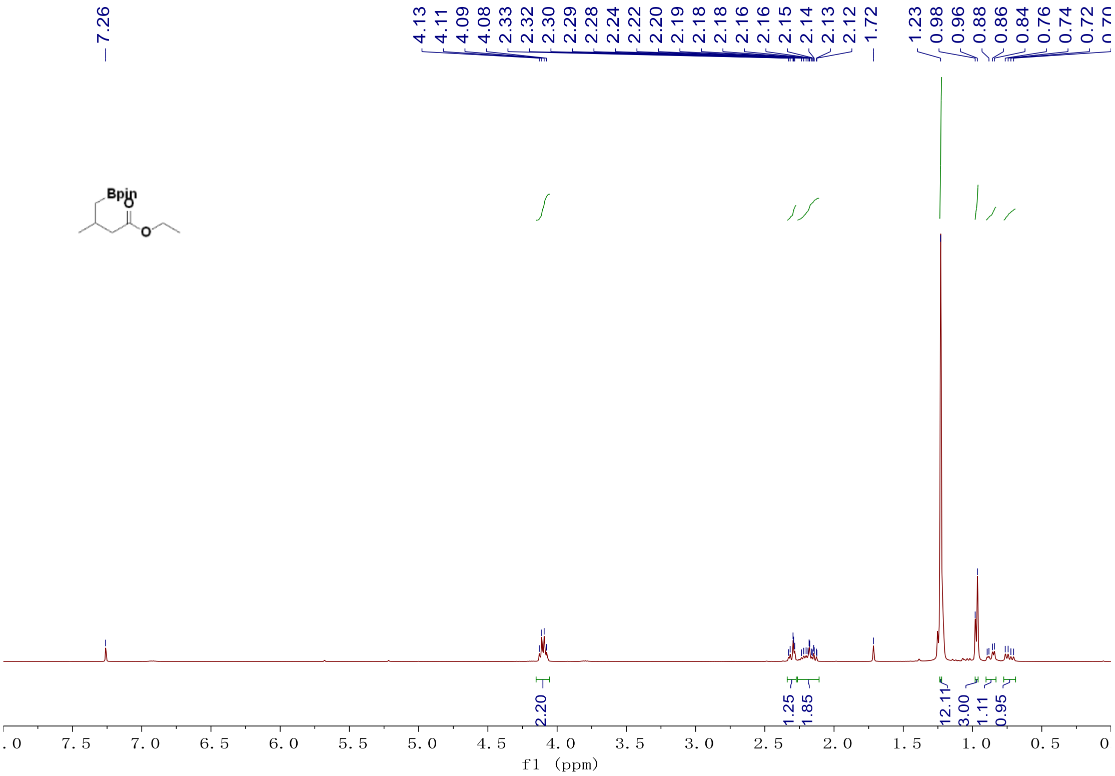

^{13}C NMR (101 MHz, $CDCl_3$) of **45** (*see procedure*):

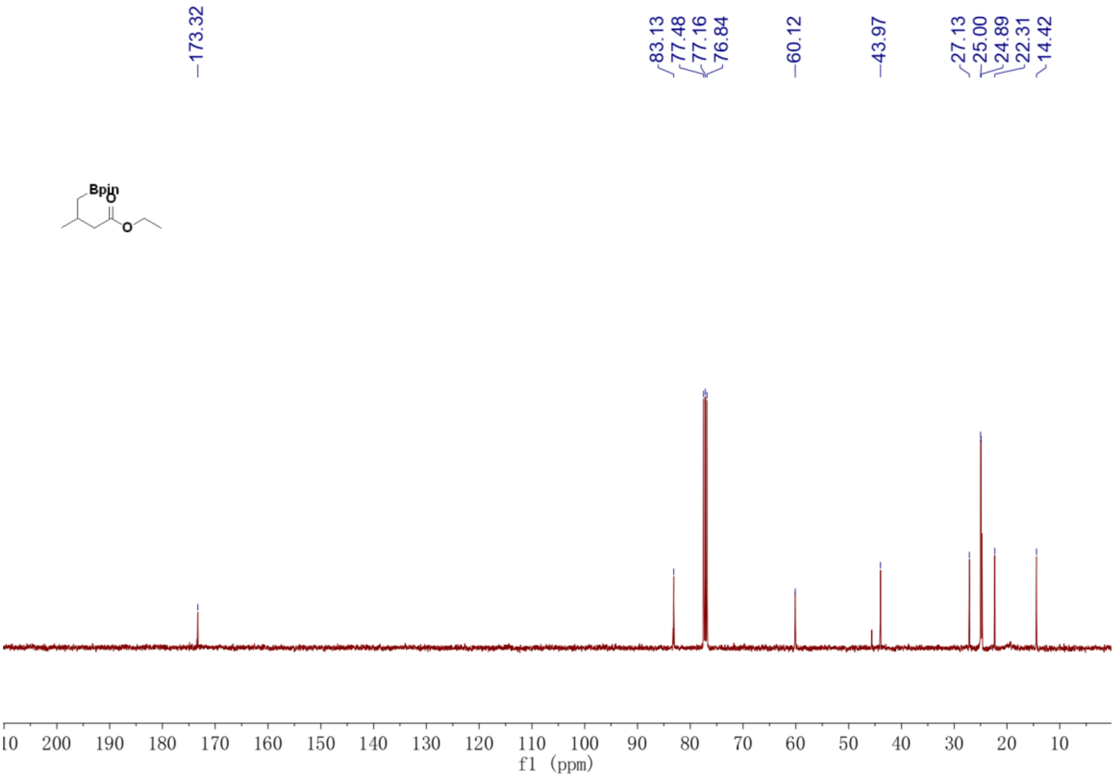

^{1}H NMR (400 MHz, $CDCl_3$) of **46** (*see procedure*):

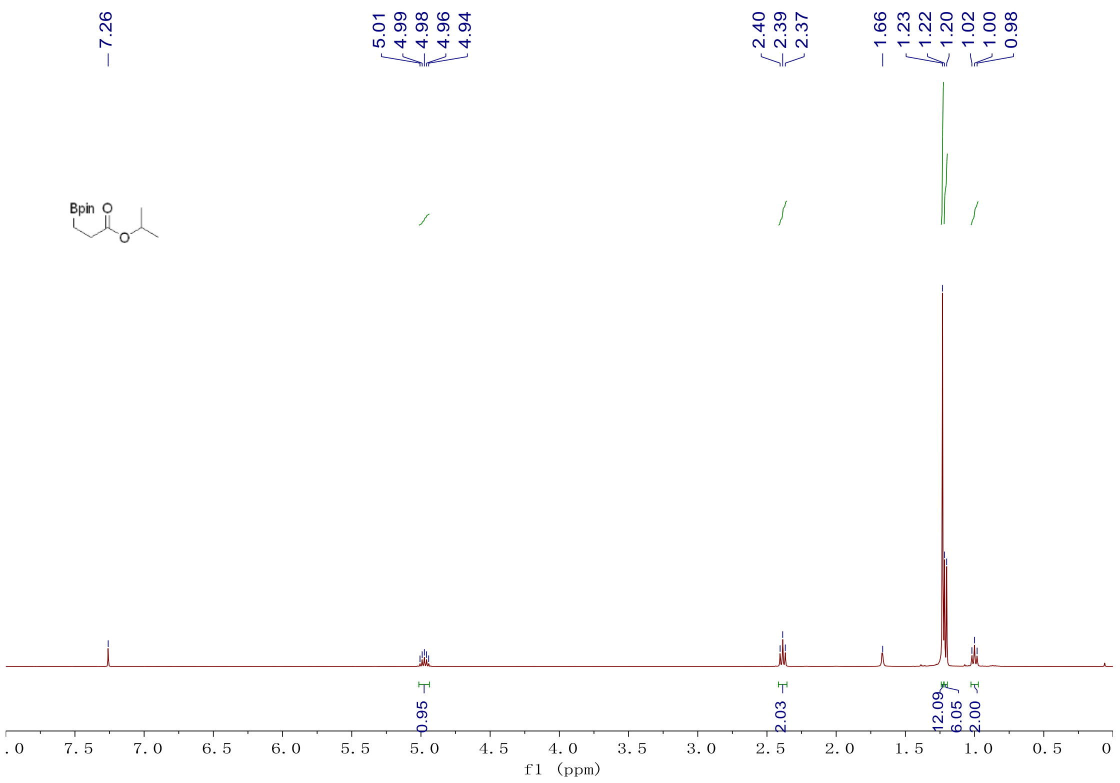

^{13}C NMR (101 MHz, $CDCl_3$) of **46** (*see procedure*):

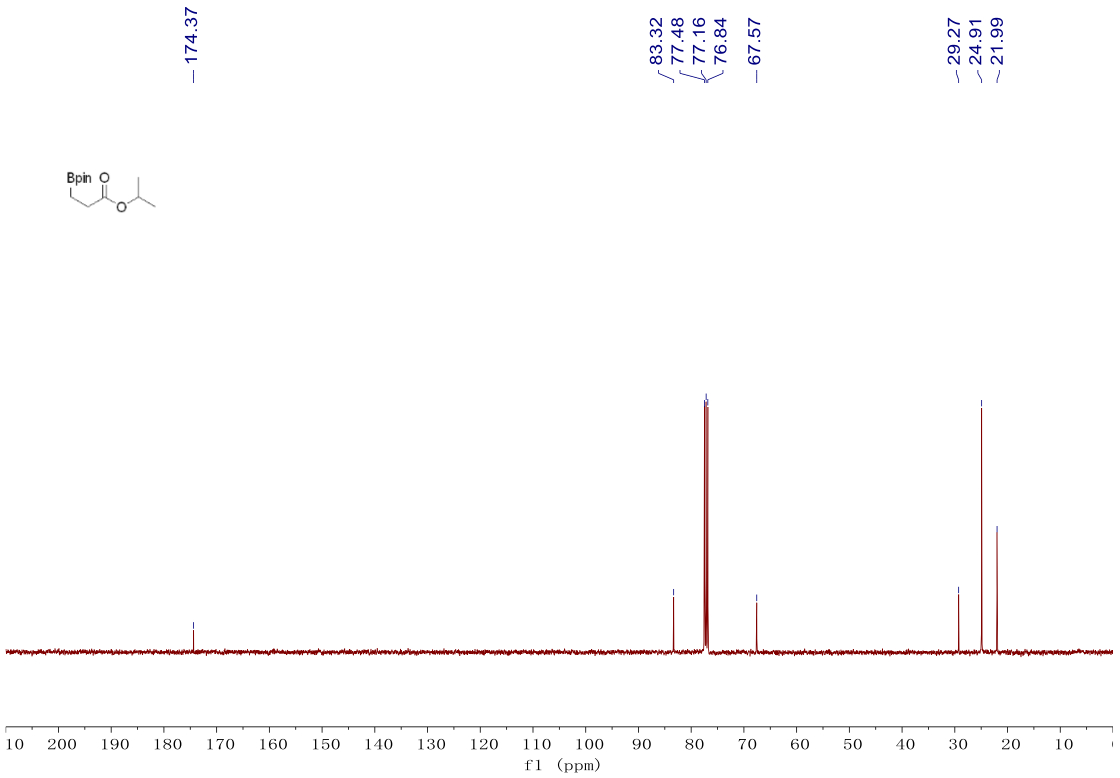

^{1}H NMR (400 MHz, $CDCl_3$) of **47** (*see procedure*):

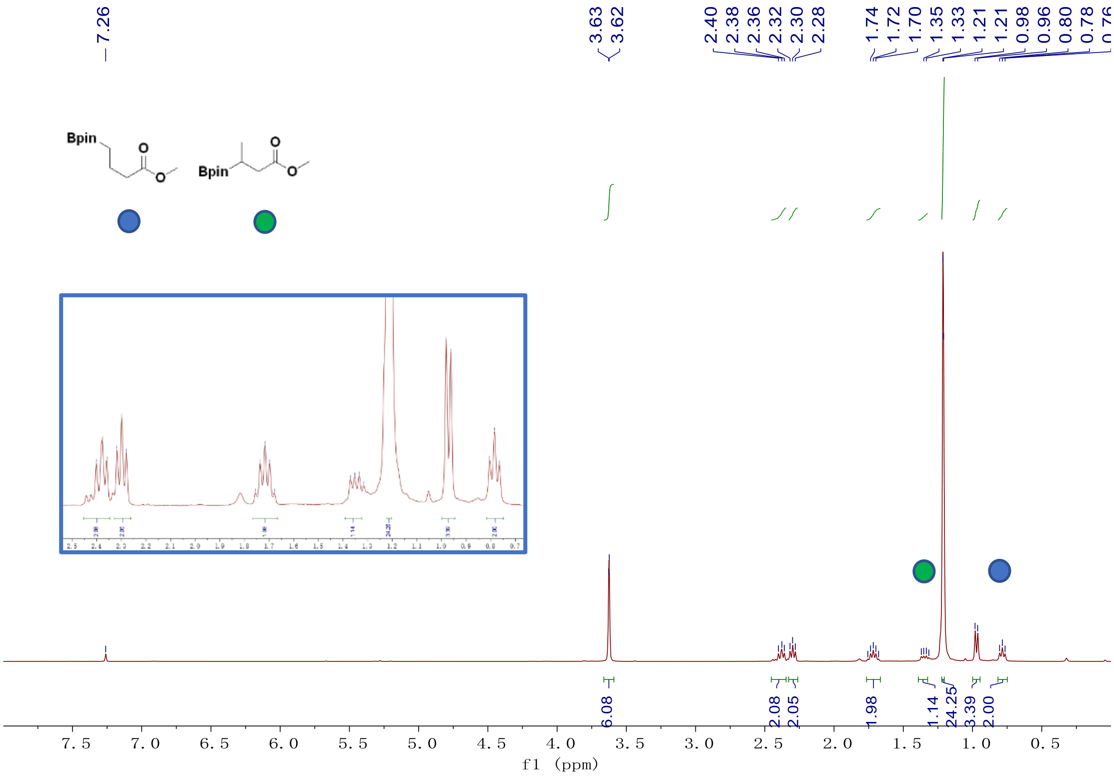

^{13}C NMR (101 MHz, $CDCl_3$) of **47** (*see procedure*):

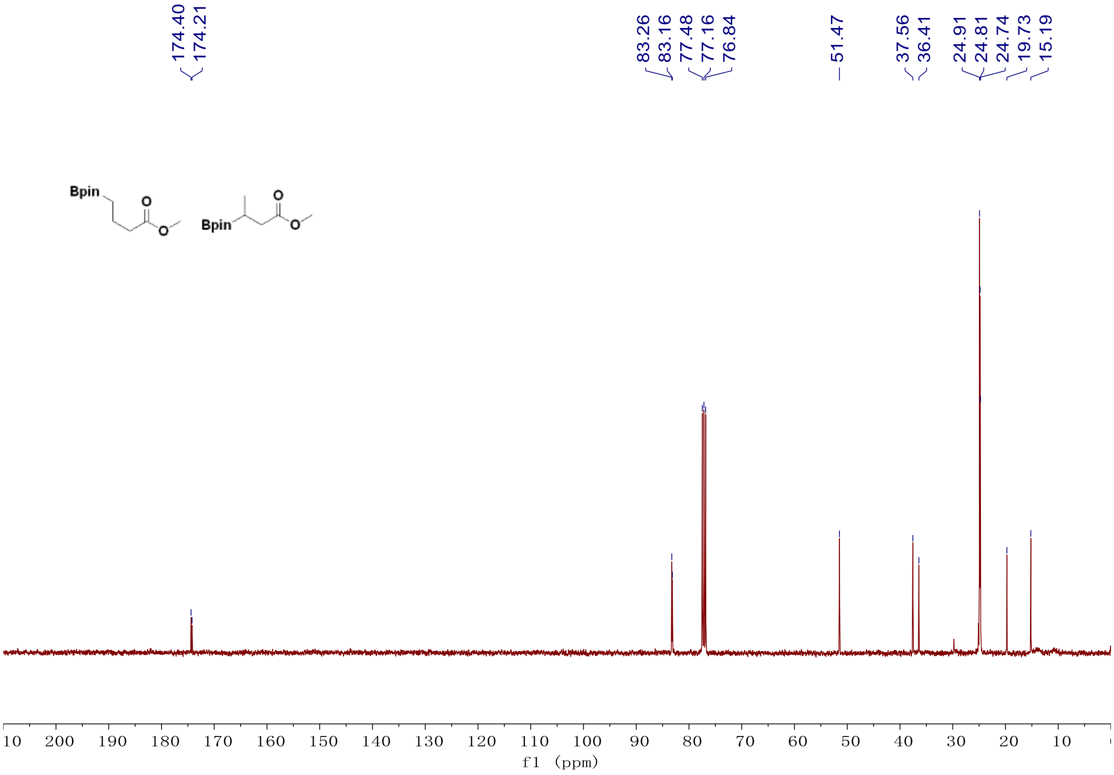

^{1}H NMR (400 MHz, $CDCl_3$) of **48-γ** (*see procedure*):

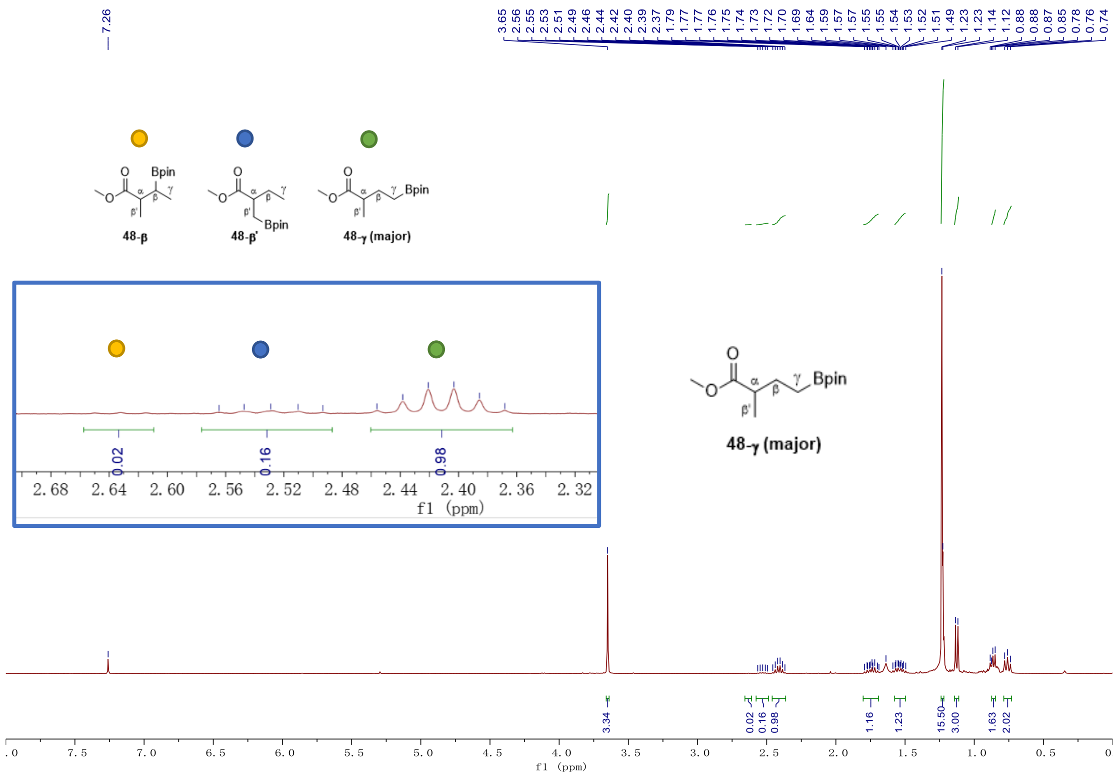

^{13}C NMR (101 MHz, $CDCl_3$) of **48-γ** (*see procedure*):

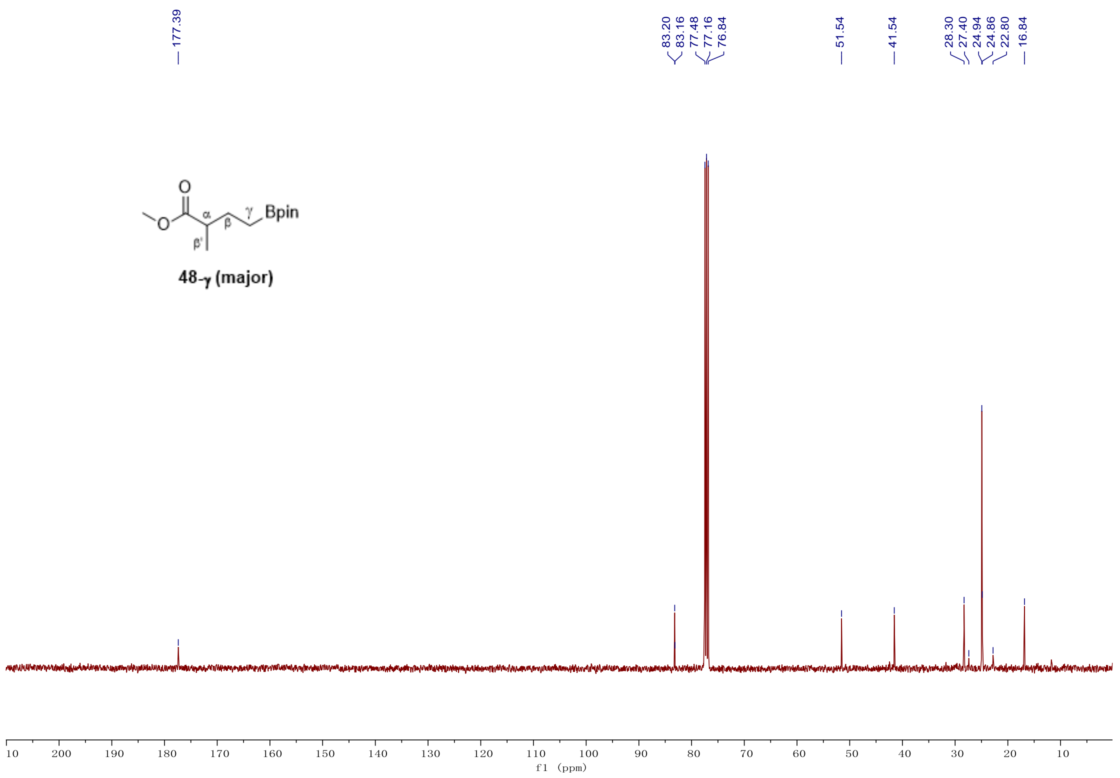

^{1}H NMR (400 MHz, $CDCl_3$) of **49** (*see procedure*):

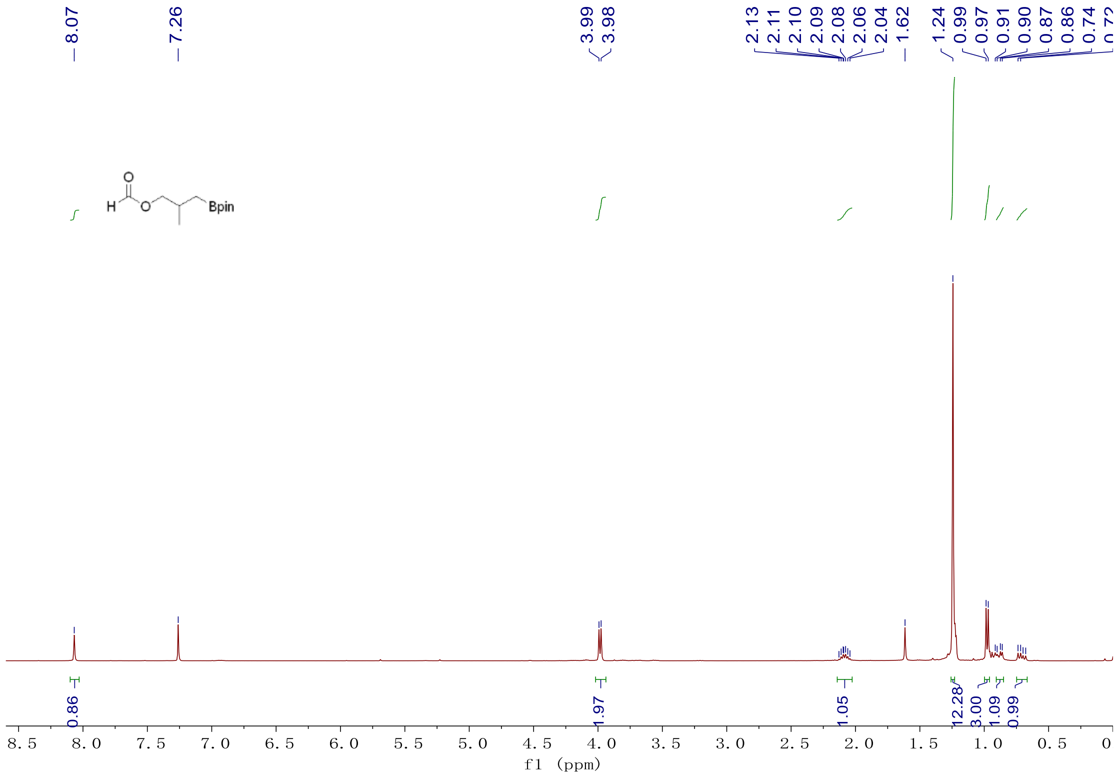

^{13}C NMR (101 MHz, $CDCl_3$) of **49** (*see procedure*):

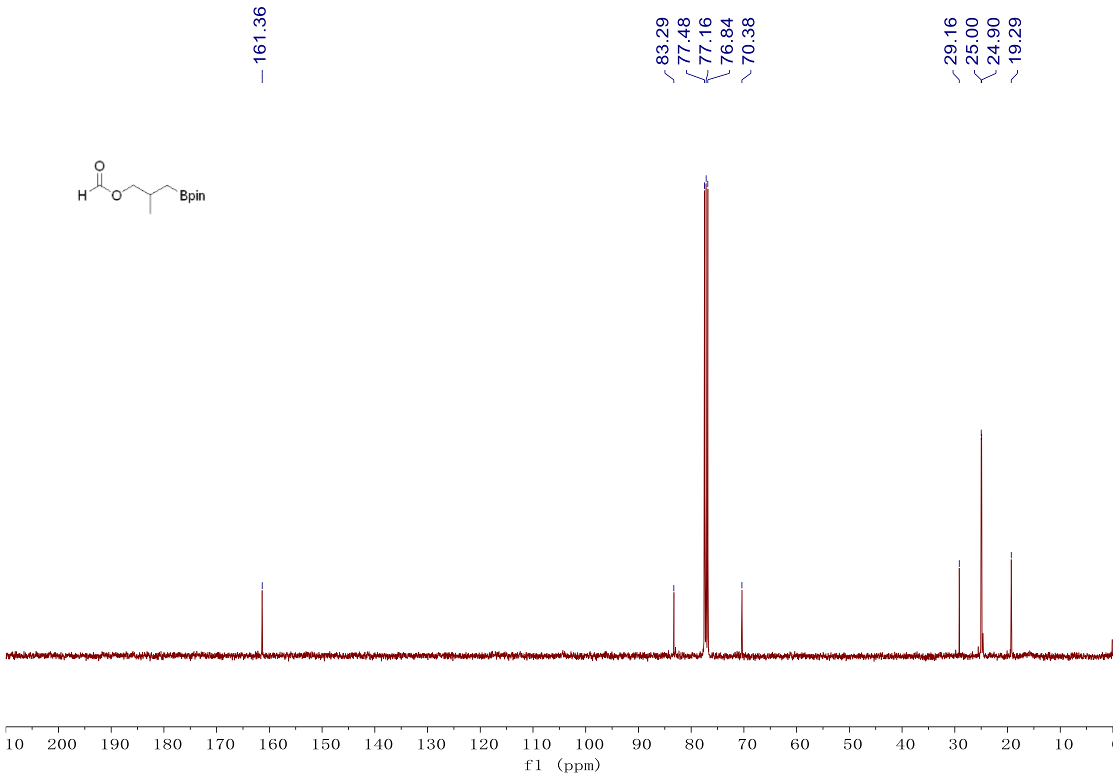

^{1}H NMR (400 MHz, $CDCl_3$) of **50** (*see procedure*):

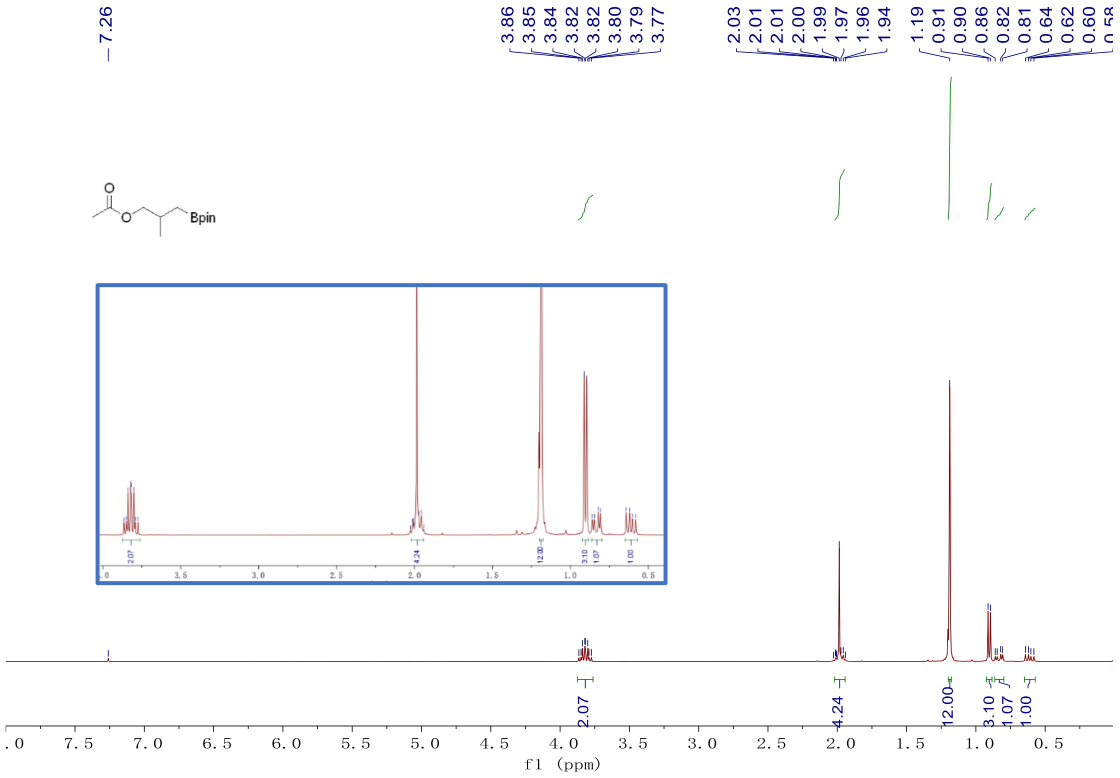

^{13}C NMR (101 MHz, $CDCl_3$) of **50** (*see procedure*):

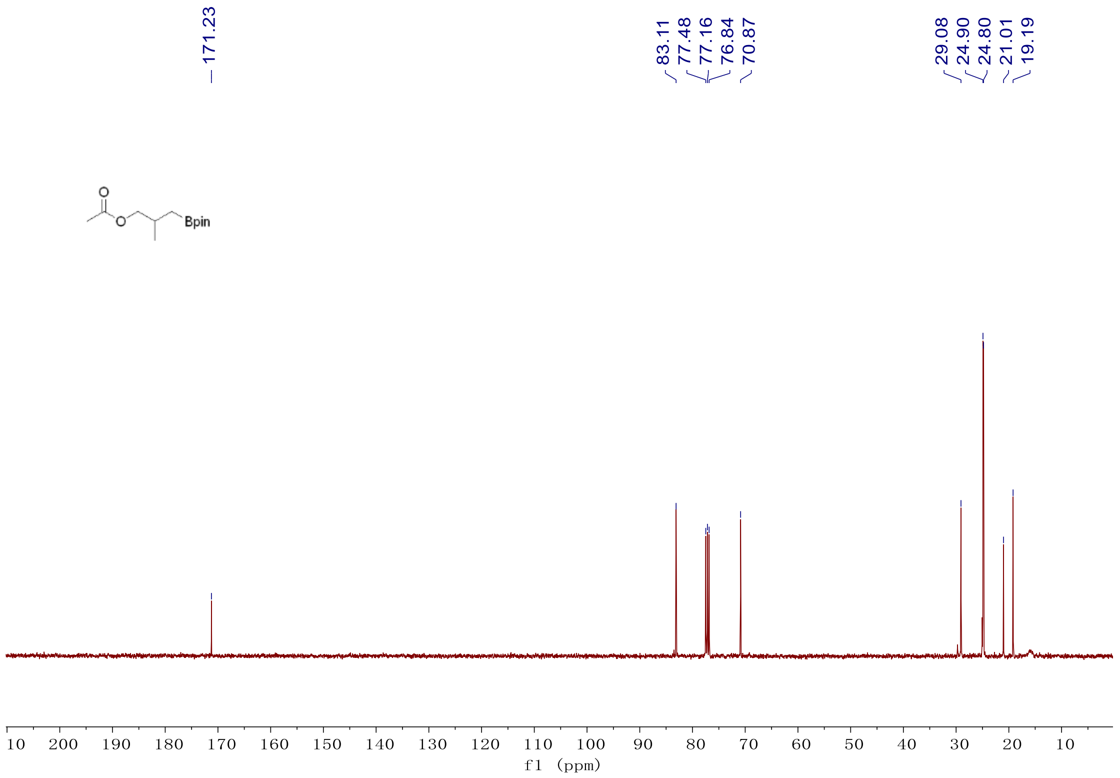

^{1}H NMR (400 MHz, $CDCl_3$) of **51** (*see procedure*):

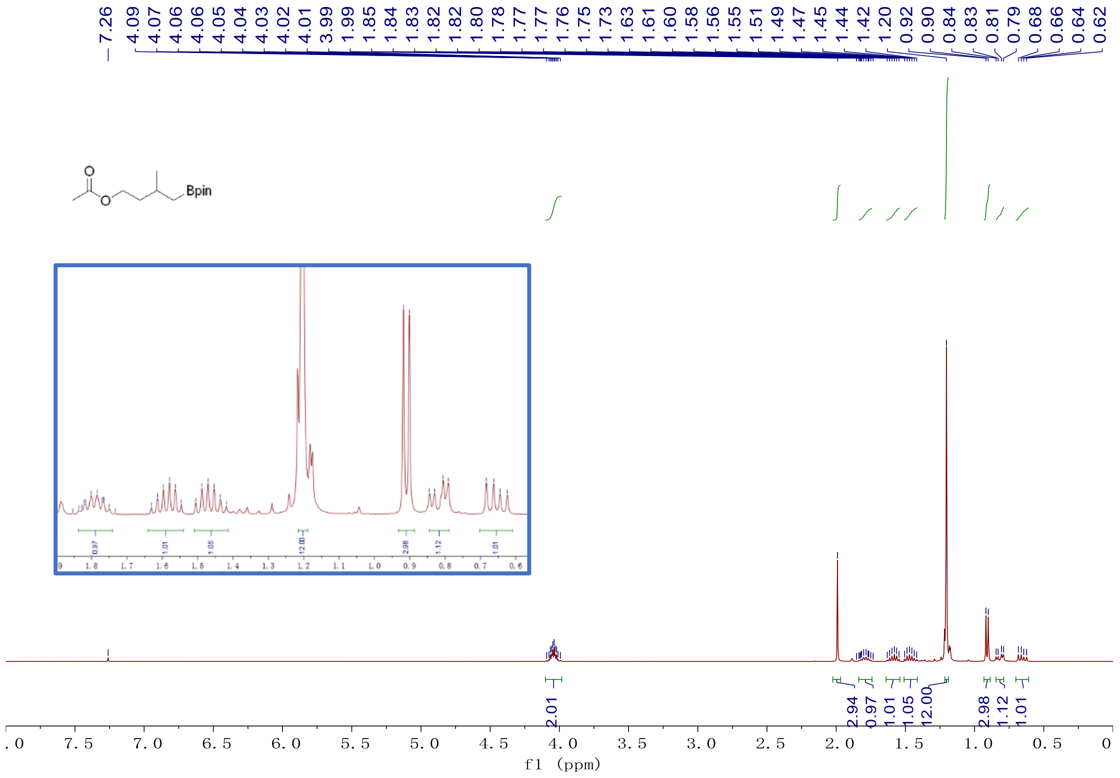

^{13}C NMR (101 MHz, $CDCl_3$) of **51** (*see procedure*):

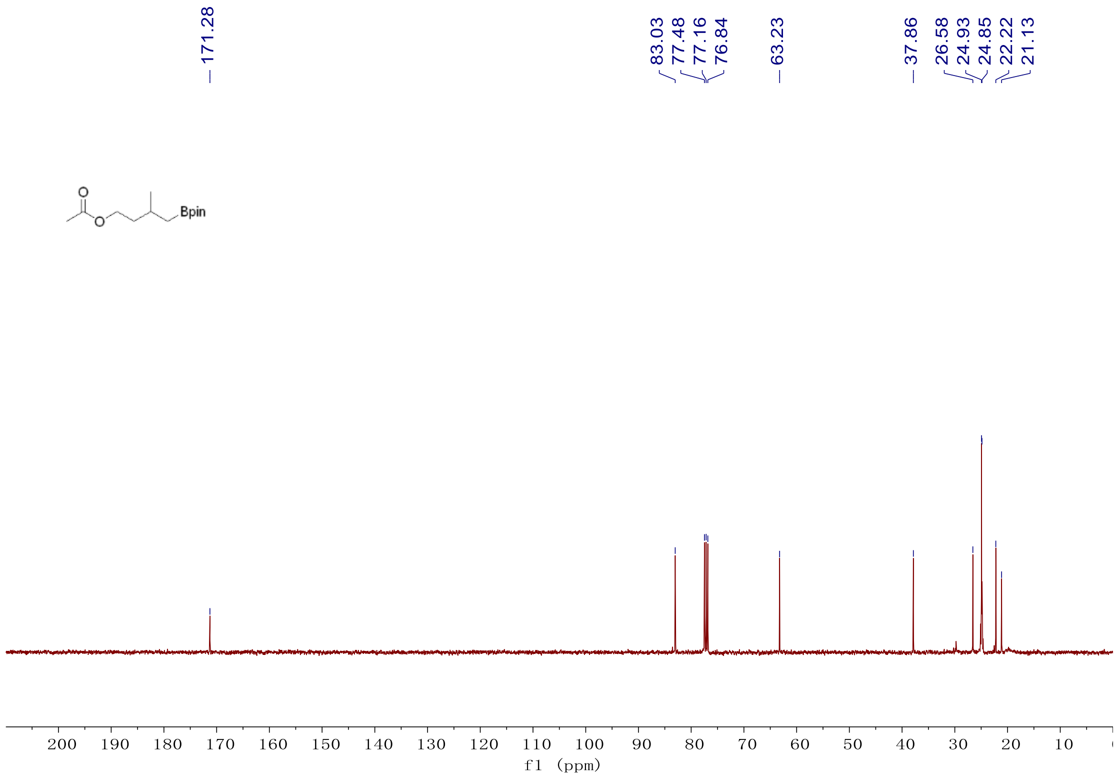

^{1}H NMR (400 MHz, $CDCl_3$) of **52** (*see procedure*):

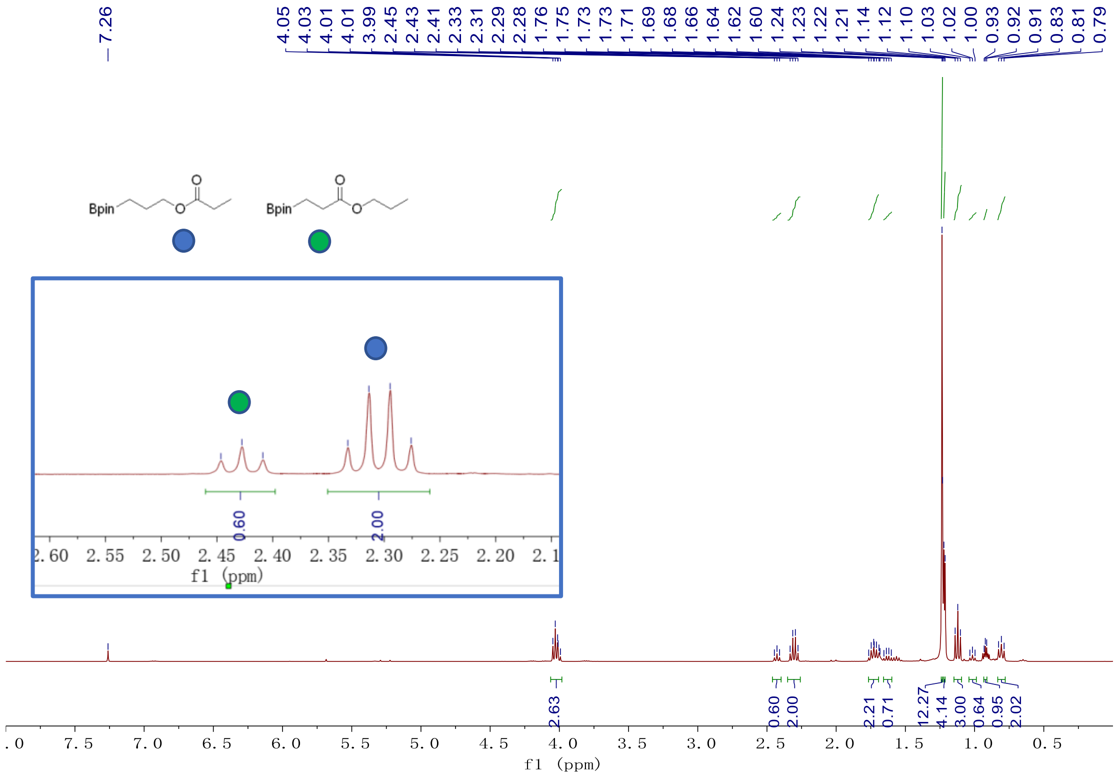

^{13}C NMR (101 MHz, $CDCl_3$) of **52** (*see procedure*):

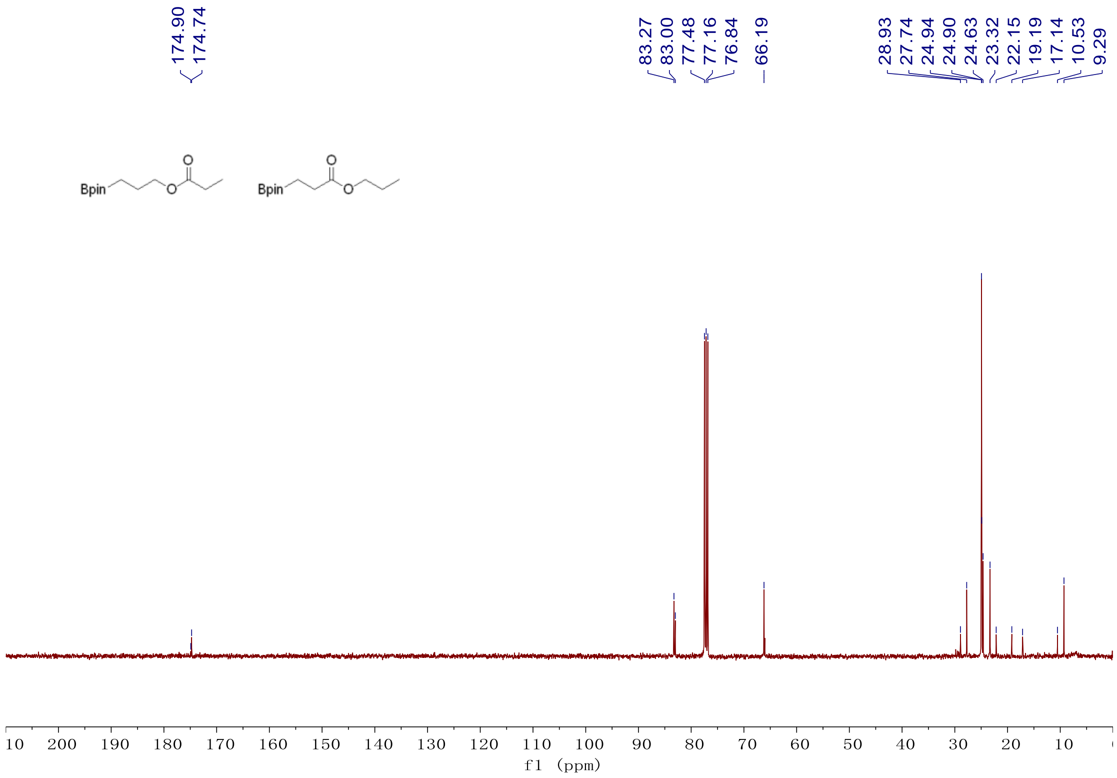

^{1}H NMR (400 MHz, $CDCl_3$) of **53** (see procedure):

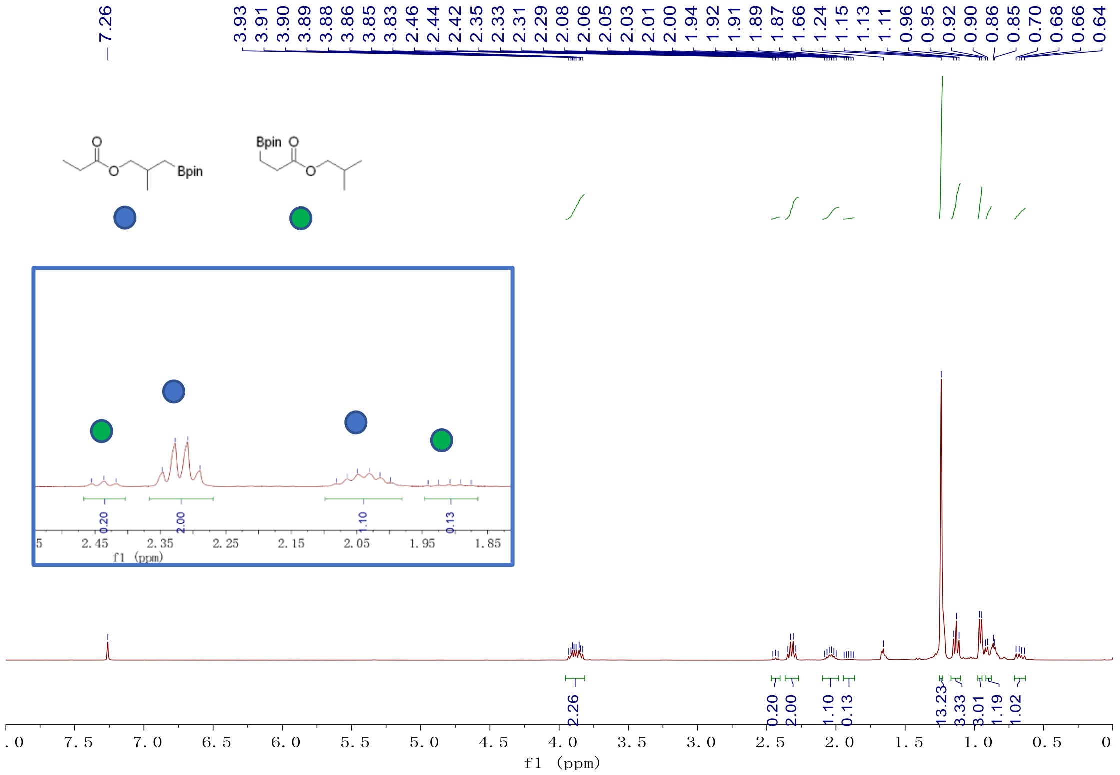

^{13}C NMR (101 MHz, $CDCl_3$) of **53** (see procedure):

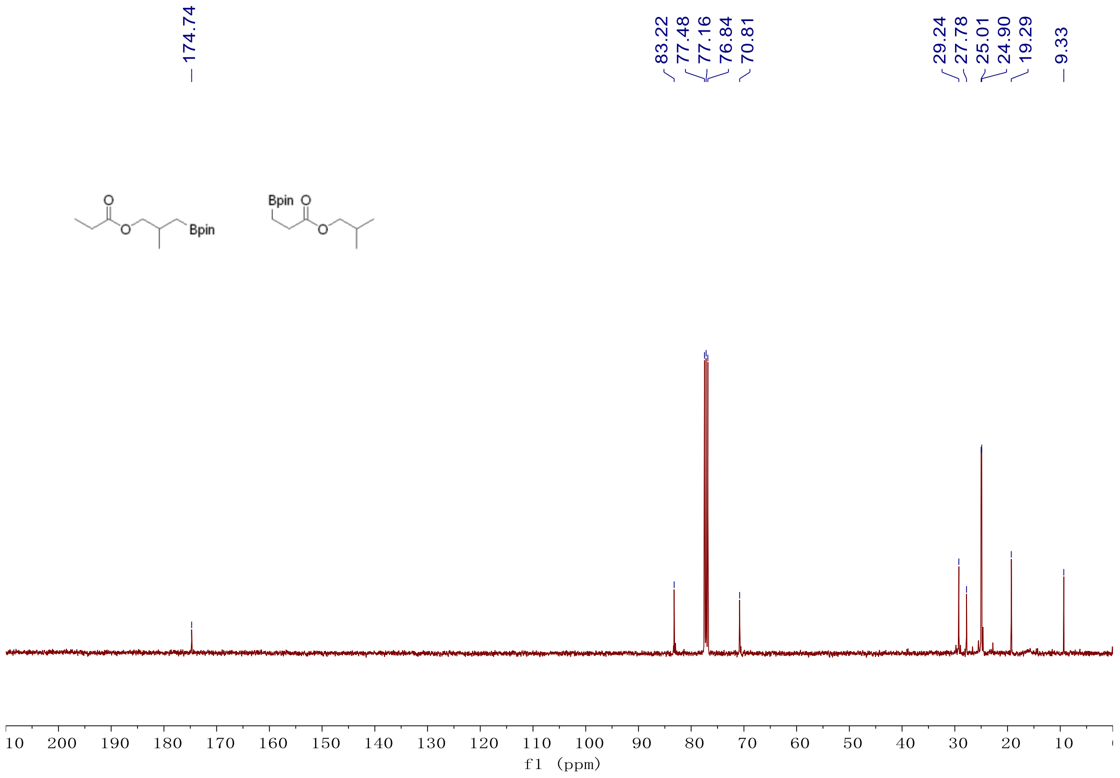

^{1}H NMR (400 MHz, $CDCl_3$) of **54** (<u>*see procedure*</u>):

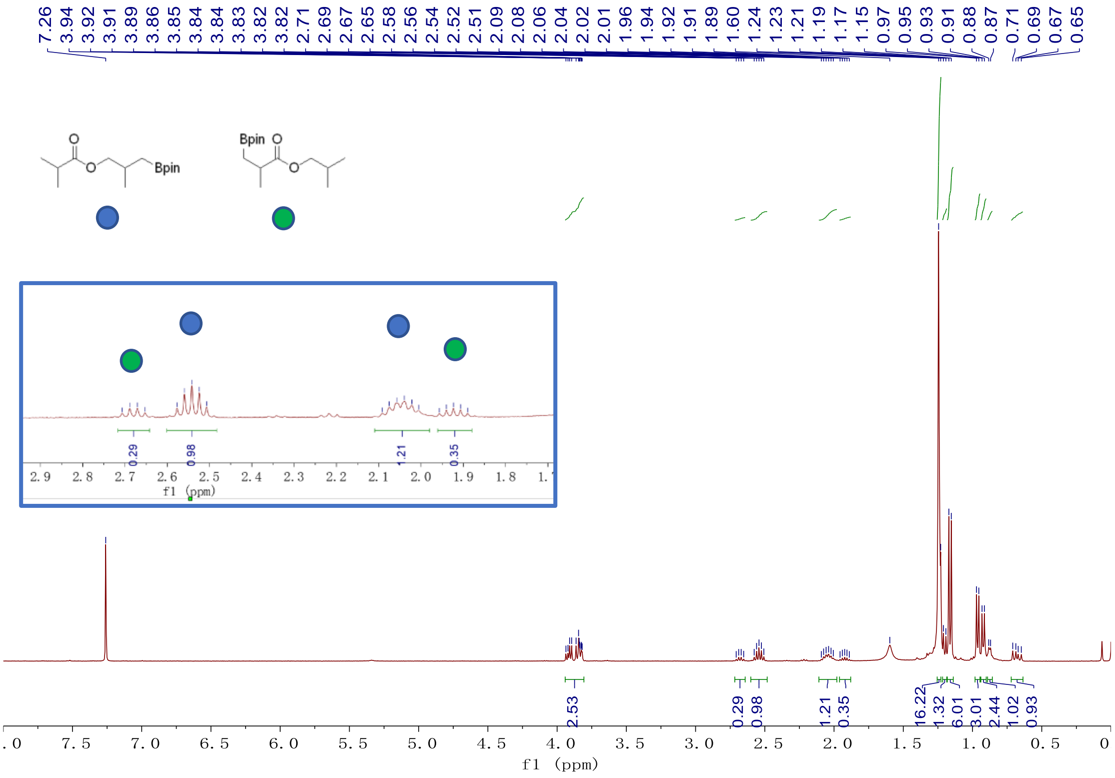

^{13}C NMR (101 MHz, $CDCl_3$) of **54** (<u>*see procedure*</u>):

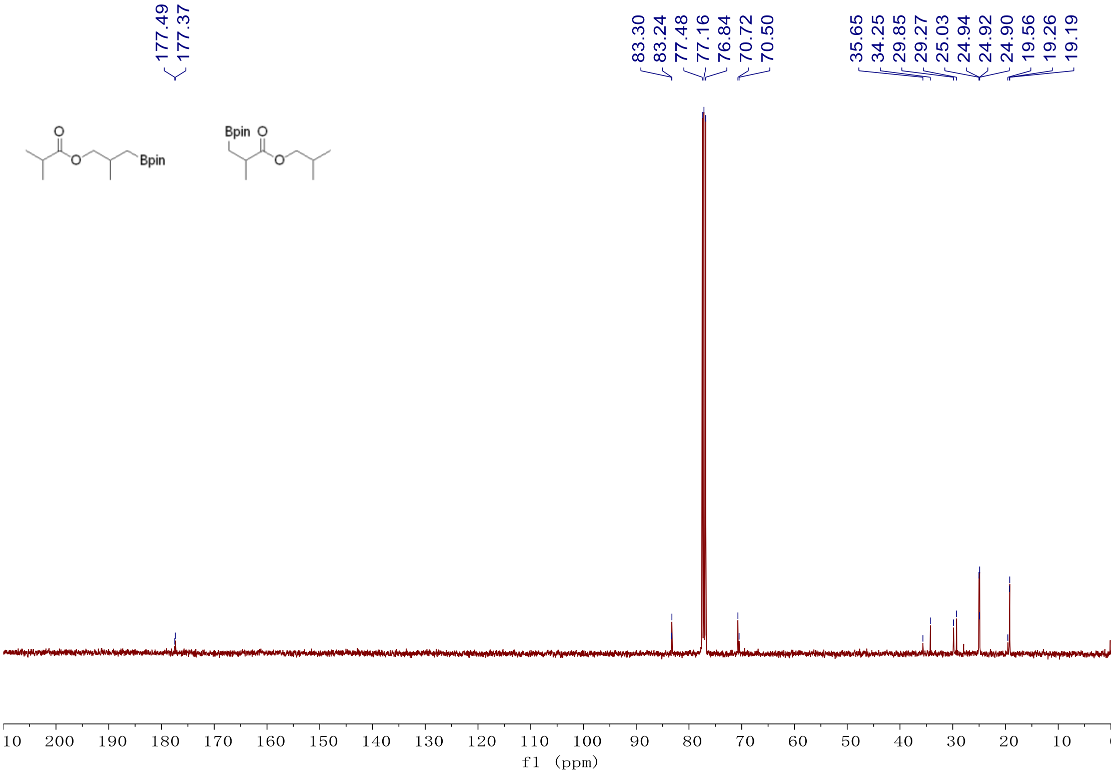

^{1}H NMR (400 MHz, $CDCl_3$) of **55** (*see procedure*):

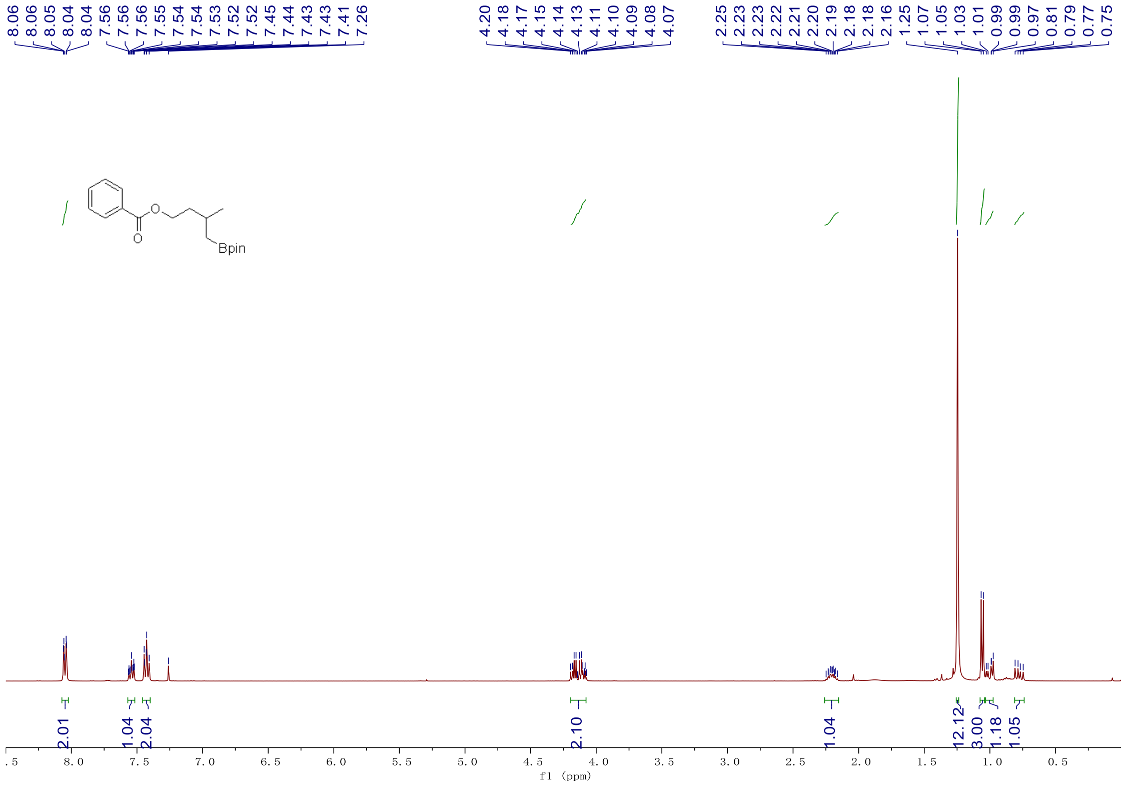

^{13}C NMR (101 MHz, $CDCl_3$) of **55** (*see procedure*):

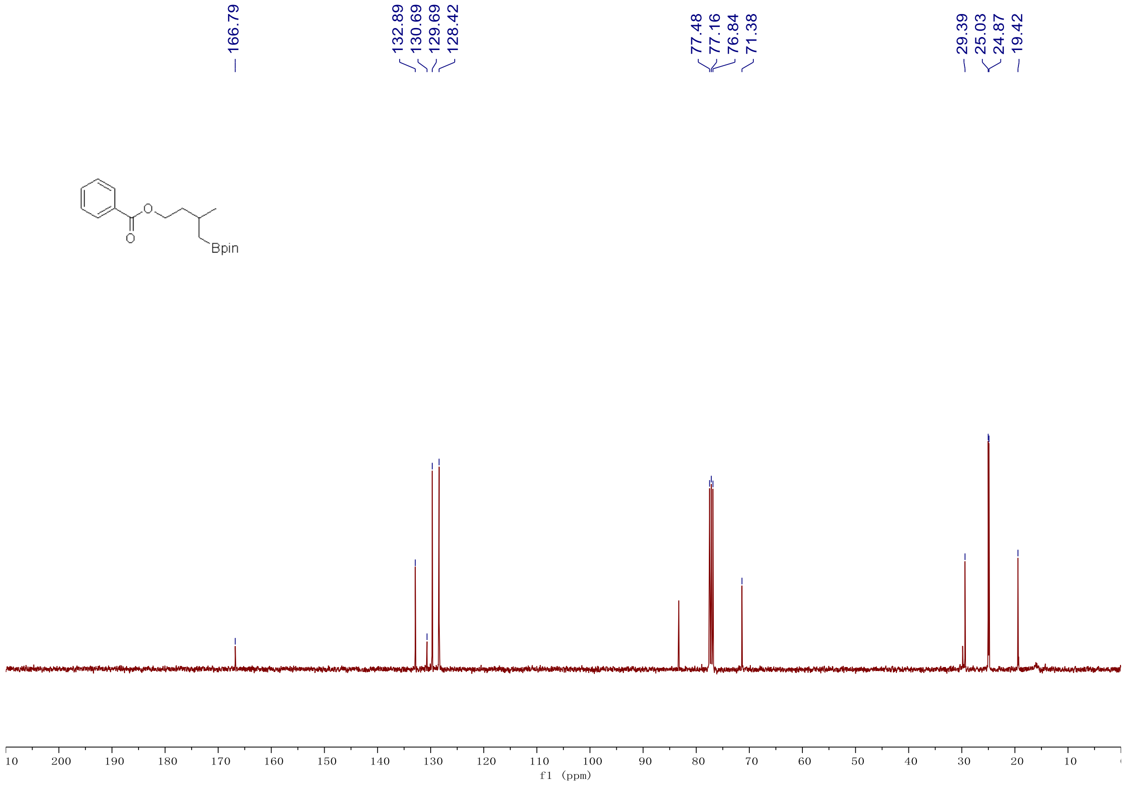

^{1}H NMR (400 MHz, $CDCl_3$) of **56** (*see procedure*):

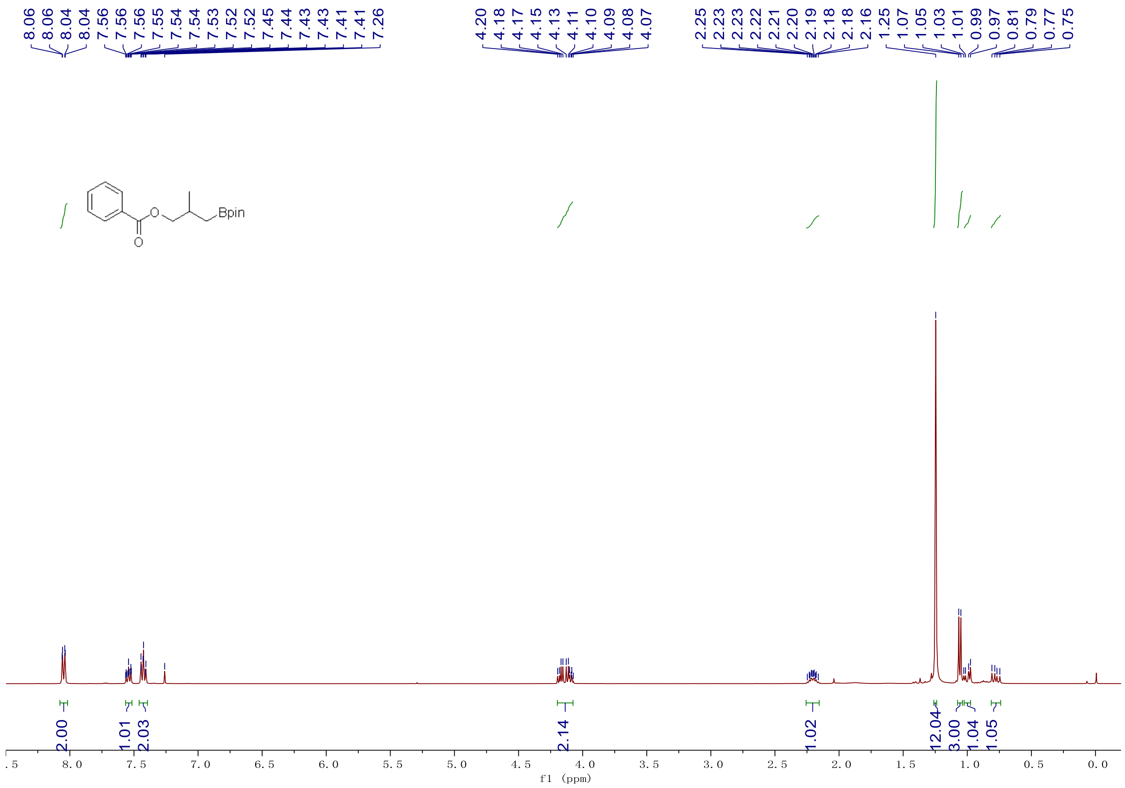

^{13}C NMR (101 MHz, $CDCl_3$) of **56** (*see procedure*):

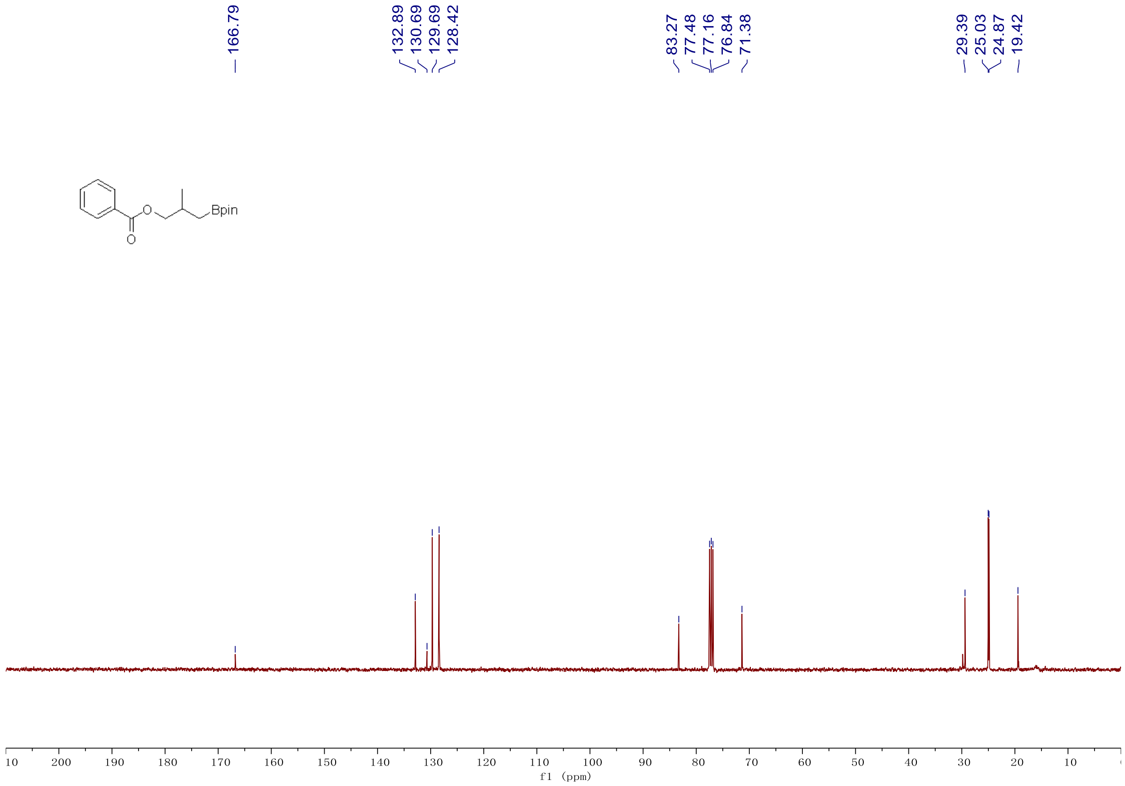

^{1}H NMR (400 MHz, $CDCl_3$) of **57** (*see procedure*):

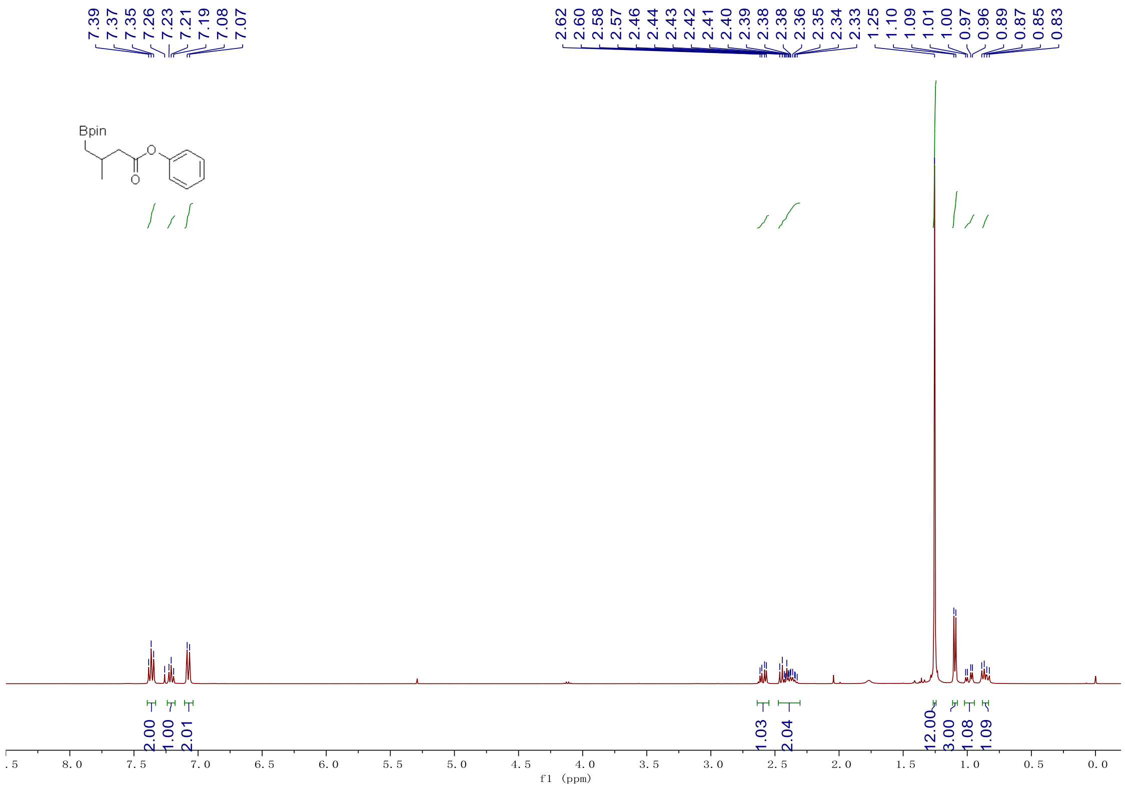

^{13}C NMR (101 MHz, $CDCl_3$) of **57** (see procedure):

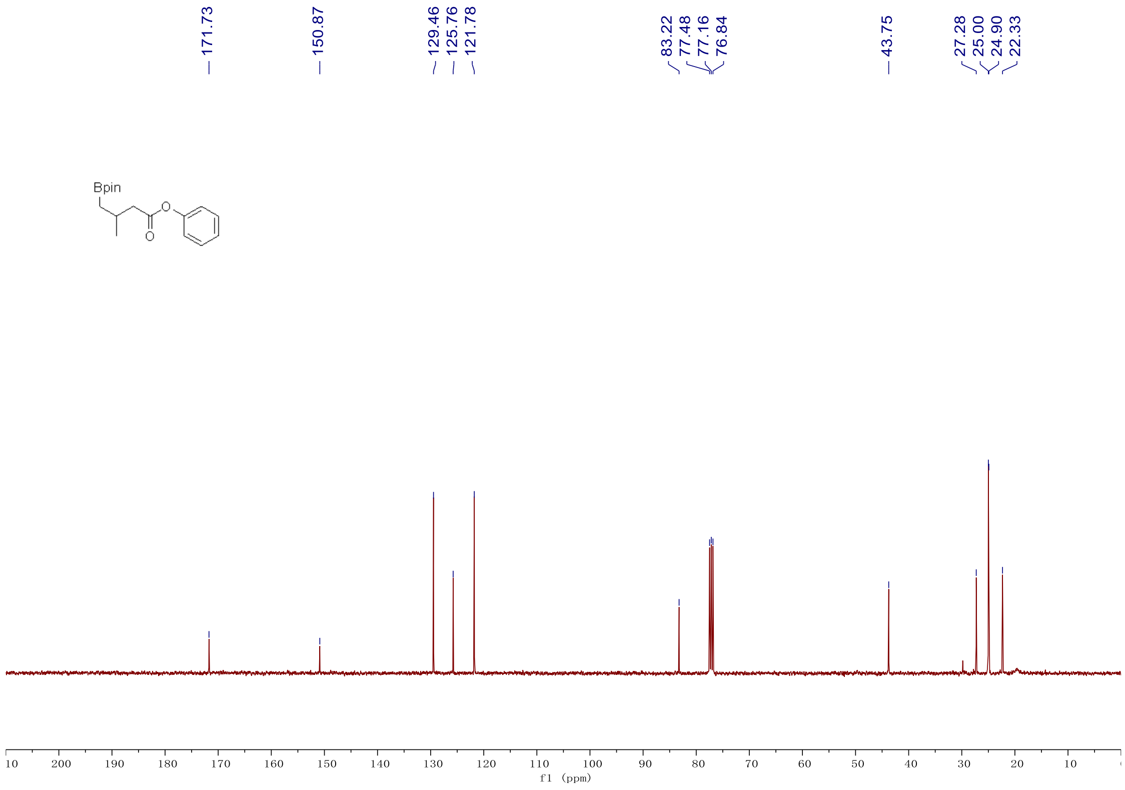

^{1}H NMR (400 MHz, $CDCl_3$) of **58-δ** (see procedure):

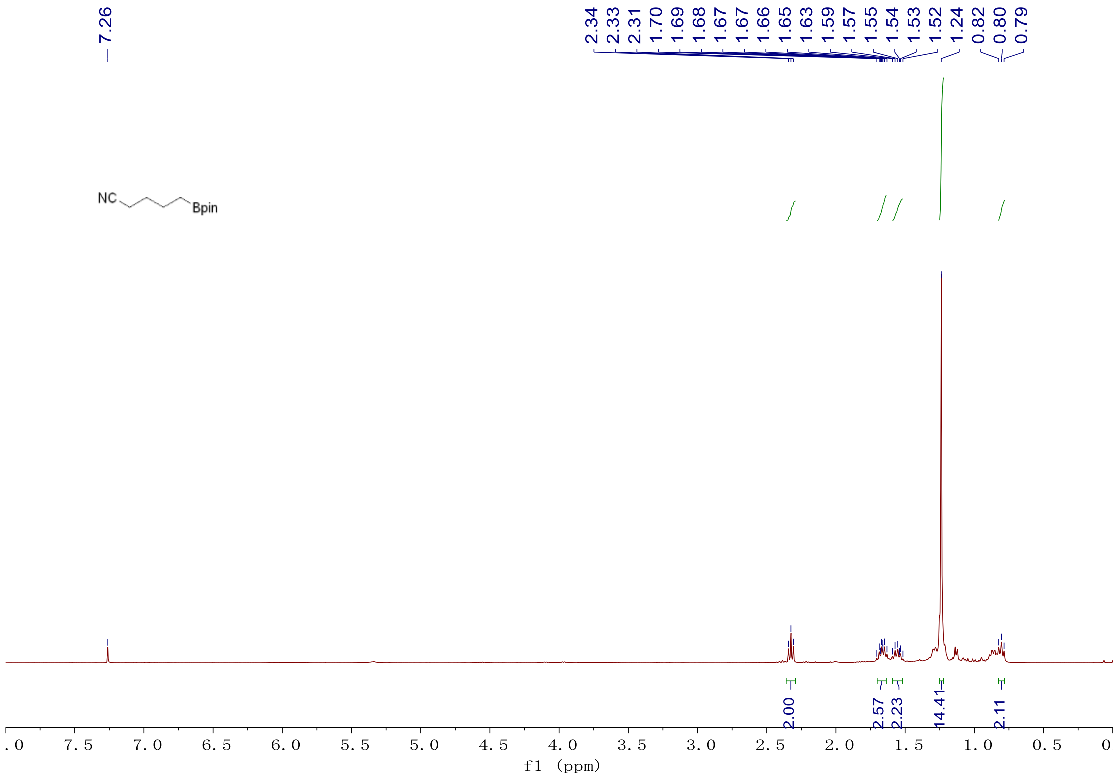

^{13}C NMR (101 MHz, $CDCl_3$) of **58-δ** (see procedure):

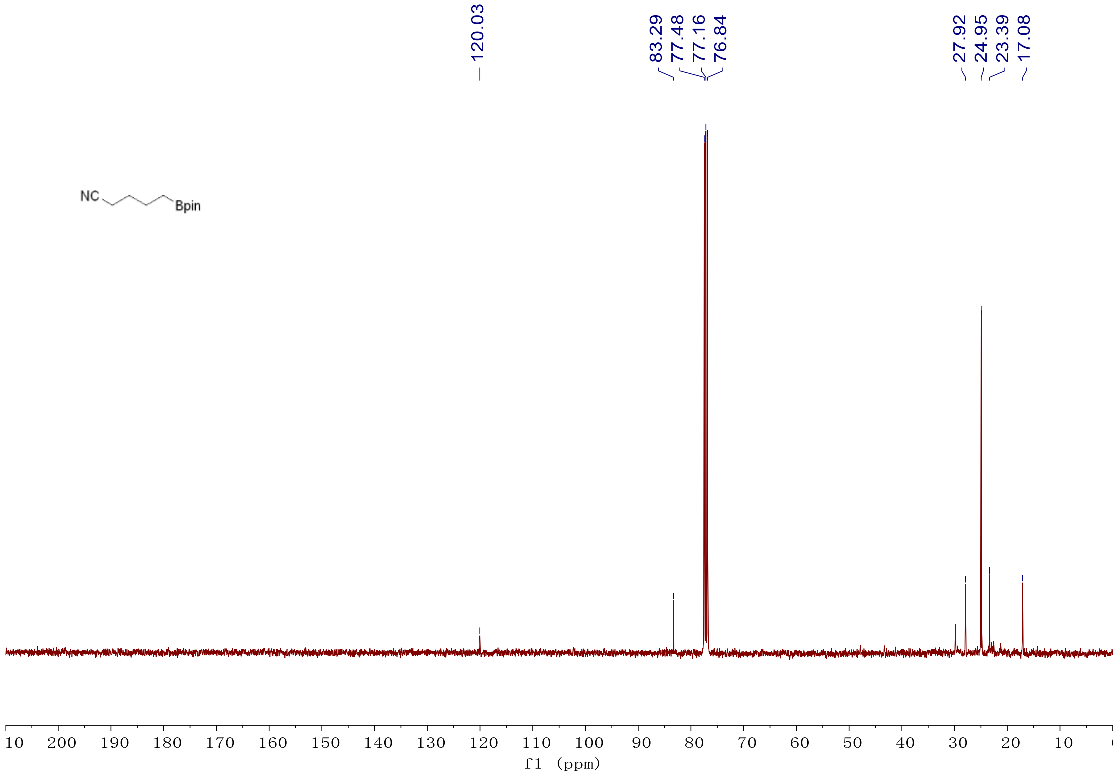

^{1}H NMR (400 MHz, $CDCl_3$) of **59** (see procedure):

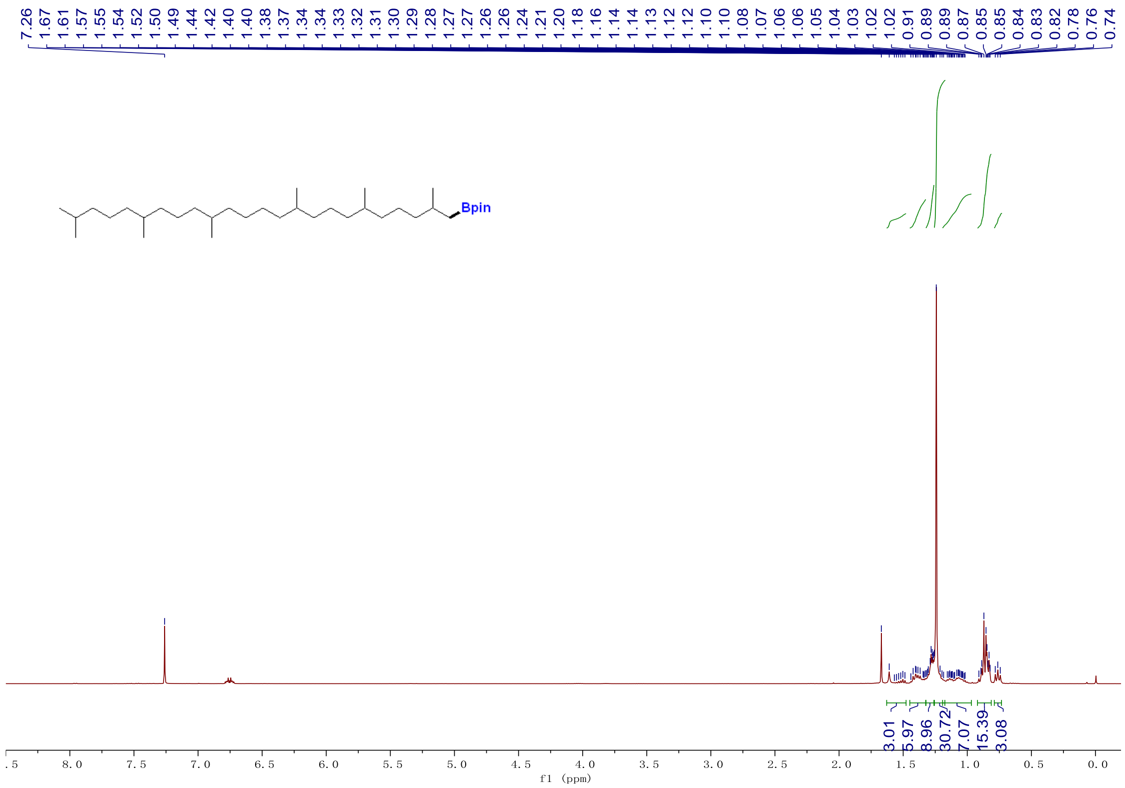

^{13}C NMR (101 MHz, $CDCl_3$) of **59** (*see procedure*):

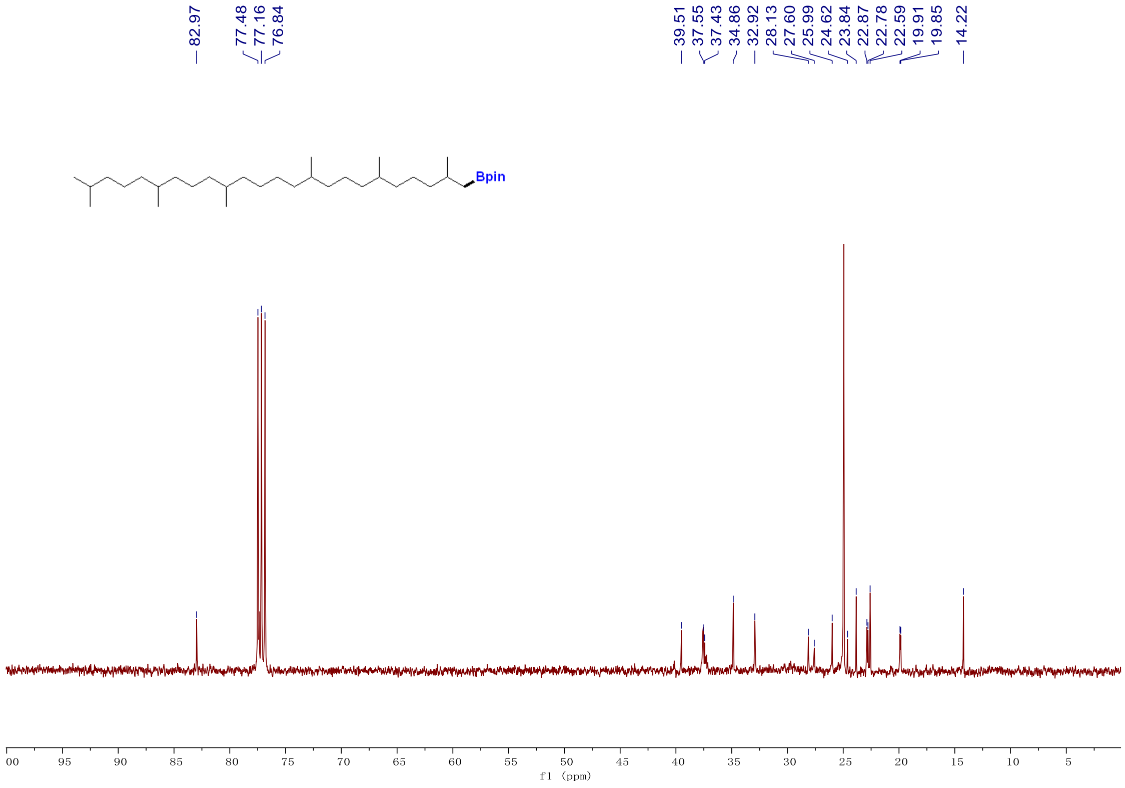

^{1}H NMR (400 MHz, $CDCl_3$) of **61**

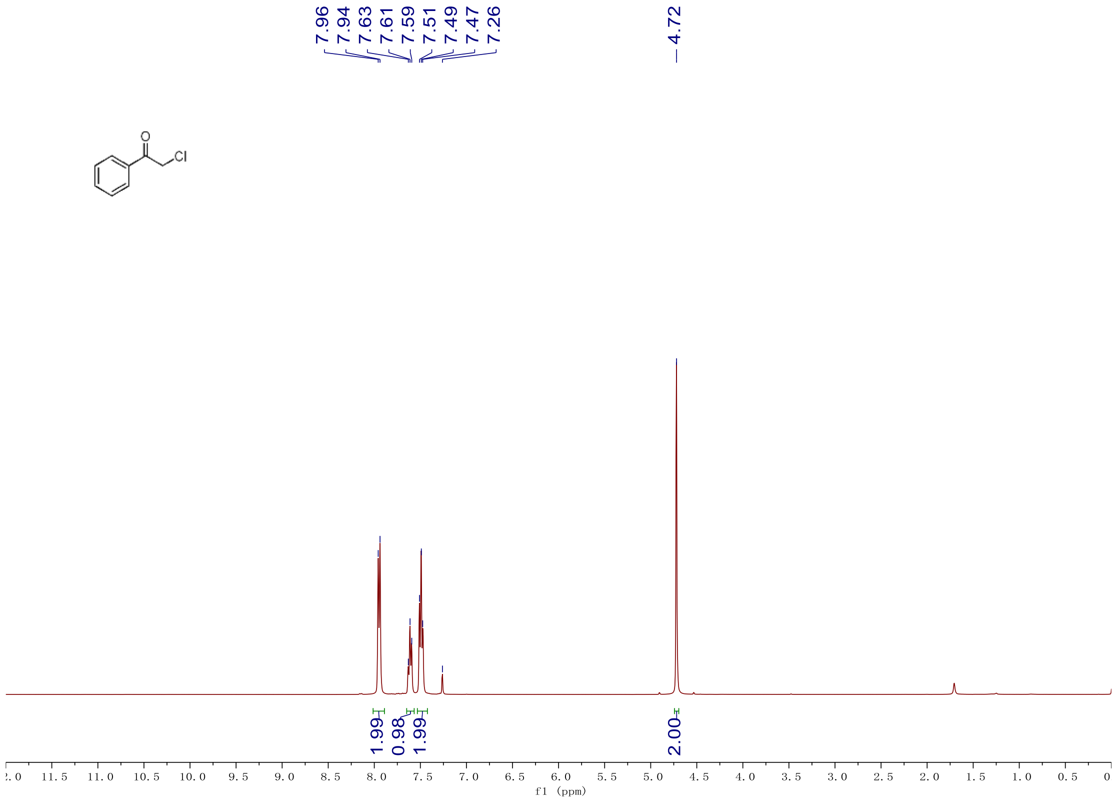

^{13}C NMR (101 MHz, $CDCl_3$) of **61**

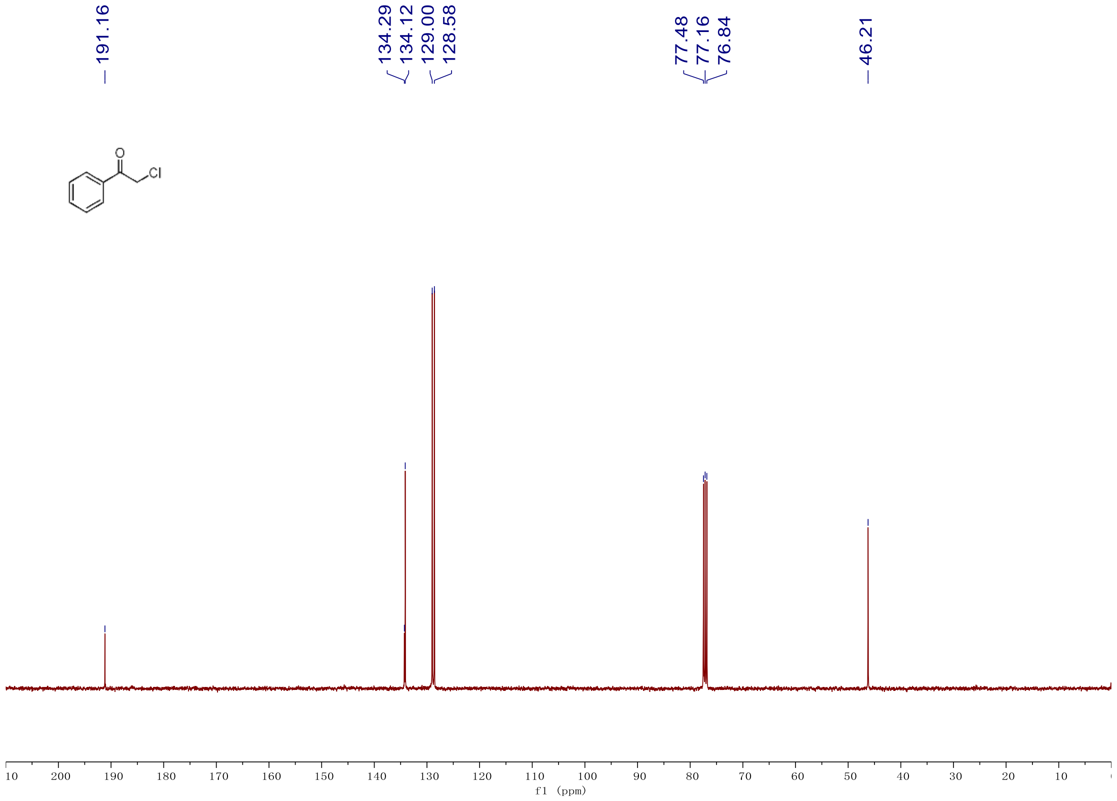

^{1}H NMR (400 MHz, $CDCl_3$) of **63**

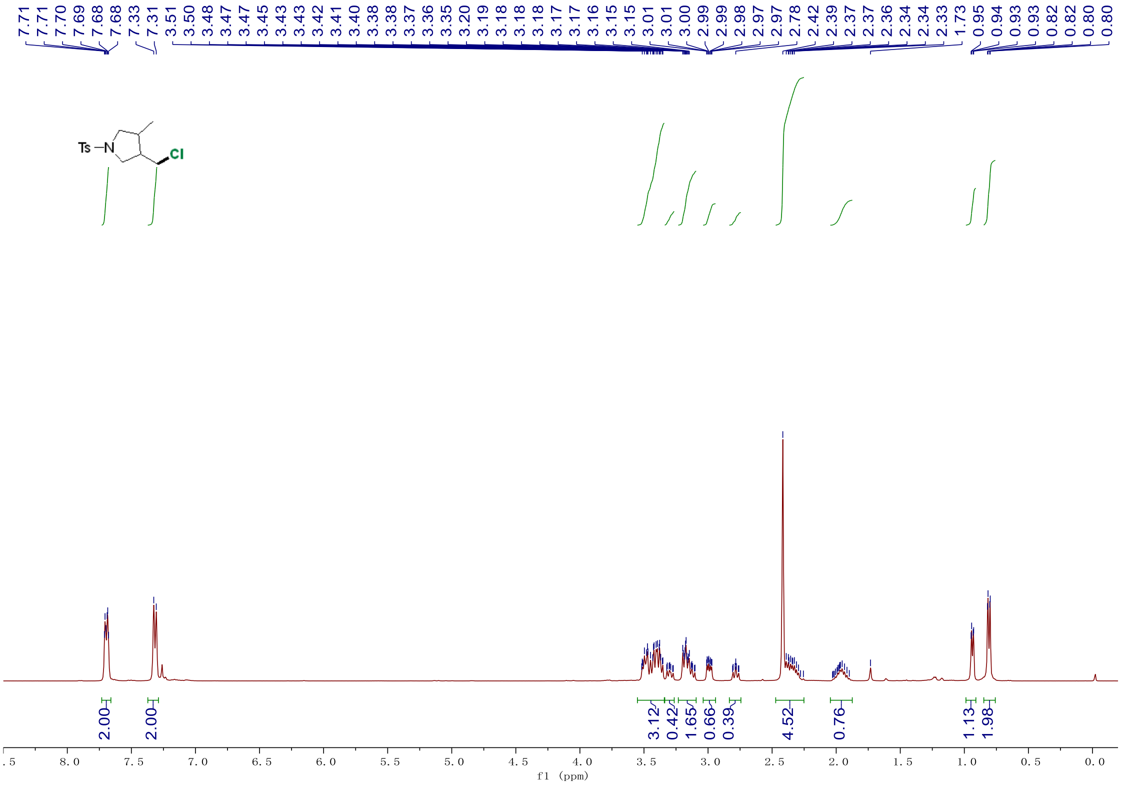

^{13}C NMR (101 MHz, $CDCl_3$) of **63**

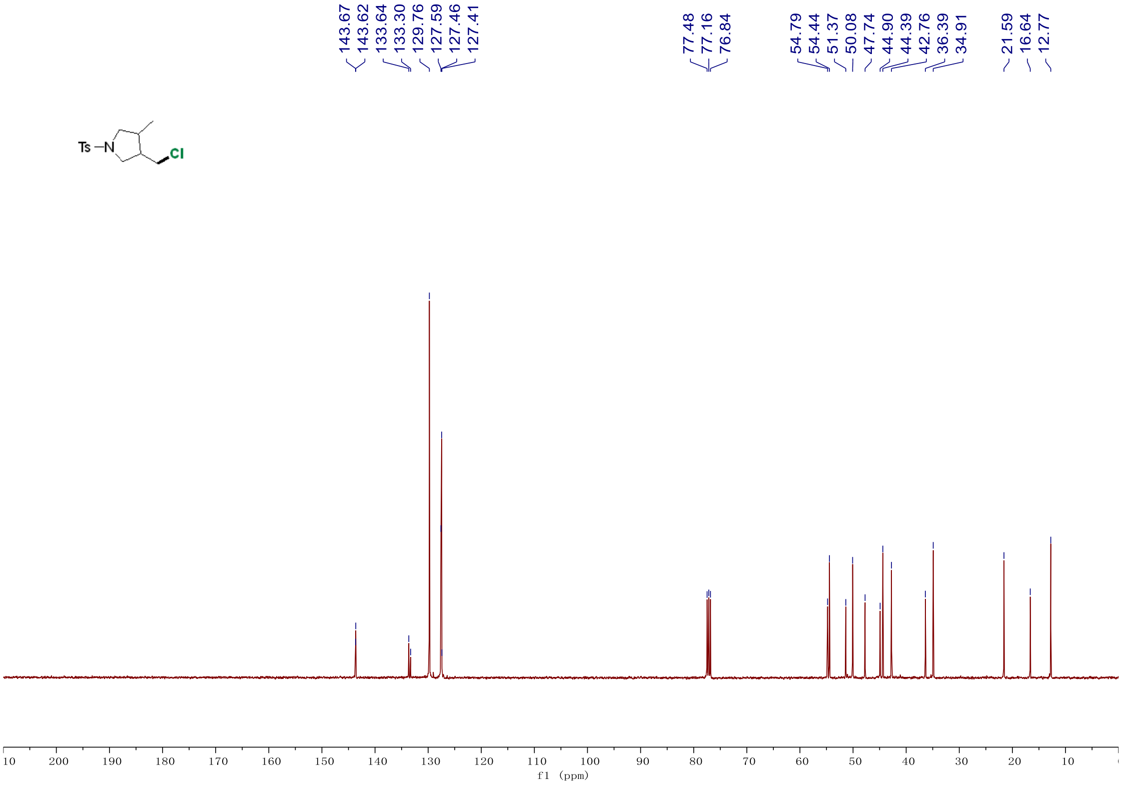

^{1}H NMR (400 MHz, $CDCl_3$) of **65**

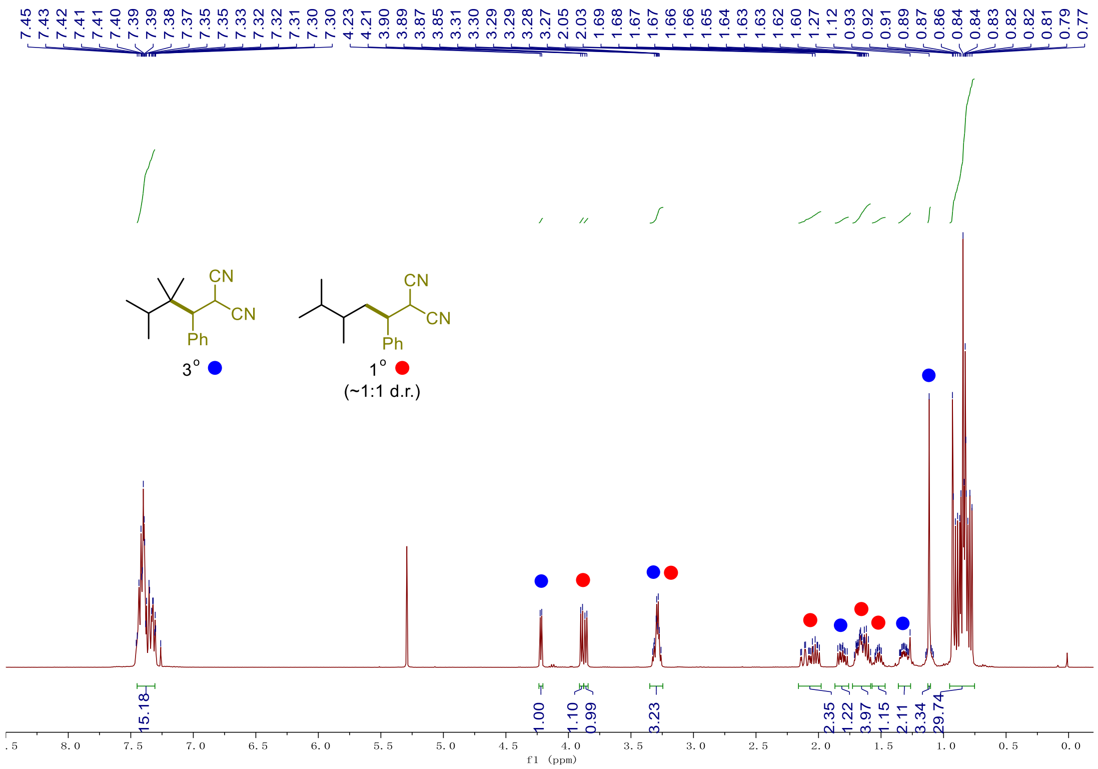

^{13}C NMR (101 MHz, CDCl$_3$) of **65**

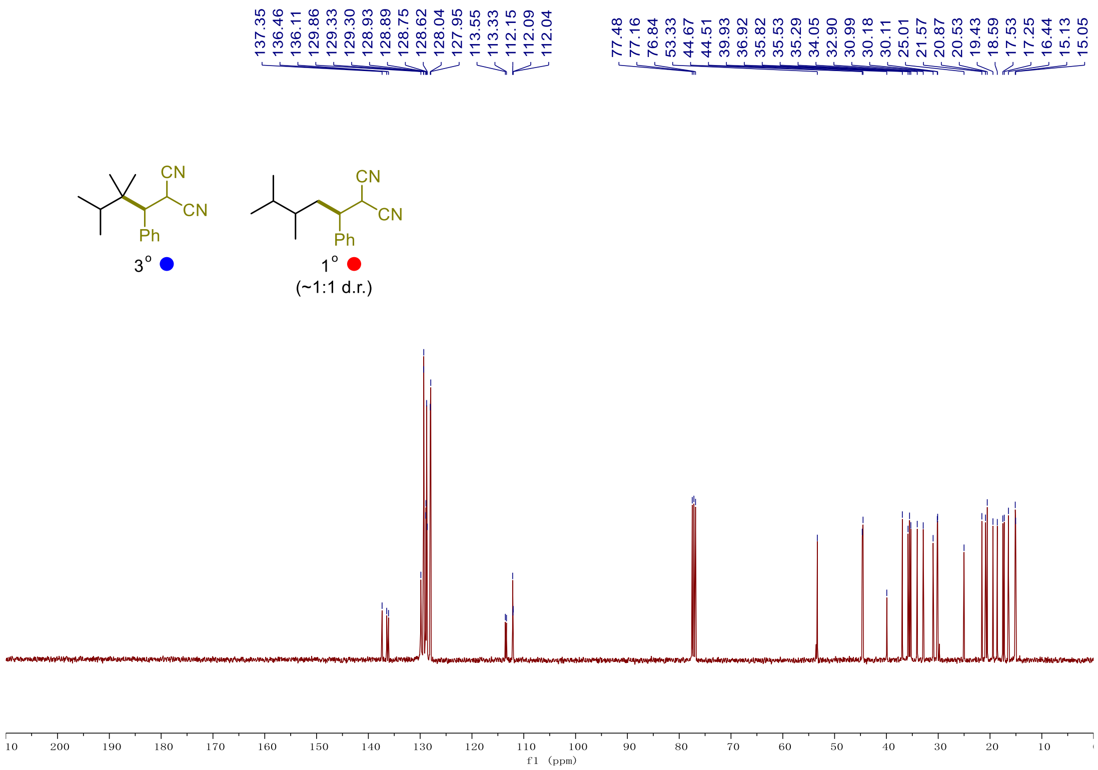

^{1}H NMR (400 MHz, CDCl$_3$) of **67**

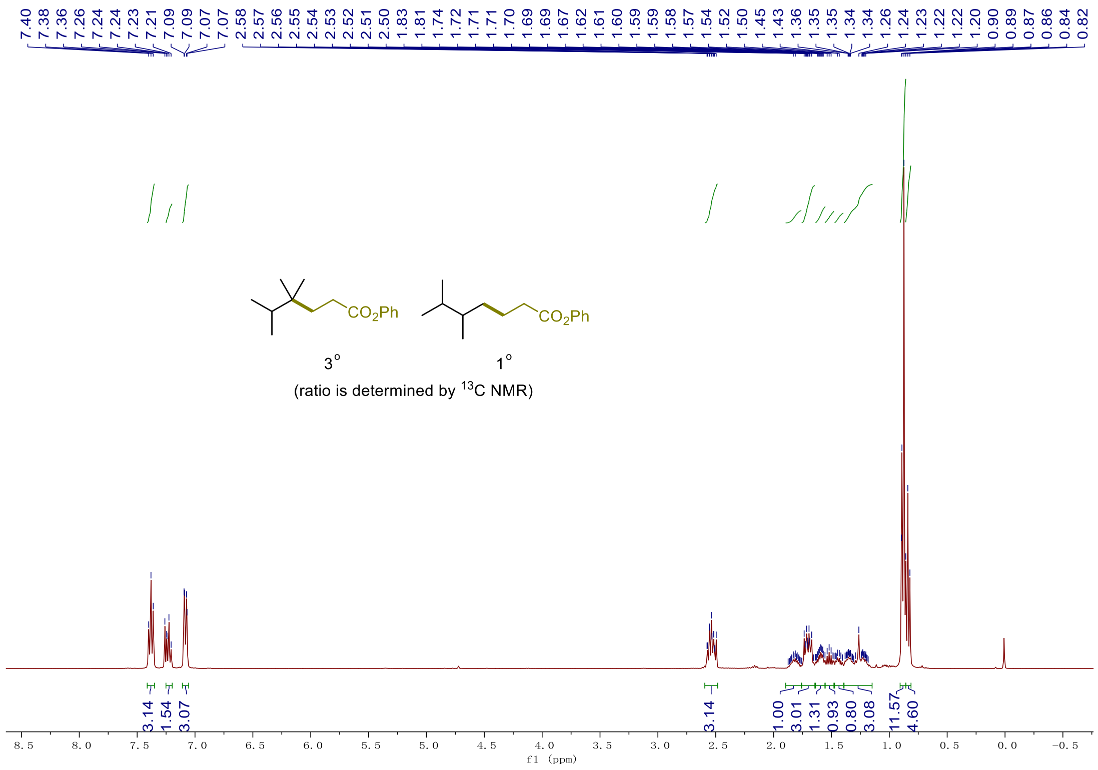

^{13}C NMR (101 MHz, $CDCl_3$) of **67**

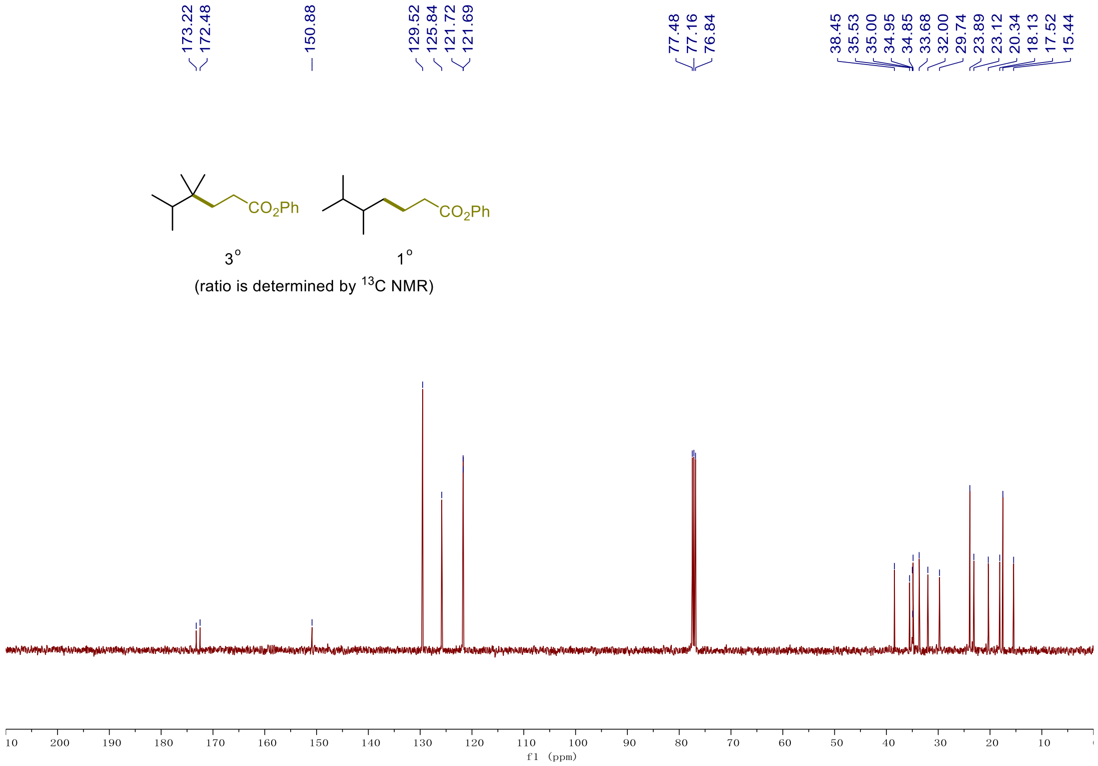

5. GC Spectra

All the GC data recorded by analysis of the crude reaction mixtures.

GC of **8** (see procedure):

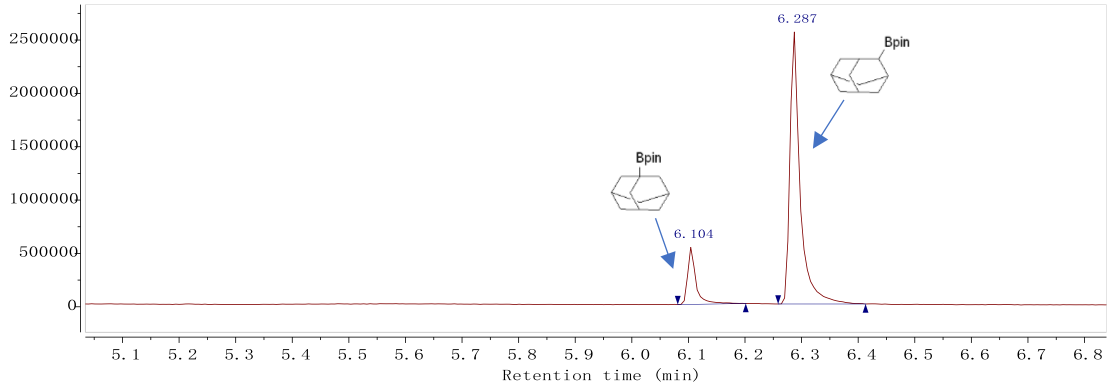

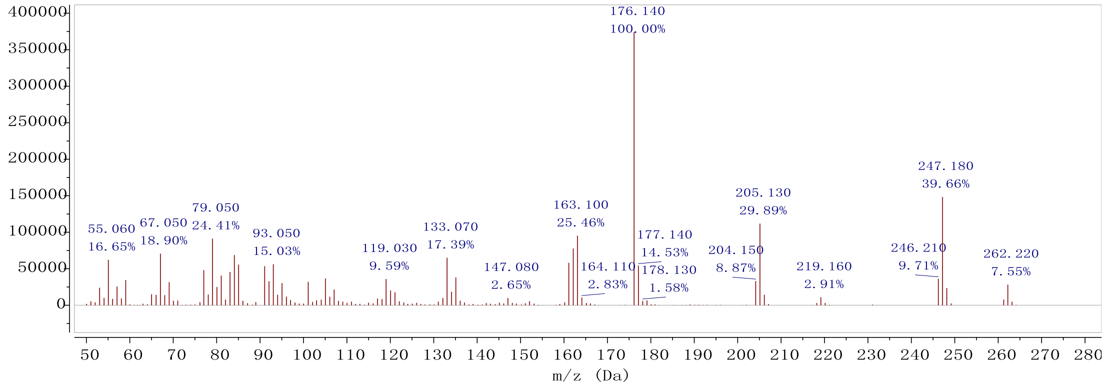

GC of **9** (see procedure):

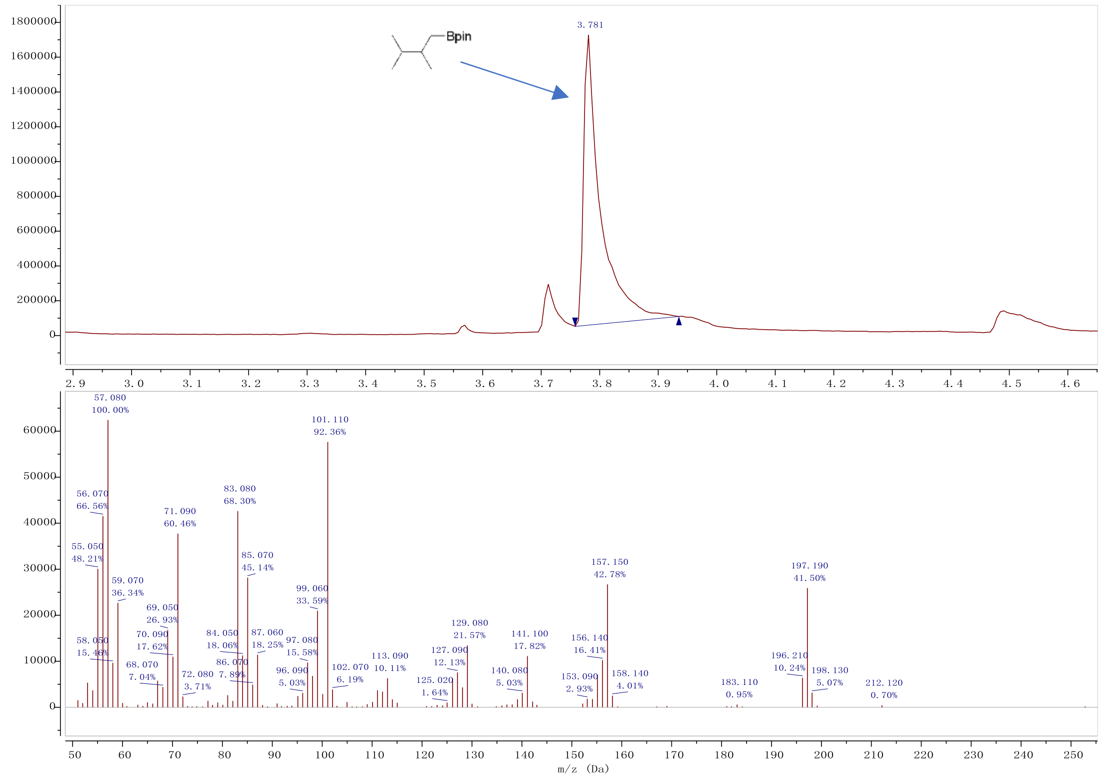

GC of **11** (see procedure):

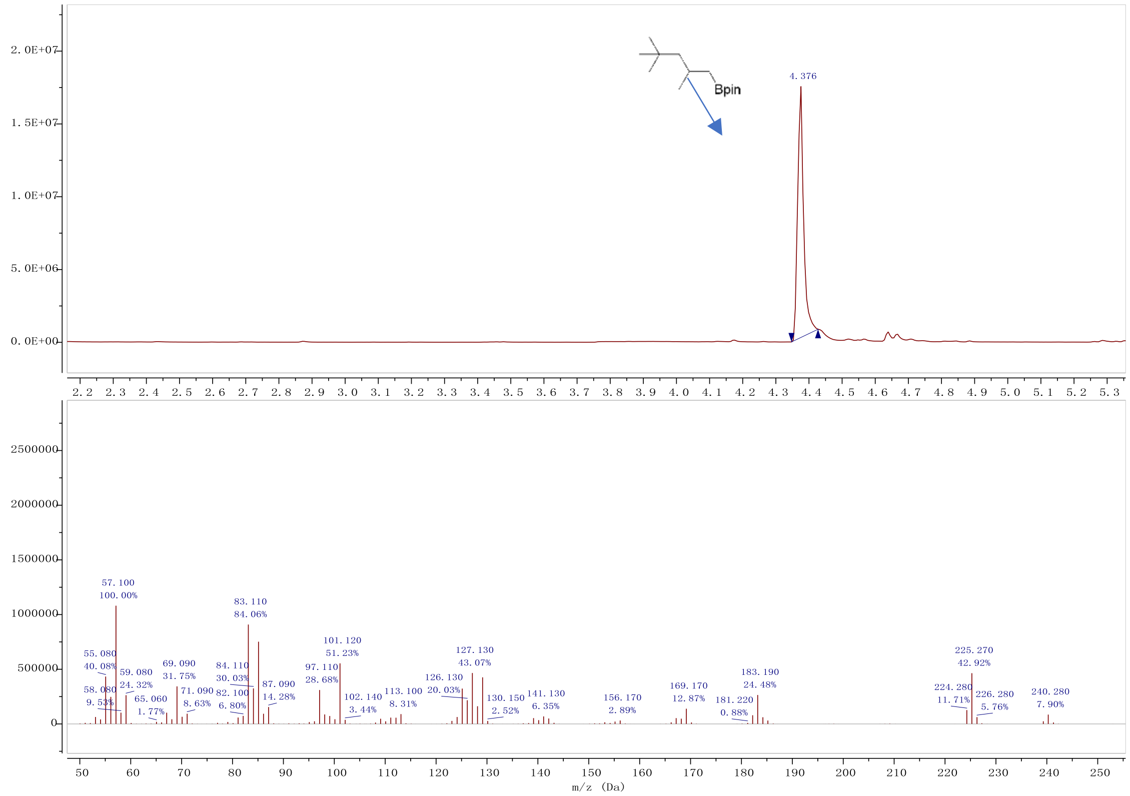

GC of **13** (see procedure):

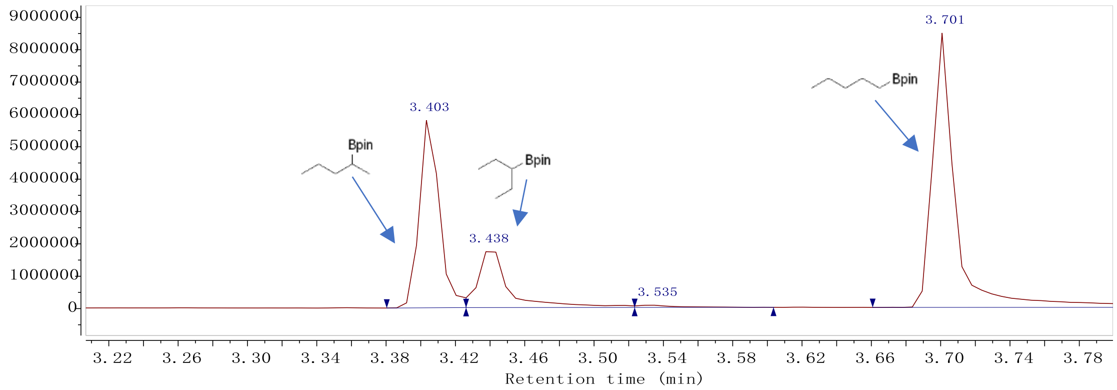

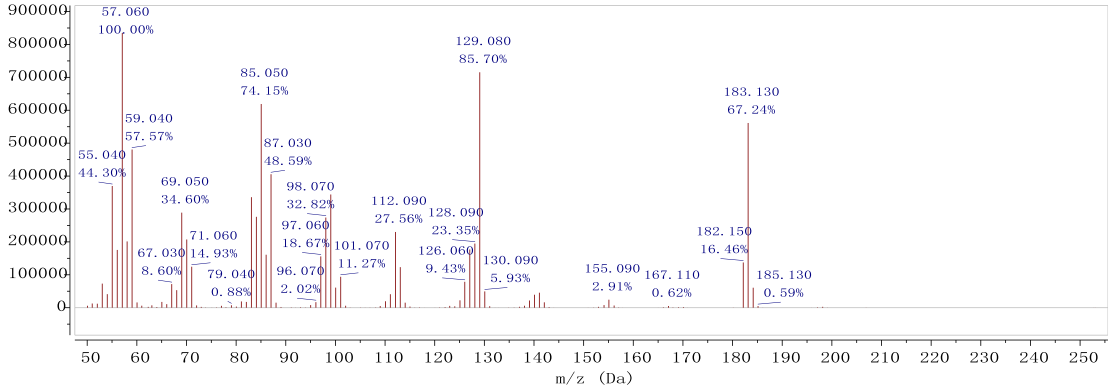

GC of **14** (see procedure):

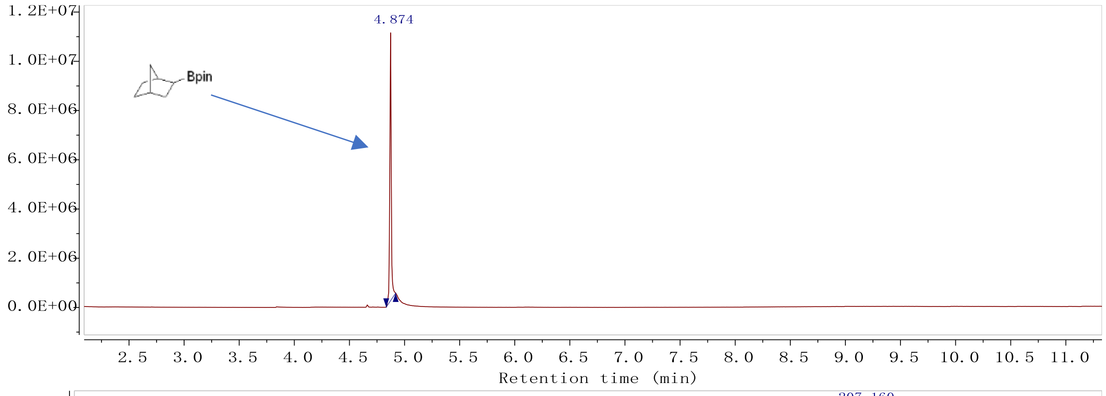

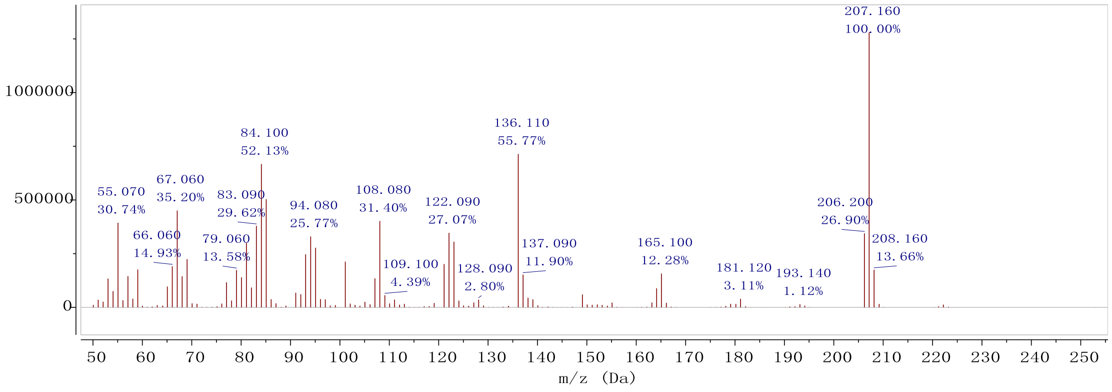

GC of **17** (see procedure):

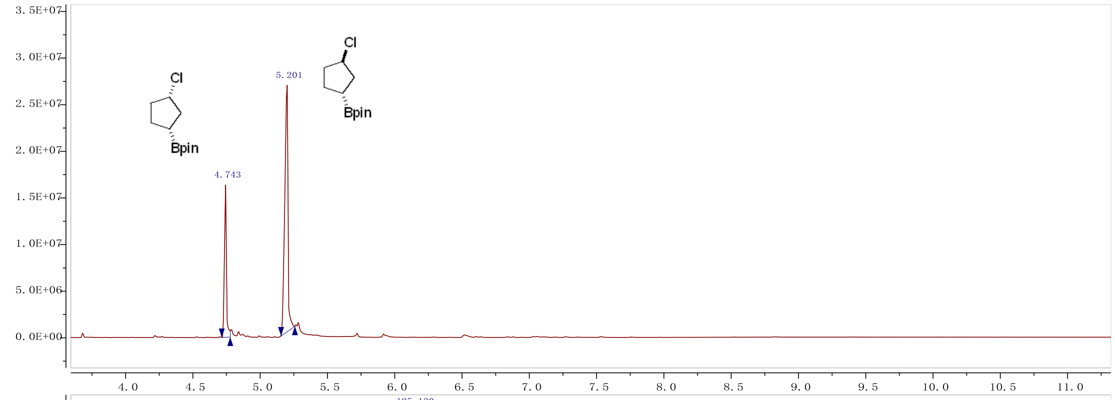

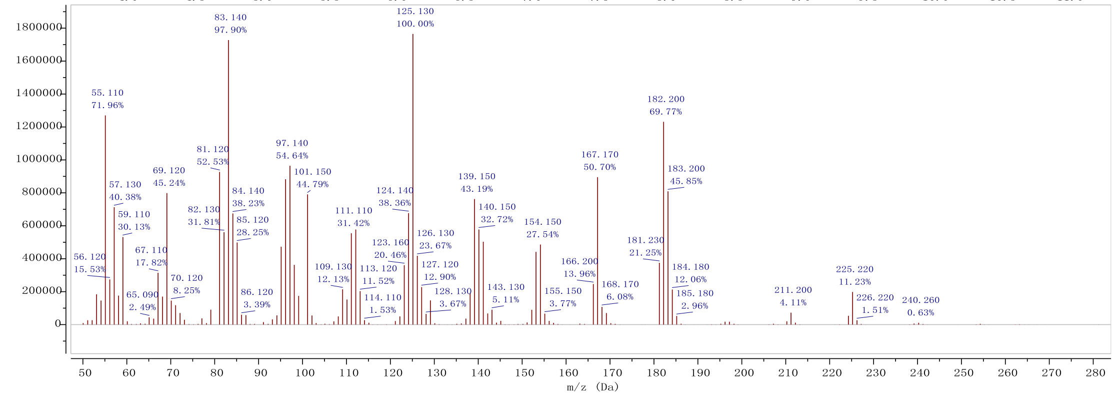

GC of **26** (see procedure):

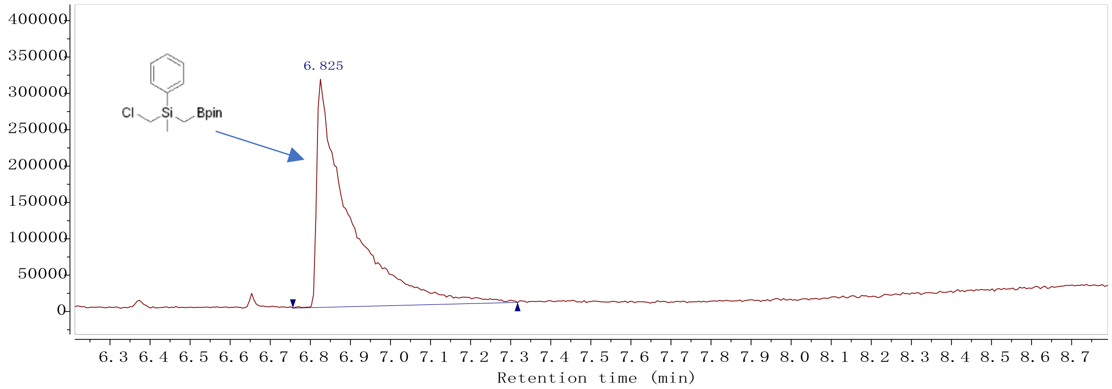

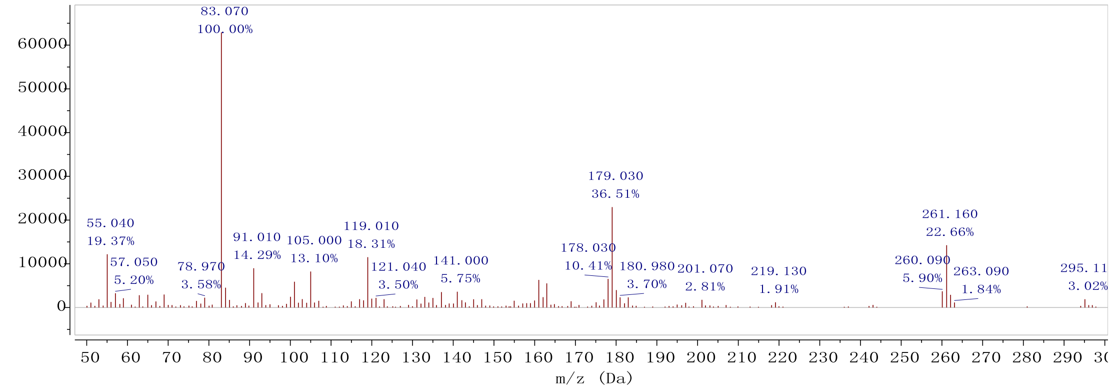

GC of **28** (see procedure):

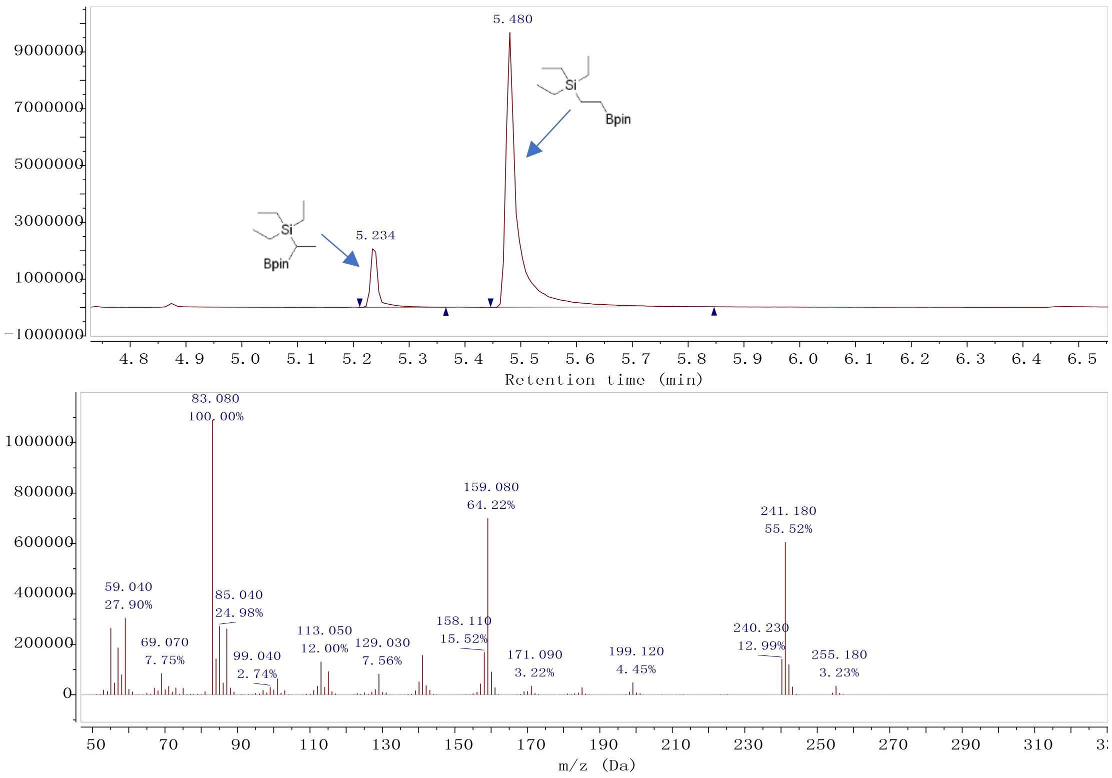

GC of **34** (see procedure):

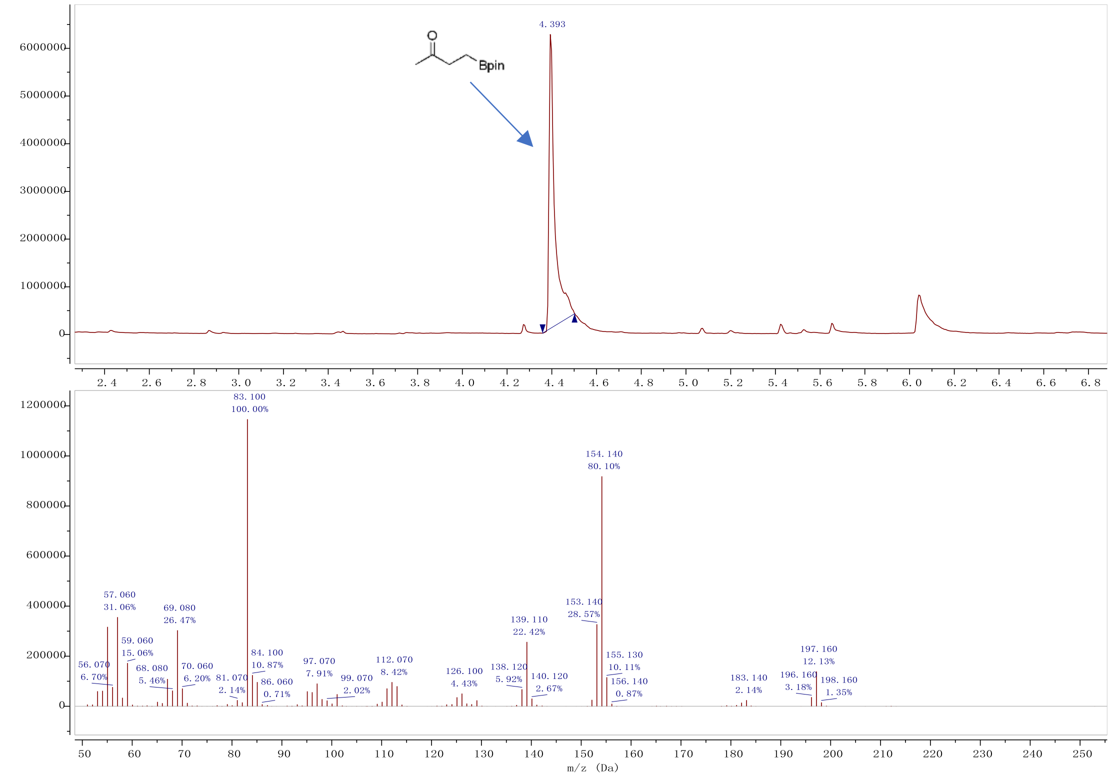

GC of **35** (see procedure):

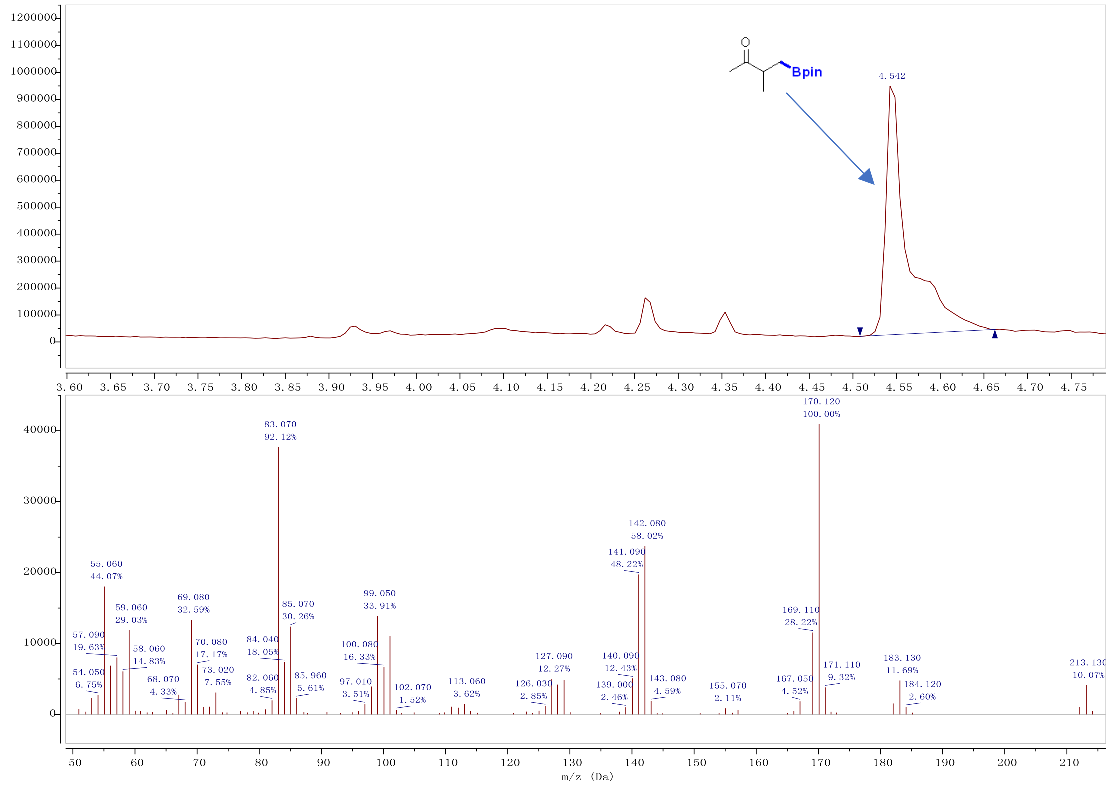

GC of **36** (see procedure):

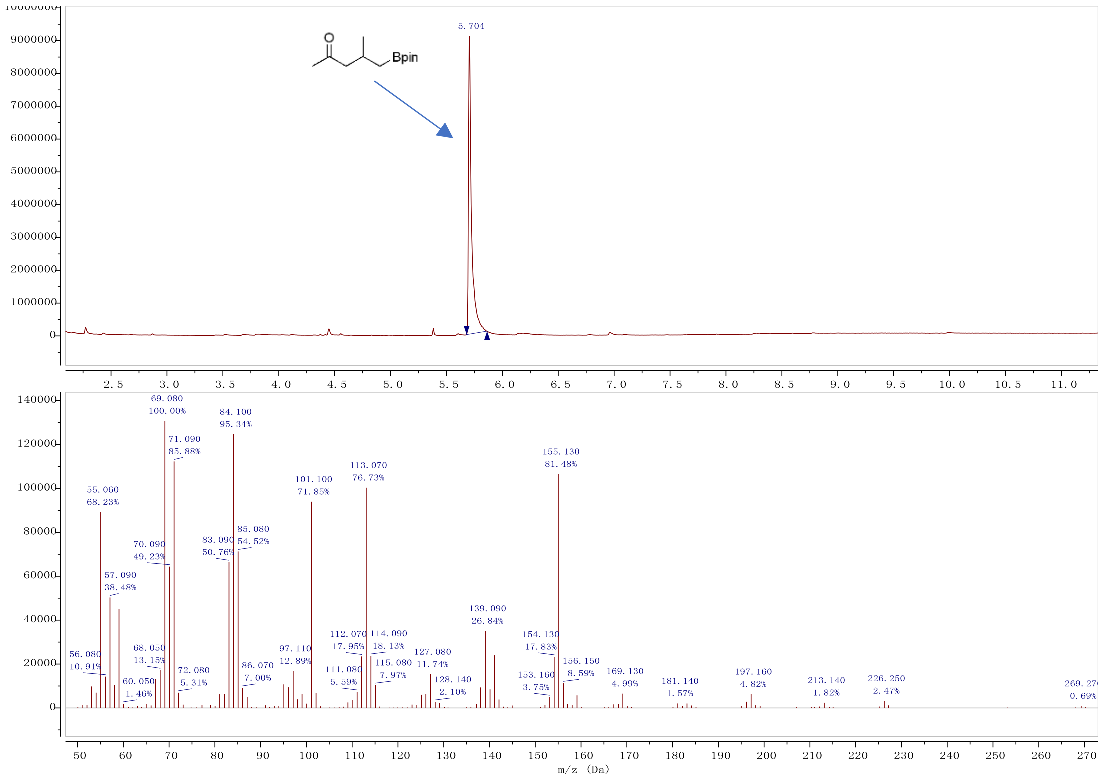

GC of **38** (see procedure):

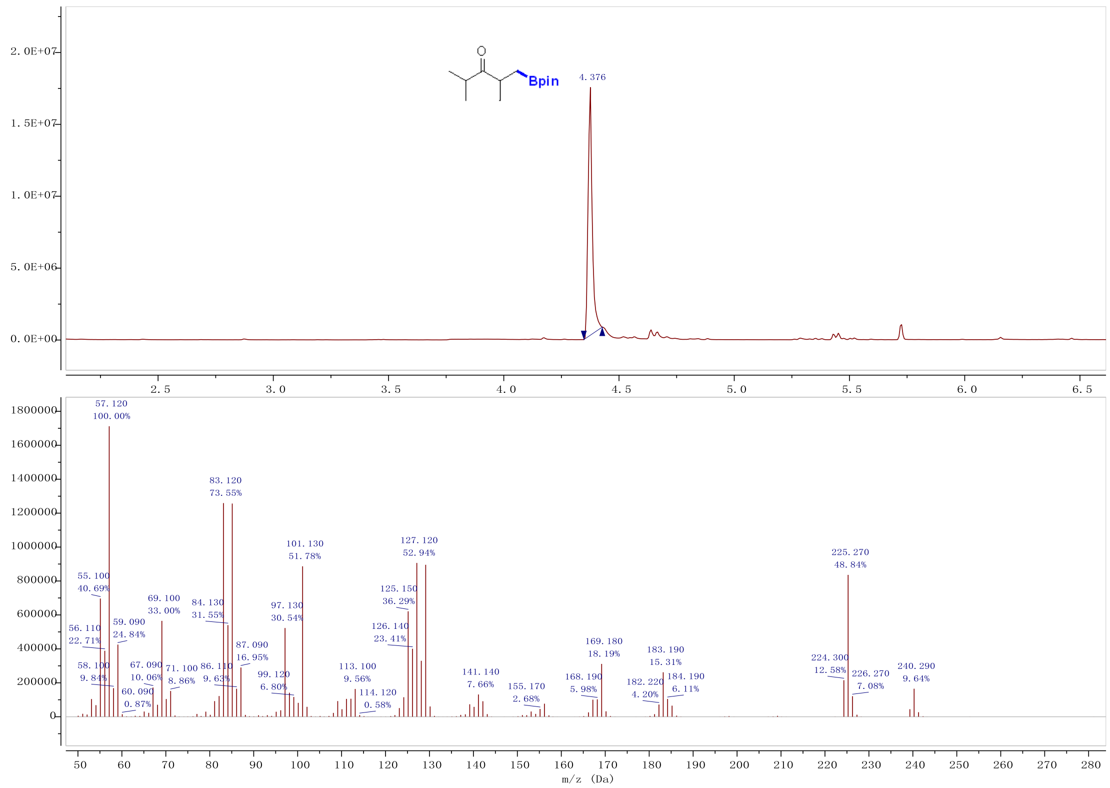

GC of **39** ([see procedure]()):

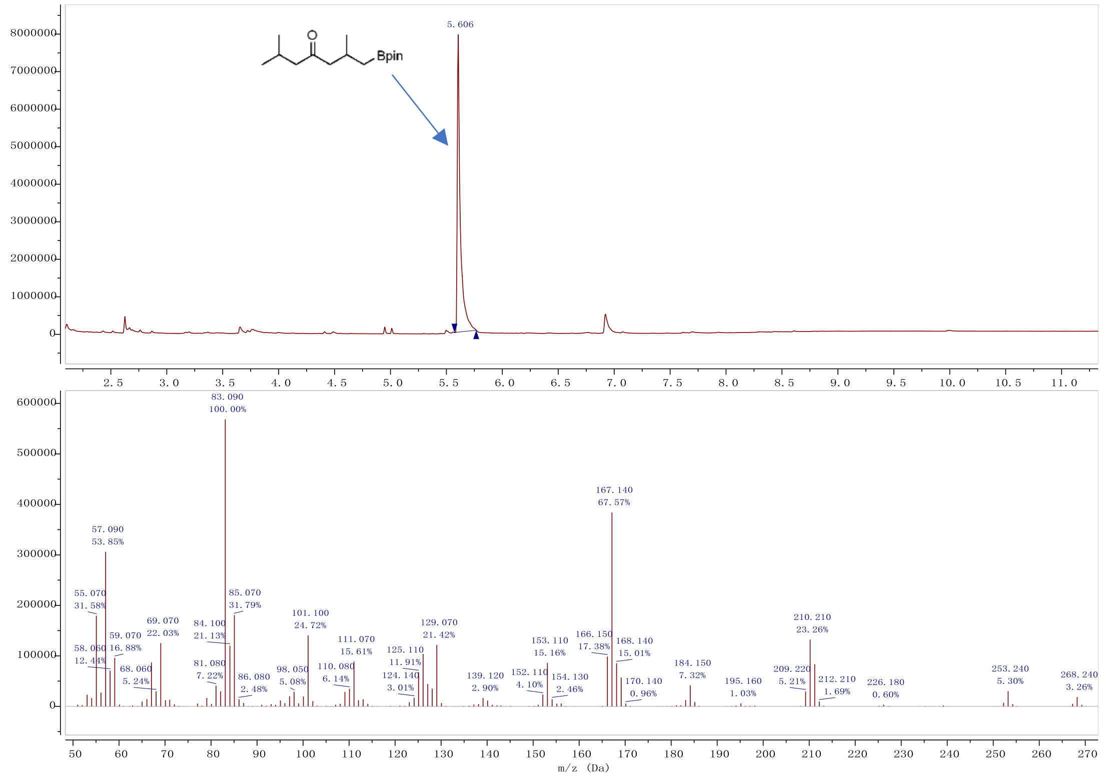

GC of **41** ([see procedure]()):

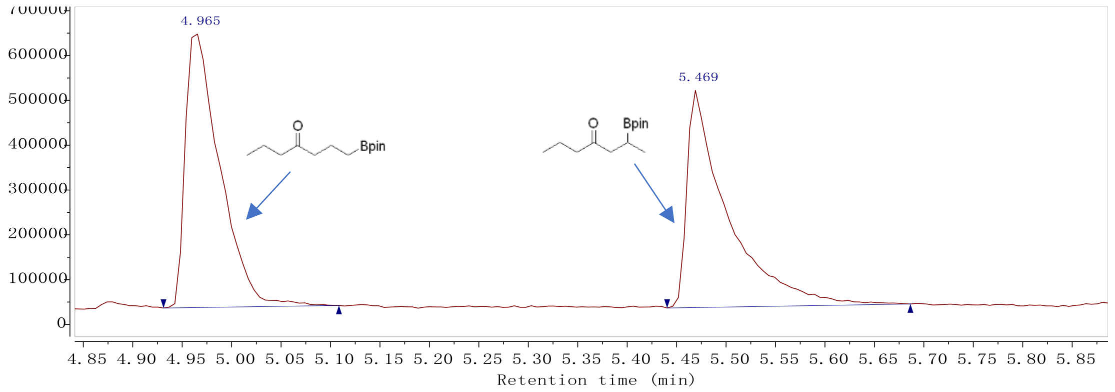

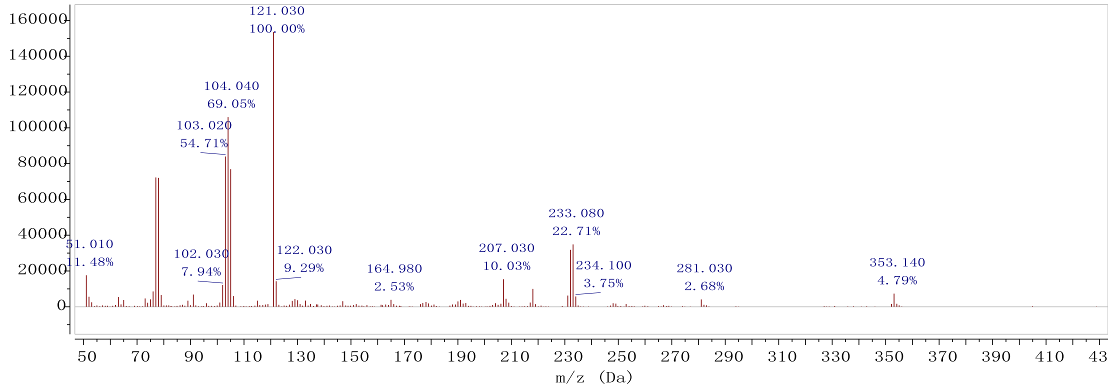

GC of **42** (see procedure):

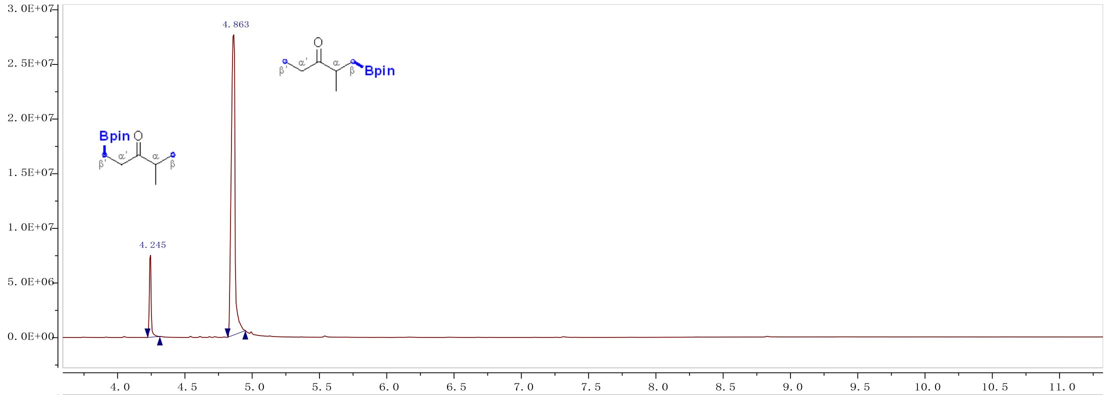

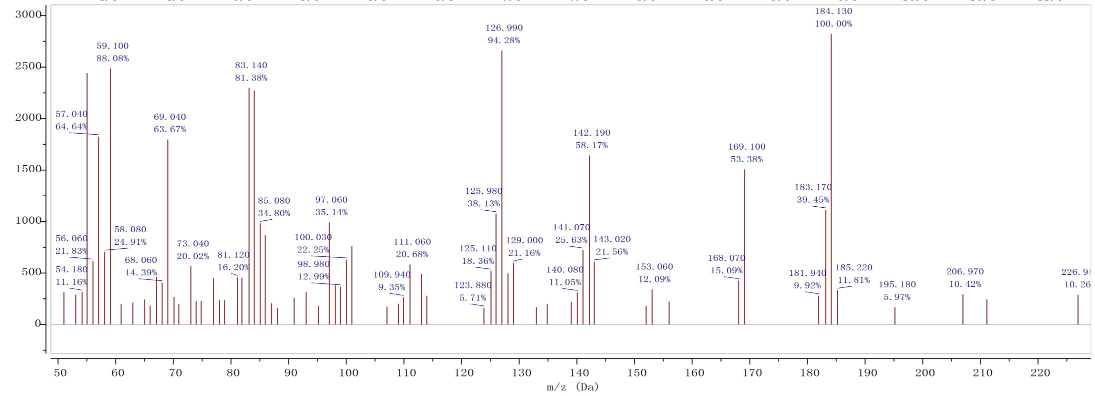

GC of **48** (see procedure):

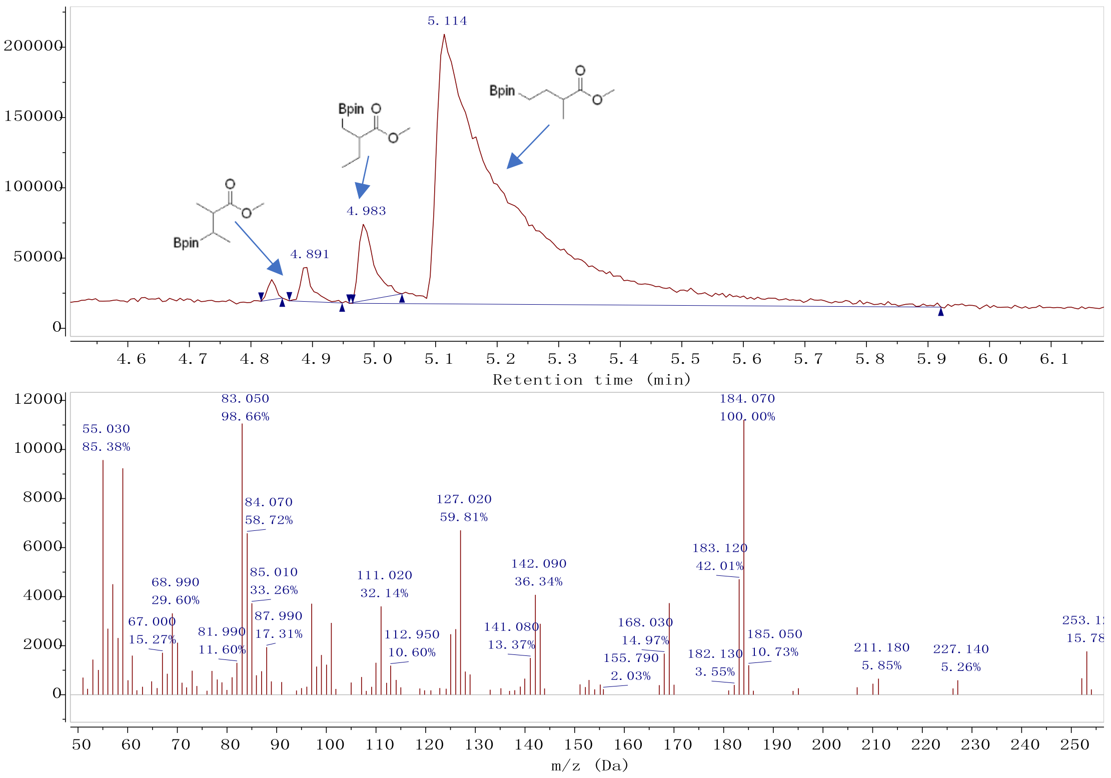

GC of **52** (see procedure):

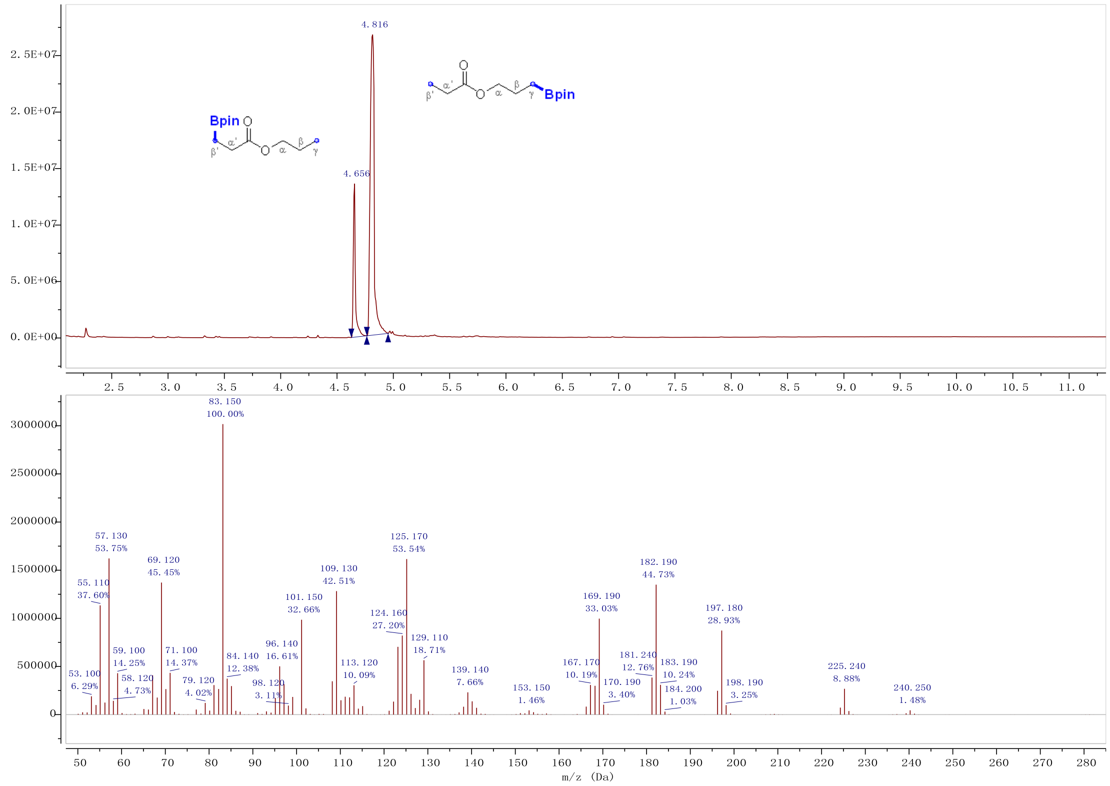

GC of **54** (see procedure):

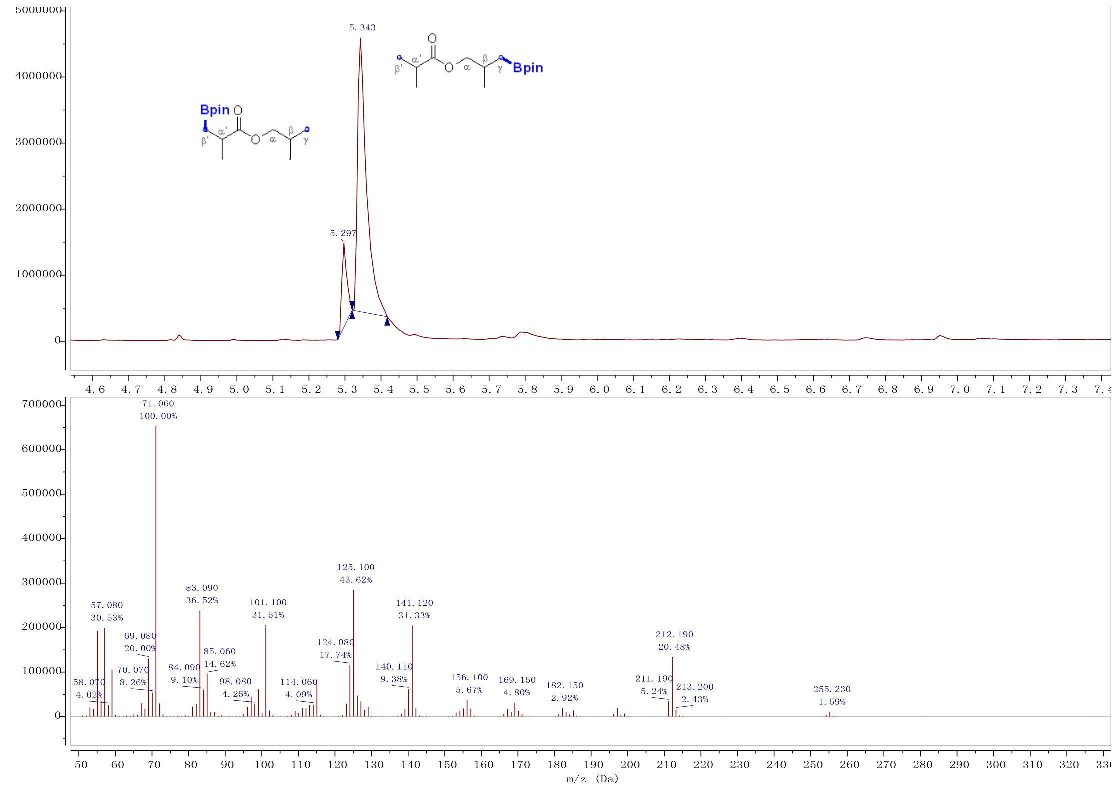

GC of **58** ([see procedure](#)):

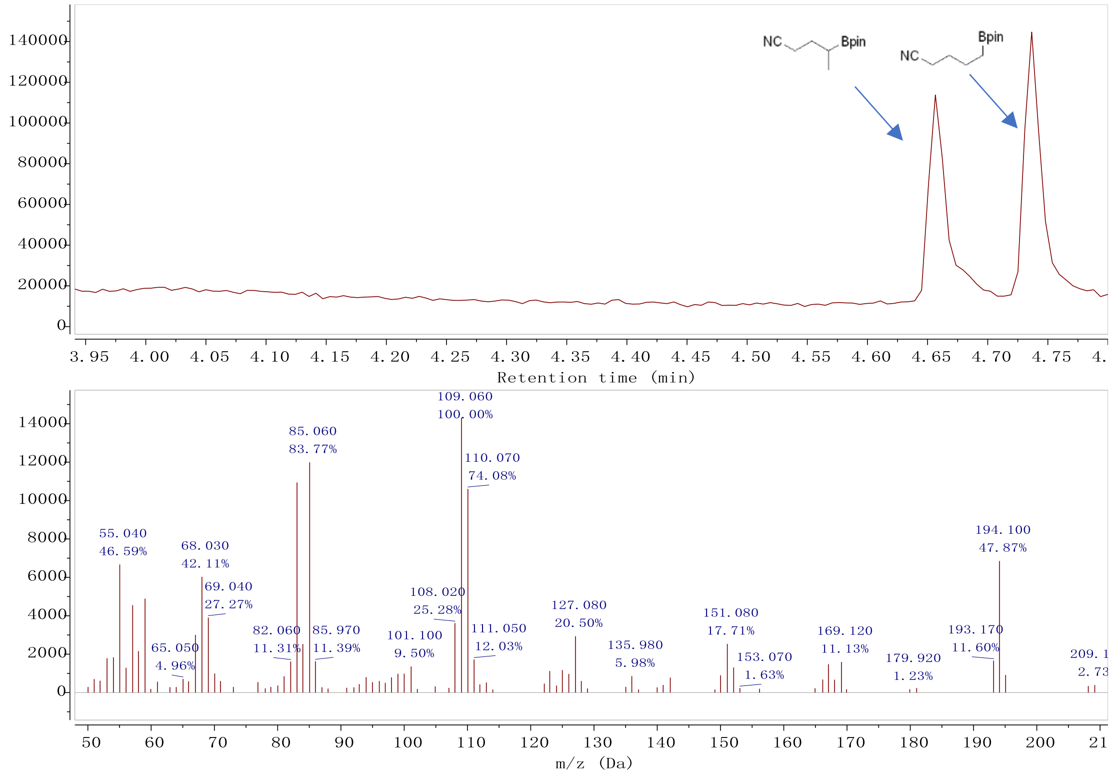

GC of **13** ([see procedure](#))

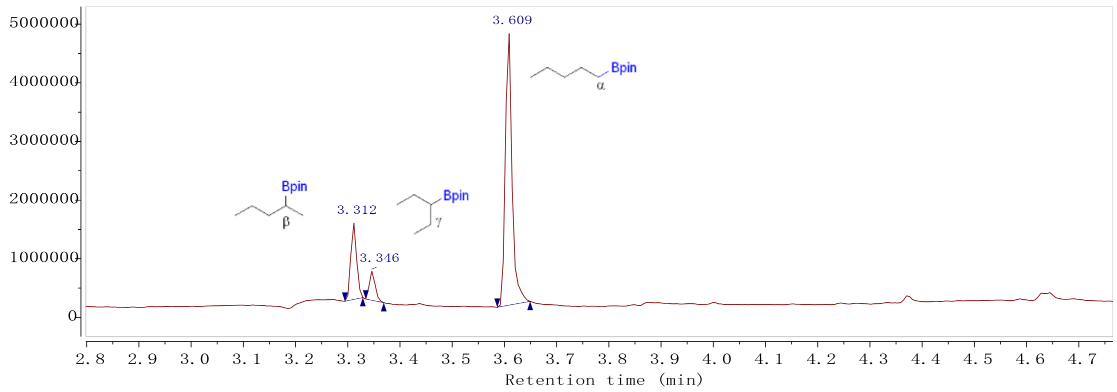
3.609
Bpin
α
3.312
3.346
β
γ
Retention time (min)

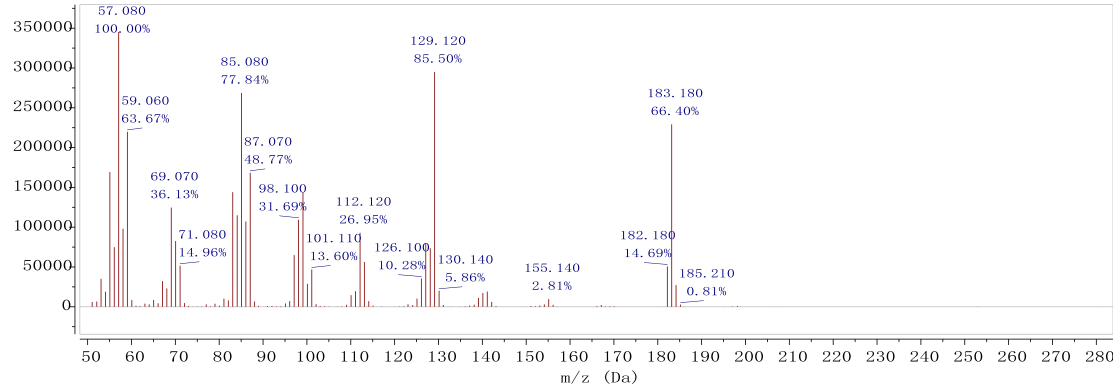
57.080 100.00%
59.060 63.67%
69.070 36.13%
71.080 14.96%
85.080 77.84%
87.070 48.77%
98.100 31.69%
101.110 13.60%
112.120 26.95%
126.100 10.28%
129.120 85.50%
130.140 5.86%
155.140 2.81%
182.180 14.69%
183.180 66.40%
185.210 0.81%
m/z (Da)

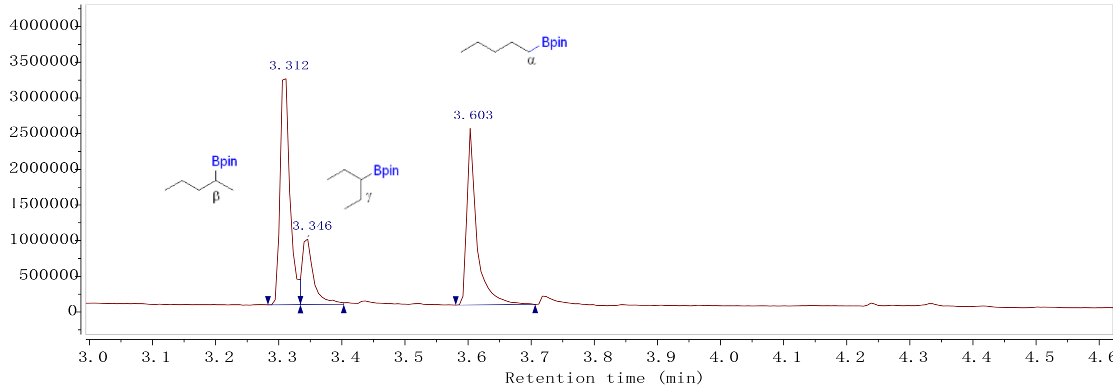
3.312
3.346
3.603
Bpin
α
β
γ
Retention time (min)

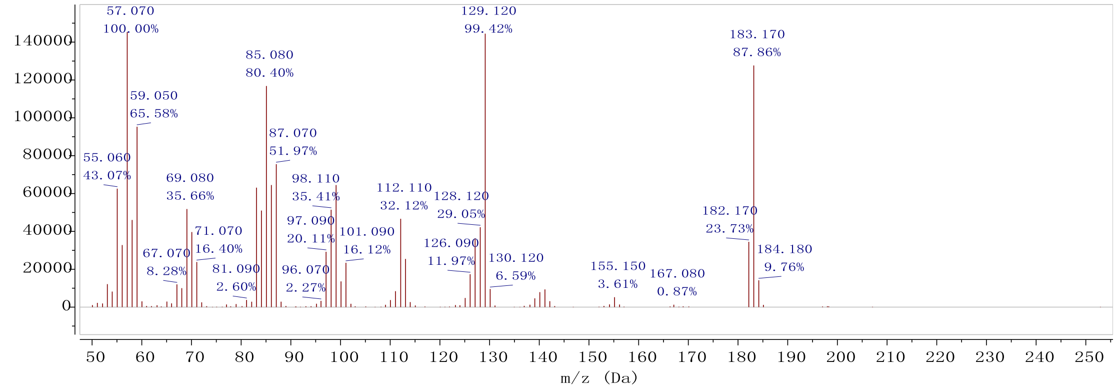
55.060 43.07%
57.070 100.00%
59.050 65.58%
67.070 8.28%
69.080 35.66%
71.070 16.40%
81.090 2.60%
85.080 80.40%
87.070 51.97%
96.070 2.27%
97.090 20.11%
98.110 35.41%
101.090 16.12%
112.110 32.12%
126.090 11.97%
128.120 29.05%
129.120 99.42%
130.120 6.59%
155.150 3.61%
167.080 0.87%
182.170 23.73%
183.170 87.86%
184.180 9.76%
m/z (Da)

6. Supplementary References

1. Fischer, S., Ward, T. R., & Liang, A. D. Engineering a Metathesis-Catalyzing Artificial Metalloenzyme Based on HaloTag. *ACS Catal.* **10**, 6343–6347(2021).
2. Paul, S., & Guin, J. Radical C(sp^3)–H alkenylation, alkynylation and allylation of ethers and amides enabled by photocatalysis. *Green Chem.* **19**, 2530-2534(2017).
3. Zhang, A., Ferguson, J. S., Yamato, K., Zheng, C., & Gong, B. Improving Foldamer Synthesis through Protecting Group Induced Unfolding of Aromatic Oligoamides. *Org. Lett.* **8**, 5117–5120(2006).
4. Mo, Z.-Y., Swaroop, R T., Tong, W., Zhang, Y.-Z., Tang, H.-T., Pan, Y.-M., Sun, H.-B., Chen, Z.-F. *Green Chem.*, **20**, 4428-4432 (2018).
5. He, M.-X., Mo, Z.-Y., Wang, Z.-Q. Cheng, S.-Y., Xie, R.-R., Tang, H.-T., Pan, Y.-M. *Org. Lett.* 22, 724-728 (2020**)**.
6. Zheng, L., Huang, H., Yang, C., Xia, W. UV light-mediated difunctionalization of alkenes through aroyl radical addition/1,4-/1,2-aryl shift cascade reactions. *Org. Lett.* **17**, 1034–1037 (2015)
7. Shu, C., Noble, A. & Aggarwal, V. K. Metal-free photoinduced C(sp^3)–H borylation of alkanes. *Nature* **586**, 714−719 (2020).
